# Systematic Preparation of a 66-IgG Library with Symmetric and Asymmetric Homogeneous Glycans and Their Functional Evaluation

**DOI:** 10.1021/jacs.4c06558

**Published:** 2024-08-06

**Authors:** Shino Manabe, Shogo Iwamoto, Satoru Nagatoishi, Asako Hoshinoo, Ai Mitani, Wataru Sumiyoshi, Takashi Kinoshita, Yoshiki Yamaguchi, Kouhei Tsumoto

**Affiliations:** † School of Pharmacy and Pharmaceutical Sciences and Institute of Medicinal Chemistry, Hoshi University, Ebara, Shinagawa-ku, Tokyo 142-8501, Japan; ‡ Research Center for Pharmaceutical Development, Graduate School of Pharmaceutical Sciences & Faculty of Pharmaceutical Sciences, Tohoku University, Aoba, Aramaki, Aoba-ku, Sendai, Miyagi 980-8578, Japan; § Fushimi Pharmaceutical Co., Ltd., Nakazu, Marugame, Kagawa 763-8605, Japan; ∥ Medical Device Development and Regulation Research Center, School of Engineering, The University of Tokyo, Hongo, Bunkyo-ku, Tokyo 113-0033, Japan; ⊥ Department of Bioengineering, School of Engineering, The University of Tokyo, Hongo, Bunkyo-ku, Tokyo 113-0033, Japan; # Institute of Molecular Biomembrane and Glycobiology, Tohoku Medical and Pharmaceutical University, Komatsushima, Aoba-ku, Sendai, Miyagi 980-8558, Japan

## Abstract

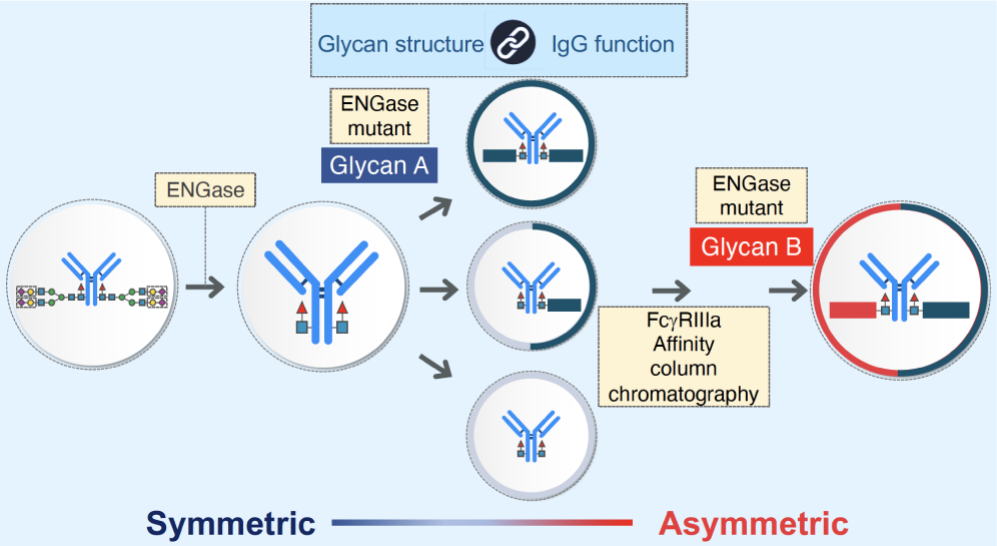

Immunoglobulin G (IgG) antibodies possess a conserved *N*-glycosylation site in the Fc domain. In FcγRIIIa affinity column chromatography, unglycosylated, hemiglycosylated, and fully glycosylated IgG retention times differ considerably. Using retention-time differences, 66 different trastuzumab antibodies with symmetric and asymmetric homogeneous glycans were prepared systematically, substantially expanding the scope of IgGs with homogeneous glycans. Using the prepared trastuzumab with homogeneous glycans, thermal stability and antibody-dependent cellular cytotoxicity were investigated. In some glycan series, a directly proportional relationship was observed between the thermal unfolding temperature (*T*_m_) and the calorimetric unfolding heat (Δ*H*_cal_). Antibody function could be deduced from the combination of a pair of glycans in an intact form. Controlling glycan structure through the combination of a pair of glycans permits the precise tuning of stability and effector functions of IgG. Overall, our technology can be used to investigate the effects of glycans on antibody functions.

## Introduction

Immunoglobulin G1 (IgG1) is the most widely used scaffold for the development of monoclonal antibodies for the treatment of human diseases. To block or stimulate signal transduction, specific antibodies perform their function by binding their antigen-binding Fab domains to target epitopes. By contrast, antibody-mediated effector functions are important for therapeutic antibodies. The expression of effector functions requires the binding of the crystallizable fragment (Fc) of IgG to Fcγ receptors (FcγRs), which are expressed on the surface of the recruited cells. IgG Fc is a symmetrical homodimer comprising the C-terminal half of Ig heavy-chain polypeptides, each with an N-terminal C_H_2 domain and a C-terminal C_H_3 domain. IgG carries a conserved *N*-glycan attached to each Asn297 residue in the C_H_2 domain. Owing to their complex biosynthetic pathways, *N*-glycans can occur in multiple compositions, resulting in over 400 variations.^1,2^*N*-glycosylation affects the thermal stability, conformation, aggregation, and effector functions of therapeutic antibodies. Nonhuman glycan structures in host cells can cause severe immunogenicity.^1−8^

To understand the contribution of each *N*-glycan component to the antibody effector function, homogeneous glycan IgGs were generated using a chemo-enzymatic approach.^9−14^ Previous studies have also investigated the relationship between glycan structure and antibody-dependent cellular cytotoxicity (ADCC) based on insights on structural biology using nuclear magnetic resonance (NMR).^15,16^

**Figure 1:**
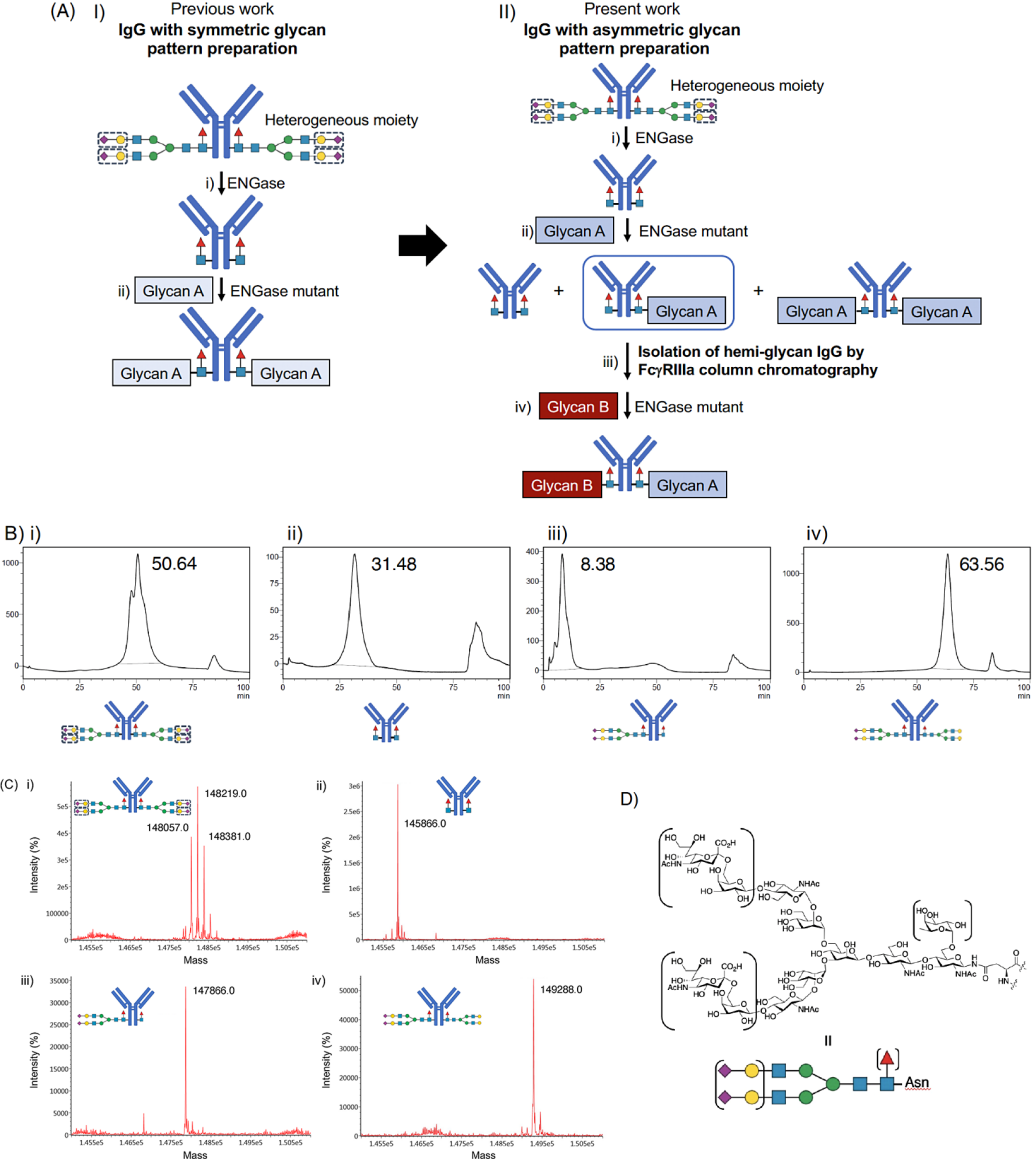
Schematic outline of glycan remodeling. (A) Schematic outline of IgG with a homogeneous glycan. I) Preparation of symmetric homogeneous glycan-carrying IgG; II) Preparation of asymmetric homogeneous glycan-carrying IgG. (B) FcγRIIIa-immobilized affinity column chromatography (TSKgel FcR-IIIA-5PW) HPLC profiles. (i) HPLC profile of commercially available trastuzumab; (ii) HPLC profile of glycan-truncated trastuzumab; (iii) HPLC profile of hemiglycan trastuzumab, glycan structure is SG; (iv) HPLC profile of asymmetric homogeneous trastuzumab, glycan structures are SG and G2. (C) ESI-MS profiles. (i) Deconvoluted ESI-MS profile of commercially available trastuzumab; (ii) Deconvoluted ESI-MS profile of glycan-truncated trastuzumab; (iii) deconvoluted ESI-MS profile of hemiglycan trastuzumab, glycan structure is SG; (iv) deconvoluted ESI-MS profile of asymmetric homogeneous trastuzumab; glycan structures are SG and G2. (D) Typical *N*-glycan structure in IgG. Heterogeneous parts are indicated in parentheses.

To prepare IgG with homogeneous glycans, glycan remodeling was performed in the following two steps: (i) removal of inherent heterogeneous glycans via hydrolysis of the β-1,4-glycosidic linkage within the core *N,N*′-diacetylchitobiose by endo-β-*N*-acetylglucosaminidase (ENGase) and (ii) addition of glycans by a combination of ENGase mutants and glycan oxazoline as a donor substrate (Figure 1A). ENGase mutants retain their glycan transfer activity, despite a decrease in hydrolytic activity. IgGs containing a pair of symmetrical glycans were prepared using this method. However, antibodies have glycan pairs with asymmetrical structures, contributing to the antibody diversity.

To elucidate the significance of the glycan structure in antibody function, the systematic preparation of homogeneous glycan-carrying IgGs, including asymmetric ones, is essential. A previous study attempted the desymmetrization of a pair of glycan using knobs-into holes technology, used for bispecific IgG, but the heterogeneity of glycan structures on each Fc region resulted in the formation of heterogeneous desymmetirc pair of glycans.^17^ Thus, in this study, we prepared an IgG library with asymmetric glycan pairs in a homogeneous form. We also evaluated the ADCC and thermal stability of IgGs with these glycan pairs. This study introduces a groundbreaking methodology for producing IgGs with asymmetrically homogenized glycans in their Fc regions. This is a significant advancement over previous technologies that were limited to symmetric homogenization. By enabling the production of IgGs with enhanced ADCC, our technique increases the diversity of glycan-modified IgGs by an order of magnitude. Additionally, we discovered a novel correlation between the thermal unfolding temperature and calorimetric unfolding heat. In the conventional method, where glycan analysis is performed after cleaving the glycans from IgG, two glycan structures emerge from a single glycan pair, making it impossible to deduce IgG glycan pairs (Figure S1).

## Results

### Preparation of a Library Containing 66 Trastuzumab Antibodies with Homogeneous Glycans

To develop a methodology to prepare IgG with asymmetric homogeneous glycans, trastuzumab was selected as the model IgG owing to its widespread use in the treatment of breast and gastric cancers. Heterogeneous glycans were removed using EndoS, an ENGase. Glycans can then be added to the glycan-truncated trastuzumab using EndoS D233Q and glycan oxazolines as donors (Figure 1B).^9−14^ Using FcγRIIIa-immobilized affinity column chromatography,^18−20^ we discovered that the retention times of glycan-truncated trastuzumab, hemiglycan trastuzumab, and full-glycan trastuzumab were 8.4, 30–31, and 51–64 min, respectively, which were significantly different (Figure 1B and Table 1). Taking advantage of the different retention times in FcγRIIIa affinity column chromatography, hemiglycosylated trastuzumab was isolated. Then, the second glycan, which has a structure different from that of the first, was added to the isolated hemiglycosylated trastuzumab to produce IgG with asymmetric glycans. Thus, IgG with an asymmetric homogeneous glycan can be prepared as follows: (i) removal of a heterogeneous moiety at the β-1,4-glycosidic linkage within the *N,N*′*-*diacetylchitobiose core of *N*-glycans; (ii) partial addition of glycan oxazoline to the glycan-truncated IgG by the ENGase mutant; (iii) isolation of hemiglycan IgG by FcγRIIIa affinity column chromatography; and (iv) addition of another type of glycan oxazoline to hemiglycan IgG to produce an asymmetric IgG. The homogeneity of the prepared trastuzumab was confirmed using mass spectrometry (MS) in each step (Figure 1C). Based on this methodology, 66 trastuzumab antibodies with homogeneous symmetric and asymmetric glycans were systematically prepared (Table 1).

**Table 1:**
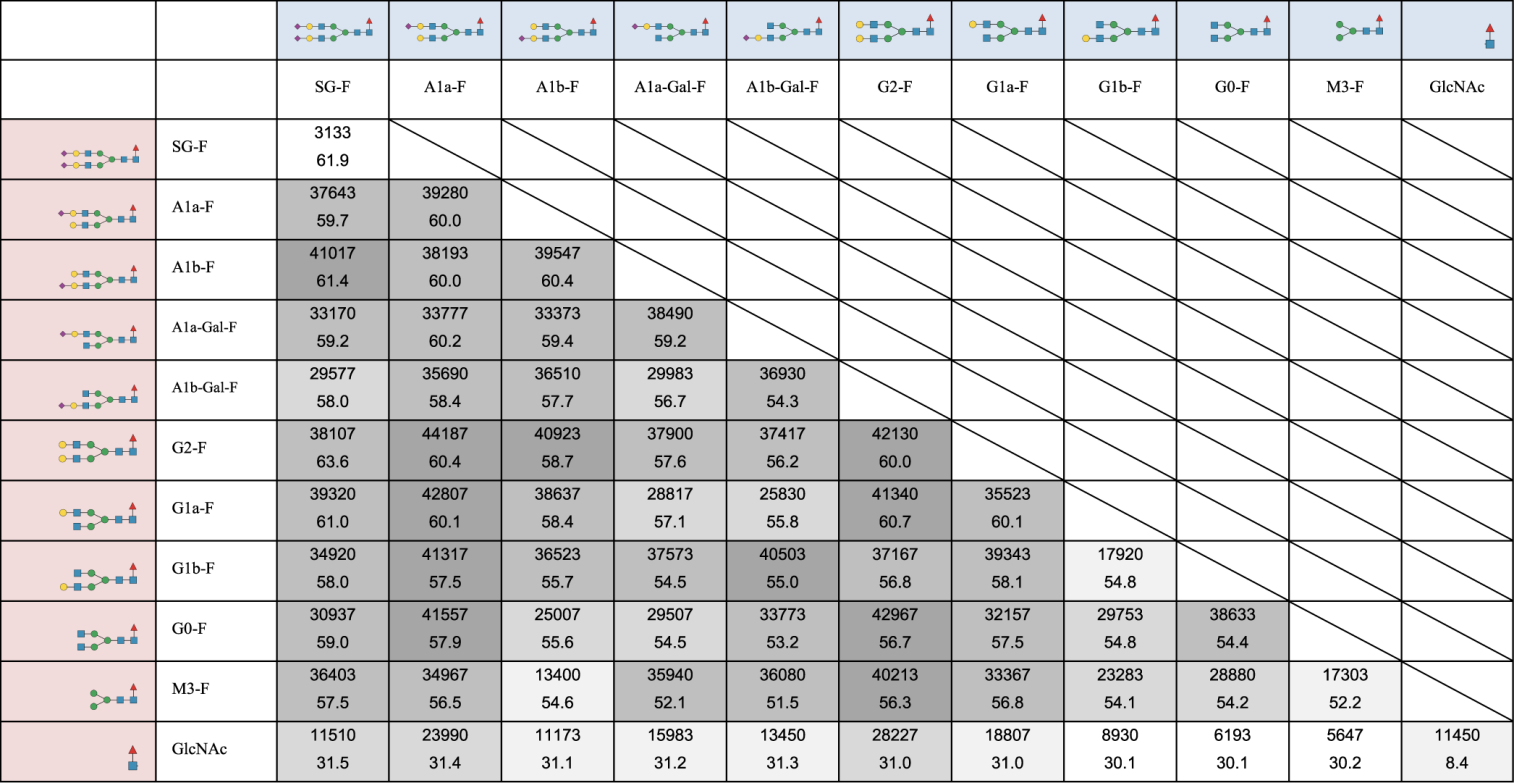
Relative Luminous Intensity and Retention Time of Glyco-Engineered Trastuzumab in FcγRIIIa-Immobilized Column Chromatography^a^^b^^c^

a The upper row represents the structure of glycan A.

b The left column represents the structure of glycan B.

c In each cell, the numbers in the upper row indicate cell-based ADCC (n = 3), and the numbers in the lower row indicate retention time (min) in FcγRIIIa affinity column chromatography.

### Higher ADCC Activity was Seen in Trastuzumab with Asymmetric Glycosylation Than in Trastuzumab with Symmetric Glycosylation

Glycosylation in the Fc region modifies the antibody effector function by affecting its binding affinity for FcγRs. Upon binding, the hinge rearranges asymmetrically, excluding the second molecule of the FcγR from binding. Thus, the Fc region of the antibody interacts with the FcγR at a 1:1 ratio.^21^ Asymmetrically engineered antibodies with amino acid mutations exhibit high ADCC.^22−24^ The best-known relationship between the glycan structure and IgG effector function is demonstrated through ADCC. Glycan structure desymmetrization would affect ADCC.

Hemiglycosylated IgG have been characterized, although in a small population, and their binding affinities to FcγRs are significantly reduced compared to that of IgG with two glycans.^25^ Optimized and well-characterized assays are essential for accurate IgG function analysis to meet specific needs. When considering the specific applications of the ADCC assay, it is crucial to understand the advantages and limitations of the assay as they can be affected by factors such as cell types and assay formats. A previous study measured ADCC mediated by NK cells using IgG with a glycan repertoire.^26^ Both NK and Jurkat T effector cells utilize the ADCC pathway immediately after FcγRIIIa binding, leading to dephosphorylation of pNFAT2,^27^ its transport into the nucleus, and activation of gene expression. The sensitivity of Jurkat T cell is similar to that of original NK cells, and it is widely used for ADCC measurement.^28−31^ This assay is often employed for ADCC measurements with glycan structures. Based on previous studies,^32,33^ we measured ADCC using a cell-reporter assay on Jurkat T cells that express FcγRIIIa. Hemitrastuzumab had a significantly lower ADCC (Table 1). Asymmetric glycan-carrying trastuzumab (A1a-F/G2-F, G2-F/G0-F, A1a-F/G1a-F, A1a-F/G0-F, G2-F/G1a-F, A1a-F/G1b-F, SG-F/A1b-F, A1b-F/G2-F, A1b-G-F/G1b-F, and G2-F/M3-F) showed ADCC similar to that of G2-F/G2-F, with the highest ADCC observed for symmetric glycan.

While the positive impact of galactosylation has been well established,^26^ many studies reported varying effects of galactose residue numbers on ADCC, including upregulation, no effect, or even downregulation.^9,34−36^ This ambiguity may arise because of the high diversity of glycans and the lack of direct glycan structural analyses on IgG. After cleavage of glycans from IgGs, the conventional method cannot distinguish between the individual glycan chains in the pair. In addition, the glycan structure differs according to the batch, even for therapeutic IgG in clinical use.^37^ Our results showed a correlation between the number of galactose residues and ADCC. As the number of galactose residues increased, ADCC increased. Namely, more effective ADCC was seen with the G2 series than with the G0-F series, G1-F series, A1a-Gal-F series, and A1b-Gal-F series. Furthermore, the G1a-F series demonstrated more effective ADCC than that of the G1b-F series, although having the same number of sugar residues.^38^ When we compared the A1a-F series with the A1b-F series, a similar tendency was observed. In most cases, the A1a-F series had a higher ADCC than did the A1b-F series.

**Figure 2:**
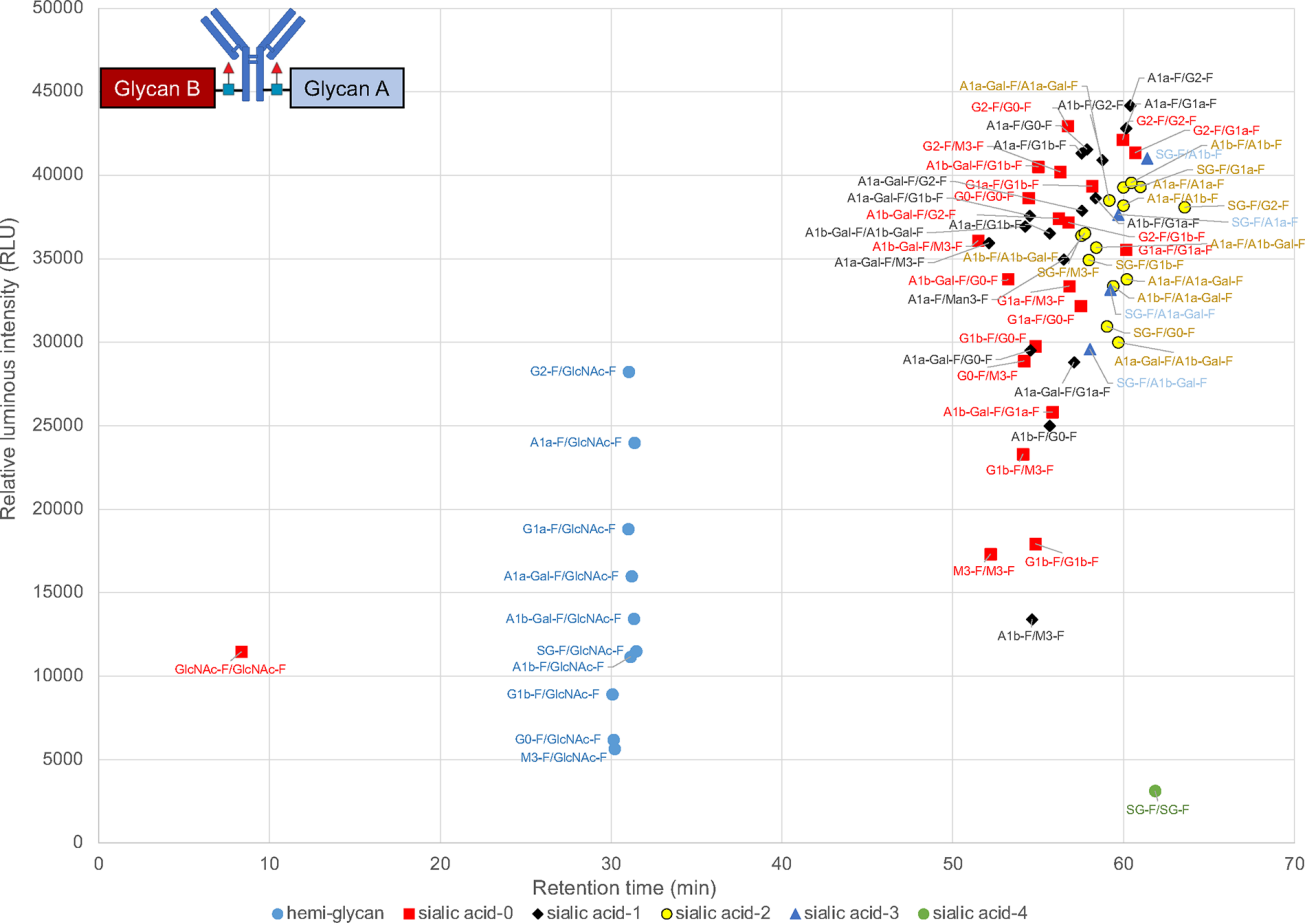
Scatter plot of the relationship between retention time in FcγRIIIa column chromatography and ADCC. Blue-circle dots: trastuzumab with hemiglycan; Red-square dots: trastuzumab without sialic acid; black-diamond dots: trastuzumab with one sialic acid residue; yellow-circle dots: trastuzumab with two sialic acid residues; blue triangle dots: trastuzumab with three sialic acid residues; green circle dot: trastuzumab with four sialic acid residues.

In our experiments, FcγRIIIa affinity column chromatography retention times and ADCC were roughly correlated (Figure 2, for expansion of Figure S2).^18−20^ As the size of the glycans increased, the retention time of the column tended to increase. The lack of strict correlation alignment with strict precision between ADCC and retention time may be because the FcγRIIIa used in affinity column chromatography has no glycan and eight amino acid mutations.^18,39,40^ The other possibility is that cell-based ADCC reporter assay was the integrated result of other interactions with FcγR, including FcγRIIb, which has an inhibitory function.

As previously reported,^18−20^ the G0-F series were dominant in the short retention time fraction, and the G1-F and G2-F series were included in the long retention time fraction on FcγRIIIa column chromatography using antibodies with heterogeneous glycans. Importantly, FcγRIIIa affinity column chromatography recognized a glycan branching pattern. Namely, the Man α1–6 arm (A1a-F, A1a-Gal-F, and G1a-F) series had longer retention times than did the corresponding Man α1–3 arm series (A1b-F, A1b-Gal-F, and G1b-F) in correlation with ADCC. The Man α1–6 arm (A1a-F and G1a-F) series has more effective ADCC than does the Man α1–3 arm series (A1b-F and G1b-F). The retention-time difference may be influenced by the whole IgG structure caused by glycan. The Man α1–3 arm may be more dynamic than the Man α1–6 arm.^41−43^ The mannose residue in the Man α1–6 arm in G1a interacts with the inner surface of the C_H_2 elbow region, and multiple interactions between the glycan components of the Man α1–6 arm and protein portions of an IgG affect the suitable conformation to bind FcγRIIIa. According to previous reports,^41−46^ the galactose residue of the Man α1–6 arm interacts with Lys 250 in the C_H_2 domain, and CH-π interactions occur between the Manα1–6 arm and two Phe residues (Phe 245 and Phe 247).

Reportedly, *N*-acetylneuraminic acid exerts both a positive and negative effect on ADCC.^9,10,47^ Although the A1a-F series showed a high ADCC, in general, the range in ADCC values reduced when the number of *N*-acetylneuraminic acid residues was more than 2. The A1a-F series tended to show higher ADCC than the A1b-F series.

### Direct Linear Correlation between *T*_m_ and Δ*H*_cal_ in Some Glycan Series

The structural stability of the prepared trastuzumab was evaluated in terms of the thermal unfolding temperature (*T*_m_) and calorimetric unfolding heat (Δ*H*_cal_) using differential scanning calorimetry (DSC; Table 2). In the DSC spectra, the peak below 75 °C corresponded to the C_H_2 domain of the Fc domain and the peak above 75 °C corresponded to the Fab domain. Each *T*_m_ value was considered to be the denaturation midpoint temperature derived from the C_H_2 domain. The melting transitions of each glycan-engineered IgG were indicative of the unfolding of the C_H_2 domains. We observed a relative correlation between *T*_m_ and Δ*H*_cal_; that is, antibodies with higher *T*_m_ tended to have relatively negative Δ*H*_cal_ values (Table 2). The *T*_m_ of glycan-truncated trastuzumab was substantially low at 64.9 °C. Trastuzumab antibodies with hemiglycan structures tended to have a lower *T*_m_ than those with two glycans, distributed between 66.3 and 68.7 °C. The addition of GlcNAc and galactose residues increased the stability. The addition of one sialic acid residue increased the *T*_m_ values and decreased Δ*H*_cal_; however, the *T*_m_ values tended to be low when the number of sialic acid residues was more than two. When IgG had one sialic acid residue and one glycan was short, both *T*_m_ values and Δ*H* values were significantly lowered (A1a-Gal-F/GlcNAc-F, A1a-Gal-F/Man3-F, A1b-Gal-F/GlcNAc-F, A1b-Gal-F/M3-F, A1b-F/GlcNAc-F, and A1b-F/M3). The same phenomenon was observed in the sialic-acid-free trastuzumab series, with G1a-F/M3-F, G2-F/GlcNAc-F, G1b-F/GlcNAc-F, and G0-F/GlcNAc-F showing lower *T*_m_ and Δ*H* values.

**Table 2:**
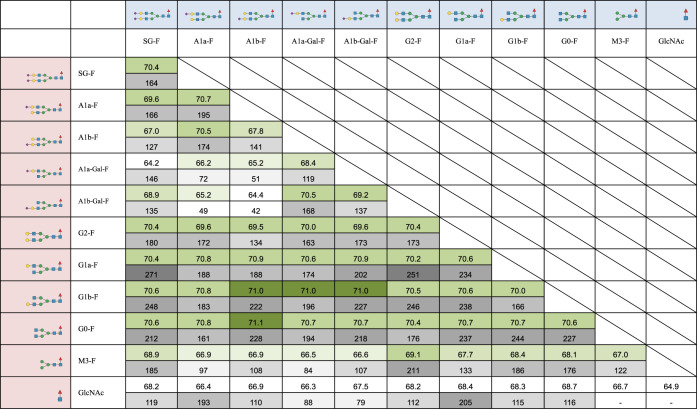
*T*_m_ and Δ*H*_cal_ of Glycoengineered Trastuzumab^a^^b^^c^

a The upper row represents the structure of glycan A.

b The left column represents the structure of glycan B.

c In each cell, the numbers in the upper row indicate *T*_m_ (°C), and the numbers in the lower row indicate Δ*H*_cal_ (kcal/mol).

**Figure 3:**
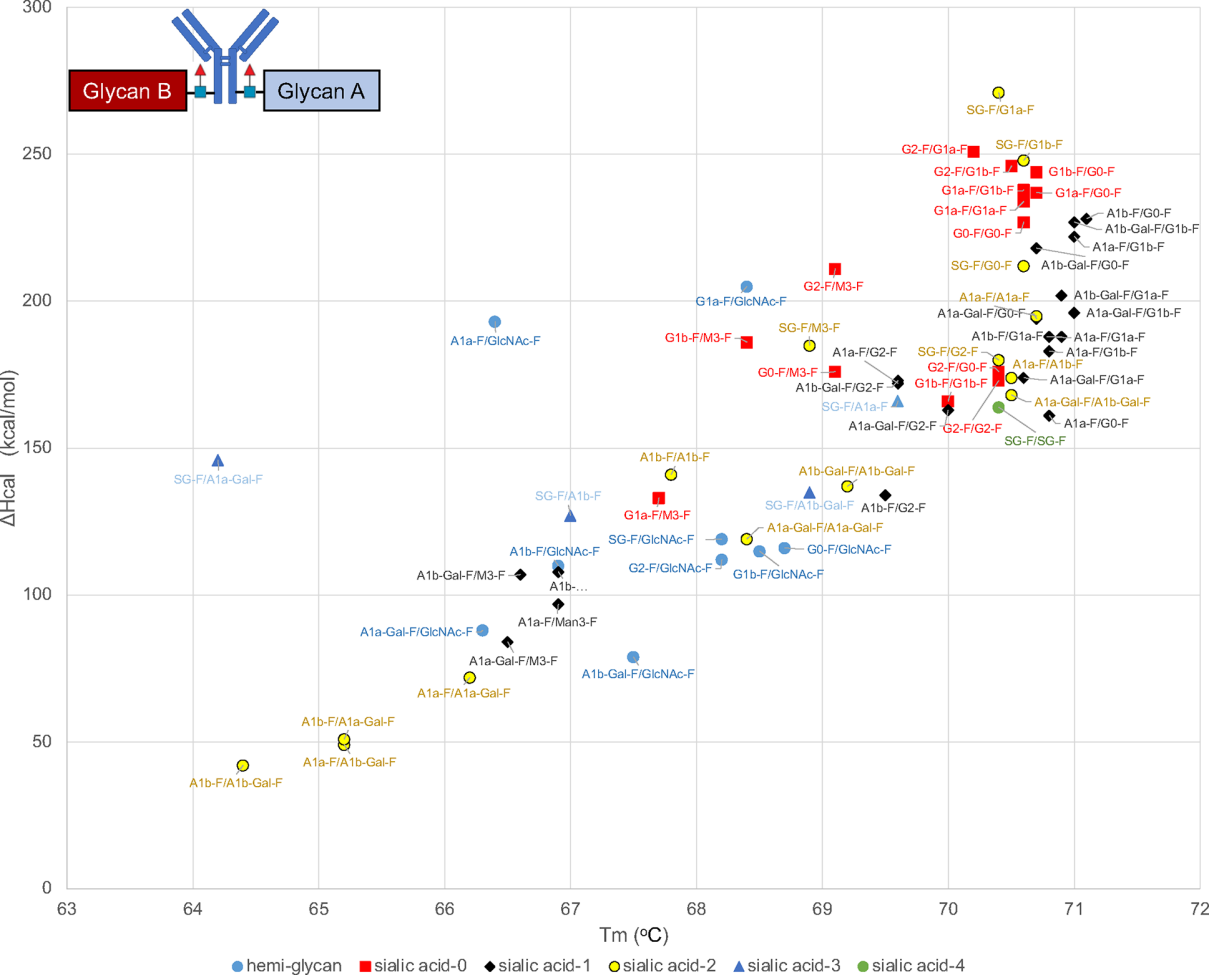
Scatter plot of the relationship between *T*_m_ and Δ*H*_cal_. Blue-circle dots: trastuzumab with hemiglycan; red-square dots: trastuzumab without sialic acid; black-diamond dots: trastuzumab with one sialic acid residue; yellow-circle dots: trastuzumab with two sialic acid residues; blue triangle dots: trastuzumab with three sialic acid residues; green circle dot: trastuzumab with four sialic acid residues.

**Figure 4:**
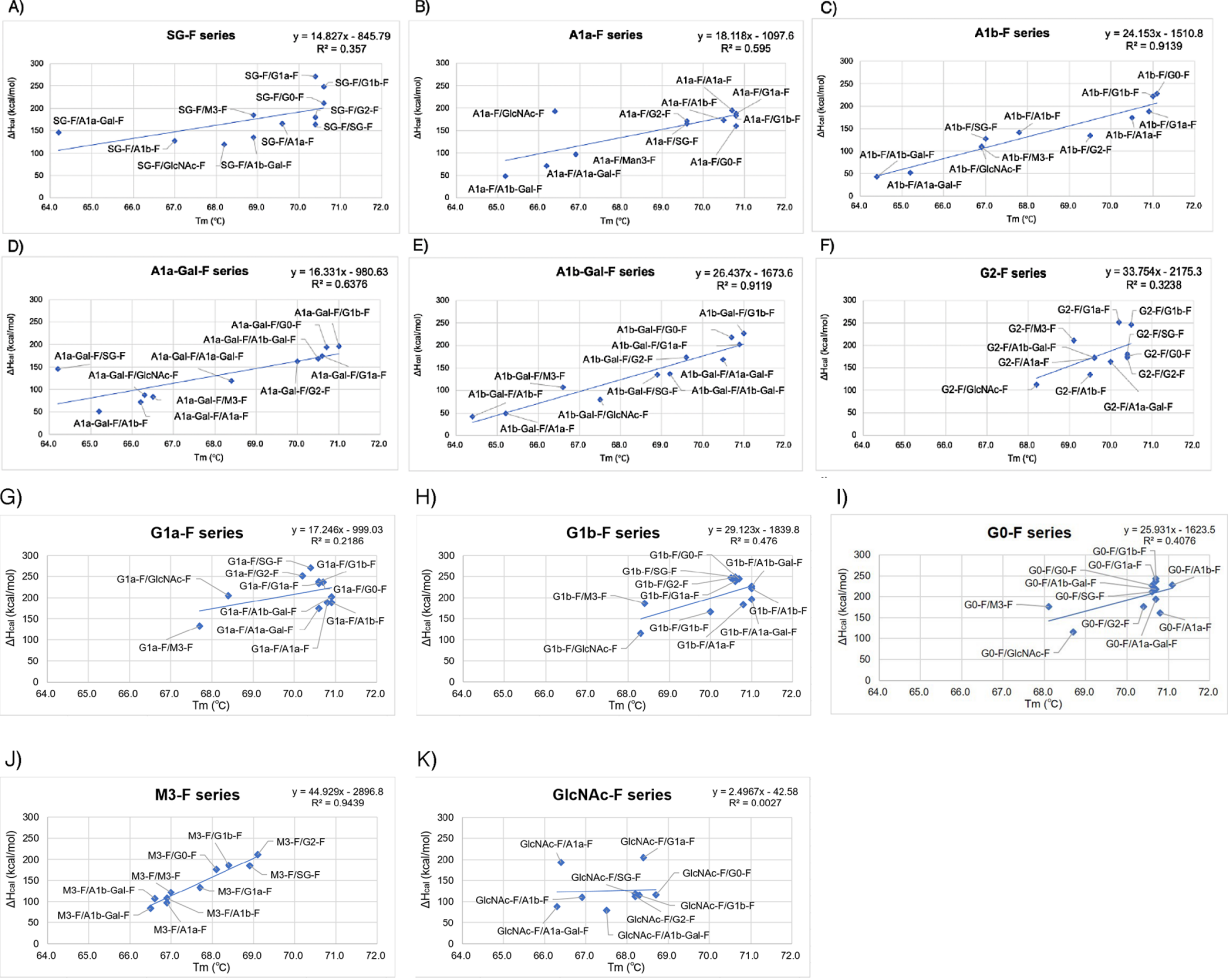
Correlation between *T*_m_ and Δ*H*_cal_. (A) SG-F series, (B) A1a-F series, (C) A1b-F series, (D) A1a-Gal-F series, (E) A1b-Gal-F series, (F) G2 series, (G) G1a series, (H) G1b series, (I) G0-F series, (J) M3-F series, (K) GlcNAc-F series, (B′) A1a-F series without GlcNAc, and (D′) A1a-Gal-F series without SG.

To clarify the effect of the glycan structure on thermal stability, one of the glycan structures in the pair of glycans was fixed and the correlation between *T*_m_ and Δ*H*_cal_ was examined (Figures 3 and 4). A strong direct linear correlation between the *T*_m_ and Δ*H*_cal_ values of trastuzumab with the A1b-F, A1b-Gal-F, and M3-F glycan series was observed (Figure 4C,E and J; *R*^2^ = 0.914, 0.912, and 0.944, respectively). When the A1a series was fixed as one glycan, a correlation was observed when the hemiglycosylated trastuzumab was eliminated (Figure 4B′; *R*^2^ = 0.954). Similarly, when A1a-Gal-F was fixed as one glycan, a correlation was observed when trastuzumab with the SG-F glycan was eliminated (Figure 4D′; *R*^2^ = 0.981). Moreover, in the linear relationship between *T*_m_ and Δ*H* for A1b-F, A1b-Gal-F, and A1a (without the hemiglycosylated form), the range of change in *T*_m_ values tended to be broader than that for M3-F. The M3-F series exhibits narrower *T*_m_ and Δ*H* ranges. Thermal stability and local fluctuation changes are involved in the stability changes of the C_H_2 domain.^41−46^ Therefore, asymmetric glycan antibodies with sialic acid have been suggested to lead to significant changes in the thermal stability and movement of C_H_2 in conjunction with each other. The IgG Fc crystal structure revealed that the oligosaccharide is integral to the Fc structure and sequestered in the internal space enclosed by the two C_H_2 domains.^18^ Thus, clarifying the reason for the impact of sialic acid on the thermal stability is imperative. The influence of sialic acid might be attributed to high dynamic properties and/or the charge of the carboxylic acid.^41−46^

The existence of asymmetric fluctuations in the C_H_2 region of antibodies has been recently reported.^6^ The results of this study strongly suggest that glycan asymmetry plays an important role in controlling fluctuations in the C_H_2 region. However, no correlation between the *T*_m_ and Δ*H*_cal_ values was observed in the other glycan series.

## Discussion

Because of their ability to quickly and efficiently measure ADCC *in vitro*, FcγRIIIa-expressing natural killer cells are classically believed to be the predominant cell type responsible for mediating ADCC *in vivo*, and the glycan structure of IgG was focused on ADCC. However, recent reports on the mechanism of tumor-targeted antibody-mediated depletion in preclinical models have shown that activating FcγR-expressing monocytes and macrophages are the key cell types responsible for cytotoxicity against tumor cells *in vivo.*(48,49) Using our prepared homogeneous glycan IgG library, interaction with other FcγRs could be measured.

In our study, a simple rule between the glycan structure and IgG function has not yet been found. To predict IgG function from a glycan structure point of view, further investigation is necessary, especially on interactions with Fab and FcγRs.^43,50^ We must understand how the glycan structure affects the conformation of the Fc domain of IgG, leading to alterations in its binding affinity to FcγRs. Glycans play a key role in maintaining the structure of the Fc region, particularly the C_H_2 domain. The effect of glycosylation on the higher-order open/closed conformation of the Fc region has been reported, although a consensus has not yet been achieved.^51,52^ These interactions reduce the fluctuation of glycans and proteins, helping preorganize the interaction surface with FcγRs.^53^ The removal of glycan results in a closed conformation of C_H_2, and binding ability to FcγRIIIa is decreased.^41^ Although glycan reportedly affects the open-closed conformation of Fc and its ability to bind to FcγRs, the openness of the Fc domain may not necessarily be directly correlated with effector functions. For instance, although IgG with the Q295F/Y296A mutation increased thermal stability, Fc receptor binding was substantially decreased.^54^ We believe that the impact of “a pair of glycans” on IgG function requires further investigation, as its function does not solely result from the additive property of the glycan structure. In other words, the function of IgG with glycans A and B does not reflect the average of IgG with two glycans As and IgG with two glycan Bs.

We also found that the thermostability of IgG was influenced by the glycan structure, and a strong direct linear correlation between *T*_m_ and Δ*H*_cal_ was observed in some glycan series. The significance of this relationship remains unclear. To comprehensively elucidate the relationship, further structural biology analyses, such as crystallography and NMR analysis, are necessary, including molecular dynamics of IgG with asymmetric glycan.

## Conclusion

We demonstrated a methodology for preparing IgG with asymmetric homogeneous glycans. To the best of our knowledge, for the first time, we prepared a library containing more than 10 IgGs with homogeneous glycans and demonstrated a marked increase in the scope of homogeneous glycan IgGs. Before our study, the number and pattern of IgG with homogeneous glycans were limited. Moreover, the debate about the relationship between glycan structure and IgG functions has been relatively limited and primarily focused on the number of sugar residues involved. Our technology can help overcome these limitations and open a novel avenue for investigating the glycan structure–IgG function relationship. Because IgG has a pair of glycans, understanding the combination of glycan structure is important for antibody functions. Laboratory-prepared homogeneous glycosylated IgG, ranging in quantity from a few milligrams to 10 mg, is adequate for biological investigations.

Although a large ADCC improvement (e.g., 50- or 100-fold), has been achieved by core-fucose removal,^40,55^ fine-tuning of ADCC is possible using our methodology. Recently, the chemical functionalization of glycans in IgG was reported to enhance ADCC.^56^ Glycan modification of IgG using natural and artificial glycans provides a novel strategy for attenuating the Fc function and increasing the thermal stability of IgG. Protein engineering of the Fc domain of antibodies mainly involves amino acid mutations to attenuate binding to FcγR to develop efficient therapeutic antibodies.^57^ We believe that a combination of our glycan technology and point mutations of amino acids can be applied for efficient antibody development. In addition, glycan structure alters *in vivo* interactions with FcγRs to change the pharmacodynamics of IgG.^58,59^ The applicability of our methodology to other antibodies, structural biology investigations, and *in vivo* activities will be described in due course. These findings not only expand the toolkit for antibody engineering but also open new avenues for developing therapeutics with improved efficacy and stability.

## Materials and Methods

### General

TSKgel FcR-IIIA-NPR (4.6 mm I.D. × 7.5 cm) and TSKgel FcR-IIIA-5PW (7.8 mm I.D. × 7.5 cm) columns from TOSOH (Tokyo, Japan) were used for the analysis of all samples. Protein A column chromatography (TOYOPEARL AF-rProtein A HF-650F SkillPak = 1 mL) was obtained from TOSOH. HPLC (Prominence, SHIMADZU, Kyoto, Japan) was used for analyses and purification. Antibody concentration was measured using a NanoDrop Lite (Thermo Fisher Scientific, Waltham, MA, USA). 2-Chloro-1,3-dimethyl-1H-benzimidazol-3-ium chloride, used for preparing oxazoline, was purchased from Fushimi Pharmaceutical Co., Ltd. (Marugame, Japan). Glycans were procured from Fushimi Pharmaceutical Co., Ltd. and Glytech, Inc. (Kyoto, Japan). EndoS was purchased from New England BioLabs Japan (Tokyo, Japan). EndoS D233Q was prepared according to a previous study.^60^

Sephadex G-25 was purchased from Cytiva (Tokyo, Japan) for the purification of glycan oxazoline. ^1^H NMR spectra of oxazolines were measured on a JEOL (Akishima, Japan) JMN-ECZL spectrometer (400 MHz) in D_2_O at ambient temperature (20–25 °C).

### Preparation of Glycan Oxazoline

To a solution of glycan hemiacetal (20 mg) and Et_3_N (80 μL) in D_2_O (1 mL) was added 2-chloro-1,3-dimethyl-1H-benzimidazol-3-ium chloride (21.5 mg) was added. The consumption of hemiacetal was monitored by employing ^1^H NMR, and oxazoline was purified using size exclusion chromatography (Sephadex G-25) with H_2_O containing 0.1% Et_3_N. The product was confirmed in D_2_O using ^1^H NMR.^61^

### FcγRIIIa Affinity Column Chromatography Analysis

Mobile phase A was composed of 50 mM citrate at pH 6.5, and mobile phase B was composed of 50 mM citrate at pH 4.5. Trastuzumab was dissolved in mobile phase A. A linear gradient of buffer B (0 to 100%) at a flow rate of 1 mL/min was applied to the column for 20 min to elute the IgG in a Shimadzu purifier system with a fluorescence detector, RF-20A_XS_ (excitation 280 nm, emission 348 nm). The gradient was started at 2 min. Samples (50 μL) were injected into the sample loop at a column temperature of 25 °C.

### FcγRIIIa Affinity Column Chromatography Purification

Elution time and injection volume differed from the conditions in the FcγRIIIa affinity column chromatography analysis, but the other conditions were the same. A flow rate of 1 mL/min was applied to the column for 45 min to elute the IgG. For purification, samples (2 mL) were injected into the sample loop.

### Removal of *N-*Glycan on IgG Using EndoS

Trastuzumab (10 mg/mL in 50 mM sodium citrate buffer, pH 6.5) was incubated with 30 μg of EndoS at 30 °C for 16 h. The reaction was monitored using a TSKgel FcR-IIIA-NPR column chromatography. The product was purified using a protein A column and HPLC. The product was loaded into the column with a buffer of 50 mM sodium phosphate at pH 6.5 and eluted with a buffer of 50 mM citrate at pH 3.0, 25 °C, and a flow rate of 1 mL/min.

### General Procedure for the Addition of *N*-Glycan on IgG Using EndoS D233Q

Glycan-modified trastuzumab was prepared using an *in vitro* enzymatic reaction with endoS D233Q. Trastuzumab with GlcNAc (3 mg/mL in 50 mM citrate buffer pH 6.5) was incubated with endoS D233Q in the presence of glycan oxazoline, and a TSKgel FcR-IIIA-NPR column (TOSOH) was used for monitoring the reaction. TSKgel FcR-IIIA-5PW (7.8 mm I.D. × 7.5 cm) was used for the purification of the product. The mobile phases were buffer A (50 mM citrate at pH 6.5) and buffer B (50 mM citrate at pH 4.5). Separations were carried out at 25 °C and a flow rate of 1 mL/min.

### MS Measurement of IgGs with Homogeneous Glycans

The LC-MS/MS system was composed of a Waters Acquity UPLC H-Class Bio System with an MS/MS detector. Waters Vion IMS qTOF instrument was operated in positive ion/sensitivity mode at an *m*/*z* range of 400–4,000. The capillary voltage was set at 3 kV, and the cone voltage was set at 150 V with a source temperature of 120 °C and deconvolution temperature of 300 °C. Instrument control, data processing, and deconvolution were performed using Waters UNIFI v.1.9.4.053 with an advanced maximum entropy (MaxEnt)-based procedure. The samples were analyzed on a Waters MassPREP Micro Desalting Column (1000 Å, 20 μm, 2.1 × 5 mm) at 80 °C with a gradient of 0.1% formic acid in water and acetonitrile.

### ADCC Reporter Assay

According to previous studies,^9,46^ ADCC was performed using a Promega reporter bioassay kit (Cat # G7010). SK-BR3 (American Type Culture Collection [ATCC], Manassas, VA, USA) cells were maintained in McCoy’s 5A (modified) medium (ATCC) supplemented with 10% sterile filtered fetal bovine serum (ATCC). NFAT-engineered Jurkat cells expressing the FcγIIIa receptor were used as the effector cells.

When ready for the ADCC assay, target cells were harvested and inoculated in 384-well assay microplates. The following assay was performed according to the manufacturer’s protocol. Briefly, the glyco-engineered trastuzumab (10 μL, 400 ng/mL) was added and incubated for 1 h. Then, 5 × 10^4^ cells/well of the NFAT-engineered Jurkat cells expressing FcγRIIIa was added, and the cells were incubated for 5 h at 37 °C. At the time of effector cell addition, the ratio of effector cells to target cells was estimated to be 10:1. Receptor binding activated gene transcription through the NFAT pathway in the effector cell, leading to luciferase production. A luciferase substrate was then added, and luminescence was recorded using the multimode microplate reader EnSpire (PerkinElmer).

### Thermal Stability of IgG Using Differential Scanning Calorimetry

The thermal stability of each trastuzumab glycovariant was determined using DSC in a MicroCal PEAQ-DSC instrument (Malvern, UK). Samples were dialyzed against PBS prior to each scan. Protein samples at a concentration of 10 μM were subjected to a temperature ramp between 10 and 100 °C at a scan rate of 1.0 °C/min. The thermogram of the protein sample was normalized by subtracting the signal from the reference cell containing only the buffer. The melting temperature values were calculated by a standard fitting procedure using MicroCal PEAQ-DSC software and a non-two-state model.

## Supporting Information Available

Experimental details and characterization data (PDF)

## Abbreviations

ADCC: antibody-dependent cellular cytotoxicity
ΔH_cal_: calorimetric unfolding heat
DSC: differential scanning calorimetry
ENGase: endo-β-N-acetylglucosaminidase
Fc: crystallizable fragment
FcγR: Fcγ receptor
IgG: immunoglobulin G
MS: mass spectrometry, *T*_m_, thermal unfolding temperature
