# Supplementary material for: Systematic Preparation of a 66-IgG Library with Symmetric and Asymmetric Homogeneous Glycans and Their Functional Evaluation
Source: J Am Chem Soc. 2024 Aug 6;146(33):23426–36. doi: 10.1021/jacs.4c06558 (PMC11345770; doi:10.1021/jacs.4c06558)
Supplement: Supplementary file 1 — ja4c06558_si_001.pdf [file ja4c06558_si_001.pdf]

Supporting Information

Systematic Preparation of a 66-IgG Library with Symmetric and Asymmetric Homogeneous Glycans and Their Functional Evaluation

Shino Manabe,* Shogo Iwamoto, Satoru Nagatoishi, Asako Hoshinoo, Ai Mitani, Wataru Sumiyoshi, Takashi Kinoshita, Yoshiki Yamaguchi, and Kouhei Tsumoto

Contents

1. Table of glycan structure (Table S1) ········· S3
2. Table of sample number (Table S2) ········· S5
3. Figure of limitation of conventional protein glycan analysis (Fig. S1) ········· S6
4. ADCC assay of trastuzumab with homogeneous glycan with average, STDEV, and CV values. (Fig. S2) ········· S7
5. Expansion of Fig. 2 in main text (Scatter plot of the relationship between retention time in FcγRIIIa column chromatography) (Fig. S3) ········· S10
6. FcγRIIIa affinity column chromatography HPLC analysis of trastuzumab with homogeneous glycan. (Fig. S4) ········· S11
7. Intact MS analysis of trastuzumab with homogeneous glycan. (Fig. S5) ········· S78
8. DSC measurement of trastuzumab with homogeneous glycan. (Fig. S6) ········· S144

Table S1.

Table of glycan structure.

	Symbolic structure	Chemical structure
SG-F	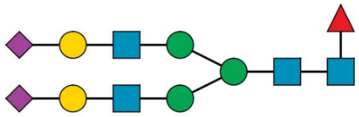	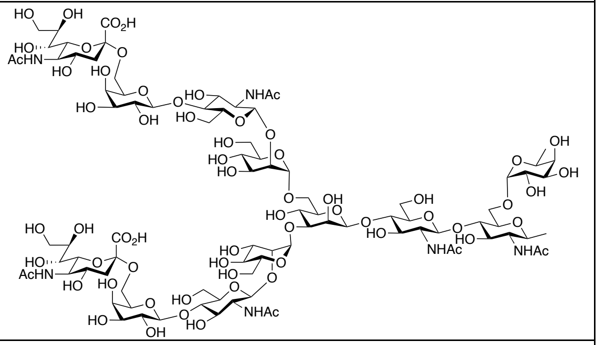
A1a-F	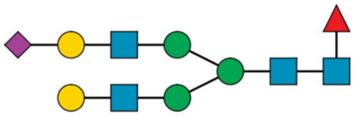	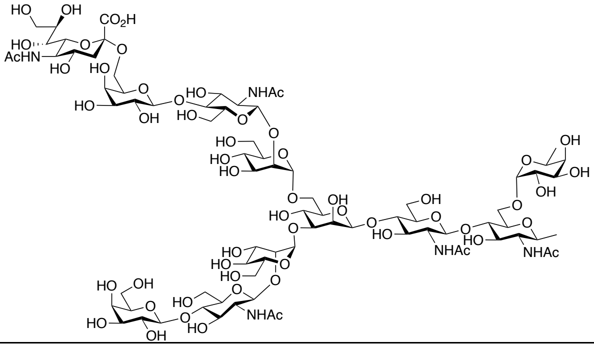
A1b-F	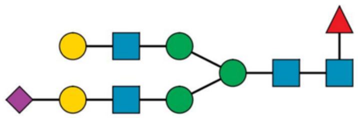	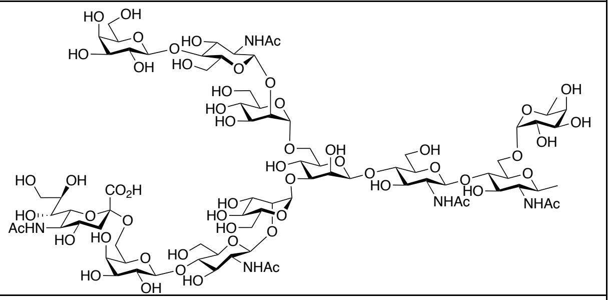
A1a-Gal-F	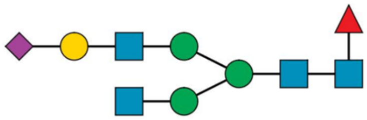	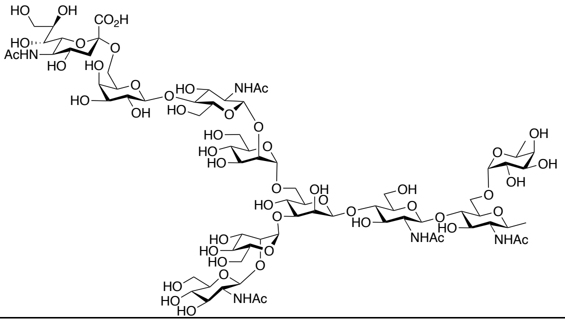
A1b-Gal-F	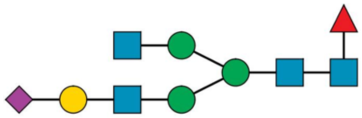	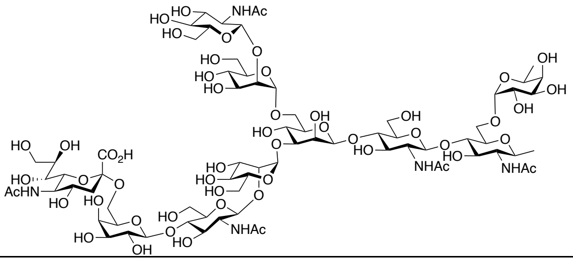

G2-F	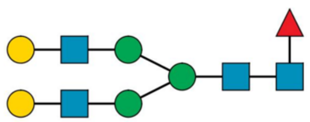	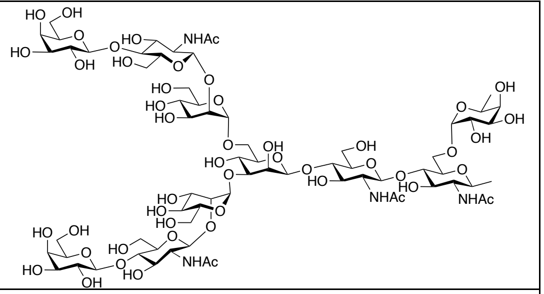
G1a-F	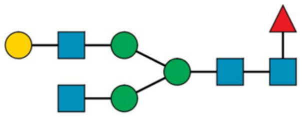	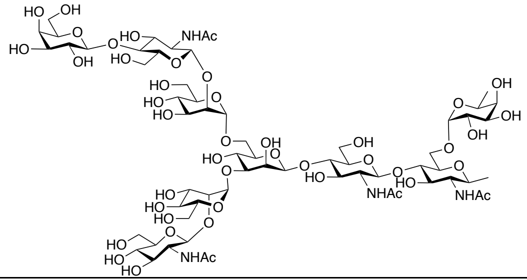
G1b-F	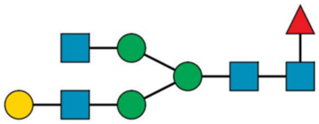	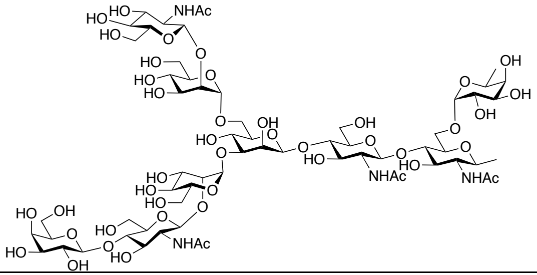
G0-F	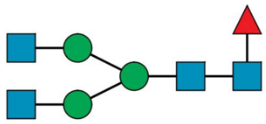	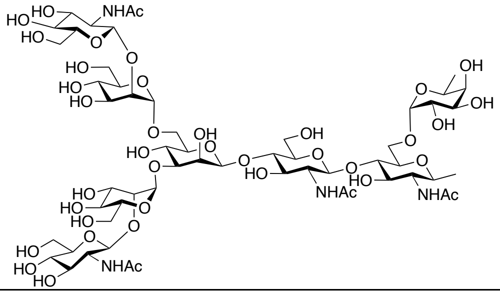
M3-F	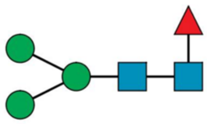	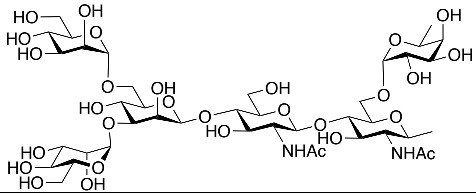
GlcNAc-F	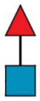	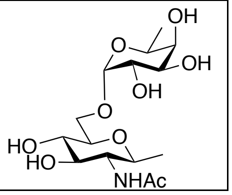

Table S2.
Table of sample number.

		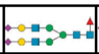	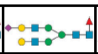	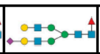	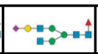	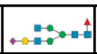	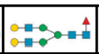	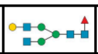	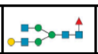	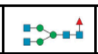	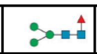	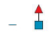
		SG-F	A1a-F	A1b-F	A1a-Gal-F	A1b-Gal-F	G2-F	G1a-F	G1b-F	G0-F	M3-F	GlcNAc-F
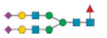	SG-F	1-1										
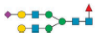	A1a-F	1-2	2-2									
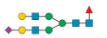	A1b-F	1-3	2-3	3-3								
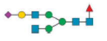	A1a-Gal-F	1-4	2-4	3-4	4-4							
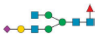	A1b-Gal-F	1-5	2-5	3-5	4-5	5-5						
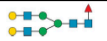	G2-F	1-6	2-6	3-6	4-6	5-6	6-6					
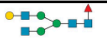	G1a-F	1-7	2-7	3-7	4-7	5-7	6-7	7-7				
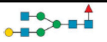	G1b-F	1-8	2-8	3-8	4-8	5-8	6-8	7-8	8-8			
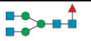	G0-F	1-9	2-9	3-9	4-9	5-9	6-9	7-9	8-9	9-9		
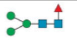	M3-F	1-10	2-10	3-10	4-10	5-10	6-10	7-10	8-10	9-10	10-10	
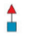	GlcNAc-F	1-11	2-11	3-11	4-11	5-11	6-11	7-11	8-11	9-11	10-11	11-11

Figure S1. Figure of limitation of conventional protein glycan analysis.

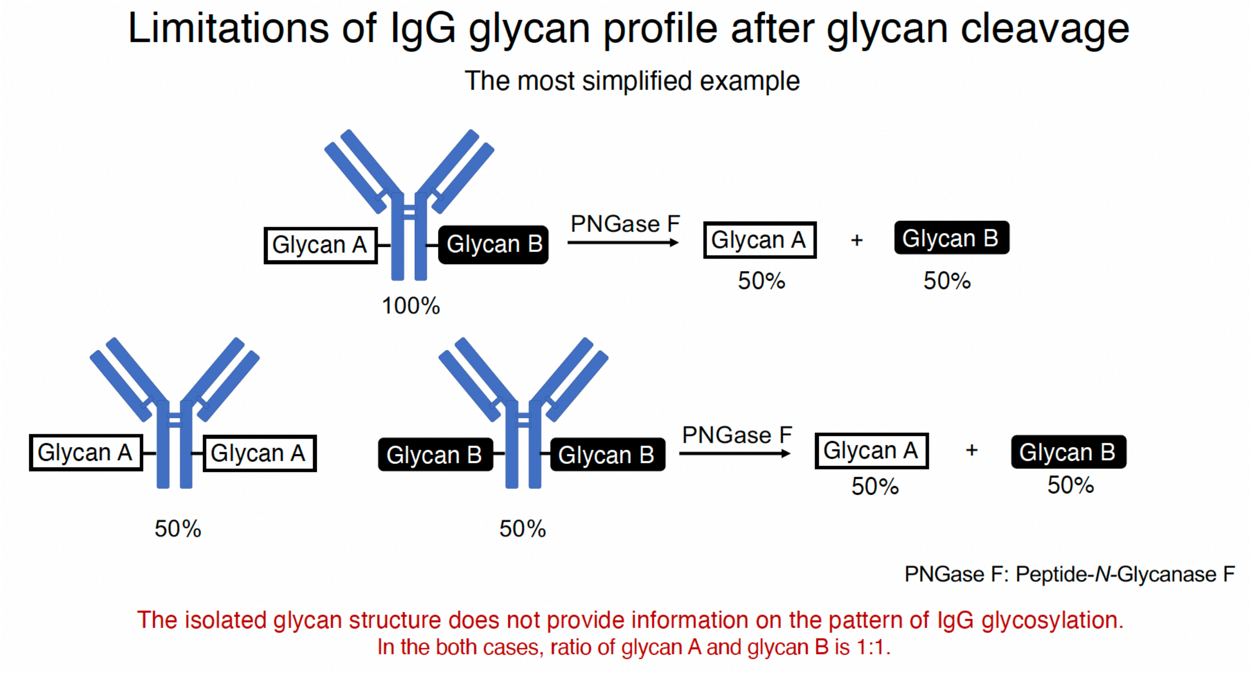

Figure S2.
ADCC assay of trastuzumab with homogeneous glycan with average, STDEV, and CV values.

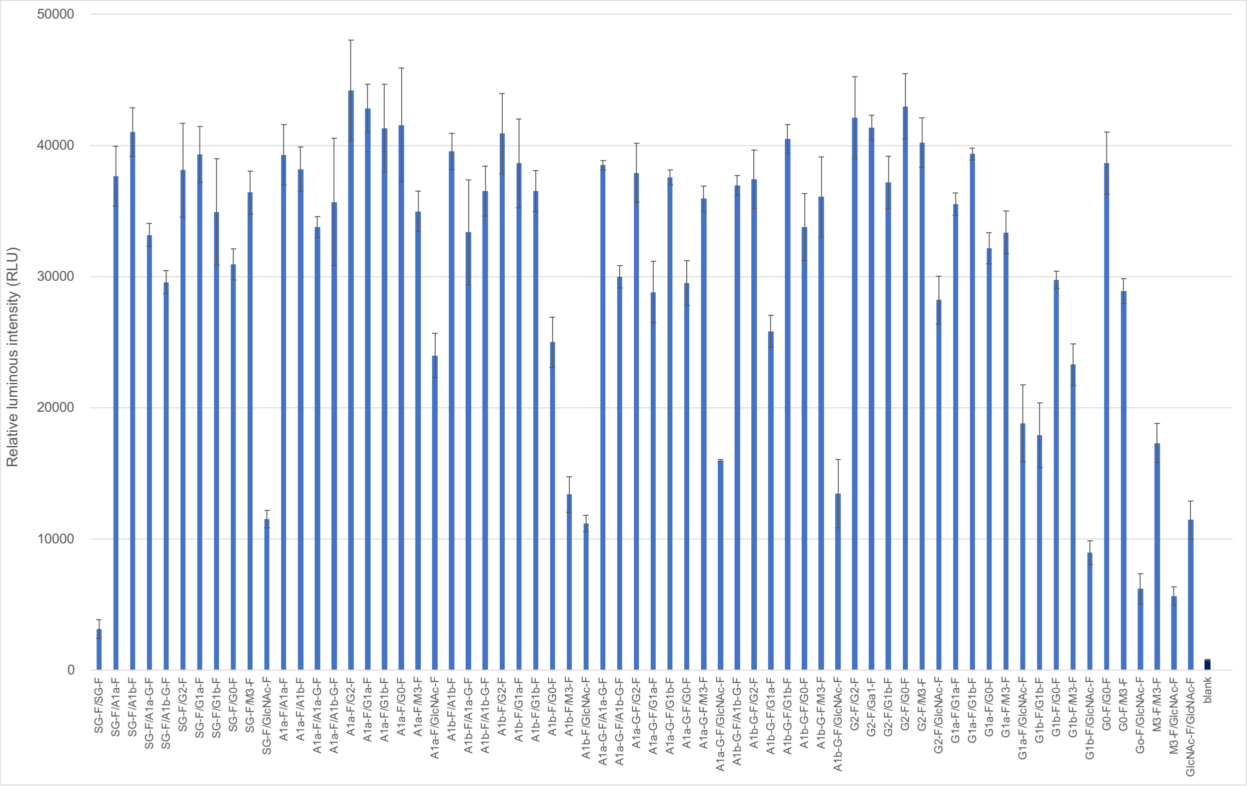

Sample No.	Glycan structure	AVERAGE	STDEV	CV
1-1	SG-F/SG-F	3133	717	23%
1-2	SG-F/A1a-F	37643	2278	6%
1-3	SG-F/A1b-F	41017	1836	4%
1-4	SG-F/A1a-G-F	33170	884	3%
1-5	SG-F/A1b-G-F	29577	881	3%
1-6	SG-F/G2-F	38107	3560	9%
1-7	SG-F/G1a-F	39320	2136	5%
1-8	SG-F/G1b-F	34920	4050	12%
1-9	SG-F/G0-F	30937	1193	4%
1-10	SG-F/M3-F	36403	1627	4%
1-11	SG-F/GlcNAc-F	11510	649	6%
2-2	A1a-F/A1a-F	39280	2286	6%
2-3	A1a-F/A1b-F	38193	1690	4%
2-4	A1a-F/A1a-G-F	33777	794	2%

2-5	A1a-F/A1b-G-F	35690	4838	14%
2-6	A1a-F/G2-F	44187	3837	9%
2-7	A1a-F/G1a-F	42807	1854	4%
2-8	A1a-F/G1b-F	41317	3328	8%
2-9	A1a-F/G0-F	41557	4327	10%
2-10	A1a-F/M3-F	34967	1530	4%
2-11	A1a-F/GlcNAc-F	23990	1701	7%
3-3	A1b-F/A1b-F	39547	1384	3%
3-4	A1b-F/A1a-G-F	33373	4019	12%
3-5	A1b-F/A1b-G-F	36510	1902	5%
3-6	A1b-F/G2-F	40923	3055	7%
3-7	A1b-F/G1a-F	38637	3382	9%
3-8	A1b-F/G1b-F	36523	1571	4%
3-9	A1b-F/G0-F	25007	1921	8%
3-10	A1b-F/M3-F	13400	1348	10%
3-11	A1b-F/GlcNAc-F	11173	613	5%
4-4	A1a-G-F/A1a-G-F	38490	359	1%
4-5	A1a-G-F/A1b-G-F	29983	836	3%
4-6	A1a-G-F/G2-F	37900	2246	6%
4-7	A1a-G-F/G1a-F	28817	2352	8%
4-8	A1a-G-F/G1b-F	37573	573	2%
4-9	A1a-G-F/G0-F	29507	1703	6%
4-10	A1a-G-F/M3-F	35940	964	3%
4-11	A1a-G-F/GlcNAc-F	15983	68	0%
5-5	A1b-G-F/A1b-G-F	36930	764	2%
5-6	A1b-G-F/G2-F	37417	2224	6%
5-7	A1b-G-F/G1a-F	25830	1211	5%
5-8	A1b-G-F/G1b-F	40503	1093	3%
5-9	A1b-G-F/G0-F	33773	2574	8%
5-10	A1b-G-F/M3-F	36080	3042	8%
5-11	A1b-G-F/GlcNAc-F	13450	2592	19%
6-6	G2-F/G2-F	42130	3126	7%
6-7	G2-F/Ga1-F	41340	935	2%
6-8	G2-F/G1b-F	37167	1992	5%
6-9	G2-F/G0-F	42967	2489	6%

6-10	G2-F/M3-F	40213	1911	5%
6-11	G2-F/GlcNAc-F	28227	1824	6%
7-7	G1a-F/G1a-F	35523	869	2%
7-8	G1a-F/G1b-F	39343	467	1%
7-9	G1a-F/G0-F	32157	1198	4%
7-10	G1a-F/M3-F	33367	1640	5%
7-11	G1a-F/GlcNAc-F	18807	2924	16%
8-8	G1b-F/G1b-F	17920	2466	14%
8-9	G1b-F/G0-F	29753	659	2%
8-10	G1b-F/M3-F	23283	1583	7%
8-11	G1b-F/GlcNAc-F	8930	900	10%
9-9	G0-F/G0-F	38633	2370	6%
9-10	G0-F/M3-F	28880	954	3%
9-11	G0-F/GlcNAc-F	6193	1156	19%
10-10	M3-F/M3-F	17303	1482	9%
10-11	M3-F/GlcNAc-F	5647	719	13%
11-11	GlcNAc-F/GlcNAc-F	11450	1438	13%
	blank	750	62	8%

Figure S3.

Expansion of Fig 2 in main text (Scatter plot of the relationship between retention time in FcγRIIIa column chromatography).

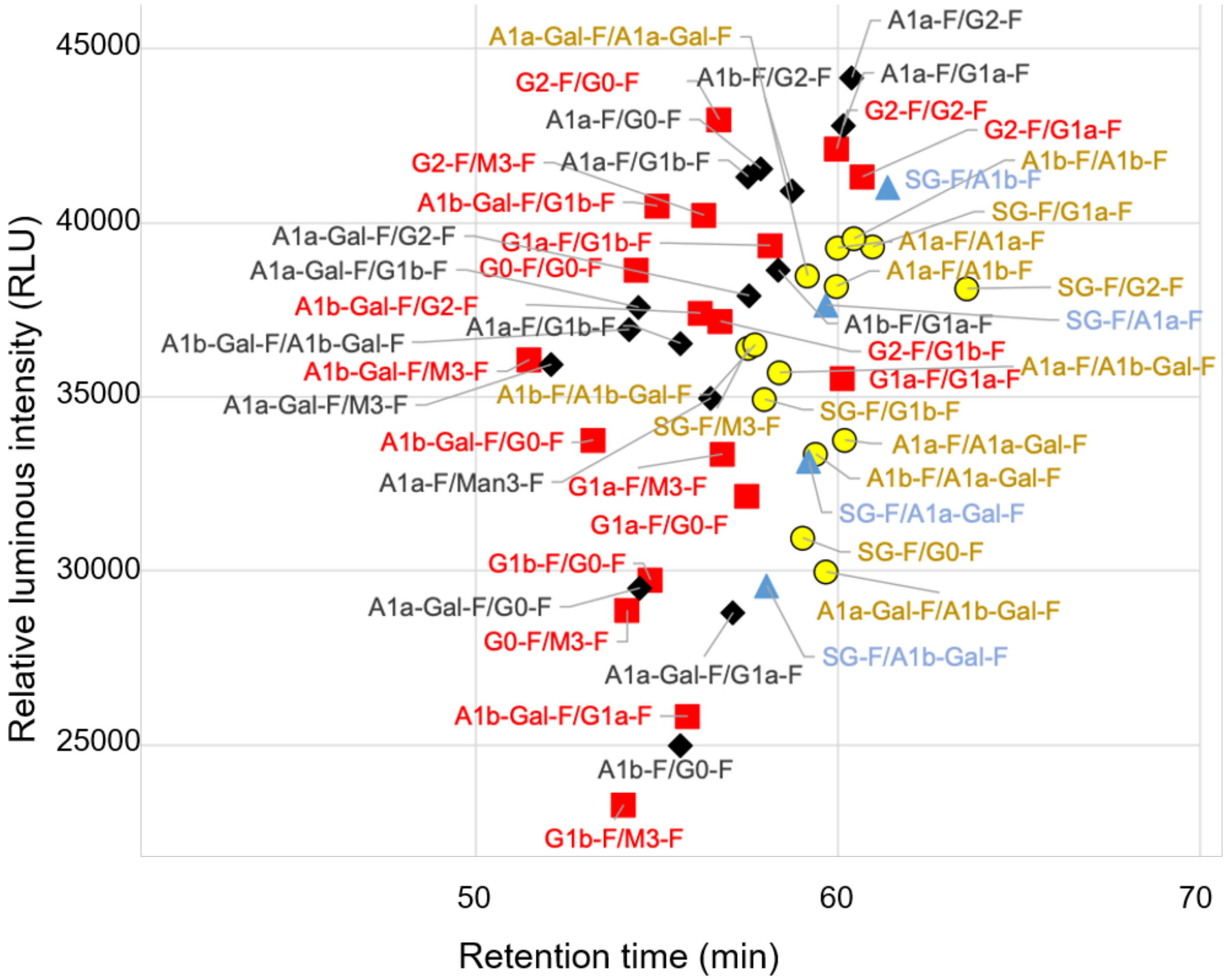

Figure S4.
FcγRIIIa affinity column chromatography analysis of trastuzumab with homogeneous glycan.

1-1 [SG-F/SG-F]

Analysis Report

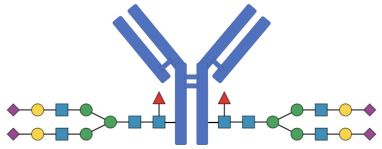

<Sample Information>

Sample Name : 230313_01_SG_SG
Sample ID : 230313_01_SG_SG
Data Filename : 230313_01_SG_SG.lcd
Method Filename : 2303_FcRIII_bunseki_AS_100min.lcm
Batch Filename :
Vial # : 1
Sample Type : Unknown
Injection Volume : 20 uL
Date Acquired : 2023/03/15 14:11:41
Acquired by : System Administrator
Date Processed : 2023/03/28 9:27:22
Processed by : System Administrator

<Chromatogram>

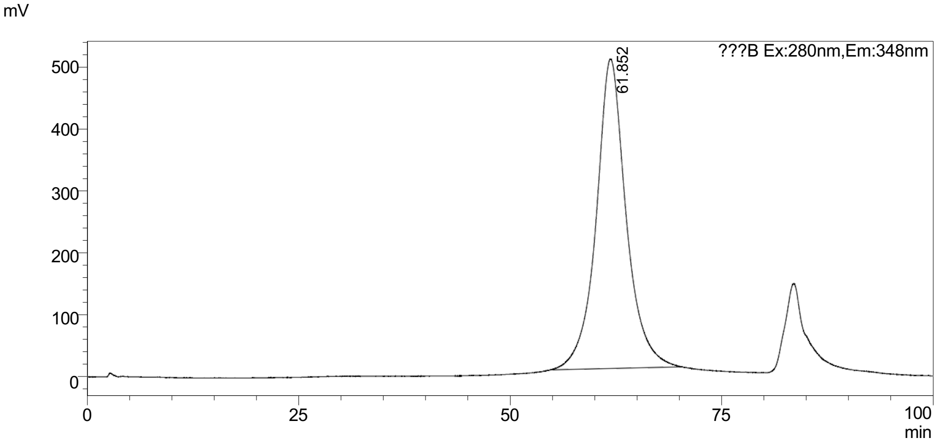

<Peak Table>

???B Ex:280nm,Em:348nm

Peak#	Ret. Time	Area	Height	Conc.	Unit	Mark	Name
1	61.852	123232395	500046	100.000		M	

1-2 [SG-F/A1a-F]

Analysis Report

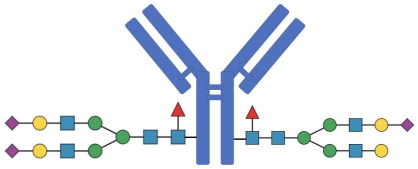

<Sample Information>

Sample Name	: 230313_02_SG_A1a		
Sample ID	: 230313_02_SG_A1a		
Data Filename	: 230313_02_SG_A1a.lcd		
Method Filename	: 2303_FcRIII_bunseki_AS_100min.lcm		
Batch Filename	:		
Vial #	: 2	Sample Type	: Unknown
Injection Volume	: 20 uL		
Date Acquired	: 2023/03/15 15:56:23	Acquired by	: System Administrator
Date Processed	: 2023/03/28 9:29:26	Processed by	: System Administrator

<Chromatogram>

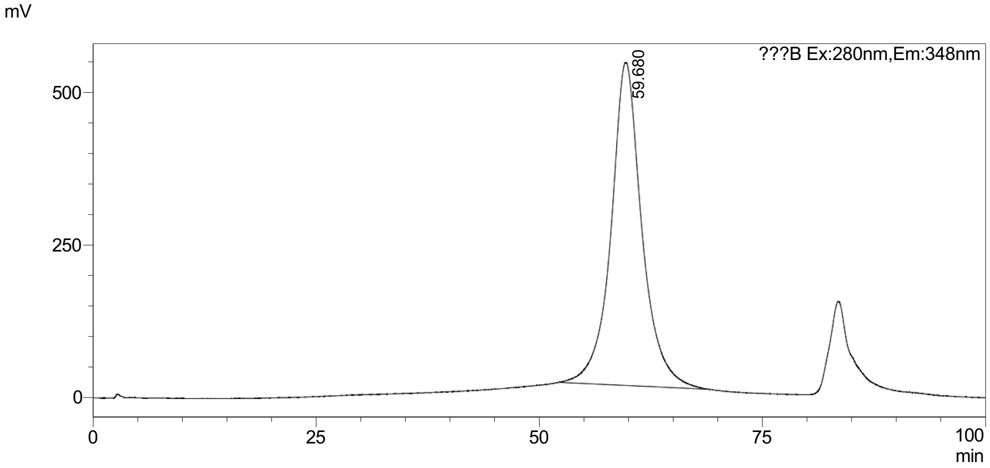

<Peak Table>

???B Ex:280nm,Em:348nm

Peak#	Ret. Time	Area	Height	Conc.	Unit	Mark	Name
1	59.680	127399402	528661	100.000		M	

1-3 [SG-F/A1b-F]

Analysis Report

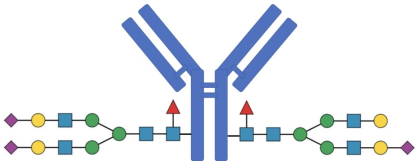

<Sample Information>

Sample Name	: 230313_03_SG_A1b		
Sample ID	: 230313_03_SG_A1b		
Data Filename	: 230313_03_SG_A1b.lcd		
Method Filename	: 2303_FcRIII_bunseki_AS_100min.lcm		
Batch Filename	:		
Vial #	: 3	Sample Type	: Unknown
Injection Volume	: 20 uL		
Date Acquired	: 2023/03/16 10:56:52	Acquired by	: System Administrator
Date Processed	: 2023/03/28 9:30:37	Processed by	: System Administrator

<Chromatogram>

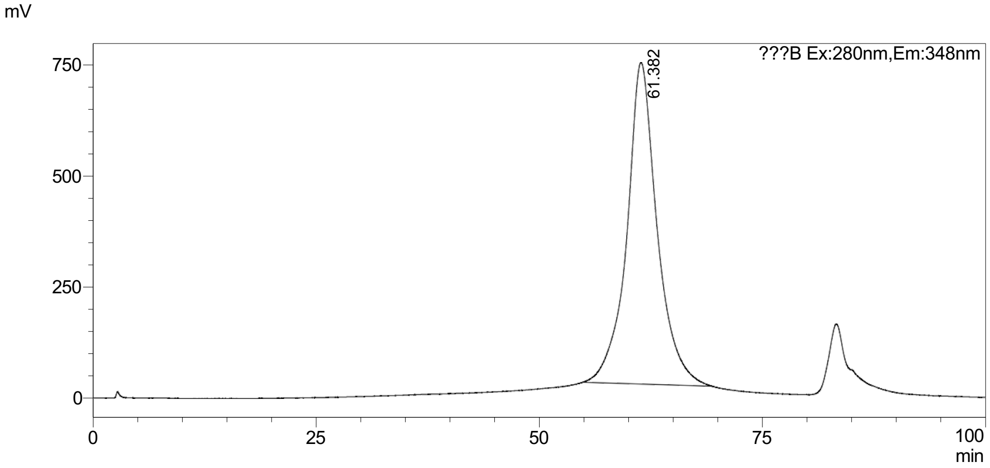

<Peak Table>

???B Ex:280nm,Em:348nm

Peak#	Ret. Time	Area	Height	Conc.	Unit	Mark	Name
1	61.382	170757741	724543	100.000		M	

1-4 [SG-F/A1a-Gal-F]

Analysis Report

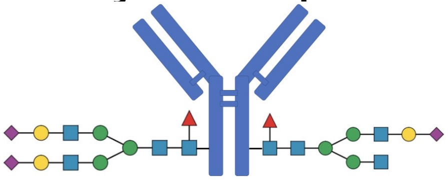

<Sample Information>

Sample Name	: 230313_04_SG_A1a-G		
Sample ID	: 230313_04_SG_A1a-G		
Data Filename	: 230313_04_SG_A1a-G.lcd		
Method Filename	: 2303_FcRIII_bunseki_AS_100min.lcm		
Batch Filename	:		
Vial #	: 4	Sample Type	: Unknown
Injection Volume	: 20 uL		
Date Acquired	: 2023/03/16 13:20:56	Acquired by	: System Administrator
Date Processed	: 2023/03/28 9:31:37	Processed by	: System Administrator

<Chromatogram>

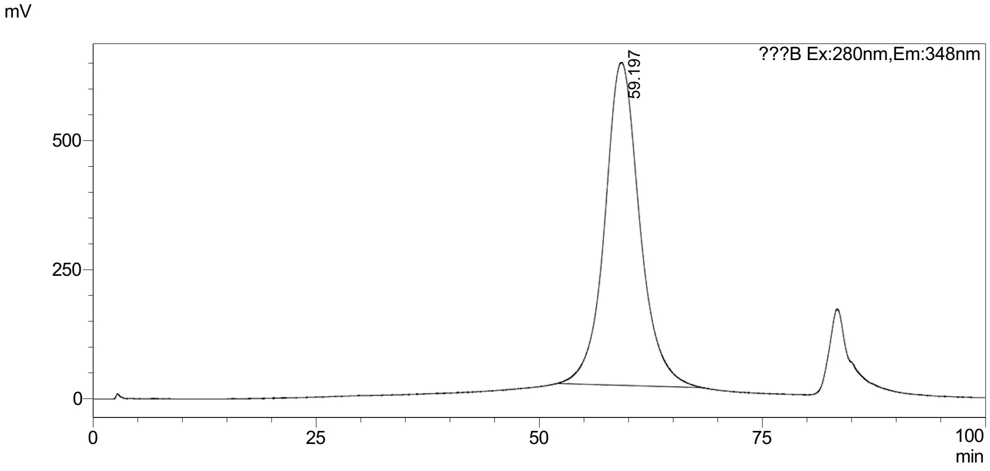

<Peak Table>

???B Ex:280nm,Em:348nm

Peak#	Ret. Time	Area	Height	Conc.	Unit	Mark	Name
1	59.197	170439301	624678	100.000		M	

1-5 [SG-F/A1b-Gal-F]

Analysis Report

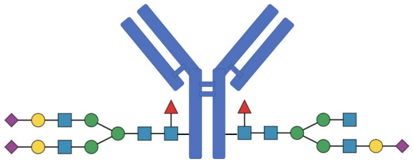

<Sample Information>

Sample Name	: 230313_05_SG_A1b-G		
Sample ID	: 230313_05_SG_A1b-G		
Data Filename	: 230313_05_SG_A1b-G.lcd		
Method Filename	: 2303_FcRIII_bunseki_AS_100min.lcm		
Batch Filename	:		
Vial #	: 5	Sample Type	: Unknown
Injection Volume	: 20 uL		
Date Acquired	: 2023/03/16 15:10:05	Acquired by	: System Administrator
Date Processed	: 2023/03/28 9:32:39	Processed by	: System Administrator

<Chromatogram>

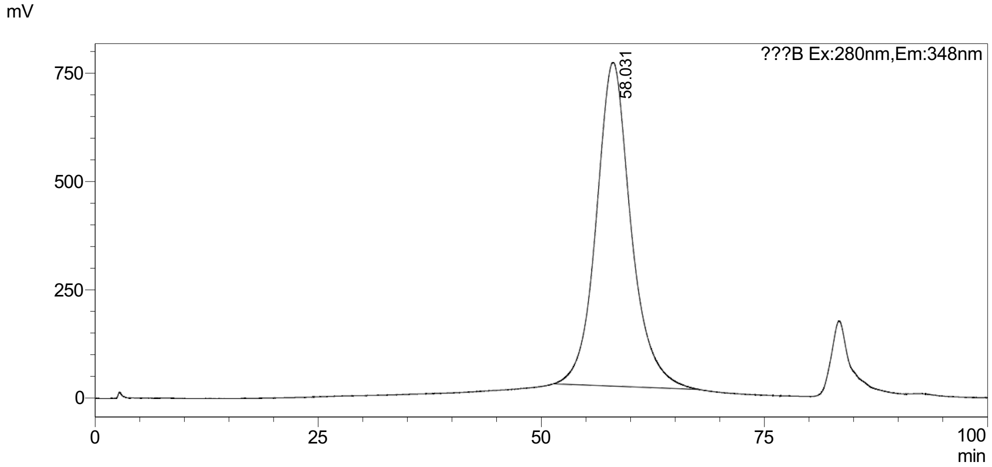

<Peak Table>

???B Ex:280nm,Em:348nm

Peak#	Ret. Time	Area	Height	Conc.	Unit	Mark	Name
1	58.031	202108883	746327	100.000		M	

1-6 [SG-F/G2-F]

Analysis Report

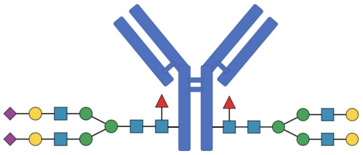

<Sample Information>

Sample Name	: 230313_06_SG_G2
Sample ID	: 230313_06_SG_G2
Data Filename	: 230313_06_SG_G2.lcd
Method Filename	: 2303_FcRIII_bunseki_AS_100min.lcm
Batch Filename	:
Vial #	: 6
Injection Volume	: 20 uL
Date Acquired	: 2023/03/16 17:02:50
Date Processed	: 2023/03/28 9:56:03

Sample Type	: Unknown
Acquired by	: System Administrator
Processed by	: System Administrator

<Chromatogram>

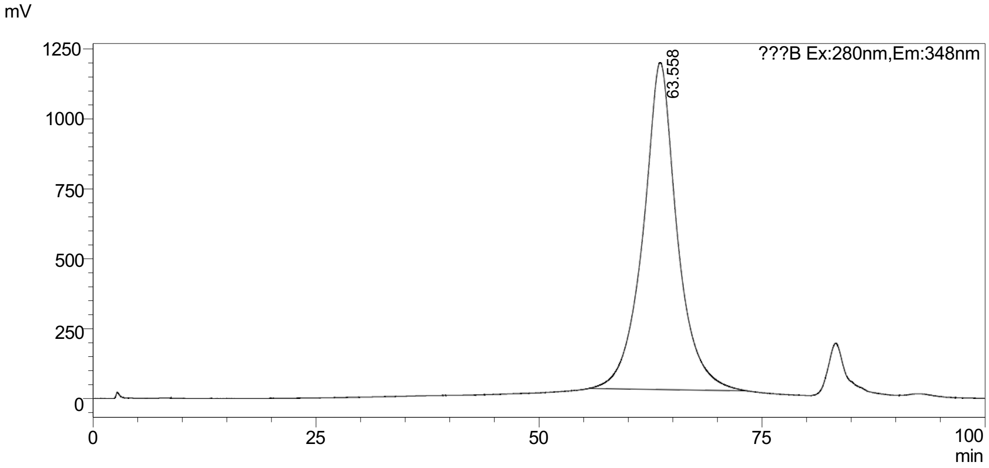

<Peak Table>

???B Ex:280nm,Em:348nm

Peak#	Ret. Time	Area	Height	Conc.	Unit	Mark	Name
1	63.558	310988551	1170085	100.000		M	

1-7 [SG-F/G1a-F]

Analysis Report

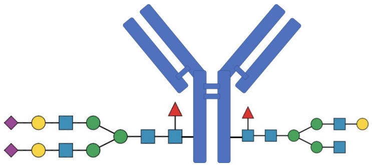

<Sample Information>

Sample Name	: 230313_07_SG_G1a		
Sample ID	: 230313_07_SG_G1a		
Data Filename	: 230313_07_SG_G1a.lcd		
Method Filename	: 2303_FcRIII_bunseki_AS_100min.lcm		
Batch Filename	:		
Vial #	: 7	Sample Type	: Unknown
Injection Volume	: 20 uL		
Date Acquired	: 2023/03/15 17:41:21	Acquired by	: System Administrator
Date Processed	: 2023/03/28 9:56:59	Processed by	: System Administrator

<Chromatogram>

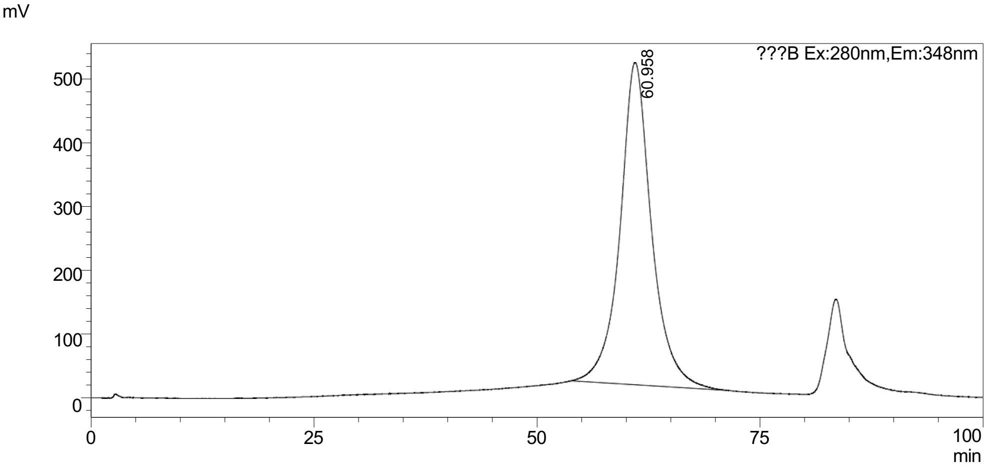

<Peak Table>

???B Ex:280nm,Em:348nm

Peak#	Ret. Time	Area	Height	Conc.	Unit	Mark	Name
1	60.958	124556913	506022	100.000		M	

1-8 [SG-F/G1b-F]

Analysis Report

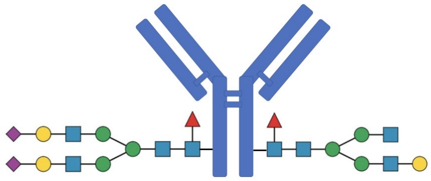

<Sample Information>

Sample Name	: 230313_08_SG_G1b		
Sample ID	: 230313_08_SG_G1b		
Data Filename	: 230313_08_SG_G1b.lcd		
Method Filename	: 2303_FcRIII_bunseki_AS_100min.lcm		
Batch Filename	:		
Vial #	: 8	Sample Type	: Unknown
Injection Volume	: 20 uL		
Date Acquired	: 2023/03/16 9:12:24	Acquired by	: System Administrator
Date Processed	: 2023/03/28 9:58:36	Processed by	: System Administrator

<Chromatogram>

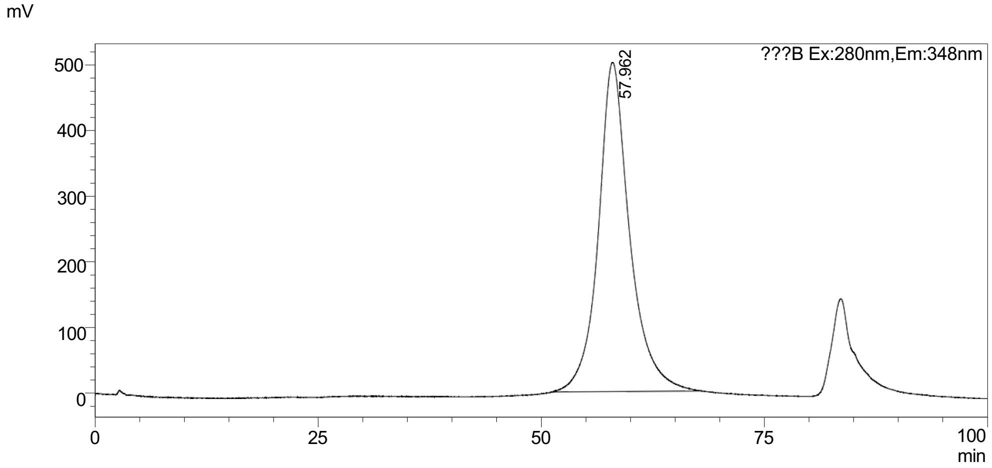

<Peak Table>

???B Ex:280nm,Em:348nm

Peak#	Ret. Time	Area	Height	Conc.	Unit	Mark	Name
1	57.962	122450086	501007	100.000		M	

1-9 [SG-F/G0-F]

Analysis Report

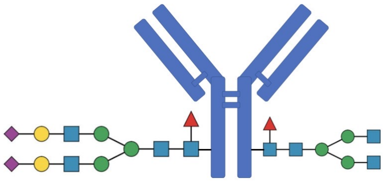

<Sample Information>

Sample Name	: 230313_09_SG_G0		
Sample ID	: 230313_09_SG_G0		
Data Filename	: 230313_09_SG_G0.lcd		
Method Filename	: 2303_FcRIII_bunseki_AS_100min.lcm		
Batch Filename	:		
Vial #	: 9	Sample Type	: Unknown
Injection Volume	: 20 uL		
Date Acquired	: 2023/03/16 18:48:27	Acquired by	: System Administrator
Date Processed	: 2023/03/28 9:59:48	Processed by	: System Administrator

<Chromatogram>

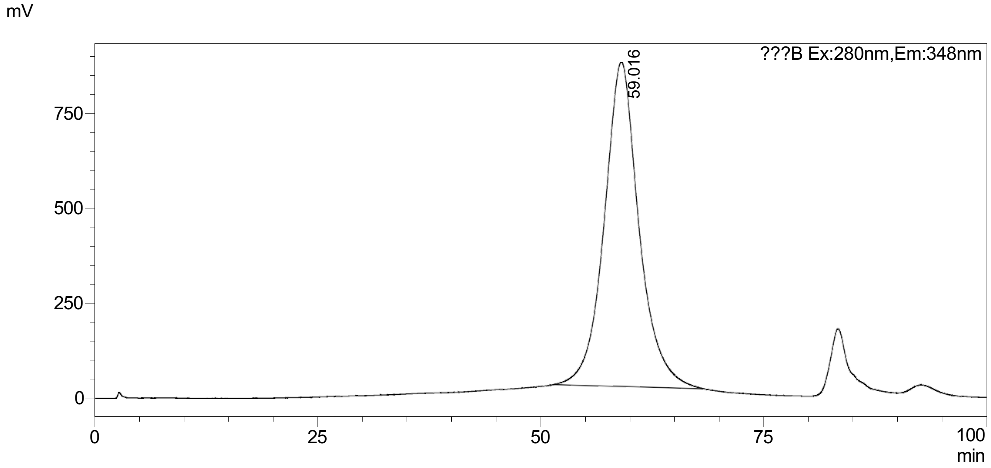

<Peak Table>

???B Ex:280nm,Em:348nm

Peak#	Ret. Time	Area	Height	Conc.	Unit	Mark	Name
1	59.016	231733308	853684	100.000		M	

1-10 [SG-F/M3-F]

Analysis Report

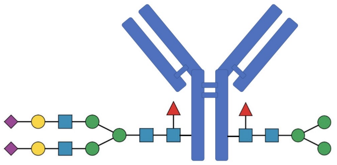

<Sample Information>

Sample Name	: 230313_10_SG_M3		
Sample ID	: 230313_10_SG_M3		
Data Filename	: 230313_10_SG_M3.lcd		
Method Filename	: 2303_FcRIII_bunseki_AS_100min.lcm		
Batch Filename	:		
Vial #	: 10	Sample Type	: Unknown
Injection Volume	: 20 uL		
Date Acquired	: 2023/03/16 20:32:21	Acquired by	: System Administrator
Date Processed	: 2023/03/28 10:00:47	Processed by	: System Administrator

<Chromatogram>

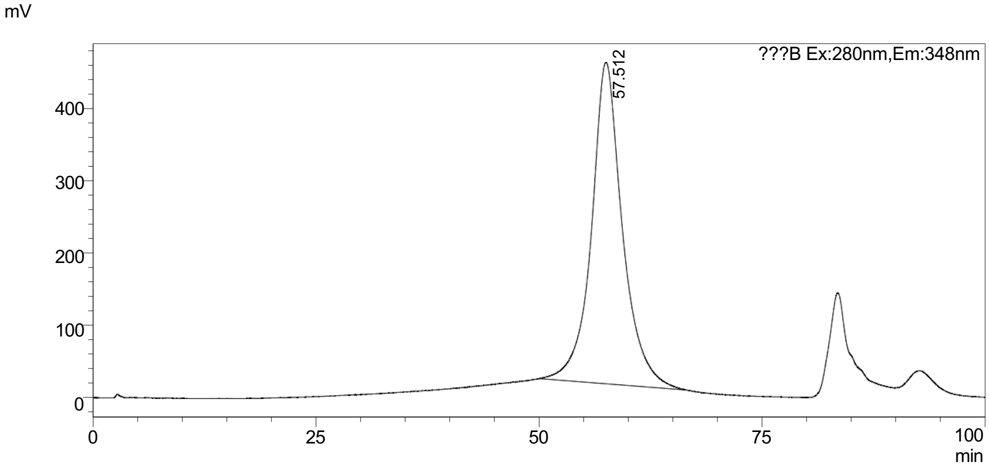

<Peak Table>

???B Ex:280nm,Em:348nm

Peak#	Ret. Time	Area	Height	Conc.	Unit	Mark	Name
1	57.512	107369803	444500	100.000		M	

1-11 [SG-F/GlcNAc-F]

Analysis Report

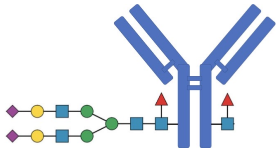

<Sample Information>

Sample Name	: 230313_11_SG_GlcNAc		
Sample ID	: 230313_11_SG_GlcNAc		
Data Filename	: 230313_11_SG_GlcNAc.lcd		
Method Filename	: 2303_FcRIII_bunseki_AS_100min.lcm		
Batch Filename	:		
Vial #	: 11	Sample Type	: Unknown
Injection Volume	: 20 uL		
Date Acquired	: 2023/03/27 10:50:06	Acquired by	: System Administrator
Date Processed	: 2023/03/28 10:02:23	Processed by	: System Administrator

<Chromatogram>

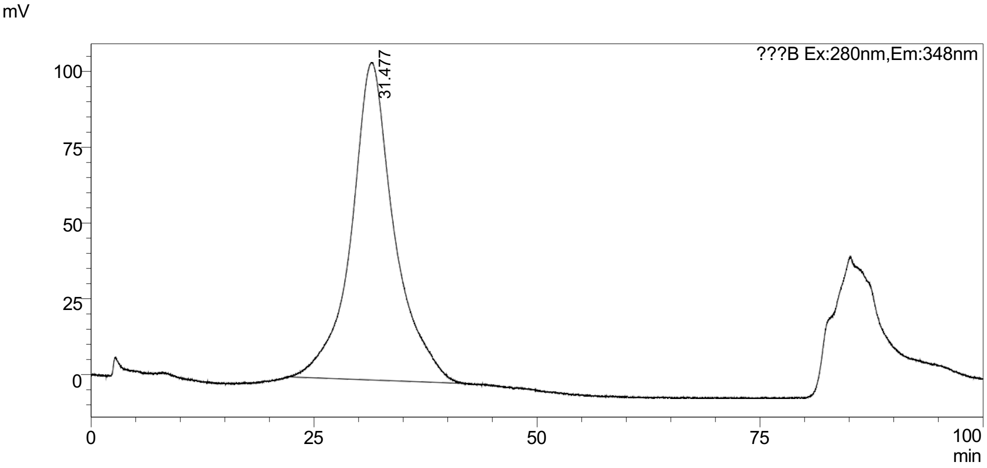

<Peak Table>

???B Ex:280nm,Em:348nm

Peak#	Ret. Time	Area	Height	Conc.	Unit	Mark	Name
1	31.477	35045211	104656	100.000		M	

2-2 [A1a-F/A1a-F]

Analysis Report

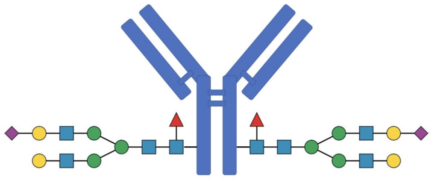

<Sample Information>

Sample Name	: 230313_12_A1a_A1a		
Sample ID	: 230313_12_A1a_A1a		
Data Filename	: 230313_12_A1a_A1a001.lcd		
Method Filename	: 2303_FcRIII_bunseki_AS_100min.lcm		
Batch Filename	:		
Vial #	: 12	Sample Type	: Unknown
Injection Volume	: 20 uL		
Date Acquired	: 2023/03/17 12:59:05	Acquired by	: System Administrator
Date Processed	: 2023/03/28 10:04:04	Processed by	: System Administrator

<Chromatogram>

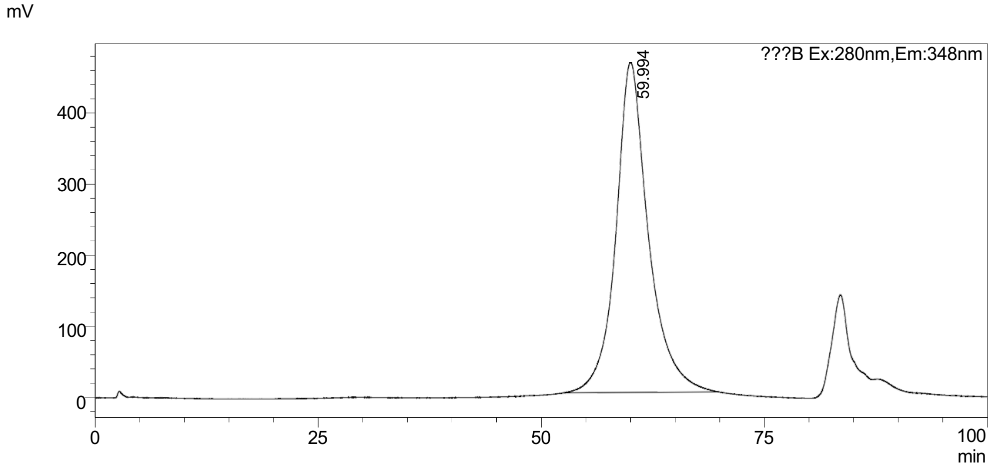

<Peak Table>

???B Ex:280nm,Em:348nm

Peak#	Ret. Time	Area	Height	Conc.	Unit	Mark	Name
1	59.994	117743404	463773	100.000		M	

2-3 [A1a-F/A1b-F]

Analysis Report

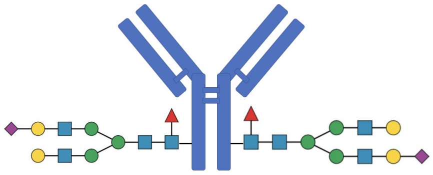

<Sample Information>

Sample Name	: 230313_13_A1a_A1b		
Sample ID	: 230313_13_A1a_A1b		
Data Filename	: 230313_13_A1a_A1b.lcd		
Method Filename	: 2303_FcRIII_bunseki_AS_100min.lcm		
Batch Filename	:		
Vial #	: 13	Sample Type	: Unknown
Injection Volume	: 20 uL		
Date Acquired	: 2023/03/17 14:43:41	Acquired by	: System Administrator
Date Processed	: 2023/03/28 10:05:10	Processed by	: System Administrator

<Chromatogram>

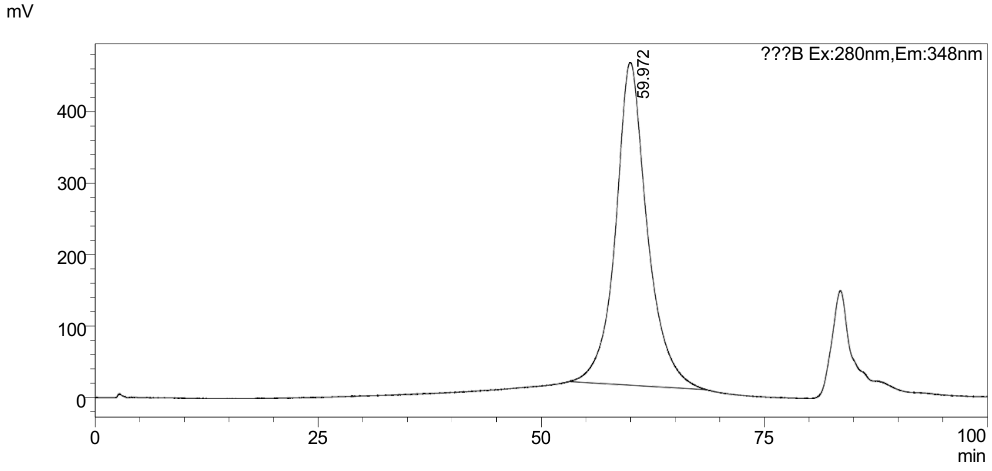

<Peak Table>

???B Ex:280nm,Em:348nm

Peak#	Ret. Time	Area	Height	Conc.	Unit	Mark	Name
1	59.972	111700793	451652	100.000		M	

2-4 [A1a-F/A1a-Gal-F]

Analysis Report

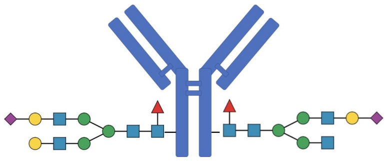

<Sample Information>

Sample Name : 230313_14_A1a_A1a-G
Sample ID : 230313_14_A1a_A1a-G
Data Filename : 230313_14_A1a_A1a-G.lcd
Method Filename : 2303_FcRIII_bunseki_AS_100min.lcm
Batch Filename :
Vial # : 14
Sample Type : Unknown
Injection Volume : 20 uL
Date Acquired : 2023/03/17 16:31:14
Acquired by : System Administrator
Date Processed : 2023/03/28 10:06:07
Processed by : System Administrator

<Chromatogram>

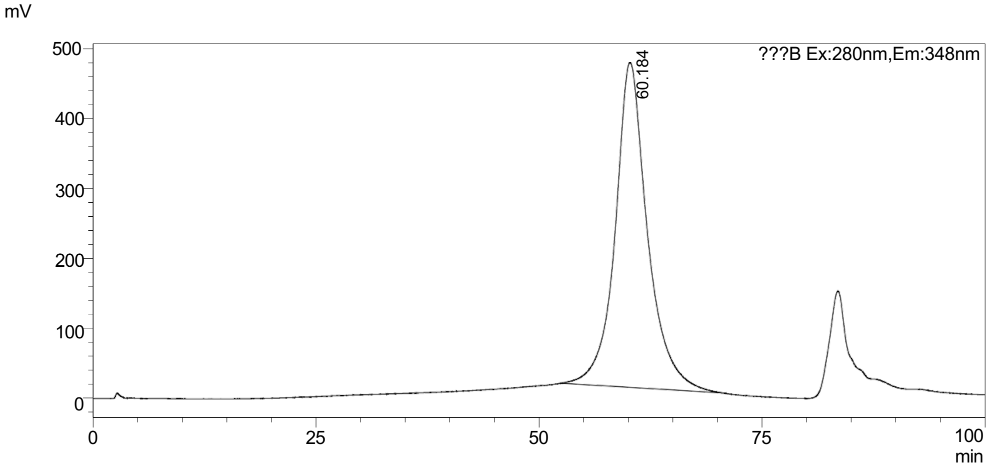

<Peak Table>

???B Ex:280nm,Em:348nm

Peak#	Ret. Time	Area	Height	Conc.	Unit	Mark	Name
1	60.184	117255210	464690	100.000		M	

2-5 [A1a-F/A1b-Gal-F]

Analysis Report

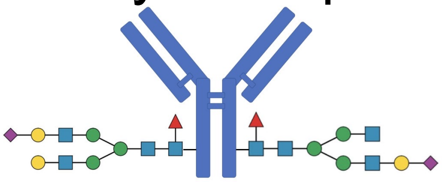

<Sample Information>

Sample Name	: 230313_15_A1a_A1b-G		
Sample ID	: 230313_15_A1a_A1b-G		
Data Filename	: 230313_15_A1a_A1b-G.lcd		
Method Filename	: 2303_FcRIII_bunseki_AS_100min.lcm		
Batch Filename	:		
Vial #	: 15	Sample Type	: Unknown
Injection Volume	: 20 uL		
Date Acquired	: 2023/03/17 18:20:16	Acquired by	: System Administrator
Date Processed	: 2023/03/28 10:07:05	Processed by	: System Administrator

<Chromatogram>

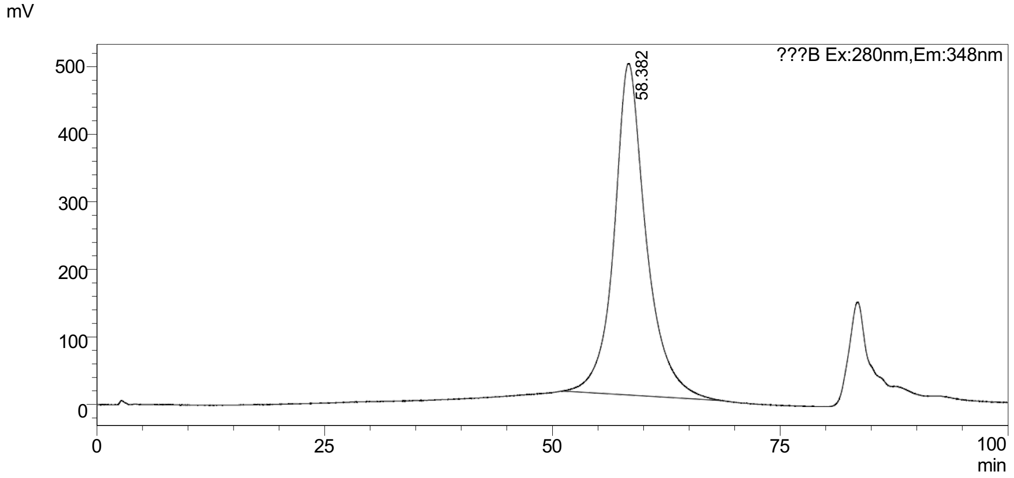

<Peak Table>

???B Ex:280nm,Em:348nm

Peak#	Ret. Time	Area	Height	Conc.	Unit	Mark	Name
1	58.382	120409571	490619	100.000		M	

2-6 [A1a-F/G2-F]

Analysis Report

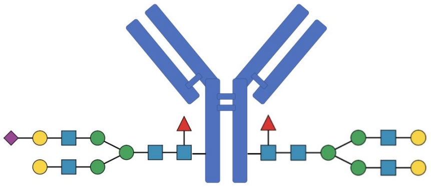

<Sample Information>

Sample Name	: 230313_16_A1a_G2		
Sample ID	: 230313_16_A1a_G2		
Data Filename	: 230313_16_A1a_G2.lcd		
Method Filename	: 2303_FcRIII_bunseki_AS_100min.lcm		
Batch Filename	:		
Vial #	: 16	Sample Type	: Unknown
Injection Volume	: 20 uL		
Date Acquired	: 2023/03/17 20:08:53	Acquired by	: System Administrator
Date Processed	: 2023/03/28 10:09:36	Processed by	: System Administrator

<Chromatogram>

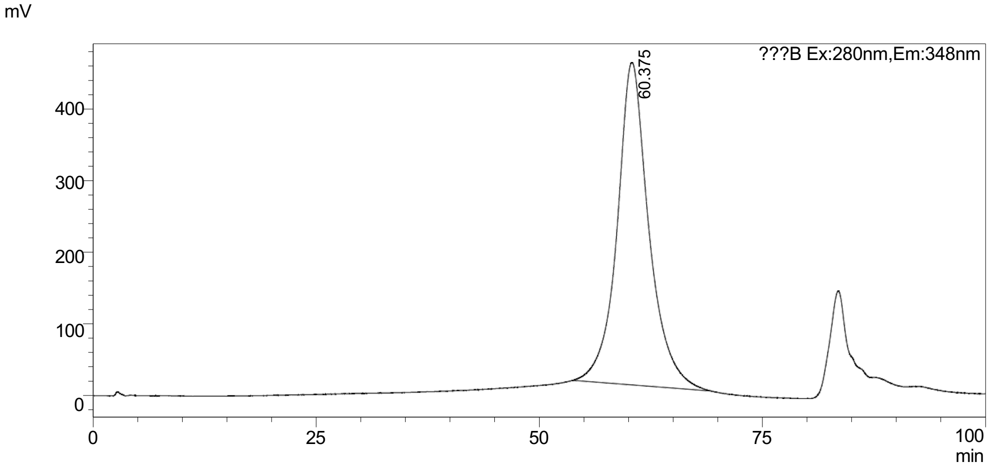

<Peak Table>

???B Ex:280nm,Em:348nm

Peak#	Ret. Time	Area	Height	Conc.	Unit	Mark	Name
1	60.375	111332077	449794	100.000		M	

2-7 [A1a-F/G1a-F]

Analysis Report

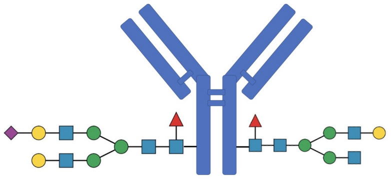

<Sample Information>

Sample Name	: 230313_17_A1a_G1a		
Sample ID	: 230313_17_A1a_G1a		
Data Filename	: 230313_17_A1a_G1a.lcd		
Method Filename	: 2303_FcRIII_bunseki_AS_100min.lcm		
Batch Filename	:		
Vial #	: 17	Sample Type	: Unknown
Injection Volume	: 20 uL		
Date Acquired	: 2023/03/18 9:20:50	Acquired by	: System Administrator
Date Processed	: 2023/03/28 10:10:35	Processed by	: System Administrator

<Chromatogram>

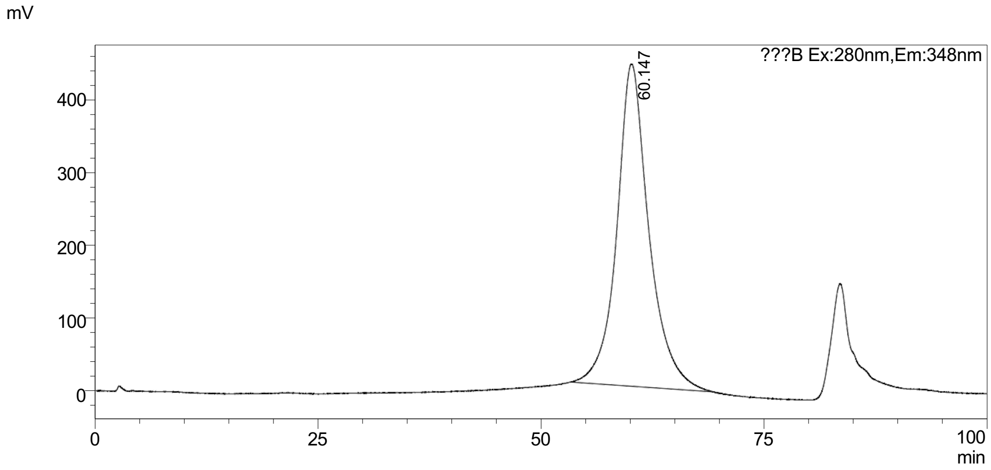

<Peak Table>

???B Ex:280nm,Em:348nm

Peak#	Ret. Time	Area	Height	Conc.	Unit	Mark	Name
1	60.147	111176665	443777	100.000		M	

2-8 [A1a-F/G1b-F]

Analysis Report

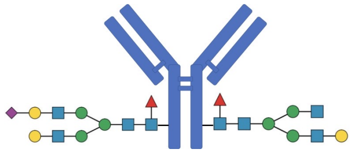

<Sample Information>

Sample Name	: 230313_18_A1a_G1b		
Sample ID	: 230313_18_A1a_G1b		
Data Filename	: 230313_18_A1a_G1b.lcd		
Method Filename	: 2303_FcRIII_bunseki_AS_100min.lcm		
Batch Filename	:		
Vial #	: 18	Sample Type	: Unknown
Injection Volume	: 20 uL		
Date Acquired	: 2023/03/18 11:04:50	Acquired by	: System Administrator
Date Processed	: 2023/03/28 10:11:36	Processed by	: System Administrator

<Chromatogram>

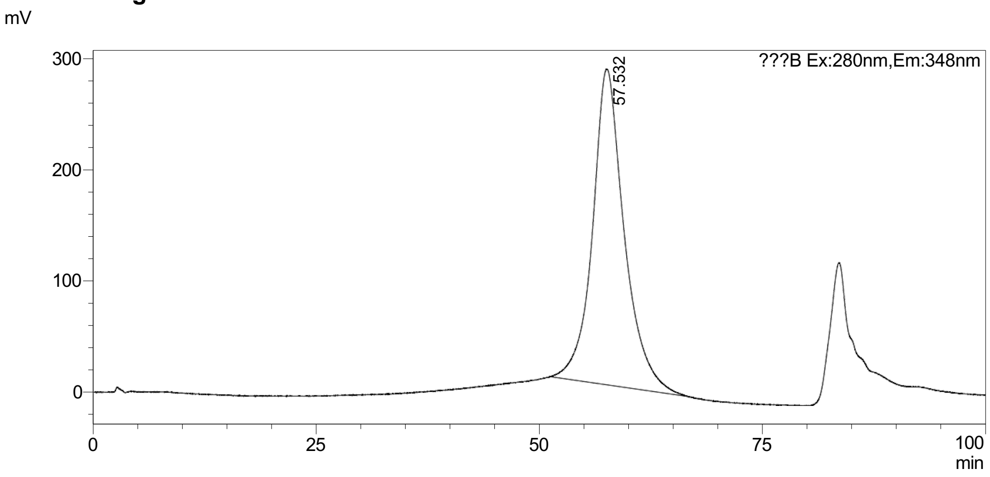

<Peak Table>

???B Ex:280nm,Em:348nm

Peak#	Ret. Time	Area	Height	Conc.	Unit	Mark	Name
1	57.532	70168625	283875	100.000		M	

2-9 [A1a-F/G0-F]

Analysis Report

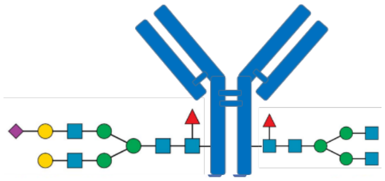

<Sample Information>

Sample Name	: 230313_19_A1a_G0		
Sample ID	: 230313_19_A1a_G0		
Data Filename	: 230313_19_A1a_G0.lcd		
Method Filename	: 2303_FcRIII_bunseki_AS_100min.lcm		
Batch Filename	:		
Vial #	: 19	Sample Type	: Unknown
Injection Volume	: 20 uL		
Date Acquired	: 2023/03/18 14:34:21	Acquired by	: System Administrator
Date Processed	: 2023/03/28 10:12:47	Processed by	: System Administrator

<Chromatogram>

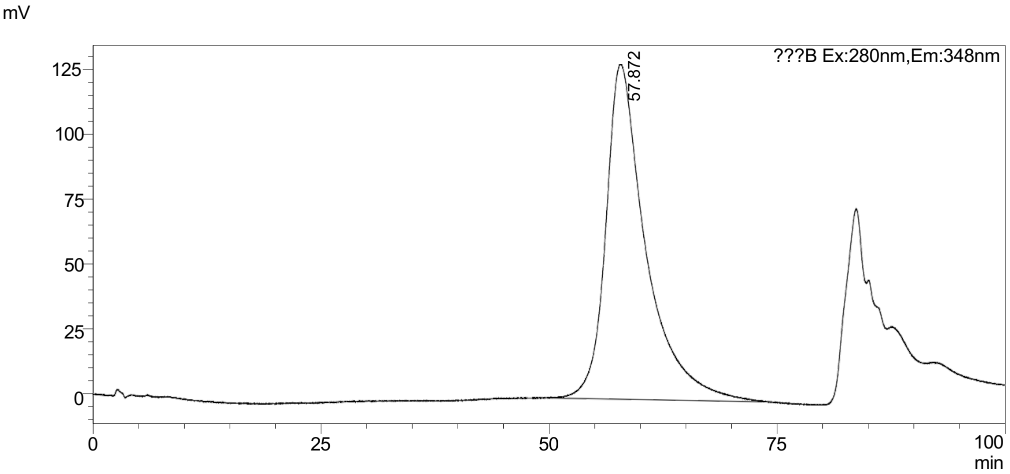

<Peak Table>

???B Ex:280nm,Em:348nm

Peak#	Ret. Time	Area	Height	Conc.	Unit	Mark	Name
1	57.872	39456502	128881	100.000		M	

2-10 [A1a-F/M3-F]

Analysis Report

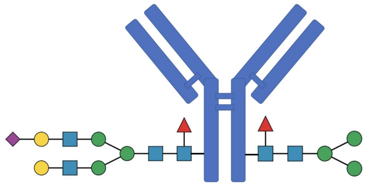

<Sample Information>

Sample Name : 230313_20_A1a_M3
Sample ID : 230313_20_A1a_M3
Data Filename : 230313_20_A1a_M3.lcd
Method Filename : 2303_FcRIII_bunseki_AS_100min.lcm
Batch Filename :
Vial # : 20
Injection Volume : 20 uL
Date Acquired : 2023/03/18 16:20:21
Date Processed : 2023/03/28 10:23:43

Sample Type : Unknown
Acquired by : System Administrator
Processed by : System Administrator

<Chromatogram>

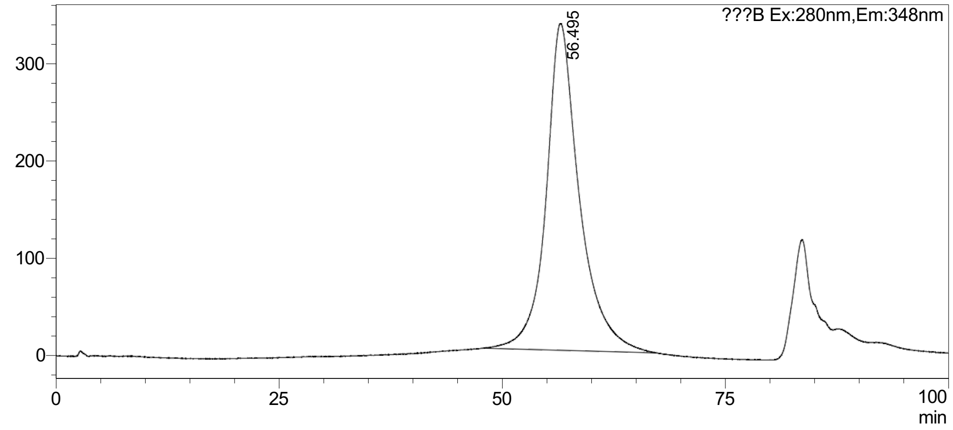

<Peak Table>

???B Ex:280nm,Em:348nm

Peak#	Ret. Time	Area	Height	Conc.	Unit	Mark	Name
1	56.495	87184321	335601	100.000		M	

2-11 [A1a-F/GlcNAc-F]

Analysis Report

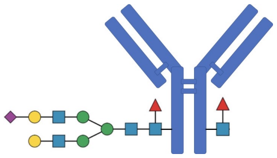

<Sample Information>

Sample Name	: 230313_21_A1a_GlcNAc
Sample ID	: 230313_21_A1a_GlcNAc
Data Filename	: 230313_21_A1a_GlcNAc.lcd
Method Filename	: 2303_FcRIII_bunseki_AS_100min.lcm
Batch Filename	:
Vial #	: 21
Injection Volume	: 20 uL
Date Acquired	: 2023/03/27 12:37:07
Date Processed	: 2023/03/28 10:24:59

Sample Type	: Unknown
Acquired by	: System Administrator
Processed by	: System Administrator

<Chromatogram>

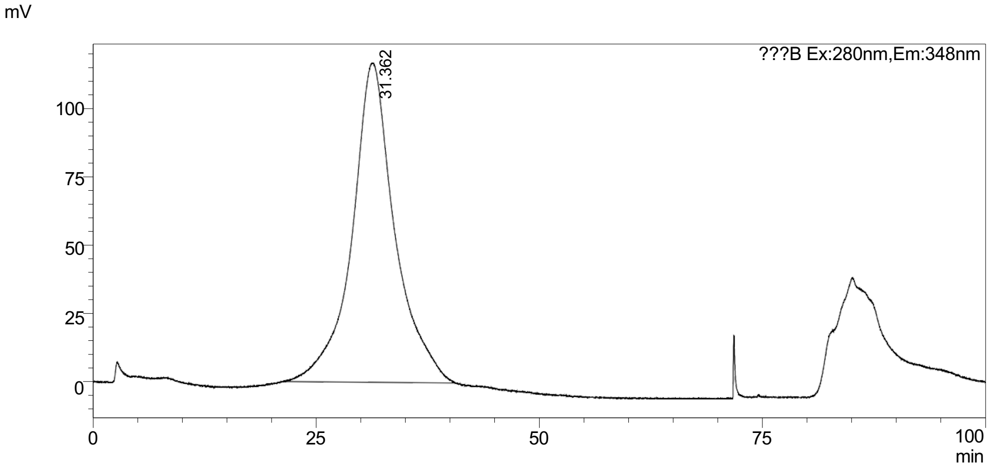

<Peak Table>

???B Ex:280nm,Em:348nm

Peak#	Ret. Time	Area	Height	Conc.	Unit	Mark	Name
1	31.362	38989906	116985	100.000		M	

3-3 [A1b-F/A1b-F]

Analysis Report

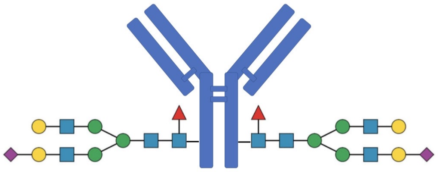

<Sample Information>

Sample Name	: 230313_22_A1b_A1b		
Sample ID	: 230313_22_A1b_A1b		
Data Filename	: 230313_22_A1b_A1b001.lcd		
Method Filename	: 2303_FcRIII_bunseki_AS_100min.lcm		
Batch Filename	:		
Vial #	: 22	Sample Type	: Unknown
Injection Volume	: 20 uL		
Date Acquired	: 2023/03/20 11:23:07	Acquired by	: System Administrator
Date Processed	: 2023/03/28 10:26:01	Processed by	: System Administrator

<Chromatogram>

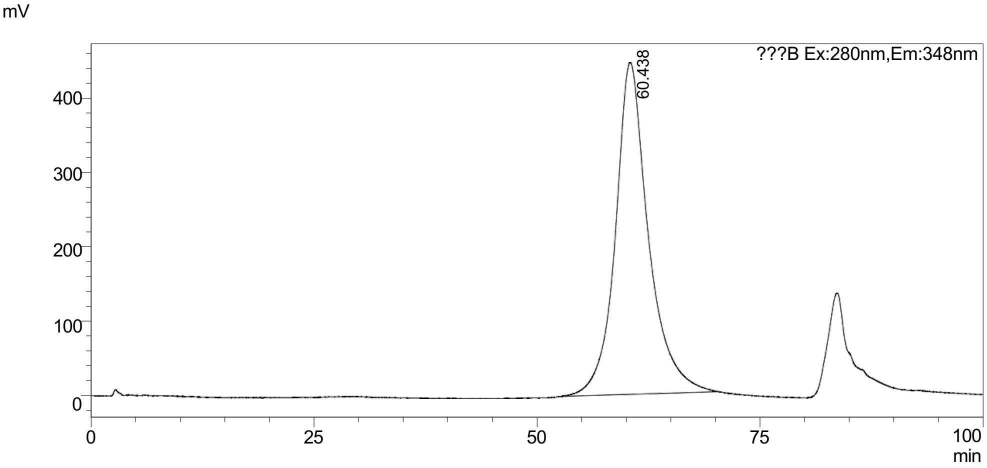

<Peak Table>

???B Ex:280nm,Em:348nm

Peak#	Ret. Time	Area	Height	Conc.	Unit	Mark	Name
1	60.438	114913285	446322	100.000		M	

3-4 [A1b-F/A1a-Gal-F]

Analysis Report

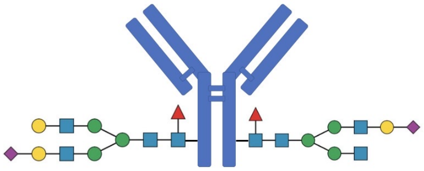

<Sample Information>

Sample Name	: 230313_23_A1b_A1a-G
Sample ID	: 230313_23_A1b_A1a-G
Data Filename	: 230313_23_A1b_A1a-G.lcd
Method Filename	: 2303_FcRIII_bunseki_AS_100min.lcm
Batch Filename	:
Vial #	: 23
Injection Volume	: 20 uL
Date Acquired	: 2023/03/20 13:07:06
Date Processed	: 2023/03/28 10:27:08

Sample Type	: Unknown
Acquired by	: System Administrator
Processed by	: System Administrator

<Chromatogram>

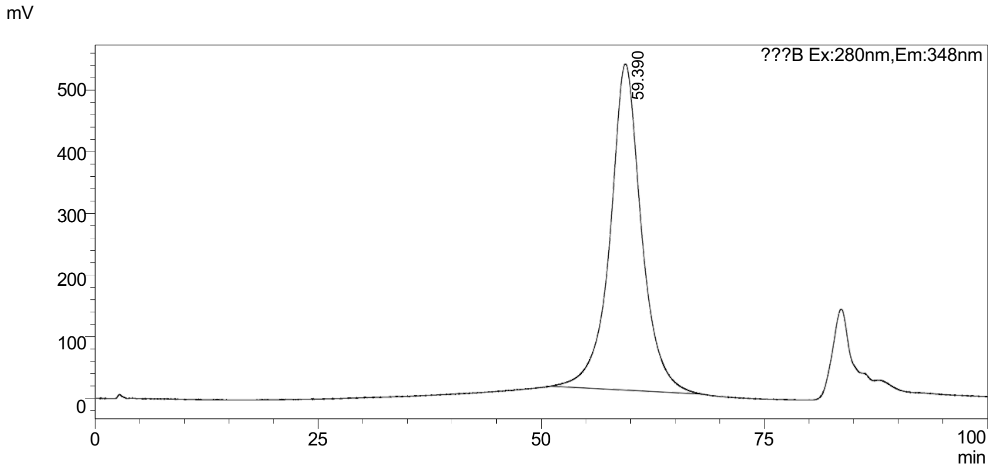

<Peak Table>

???B Ex:280nm,Em:348nm

Peak#	Ret. Time	Area	Height	Conc.	Unit	Mark	Name
1	59.390	129802697	529397	100.000		M	

3-5 [A1b-F/A1b-Gal-F]

Analysis Report

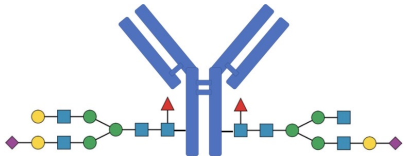

<Sample Information>

Sample Name	: 230313_24_A1b_A1b-G		
Sample ID	: 230313_24_A1b_A1b-G		
Data Filename	: 230313_24_A1b_A1b-G.lcd		
Method Filename	: 2303_FcRIII_bunseki_AS_100min.lcm		
Batch Filename	:		
Vial #	: 24	Sample Type	: Unknown
Injection Volume	: 20 uL		
Date Acquired	: 2023/03/20 14:51:54	Acquired by	: System Administrator
Date Processed	: 2023/03/28 10:30:46	Processed by	: System Administrator

<Chromatogram>

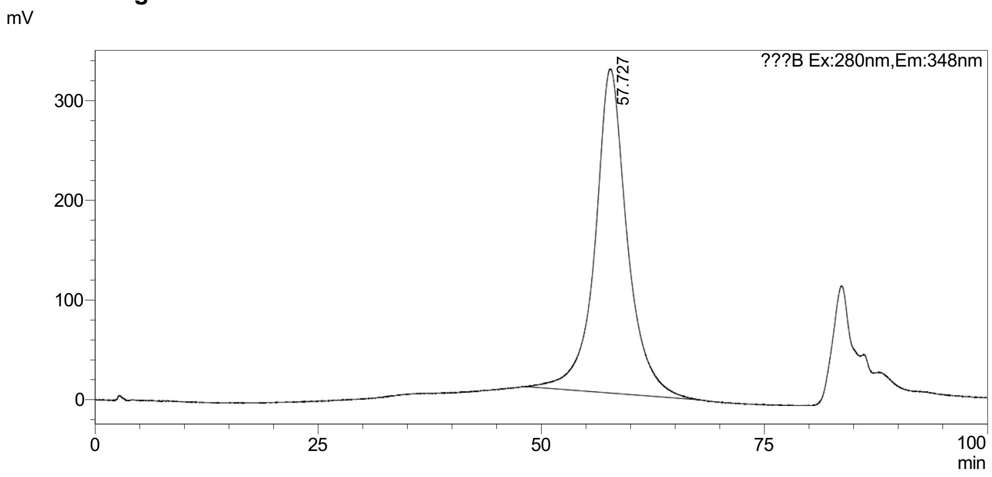

<Peak Table>

???B Ex:280nm,Em:348nm

Peak#	Ret. Time	Area	Height	Conc.	Unit	Mark	Name
1	57.727	81670485	324690	100.000		M	

3-6 [A1b-F/G2-F]

Analysis Report

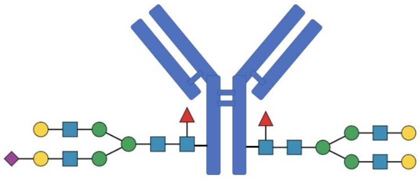

<Sample Information>

Sample Name	: 230313_25_A1b_G2		
Sample ID	: 230313_25_A1b_G2		
Data Filename	: 230313_25_A1b_G2.lcd		
Method Filename	: 2303_FcRIII_bunseki_AS_100min.lcm		
Batch Filename	:		
Vial #	: 25	Sample Type	: Unknown
Injection Volume	: 20 uL		
Date Acquired	: 2023/03/22 9:26:23	Acquired by	: System Administrator
Date Processed	: 2023/03/28 10:31:46	Processed by	: System Administrator

<Chromatogram>

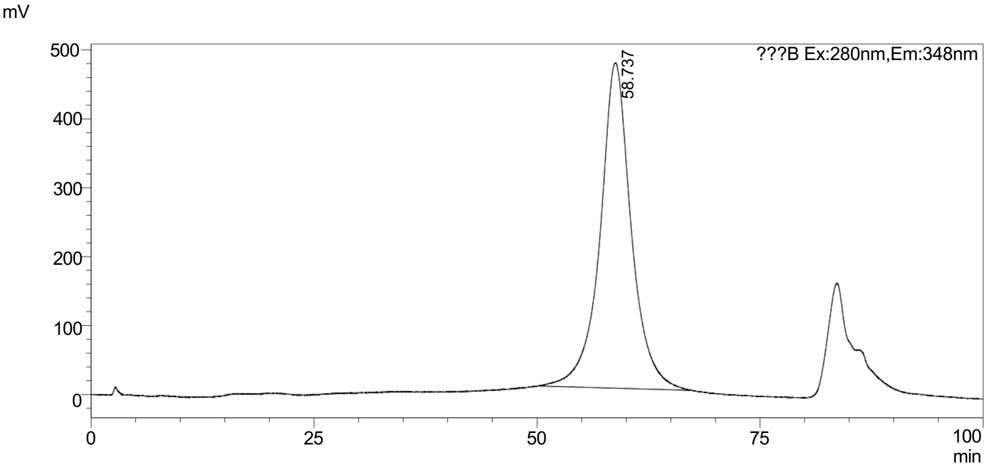

<Peak Table>

???B Ex:280nm,Em:348nm

Peak#	Ret. Time	Area	Height	Conc.	Unit	Mark	Name
1	58.737	115226885	472301	100.000		M	

3-7 [A1b-F/G1a-F]

Analysis Report

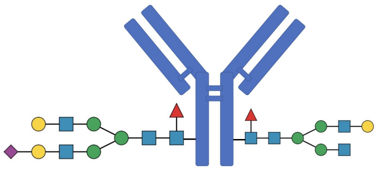

<Sample Information>

Sample Name	: 230313_26_A1b_G1a		
Sample ID	: 230313_26_A1b_G1a		
Data Filename	: 230313_26_A1b_G1a.lcd		
Method Filename	: 2303_FcRIII_bunseki_AS_100min.lcm		
Batch Filename	:		
Vial #	: 26	Sample Type	: Unknown
Injection Volume	: 20 uL		
Date Acquired	: 2023/03/22 11:11:25	Acquired by	: System Administrator
Date Processed	: 2023/03/28 10:32:53	Processed by	: System Administrator

<Chromatogram>

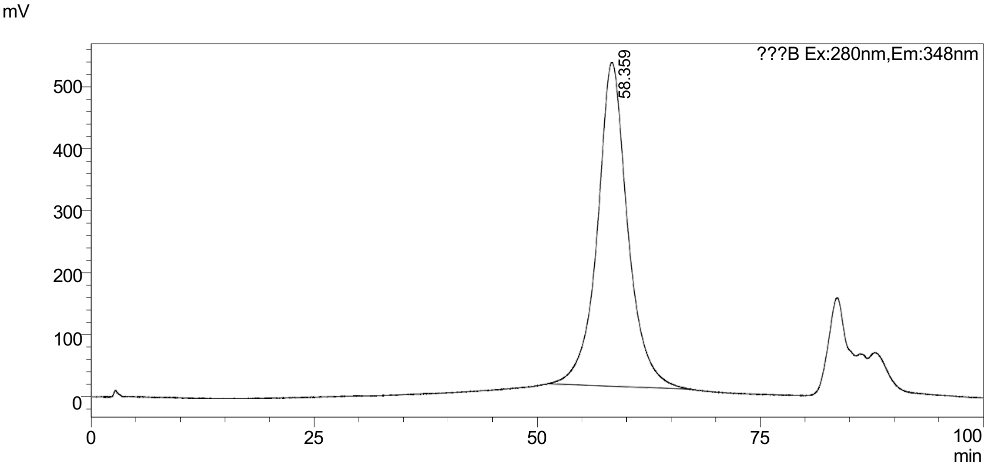

<Peak Table>

???B Ex:280nm,Em:348nm

Peak#	Ret. Time	Area	Height	Conc.	Unit	Mark	Name
1	58.359	123800179	521890	100.000		M	

3-8 [A1b-F/G1b-F]

Analysis Report

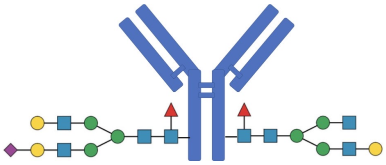

<Sample Information>

Sample Name	: 230313_27_A1b_G1b		
Sample ID	: 230313_27_A1b_G1b		
Data Filename	: 230313_27_A1b_G1b.lcd		
Method Filename	: 2303_FcRIII_bunseki_AS_100min.lcm		
Batch Filename	:		
Vial #	: 27	Sample Type	: Unknown
Injection Volume	: 20 uL		
Date Acquired	: 2023/03/22 12:58:20	Acquired by	: System Administrator
Date Processed	: 2023/03/28 10:34:20	Processed by	: System Administrator

<Chromatogram>

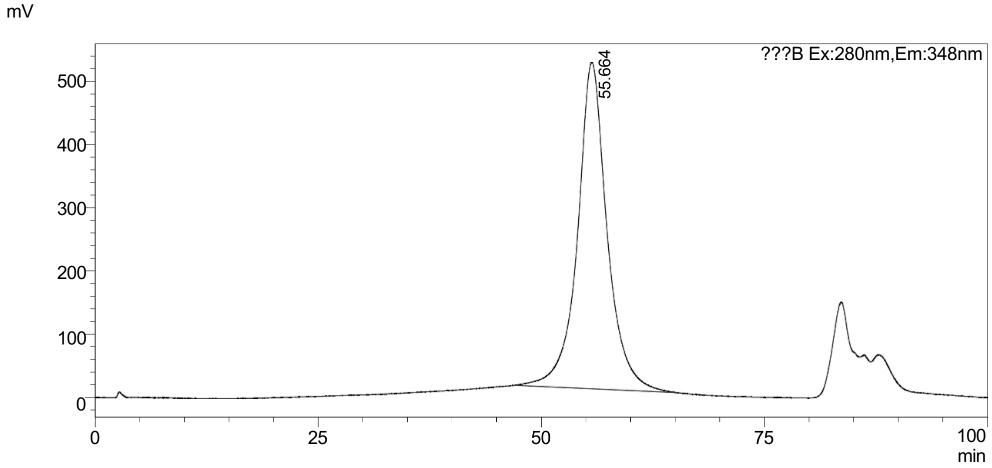

<Peak Table>

???B Ex:280nm,Em:348nm

Peak#	Ret. Time	Area	Height	Conc.	Unit	Mark	Name
1	55.664	119454981	515967	100.000		M	

3-9 [A1b-F/G0-F]

Analysis Report

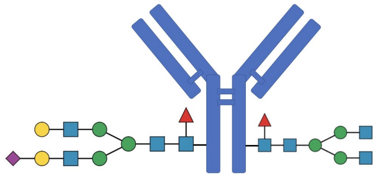

<Sample Information>

Sample Name	: 230313_28_A1b_G0		
Sample ID	: 230313_28_A1b_G0		
Data Filename	: 230313_28_A1b_G0.lcd		
Method Filename	: 2303_FcRIII_bunseki_AS_100min.lcm		
Batch Filename	:		
Vial #	: 28	Sample Type	: Unknown
Injection Volume	: 20 uL		
Date Acquired	: 2023/03/22 14:43:35	Acquired by	: System Administrator
Date Processed	: 2023/03/28 10:35:10	Processed by	: System Administrator

<Chromatogram>

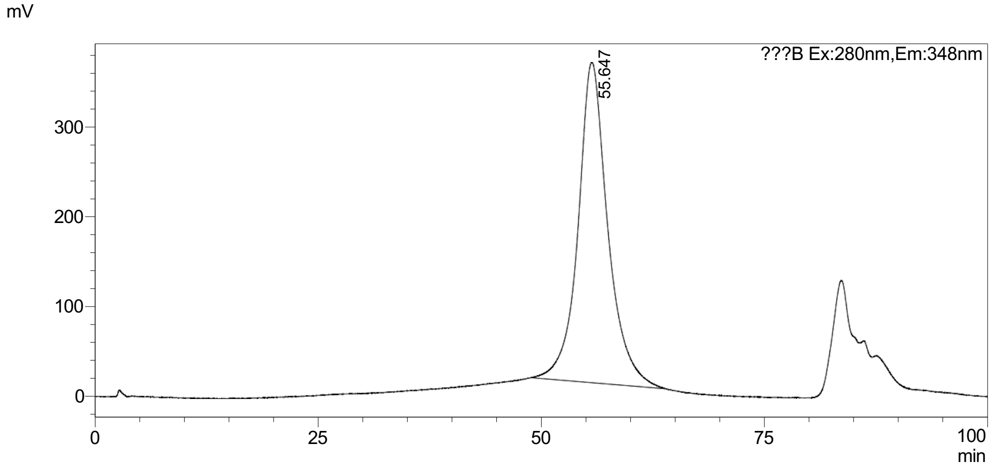

<Peak Table>

???B Ex:280nm,Em:348nm

Peak#	Ret. Time	Area	Height	Conc.	Unit	Mark	Name
1	55.647	82781889	356639	100.000		M	

3-10 [A1b-F/M3-F]

Analysis Report

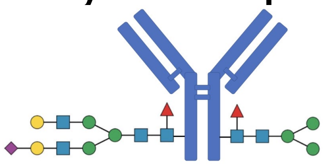

<Sample Information>

Sample Name	: 230313_29_A1b_M3		
Sample ID	: 230313_29_A1b_M3		
Data Filename	: 230313_29_A1b_M3.lcd		
Method Filename	: 2303_FcRIII_bunseki_AS_100min.lcm		
Batch Filename	:		
Vial #	: 29	Sample Type	: Unknown
Injection Volume	: 20 uL		
Date Acquired	: 2023/03/22 16:28:13	Acquired by	: System Administrator
Date Processed	: 2023/03/28 10:36:18	Processed by	: System Administrator

<Chromatogram>

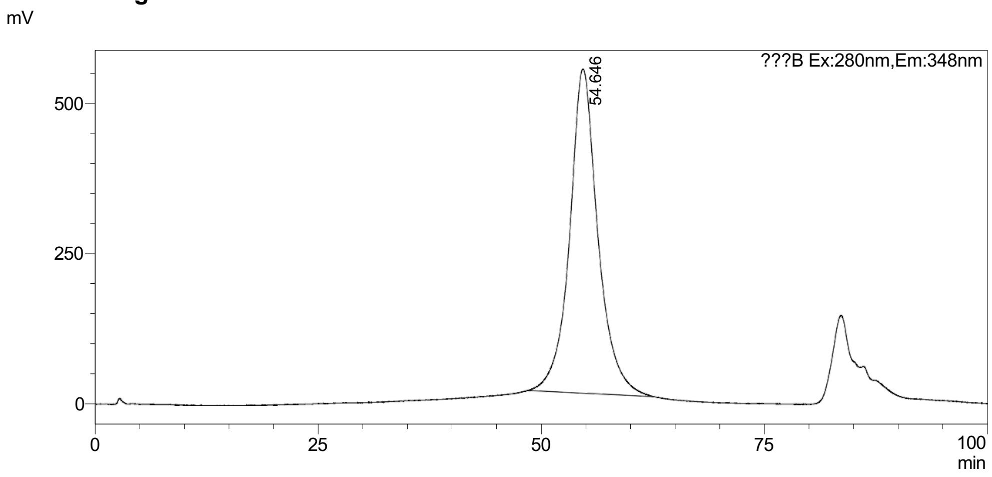

<Peak Table>

???B Ex:280nm,Em:348nm

Peak#	Ret. Time	Area	Height	Conc.	Unit	Mark	Name
1	54.646	119173386	539346	100.000		M	

3-11 [A1b-F/GlcNAc-F]

Analysis Report

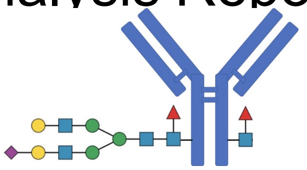

<Sample Information>

Sample Name	: 230313_30_A1b_GlcNAc		
Sample ID	: 230313_30_A1b_GlcNAc		
Data Filename	: 230313_30_A1b_GlcNAc.lcd		
Method Filename	: 2303_FcRIII_bunseki_AS_100min.lcm		
Batch Filename	:		
Vial #	: 30	Sample Type	: Unknown
Injection Volume	: 20 uL		
Date Acquired	: 2023/03/27 14:21:09	Acquired by	: System Administrator
Date Processed	: 2023/03/28 10:41:22	Processed by	: System Administrator

<Chromatogram>

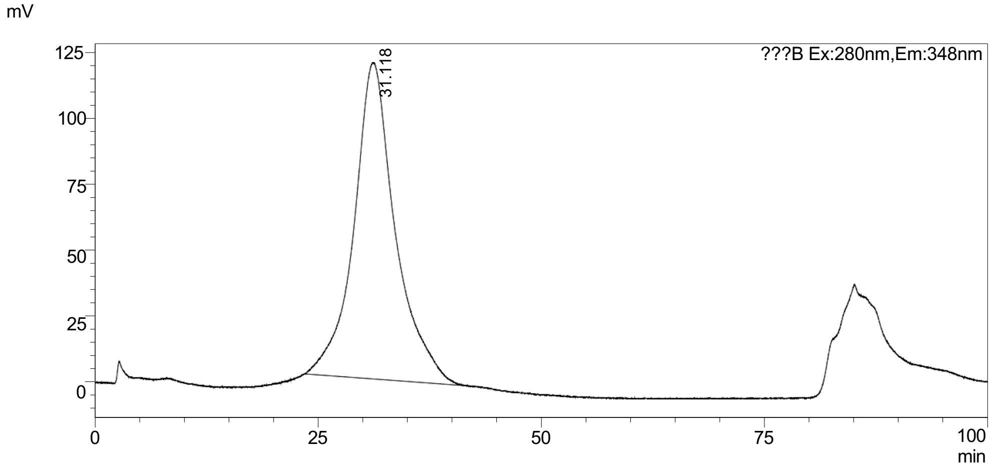

<Peak Table>

???B Ex:280nm,Em:348nm

Peak#	Ret. Time	Area	Height	Conc.	Unit	Mark	Name
1	31.118	38666385	120000	100.000		M	

4-4 [A1a-Gal-F/A1a-Gal-F]

Analysis Report

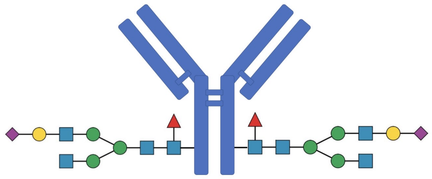

<Sample Information>

Sample Name	: 230313_31_A1a-G_A1a-G		
Sample ID	: 230313_31_A1a-G_A1a-G		
Data Filename	: 230313_31_A1a-G_A1a-G.lcd		
Method Filename	: 2303_FcRIII_bunseki_AS_100min.lcm		
Batch Filename	:		
Vial #	: 31	Sample Type	: Unknown
Injection Volume	: 20 uL		
Date Acquired	: 2023/03/22 18:46:38	Acquired by	: System Administrator
Date Processed	: 2023/03/28 10:42:51	Processed by	: System Administrator

<Chromatogram>

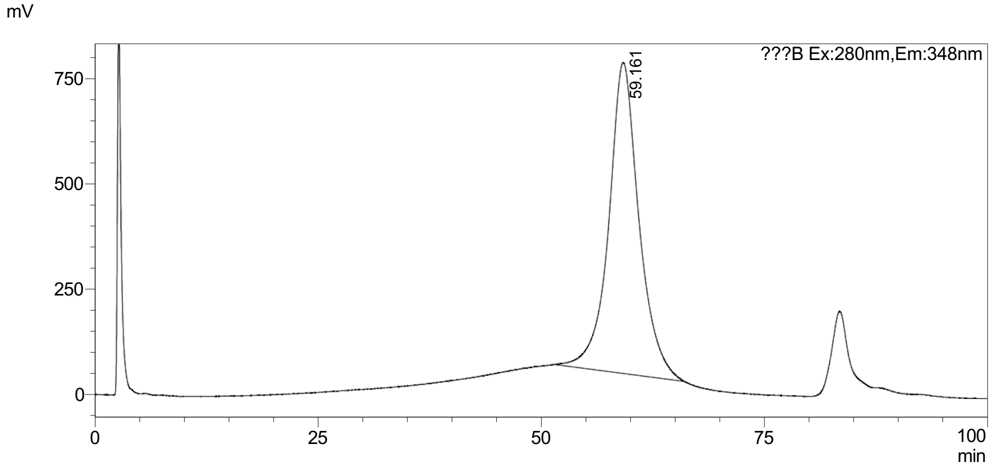

<Peak Table>

???B Ex:280nm,Em:348nm

Peak#	Ret. Time	Area	Height	Conc.	Unit	Mark	Name
1	59.161	174190167	737852	100.000		M	

4-5 [A1a-Gal-F/A1b-Gal-F]

Analysis Report

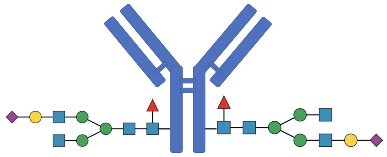

<Sample Information>

Sample Name	: 230313_32_A1a-G_A1b-G
Sample ID	: 230313_32_A1a-G_A1b-G
Data Filename	: 230313_32_A1a-G_A1b-G.lcd
Method Filename	: 2303_FcRIII_bunseki_AS_100min.lcm
Batch Filename	:
Vial #	: 32
Injection Volume	: 20 uL
Date Acquired	: 2023/03/22 20:56:45
Date Processed	: 2023/03/28 10:43:52

Sample Type	: Unknown
Acquired by	: System Administrator
Processed by	: System Administrator

<Chromatogram>

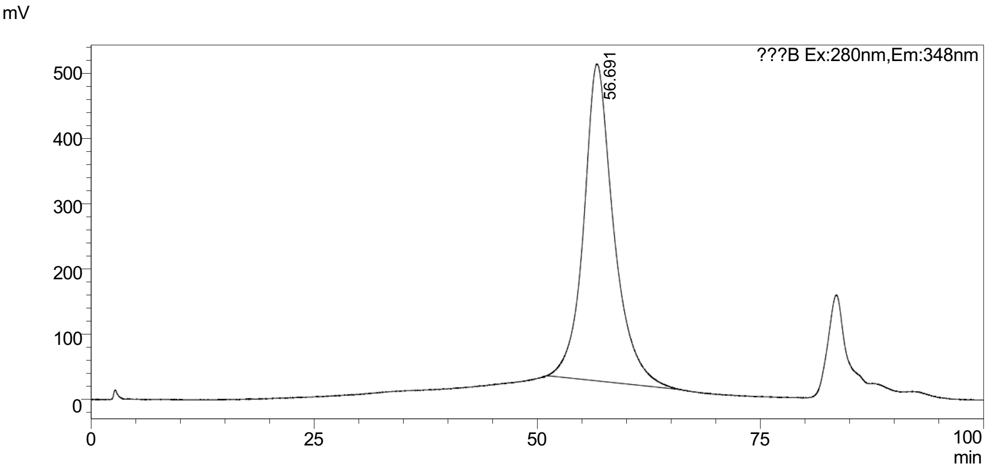

<Peak Table>

???B Ex:280nm,Em:348nm

Peak#	Ret. Time	Area	Height	Conc.	Unit	Mark	Name
1	56.691	114137500	486254	100.000		M	

4-6 [A1a-Gal-F/G2-F]

Analysis Report

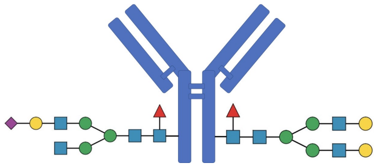

<Sample Information>

Sample Name	: 230313_33_A1a-G_G2
Sample ID	: 230313_33_A1a-G_G2
Data Filename	: 230313_33_A1a-G_G2.lcd
Method Filename	: 2303_FcRIII_bunseki_AS_100min.lcm
Batch Filename	:
Vial #	: 33
Injection Volume	: 20 uL
Date Acquired	: 2023/03/23 9:32:37
Date Processed	: 2023/03/28 10:44:48

Sample Type : Unknown

Acquired by : System Administrator

Processed by : System Administrator

<Chromatogram>

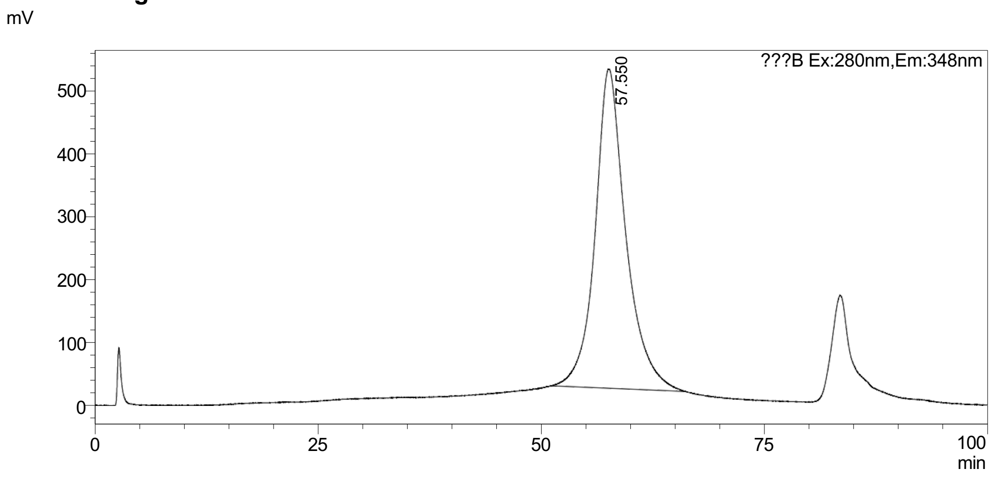

<Peak Table>

???B Ex:280nm,Em:348nm

Peak#	Ret. Time	Area	Height	Conc.	Unit	Mark	Name
1	57.550	121844268	507635	100.000		M	

4-7 [A1a-Gal-F/G1a-F]

Analysis Report

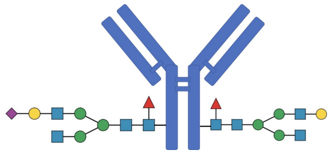

<Sample Information>

Sample Name	: 230313_34_A1a-G_G1a
Sample ID	: 230313_34_A1a-G_G1a
Data Filename	: 230313_34_A1a-G_G1a.lcd
Method Filename	: 2303_FcRIII_bunseki_AS_100min.lcm
Batch Filename	:
Vial #	: 34
Injection Volume	: 20 uL
Date Acquired	: 2023/03/23 11:16:43
Date Processed	: 2023/03/28 10:45:55
Sample Type	: Unknown
Acquired by	: System Administrator
Processed by	: System Administrator

<Chromatogram>

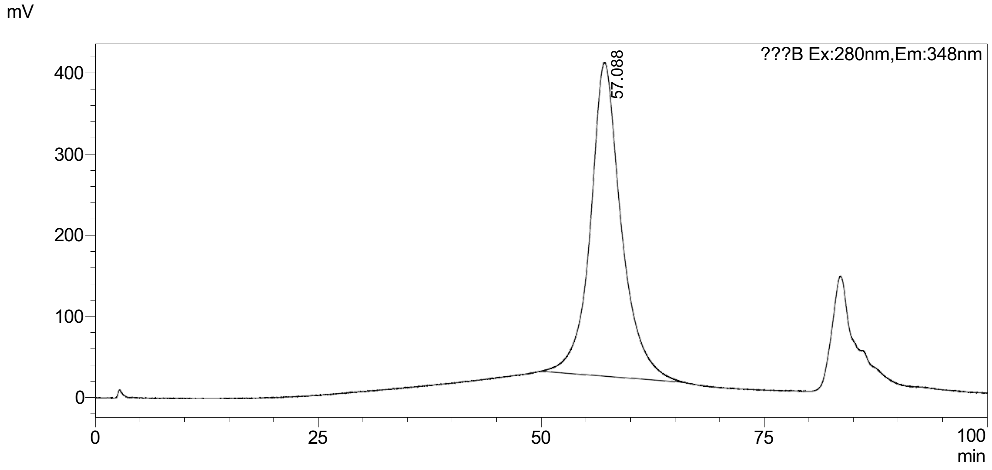

<Peak Table>

???B Ex:280nm,Em:348nm

Peak#	Ret. Time	Area	Height	Conc.	Unit	Mark	Name
1	57.088	93162833	386074	100.000		M	

4-8 [A1a-Gal-F/G1b-F]

Analysis Report

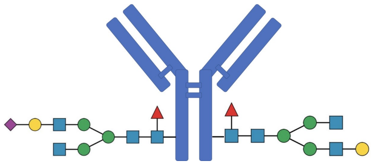

<Sample Information>

Sample Name	: 230313_35_A1a-G_G1b
Sample ID	: 230313_35_A1a-G_G1b
Data Filename	: 230313_35_A1a-G_G1b.lcd
Method Filename	: 2303_FcRIII_bunseki_AS_100min.lcm
Batch Filename	:
Vial #	: 35
Injection Volume	: 20 uL
Date Acquired	: 2023/03/23 13:00:32
Date Processed	: 2023/03/28 10:48:44
Sample Type	: Unknown
Acquired by	: System Administrator
Processed by	: System Administrator

<Chromatogram>

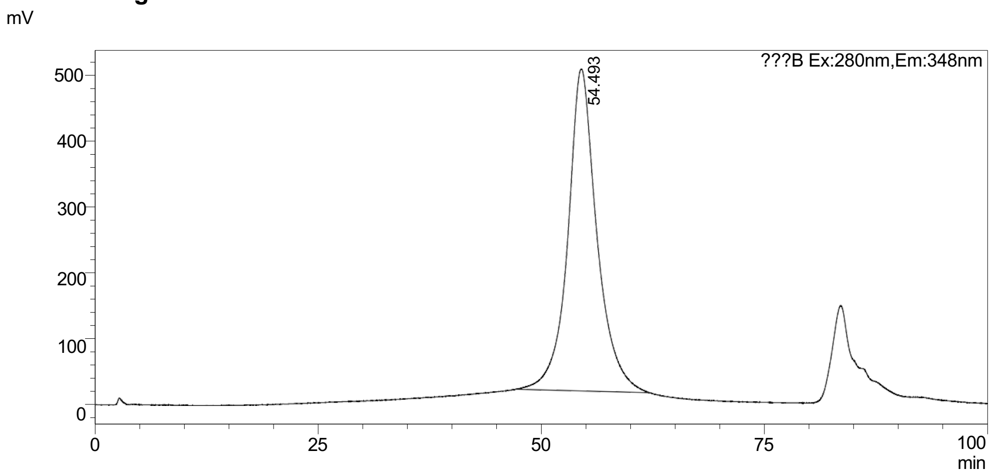

<Peak Table>

???B Ex:280nm,Em:348nm

Peak#	Ret. Time	Area	Height	Conc.	Unit	Mark	Name
1	54.493	110880695	488474	100.000		M	

4-9 [A1a-Gal-F/G0-F]

Analysis Report

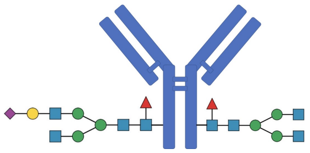

<Sample Information>

Sample Name	: 230313_36_A1a-G_G0		
Sample ID	: 230313_36_A1a-G_G0		
Data Filename	: 230313_36_A1a-G_G0.lcd		
Method Filename	: 2303_FcRIII_bunseki_AS_100min.lcm		
Batch Filename	:		
Vial #	: 36	Sample Type	: Unknown
Injection Volume	: 20 uL		
Date Acquired	: 2023/03/23 14:44:27	Acquired by	: System Administrator
Date Processed	: 2023/03/28 10:50:02	Processed by	: System Administrator

<Chromatogram>

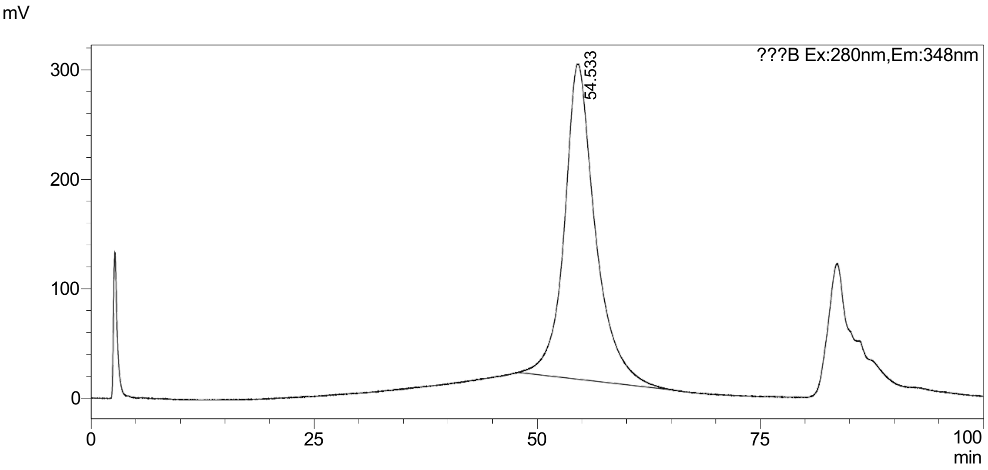

<Peak Table>

???B Ex:280nm,Em:348nm

Peak#	Ret. Time	Area	Height	Conc.	Unit	Mark	Name
1	54.533	69918343	288158	100.000		M	

4-10 [A1a-Gal-F/M3-F]

Analysis Report

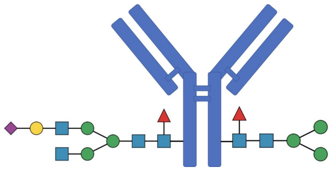

<Sample Information>

Sample Name	: 230313_37_A1a-G_M3		
Sample ID	: 230313_37_A1a-G_M3		
Data Filename	: 230313_37_A1a-G_M3.lcd		
Method Filename	: 2303_FcRIII_bunseki_AS_100min.lcm		
Batch Filename	:		
Vial #	: 37	Sample Type	: Unknown
Injection Volume	: 20 uL		
Date Acquired	: 2023/03/23 16:32:46	Acquired by	: System Administrator
Date Processed	: 2023/03/28 10:51:12	Processed by	: System Administrator

<Chromatogram>

mV

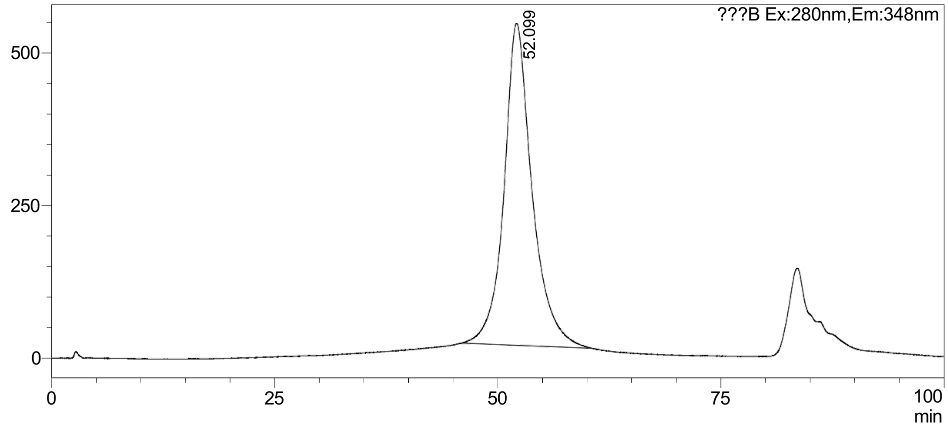

<Peak Table>

???B Ex:280nm,Em:348nm

Peak#	Ret. Time	Area	Height	Conc.	Unit	Mark	Name
1	52.099	115624201	527668	100.000		M	

4-11 [A1a-Gal-F/GlcNAc-F]

Analysis Report

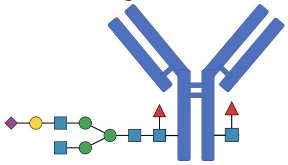

<Sample Information>

Sample Name	: 230313_38_A1a-G_GlcNAc		
Sample ID	: 230313_38_A1a-G_GlcNAc		
Data Filename	: 230313_38_A1a-G_GlcNAc.lcd		
Method Filename	: 2303_FcRIII_bunseki_AS_100min.lcm		
Batch Filename	:		
Vial #	: 38	Sample Type	: Unknown
Injection Volume	: 20 uL		
Date Acquired	: 2023/03/27 16:04:58	Acquired by	: System Administrator
Date Processed	: 2023/03/28 10:52:28	Processed by	: System Administrator

<Chromatogram>

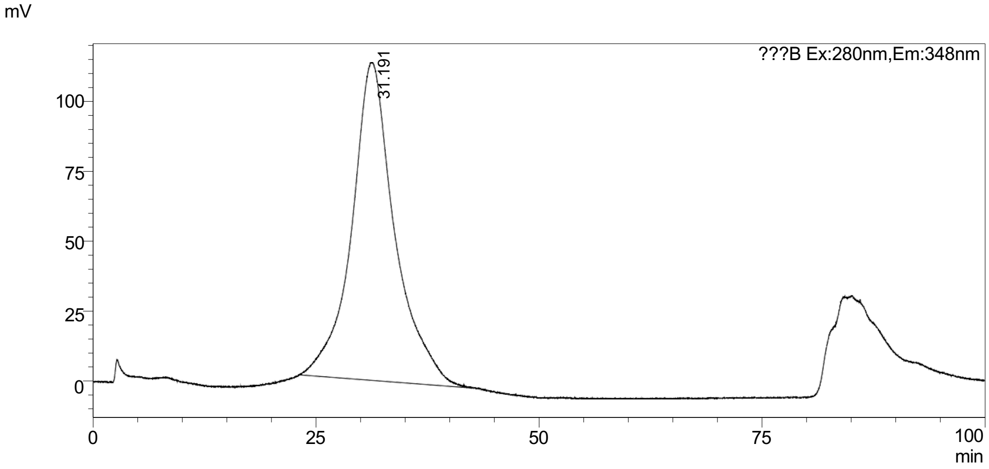

<Peak Table>

???B Ex:280nm,Em:348nm

Peak#	Ret. Time	Area	Height	Conc.	Unit	Mark	Name
1	31.191	38138746	113606	100.000		M	

5-5 [A1b-Gal-F/A1b-Gal-F]

Analysis Report

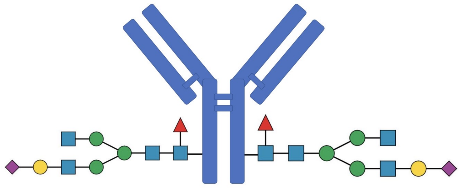

<Sample Information>

Sample Name	: 230313_39_A1b-G_A1b-G		
Sample ID	: 230313_39_A1b-G_A1b-G		
Data Filename	: 230313_39_A1b-G_A1b-G.lcd		
Method Filename	: 2303_FcRIII_bunseki_AS_100min.lcm		
Batch Filename	:		
Vial #	: 39	Sample Type	: Unknown
Injection Volume	: 20 uL		
Date Acquired	: 2023/03/23 18:17:00	Acquired by	: System Administrator
Date Processed	: 2023/03/28 10:53:33	Processed by	: System Administrator

<Chromatogram>

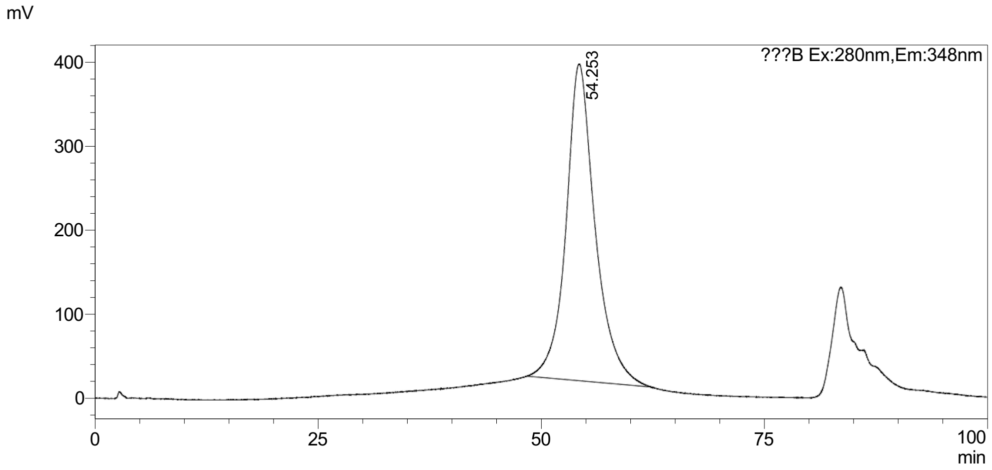

<Peak Table>

???B Ex:280nm,Em:348nm

Peak#	Ret. Time	Area	Height	Conc.	Unit	Mark	Name
1	54.253	85940905	377456	100.000		M	

5-6 [A1b-Gal-F/G2-F]

Analysis Report

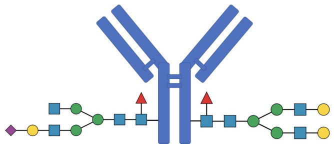

<Sample Information>

Sample Name : 230313_40_A1b-G_G2
Sample ID : 230313_40_A1b-G_G2
Data Filename : 230313_40_A1b-G_G2.lcd
Method Filename : 2303_FcRIII_bunseki_AS_100min.lcm
Batch Filename : 230323.lcb
Vial # : 40
Injection Volume : 20 uL
Date Acquired : 2023/03/23 20:02:02
Date Processed : 2023/03/28 11:02:12

Sample Type : Unknown
Acquired by : System Administrator
Processed by : System Administrator

<Chromatogram>

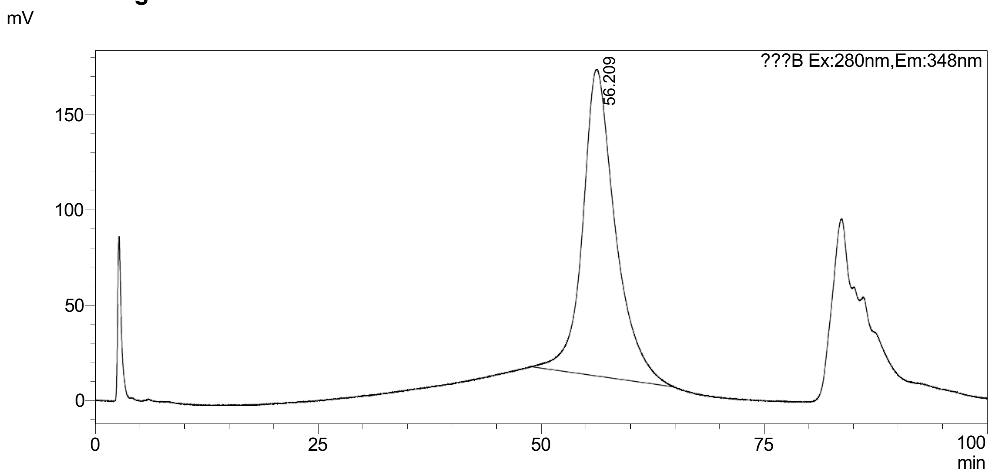

<Peak Table>

???B Ex:280nm,Em:348nm

Peak#	Ret. Time	Area	Height	Conc.	Unit	Mark	Name
1	56.209	41265044	161081	100.000		M	

5-7 [A1b-Gal-F/G1a-F]

Analysis Report

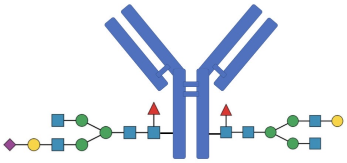

<Sample Information>

Sample Name	: 230313_40_A1b-G_G1a		
Sample ID	: 230313_40_A1b-G_G1a		
Data Filename	: 230313_41_A1b-G_G1a.lcd		
Method Filename	: 2303_FcRIII_bunseki_AS_100min.lcm		
Batch Filename	: 230323.lcb		
Vial #	: 41	Sample Type	: Unknown
Injection Volume	: 20 uL		
Date Acquired	: 2023/03/23 21:45:07	Acquired by	: System Administrator
Date Processed	: 2023/03/28 11:03:04	Processed by	: System Administrator

<Chromatogram>

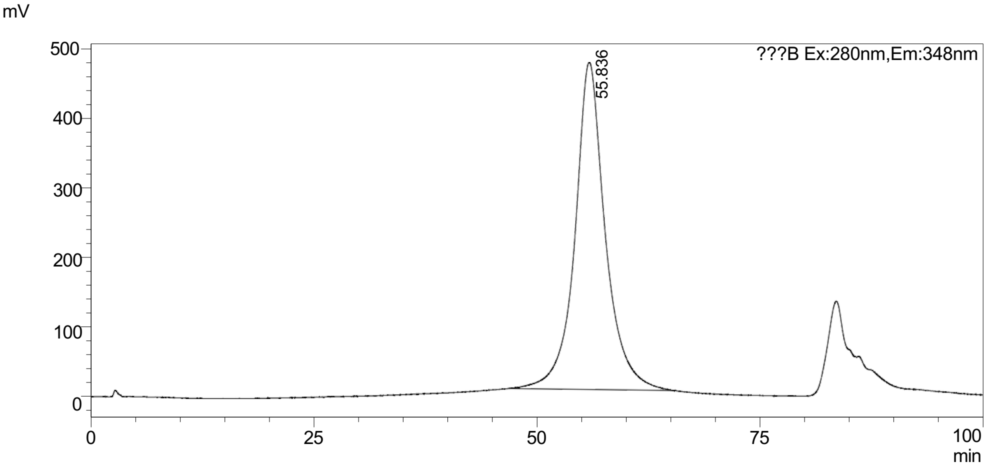

<Peak Table>

???B Ex:280nm,Em:348nm

Peak#	Ret. Time	Area	Height	Conc.	Unit	Mark	Name
1	55.836	111590085	470348	100.000		M	

5-8 [A1b-Gal-F/G1b-F]

Analysis Report

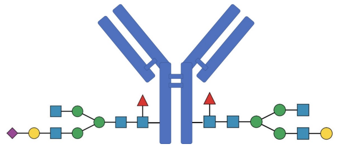

<Sample Information>

Sample Name	: 230313_42_A1b-G_G1b
Sample ID	: 230313_42_A1b-G_G1b
Data Filename	: 230313_42_A1b-G_G1b.lcd
Method Filename	: 2303_FcRIII_bunseki_AS_100min.lcm
Batch Filename	:
Vial #	: 42
Injection Volume	: 20 uL
Date Acquired	: 2023/03/24 9:11:07
Date Processed	: 2023/03/28 11:04:19

Sample Type	: Unknown
Acquired by	: System Administrator
Processed by	: System Administrator

<Chromatogram>

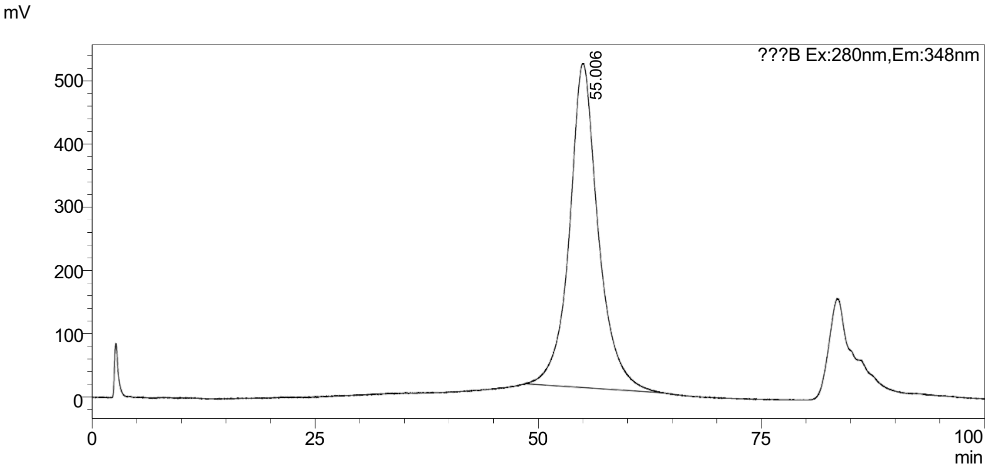

<Peak Table>

???B Ex:280nm,Em:348nm

Peak#	Ret. Time	Area	Height	Conc.	Unit	Mark	Name
1	55.006	115715348	512424	100.000		M	

5-9 [A1b-Gal-F/G0-F]

Analysis Report

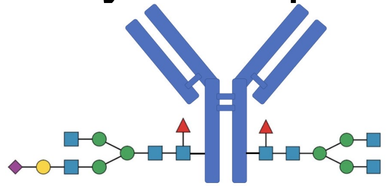

<Sample Information>

Sample Name	: 230313_43_A1b-G_G0		
Sample ID	: 230313_43_A1b-G_G0		
Data Filename	: 230313_43_A1b-G_G0.lcd		
Method Filename	: 2303_FcRIII_bunseki_AS_100min.lcm		
Batch Filename	:		
Vial #	: 43	Sample Type	: Unknown
Injection Volume	: 20 uL		
Date Acquired	: 2023/03/24 10:55:21	Acquired by	: System Administrator
Date Processed	: 2023/03/28 11:05:12	Processed by	: System Administrator

<Chromatogram>

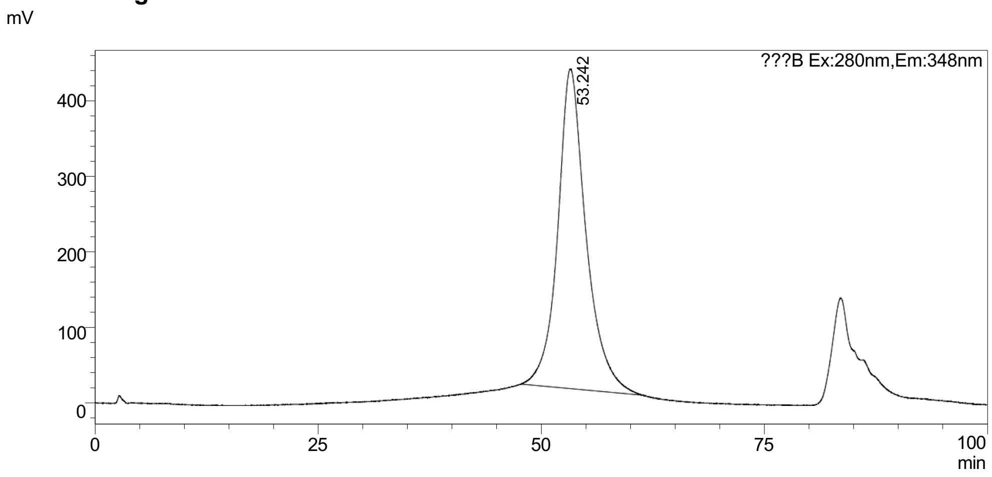

<Peak Table>

???B Ex:280nm,Em:348nm

Peak#	Ret. Time	Area	Height	Conc.	Unit	Mark	Name
1	53.242	93634248	422910	100.000		M	

5-10 [A1b-Gal-F/M3-F]

Analysis Report

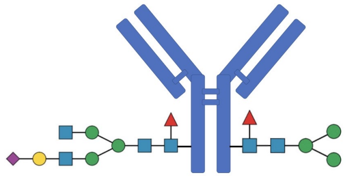

<Sample Information>

Sample Name	: 230313_44_A1b-G_M3		
Sample ID	: 230313_44_A1b-G_M3		
Data Filename	: 230313_44_A1b-G_M3.lcd		
Method Filename	: 2303_FcRIII_bunseki_AS_100min.lcm		
Batch Filename	:		
Vial #	: 44	Sample Type	: Unknown
Injection Volume	: 20 uL		
Date Acquired	: 2023/03/24 13:16:10	Acquired by	: System Administrator
Date Processed	: 2023/03/28 11:06:15	Processed by	: System Administrator

<Chromatogram>

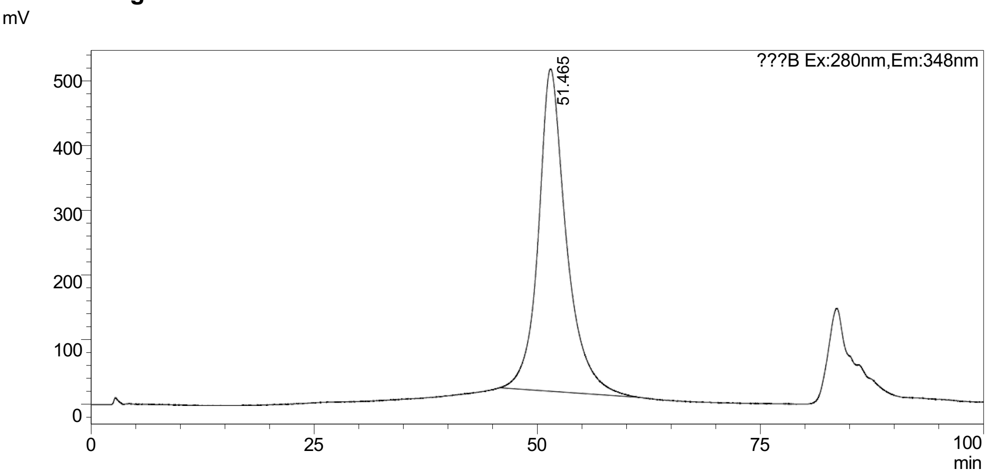

<Peak Table>

???B Ex:280nm,Em:348nm

Peak#	Ret. Time	Area	Height	Conc.	Unit	Mark	Name
1	51.465	109100373	497942	100.000		M	

5-11 [A1b-Gal-F/GlcNAc-F]

Analysis Report

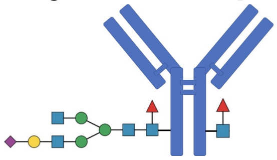

<Sample Information>

Sample Name	: 230313_45_A1b-G_GlcNAc		
Sample ID	: 230313_45_A1b-G_GlcNAc		
Data Filename	: 230313_45_A1b-G_GlcNAc.lcd		
Method Filename	: 2303_FcRIII_bunseki_AS_100min.lcm		
Batch Filename	:		
Vial #	: 45	Sample Type	: Unknown
Injection Volume	: 20 uL		
Date Acquired	: 2023/03/27 17:49:16	Acquired by	: System Administrator
Date Processed	: 2023/03/28 11:07:26	Processed by	: System Administrator

<Chromatogram>

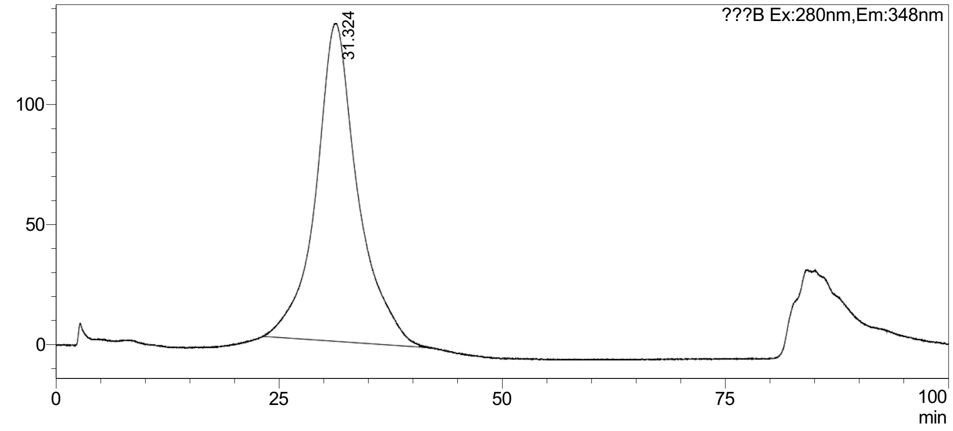

<Peak Table>

???B Ex:280nm,Em:348nm

Peak#	Ret. Time	Area	Height	Conc.	Unit	Mark	Name
1	31.324	43196672	132321	100.000		M	

6-6 [G2-F/G2-F]

Analysis Report

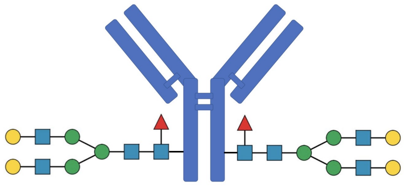

<Sample Information>

Sample Name : 230313_46_G2_G2
Sample ID : 230313_46_G2_G2
Data Filename : 230313_46_G2_G2.lcd
Method Filename : 2303_FcRIII_bunseki_AS_100min.lcm
Batch Filename :
Vial # : 46
Sample Type : Unknown
Injection Volume : 20 uL
Date Acquired : 2023/03/24 15:02:21
Acquired by : System Administrator
Date Processed : 2023/03/28 11:11:28
Processed by : System Administrator

<Chromatogram>

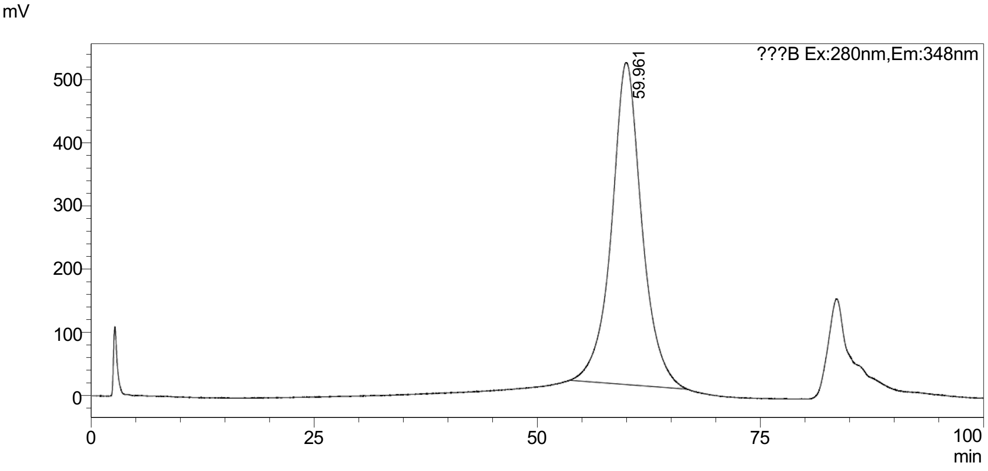

<Peak Table>

???B Ex:280nm,Em:348nm

Peak#	Ret. Time	Area	Height	Conc.	Unit	Mark	Name
1	59.961	120315957	509242	100.000		M	

6-7 [G2-F/G1a-F]

Analysis Report

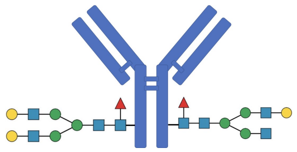

<Sample Information>

Sample Name	: 230313_47_G2_G1a		
Sample ID	: 230313_47_G2_G1a		
Data Filename	: 230313_47_G2_G1a.lcd		
Method Filename	: 2303_FcRIII_bunseki_AS_100min.lcm		
Batch Filename	:		
Vial #	: 47	Sample Type	: Unknown
Injection Volume	: 20 uL		
Date Acquired	: 2023/03/24 16:52:30	Acquired by	: System Administrator
Date Processed	: 2023/03/28 11:12:25	Processed by	: System Administrator

<Chromatogram>

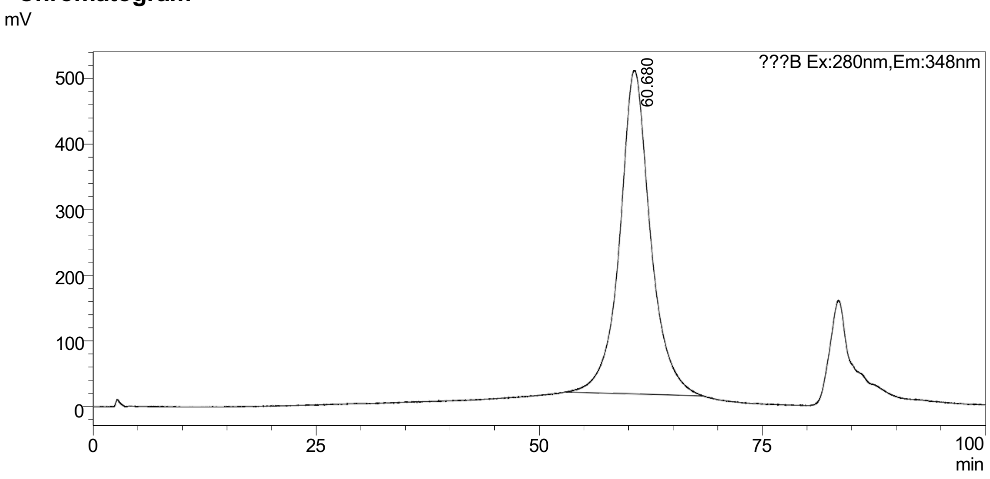

<Peak Table>

???B Ex:280nm,Em:348nm

Peak#	Ret. Time	Area	Height	Conc.	Unit	Mark	Name
1	60.680	118520592	492832	100.000		M	

6-8 [G2-F/G1b-F]

Analysis Report

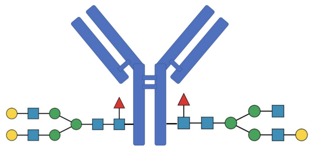

<Sample Information>

Sample Name	: 230313_48_G2_G1b
Sample ID	: 230313_48_G2_G1b
Data Filename	: 230313_48_G2_G1b.lcd
Method Filename	: 2303_FcRIII_bunseki_AS_100min.lcm
Batch Filename	:
Vial #	: 48
Injection Volume	: 20 uL
Date Acquired	: 2023/03/24 18:36:29
Date Processed	: 2023/03/28 11:13:20

Sample Type	: Unknown
Acquired by	: System Administrator
Processed by	: System Administrator

<Chromatogram>

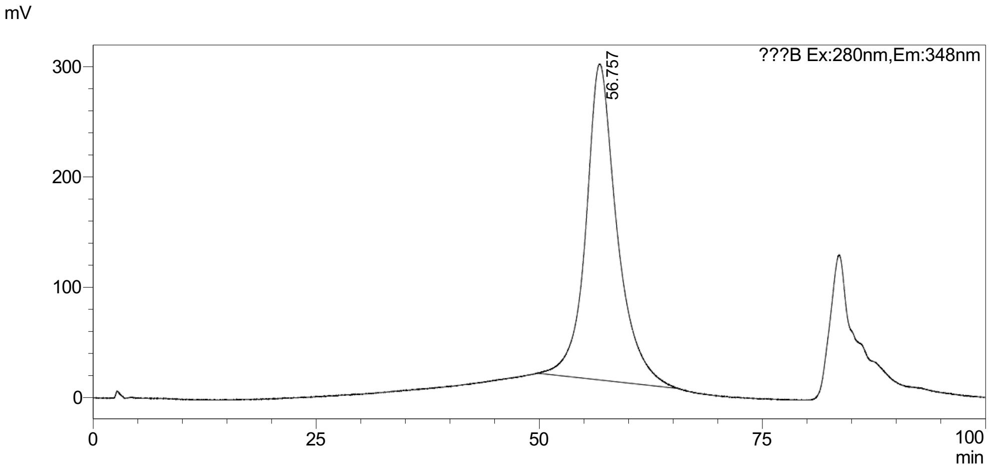

<Peak Table>

???B Ex:280nm,Em:348nm

Peak#	Ret. Time	Area	Height	Conc.	Unit	Mark	Name
1	56.757	69746154	286529	100.000		M	

6-9 [G2-F/G0-F]

Analysis Report

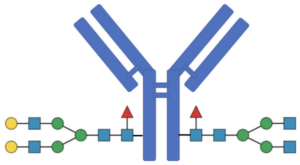

<Sample Information>

Sample Name : 230313_49_G2_G0
Sample ID : 230313_49_G2_G0
Data Filename : 230313_49_G2_G0.lcd
Method Filename : 2303_FcRIII_bunseki_AS_100min.lcm
Batch Filename :
Vial # : 49
Injection Volume : 20 uL
Date Acquired : 2023/03/24 20:20:36
Date Processed : 2023/03/28 11:14:35

Sample Type : Unknown
Acquired by : System Administrator
Processed by : System Administrator

<Chromatogram>

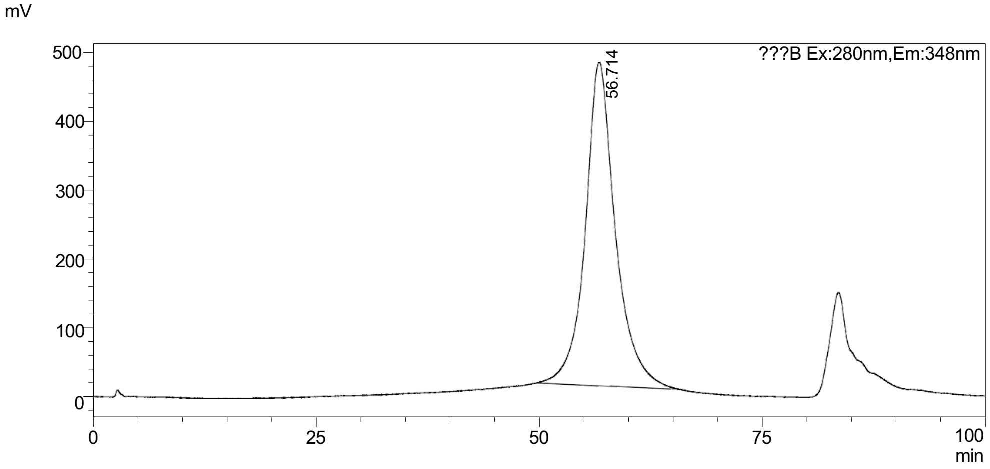

<Peak Table>

???B Ex:280nm,Em:348nm

Peak#	Ret. Time	Area	Height	Conc.	Unit	Mark	Name
1	56.714	110857047	470007	100.000		M	

6-10 [G2-F/M3-F]

Analysis Report

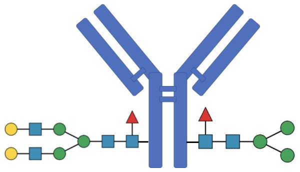

<Sample Information>

Sample Name	: 230313_50_G2_M3
Sample ID	: 230313_50_G2_M3
Data Filename	: 230313_50_G2_M3.lcd
Method Filename	: 2303_FcRIII_bunseki_AS_100min.lcm
Batch Filename	:
Vial #	: 50
Injection Volume	: 20 uL
Date Acquired	: 2023/03/25 9:13:13
Date Processed	: 2023/03/28 11:15:28
Sample Type	: Unknown
Acquired by	: System Administrator
Processed by	: System Administrator

<Chromatogram>

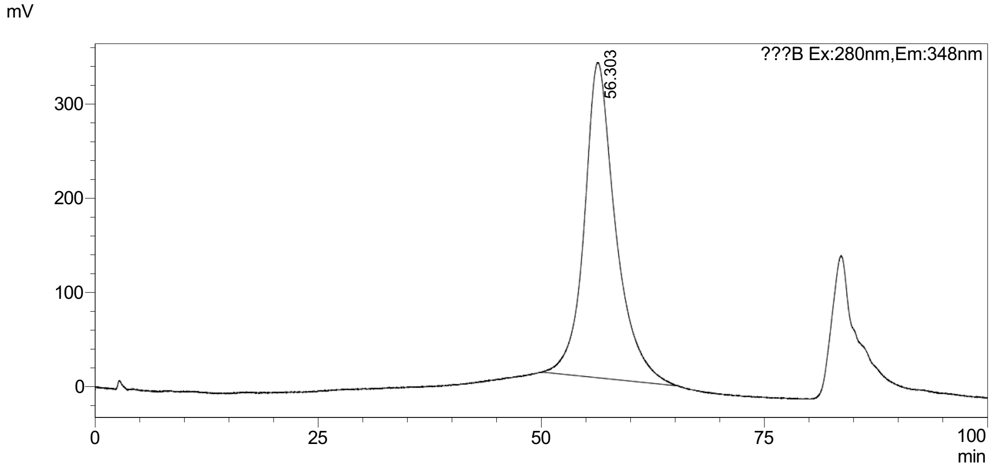

<Peak Table>

???B Ex:280nm,Em:348nm

Peak#	Ret. Time	Area	Height	Conc.	Unit	Mark	Name
1	56.303	81823972	334273	100.000		M	

6-11 [G2-F/GlcNAc-F]

Analysis Report

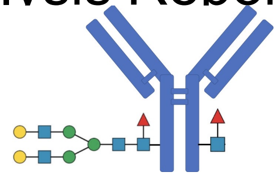

<Sample Information>

Sample Name	: 230313_51_G2_GlcNAc
Sample ID	: 230313_51_G2_GlcNAc
Data Filename	: 230313_51_G2_GlcNAc.lcd
Method Filename	: 2303_FcRIII_bunseki_AS_100min.lcm
Batch Filename	: 230327.lcb
Vial #	: 51
Injection Volume	: 20 uL
Date Acquired	: 2023/03/27 19:38:39
Date Processed	: 2023/03/28 11:18:40

Sample Type	: Unknown
Acquired by	: System Administrator
Processed by	: System Administrator

<Chromatogram>

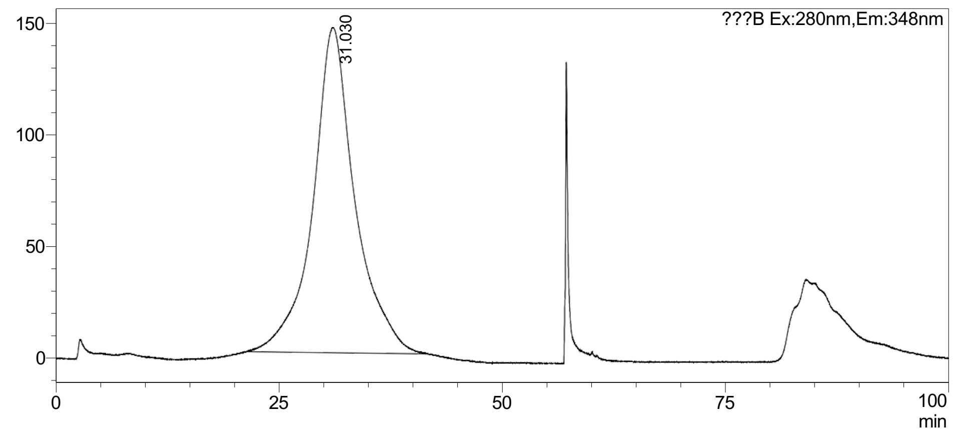

<Peak Table>

???B Ex:280nm,Em:348nm

Peak#	Ret. Time	Area	Height	Conc.	Unit	Mark	Name
1	31.030	47702587	145627	100.000		M	

7-7 [G1a-F/G1a-F]

Analysis Report

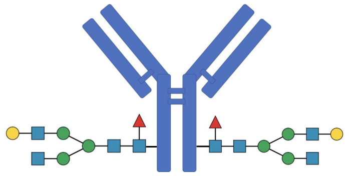

<Sample Information>

Sample Name	: 230313_52_G1a_G1a		
Sample ID	: 230313_52_G1a_G1a		
Data Filename	: 230313_52_G1a_G1a.lcd		
Method Filename	: 2303_FcRIII_bunseki_AS_100min.lcm		
Batch Filename	:		
Vial #	: 52	Sample Type	: Unknown
Injection Volume	: 20 uL		
Date Acquired	: 2023/03/25 14:26:21	Acquired by	: System Administrator
Date Processed	: 2023/03/28 11:19:29	Processed by	: System Administrator

<Chromatogram>

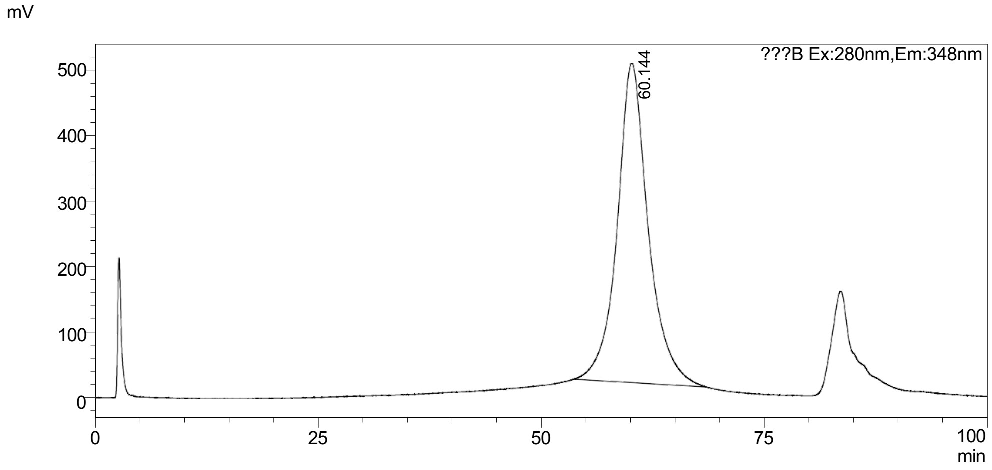

<Peak Table>

???B Ex:280nm,Em:348nm

Peak#	Ret. Time	Area	Height	Conc.	Unit	Mark	Name
1	60.144	118442669	488285	100.000		M	

7-8 [G1a-F/G1b-F]

Analysis Report

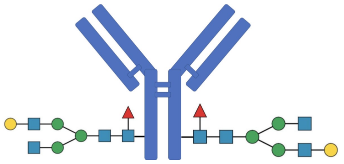

<Sample Information>

Sample Name	: 230313_53_G1a_G1b
Sample ID	: 230313_53_G1a_G1b
Data Filename	: 230313_53_G1a_G1b.lcd
Method Filename	: 2303_FcRIII_bunseki_AS_100min.lcm
Batch Filename	:
Vial #	: 53
Injection Volume	: 20 uL
Date Acquired	: 2023/03/25 16:10:07
Date Processed	: 2023/03/28 11:20:23

Sample Type	: Unknown
Acquired by	: System Administrator
Processed by	: System Administrator

<Chromatogram>

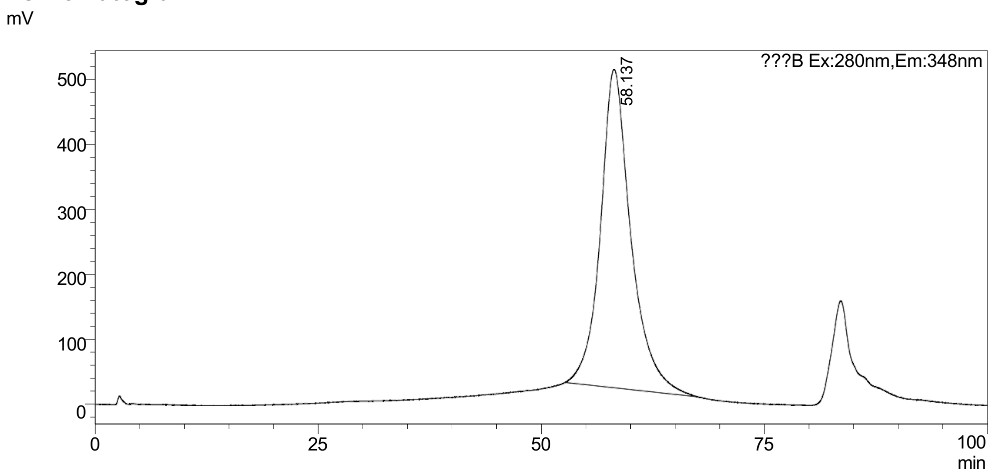

<Peak Table>

???B Ex:280nm,Em:348nm

Peak#	Ret. Time	Area	Height	Conc.	Unit	Mark	Name
1	58.137	116640661	490197	100.000		M	

7-9 [G1a-F/G0-F]

Analysis Report

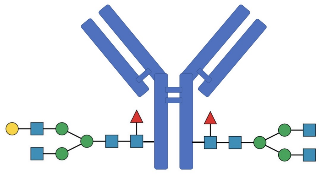

<Sample Information>

Sample Name : 230313_54_G1a_G0
Sample ID : 230313_54_G1a_G0
Data Filename : 230313_54_G1a_G0.lcd
Method Filename : 2303_FcRIII_bunseki_AS_100min.lcm
Batch Filename :
Vial # : 54
Injection Volume : 20 uL
Date Acquired : 2023/03/25 17:55:04
Date Processed : 2023/03/28 11:21:17

Sample Type : Unknown
Acquired by : System Administrator
Processed by : System Administrator

<Chromatogram>

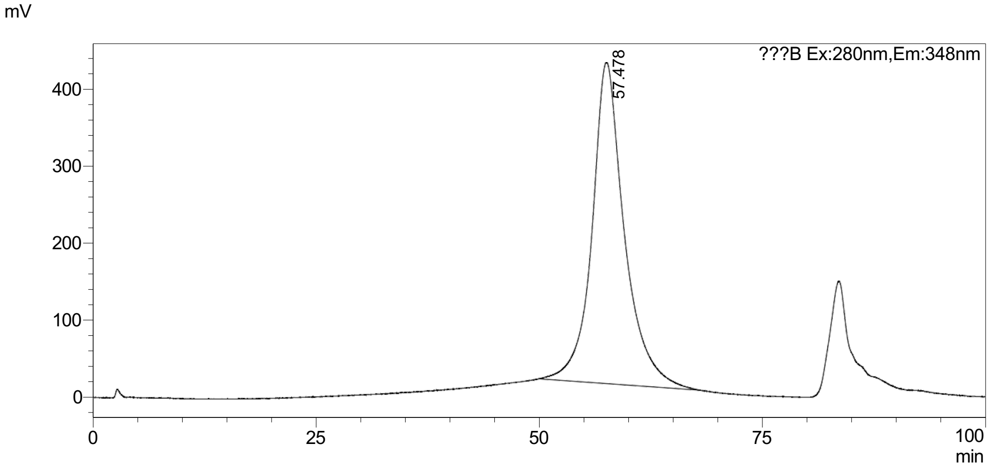

<Peak Table>

???B Ex:280nm,Em:348nm

Peak#	Ret. Time	Area	Height	Conc.	Unit	Mark	Name
1	57.478	105208812	416746	100.000		M	

7-10 [G1a-F/M3-F]

Analysis Report

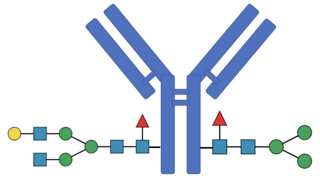

<Sample Information>

Sample Name	: 230313_55_G1a_M3		
Sample ID	: 230313_55_G1a_M3		
Data Filename	: 230313_55_G1a_M3.lcd		
Method Filename	: 2303_FcRIII_bunseki_AS_100min.lcm		
Batch Filename	:		
Vial #	: 55	Sample Type	: Unknown
Injection Volume	: 20 uL		
Date Acquired	: 2023/03/25 19:38:57	Acquired by	: System Administrator
Date Processed	: 2023/03/28 11:22:11	Processed by	: System Administrator

<Chromatogram>

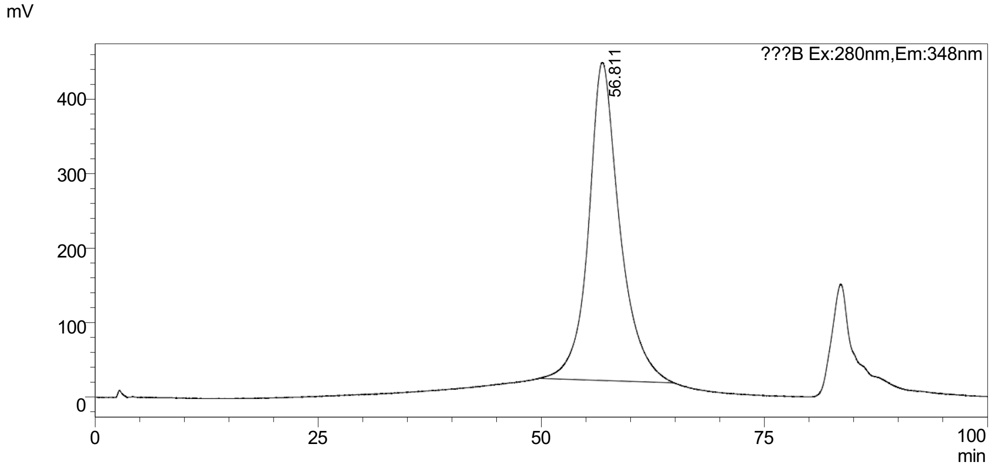

<Peak Table>

???B Ex:280nm,Em:348nm

Peak#	Ret. Time	Area	Height	Conc.	Unit	Mark	Name
1	56.811	104331759	426730	100.000		M	

7-11 [G1a-F/GlcNAc-F]

Analysis Report

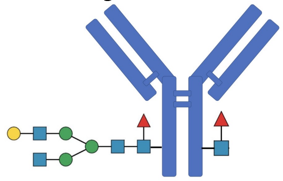

<Sample Information>

Sample Name	: 230313_56_G1a_GlcNAc		
Sample ID	: 230313_56_G1a_GlcNAc		
Data Filename	: 230313_56_G1a_GlcNAc.lcd		
Method Filename	: 2303_FcRIII_bunseki_AS_100min.lcm		
Batch Filename	: 230327.lcb		
Vial #	: 56	Sample Type	: Unknown
Injection Volume	: 20 uL		
Date Acquired	: 2023/03/27 21:21:43	Acquired by	: System Administrator
Date Processed	: 2023/03/28 11:23:07	Processed by	: System Administrator

<Chromatogram>

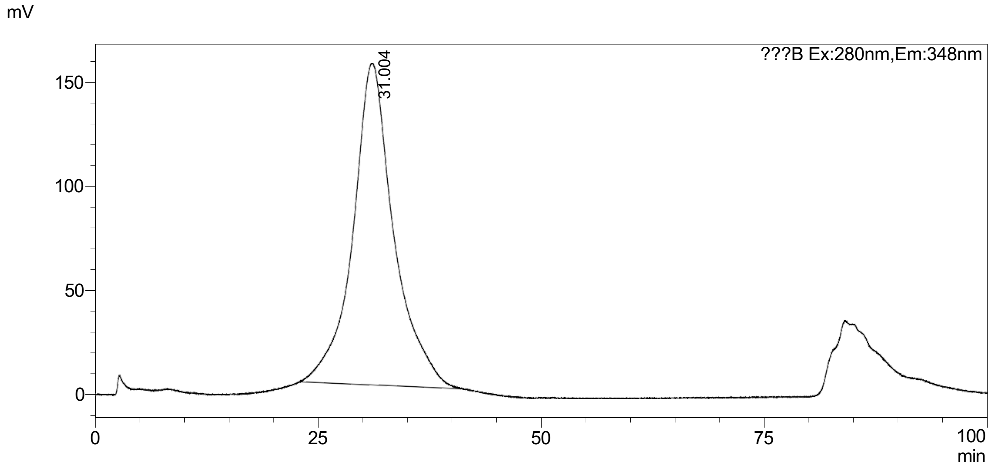

<Peak Table>

???B Ex:280nm,Em:348nm

Peak#	Ret. Time	Area	Height	Conc.	Unit	Mark	Name
1	31.004	49329167	154687	100.000		M	

8-8 [G1b-F/G1b-F]

Analysis Report

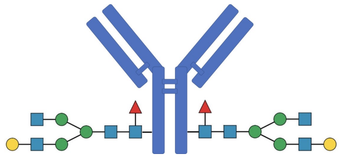

<Sample Information>

Sample Name	: 230313_57_G1b_G1b
Sample ID	: 230313_57_G1b_G1b
Data Filename	: 230313_57_G1b_G1b.lcd
Method Filename	: 2303_FcRIII_bunseki_AS_100min.lcm
Batch Filename	: 230325.lcb
Vial #	: 57
Injection Volume	: 20 uL
Date Acquired	: 2023/03/25 21:22:28
Date Processed	: 2023/03/28 11:24:01
Sample Type	: Unknown
Acquired by	: System Administrator
Processed by	: System Administrator

<Chromatogram>

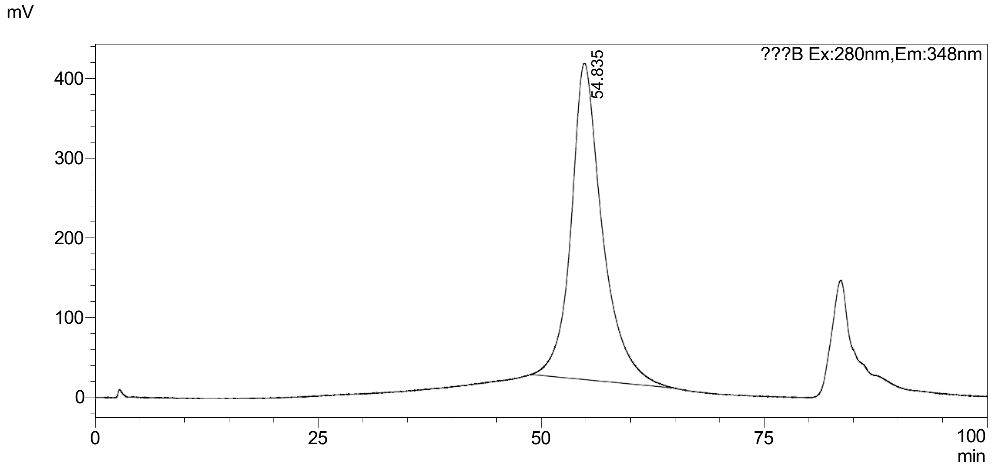

<Peak Table>

???B Ex:280nm,Em:348nm

Peak#	Ret. Time	Area	Height	Conc.	Unit	Mark	Name
1	54.835	97292942	397193	100.000		M	

8-9 [G1b-F/G0-F]

Analysis Report

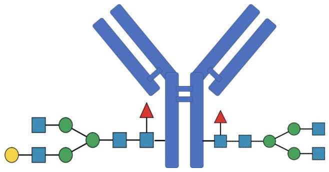

<Sample Information>

Sample Name : 230630_58_G1b_G0_re
Sample ID : 230630_58_G1b_G0_re
Data Filename : 230630_58_G1b_G0_re001.lcd
Method Filename : 2303_FcRIII_bunseki_AS_100min.lcm
Batch Filename :
Vial # : 1
Sample Type : Unknown
Injection Volume : 20 uL
Date Acquired : 2023/06/30 17:20:17
Acquired by : System Administrator
Date Processed : 2023/06/30 19:04:10
Processed by : System Administrator

<Chromatogram>

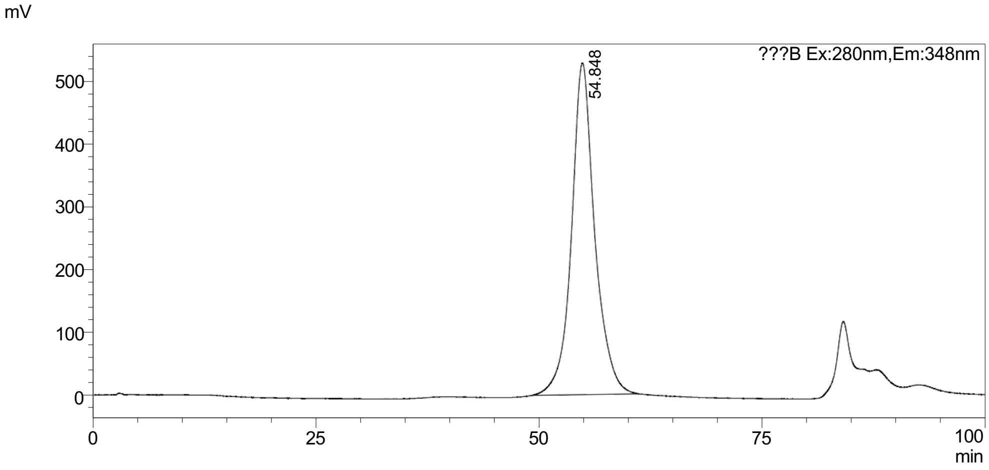

<Peak Table>

???B Ex:280nm,Em:348nm

Peak#	Ret. Time	Area	Height	Conc.	Unit	Mark	Name
1	54.848	95916985	529122	100.000		M	

8-10 [G1b-F/M3-F]

Analysis Report

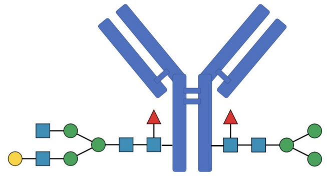

<Sample Information>

Sample Name	: 230313_59_G1b_M3
Sample ID	: 230313_59_G1b_M3
Data Filename	: 230313_59_G1b_M3.lcd
Method Filename	: 2303_FcRIII_bunseki_AS_100min.lcm
Batch Filename	:
Vial #	: 59
Injection Volume	: 20 uL
Date Acquired	: 2023/03/26 10:57:34
Date Processed	: 2023/03/28 11:26:30
Sample Type	: Unknown
Acquired by	: System Administrator
Processed by	: System Administrator

<Chromatogram>

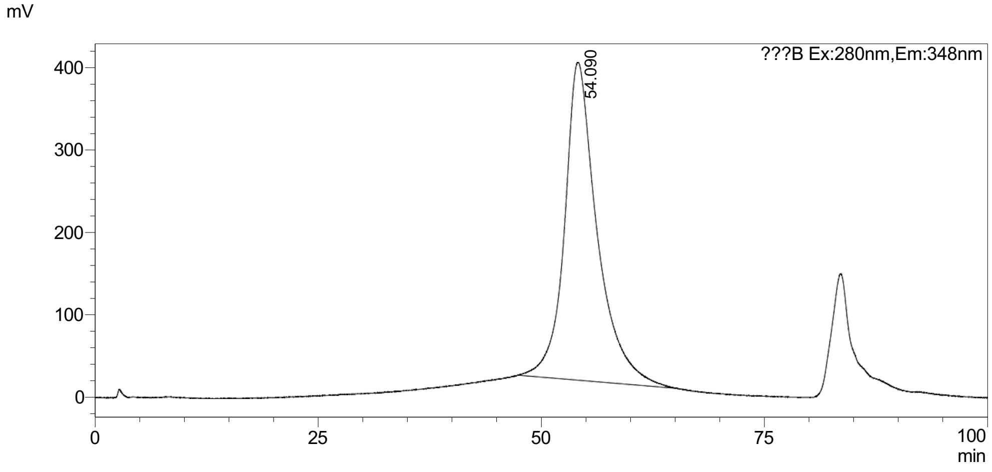

<Peak Table>

???B Ex:280nm,Em:348nm

Peak#	Ret. Time	Area	Height	Conc.	Unit	Mark	Name
1	54.090	98375752	385364	100.000		M	

8-11 [G1b-F/GlcNAc-F]

Analysis Report

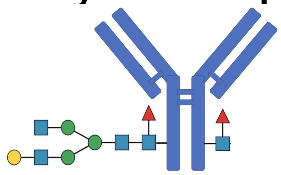

<Sample Information>

Sample Name	: 230313_60_G1b_GlcNAc		
Sample ID	: 230313_60_G1b_GlcNAc		
Data Filename	: 230313_60_G1b_GlcNAc.lcd		
Method Filename	: 2303_FcRIII_bunseki_AS_100min_.lcm		
Batch Filename	: 230327.lcb		
Vial #	: 0	Sample Type	: Unknown
Injection Volume	: 20 uL		
Date Acquired	: 2023/03/27 23:04:49	Acquired by	: System Administrator
Date Processed	: 2023/03/28 11:27:31	Processed by	: System Administrator

<Chromatogram>

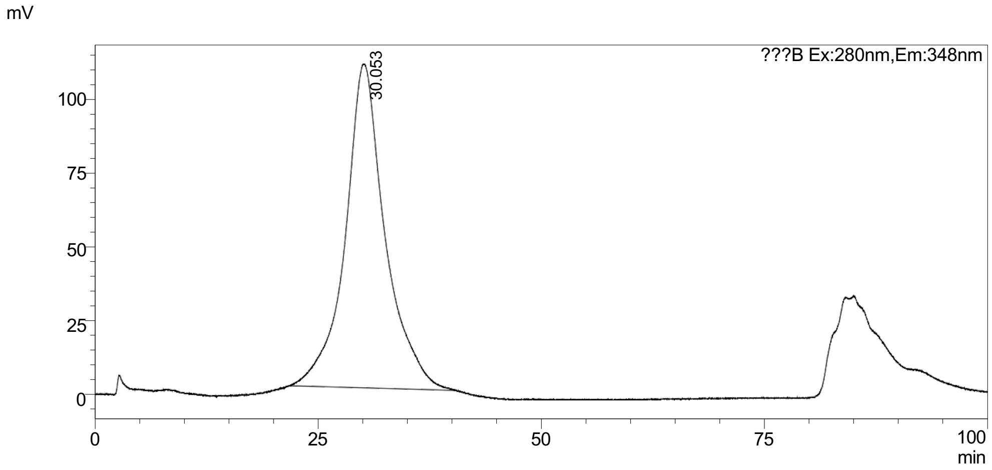

<Peak Table>

???B Ex:280nm,Em:348nm

Peak#	Ret. Time	Area	Height	Conc.	Unit	Mark	Name
1	30.053	34109644	109909	100.000		M	

9-9 [G0-F/G0-F]

Analysis Report

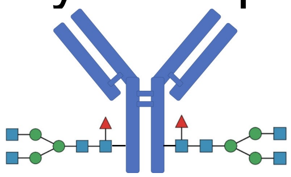

<Sample Information>

Sample Name	: 230313_61_G0_G0		
Sample ID	: 230313_61_G0_G0		
Data Filename	: 230313_61_G0_G0.lcd		
Method Filename	: 2303_FcRIII_bunseki_AS_100min.lcm		
Batch Filename	:		
Vial #	: 1	Sample Type	: Unknown
Injection Volume	: 20 uL		
Date Acquired	: 2023/03/26 12:44:40	Acquired by	: System Administrator
Date Processed	: 2023/03/28 11:28:55	Processed by	: System Administrator

<Chromatogram>

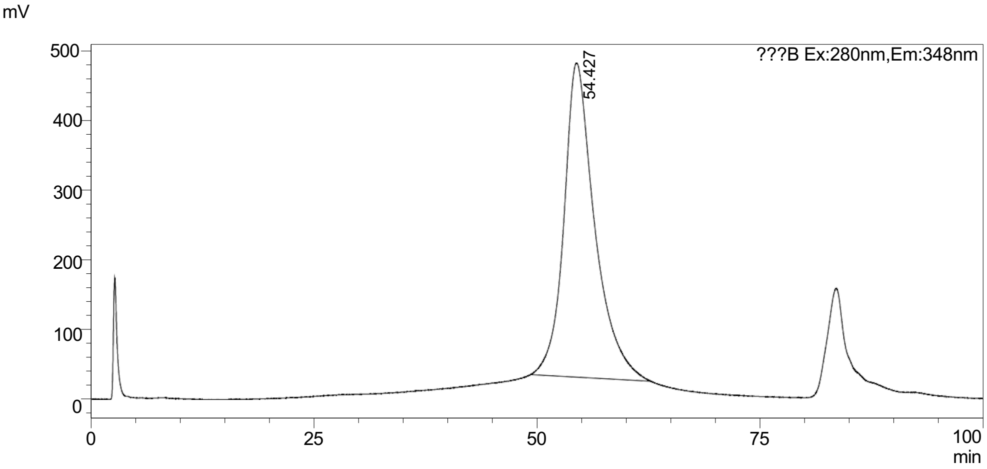

<Peak Table>

???B Ex:280nm,Em:348nm

Peak#	Ret. Time	Area	Height	Conc.	Unit	Mark	Name
1	54.427	108621517	450916	100.000		M	

9-10 [G0-F/M3-F]

Analysis Report

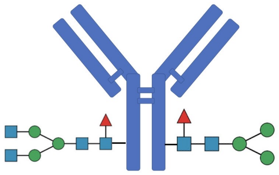

<Sample Information>

Sample Name	: 230313_62_G0_M3		
Sample ID	: 230313_62_G0_M3		
Data Filename	: 230313_62_G0_M3.lcd		
Method Filename	: 2303_FcRIII_bunseki_AS_100min.lcm		
Batch Filename	:		
Vial #	: 2	Sample Type	: Unknown
Injection Volume	: 20 uL		
Date Acquired	: 2023/03/26 14:28:35	Acquired by	: System Administrator
Date Processed	: 2023/03/28 11:29:51	Processed by	: System Administrator

<Chromatogram>

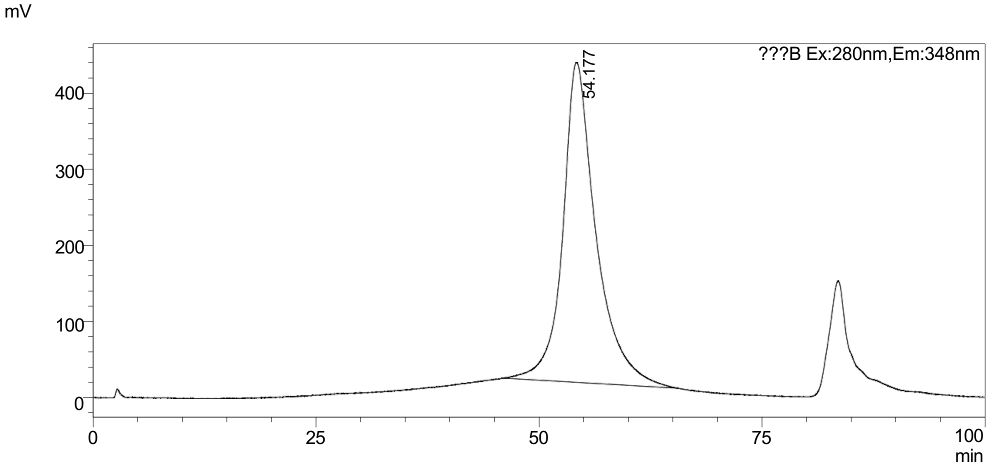

<Peak Table>

???B Ex:280nm,Em:348nm

Peak#	Ret. Time	Area	Height	Conc.	Unit	Mark	Name
1	54.177	109240334	420101	100.000		M	

9-11 [G0-F/GlcNAc-F]

Analysis Report

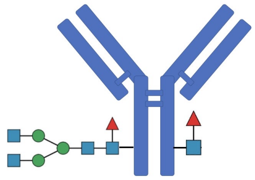

<Sample Information>

Sample Name : 230313_63_G0_GlcNAc
Sample ID : 230313_63_G0_GlcNAc
Data Filename : 230313_63_G0_GlcNAc.lcd
Method Filename : 2303_FcRIII_bunseki_AS_100min_.lcm
Batch Filename : 230327.lcb
Vial # : 3
Injection Volume : 20 uL
Date Acquired : 2023/03/28 0:47:52
Date Processed : 2023/03/28 11:31:30

Sample Type : Unknown
Acquired by : System Administrator
Processed by : System Administrator

<Chromatogram>

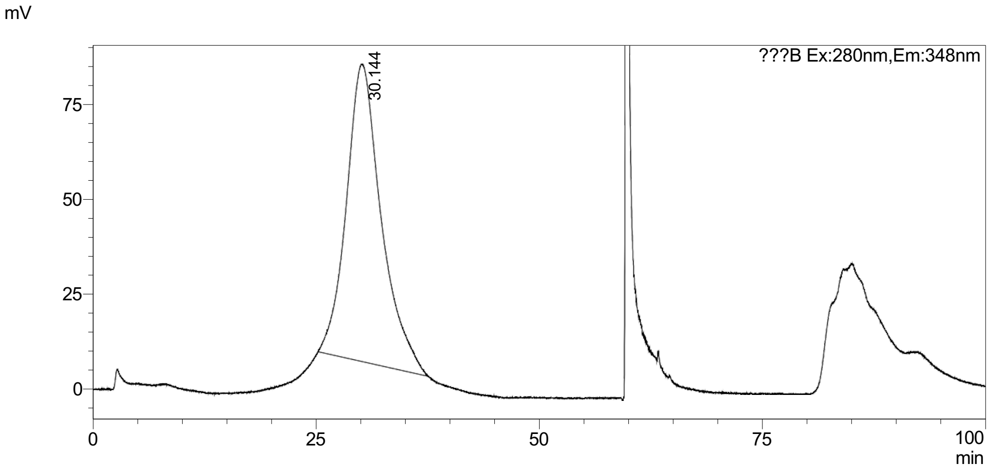

<Peak Table>

???B Ex:280nm,Em:348nm

Peak#	Ret. Time	Area	Height	Conc.	Unit	Mark	Name
1	30.144	21316815	78319	100.000		M	

10-10 [M3-F/M3-F]

Analysis Report

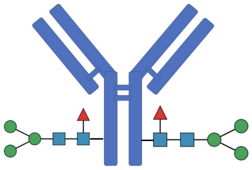

<Sample Information>

Sample Name : 230313_64_M3_M3
Sample ID : 230313_64_M3_M3
Data Filename : 230313_64_M3_M3.lcd
Method Filename : 2303_FcRIII_bunseki_AS_100min.lcm
Batch Filename :
Vial # : 4
Sample Type : Unknown
Injection Volume : 20 uL
Date Acquired : 2023/03/26 16:12:23
Acquired by : System Administrator
Date Processed : 2023/05/15 19:35:21
Processed by : System Administrator

<Chromatogram>

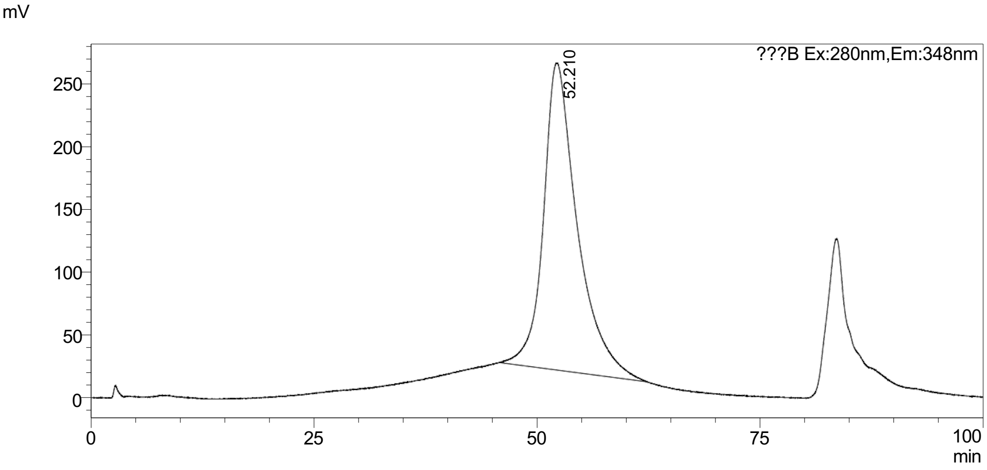

<Peak Table>

???B Ex:280nm,Em:348nm

Peak#	Ret. Time	Area	Height	Conc.	Unit	Mark	Name
1	52.210	64770929	244883	100.000		M	

10-11 [M3-F/GlcNAc-F]

Analysis Report

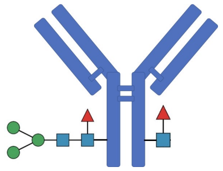

<Sample Information>

Sample Name : 230313_65_M3_GlcNAc
Sample ID : 230313_65_M3_GlcNAc
Data Filename : 230313_65_M3_GlcNAc.lcd
Method Filename : 2303_FcRIII_bunseki_AS_100min.lcm
Batch Filename :
Vial # : 5
Injection Volume : 20 uL
Date Acquired : 2023/03/28 8:47:33
Date Processed : 2023/03/28 11:32:36

Sample Type : Unknown
Acquired by : System Administrator
Processed by : System Administrator

<Chromatogram>

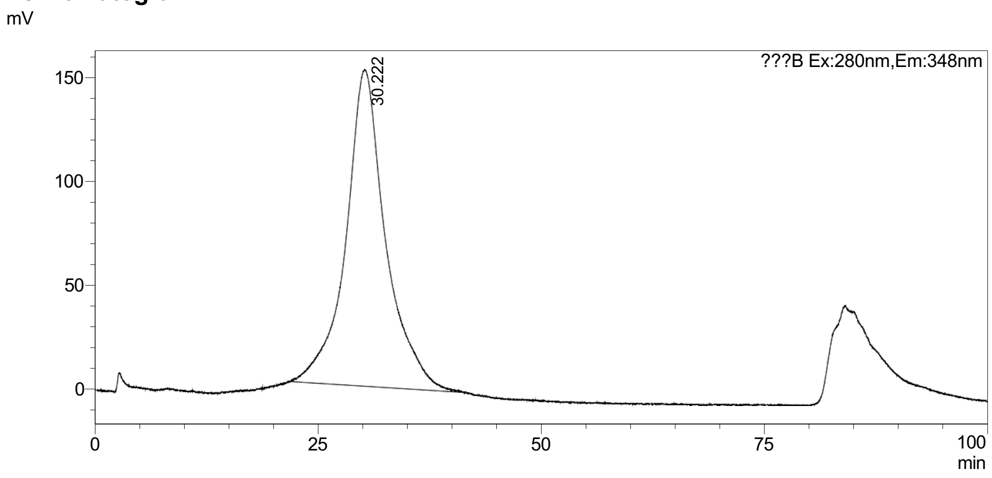

<Peak Table>

???B Ex:280nm,Em:348nm

Peak#	Ret. Time	Area	Height	Conc.	Unit	Mark	Name
1	30.222	47278468	152519	100.000		M	

11-11 [GlcNAc-F/GlcNAc-F]

Analysis Report

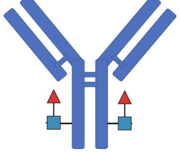

<Sample Information>

Sample Name	: 230313_66_GlcNAc_GlcNAc		
Sample ID	: 230313_66_GlcNAc_GlcNAc		
Data Filename	: 230313_66_GlcNAc_GlcNAc.lcd		
Method Filename	: 2303_FcRIII_bunseki_AS_100min.lcm		
Batch Filename	: 230325.lcb		
Vial #	: 6	Sample Type	: Unknown
Injection Volume	: 20 uL		
Date Acquired	: 2023/03/25 23:05:33	Acquired by	: System Administrator
Date Processed	: 2023/03/28 11:34:00	Processed by	: System Administrator

<Chromatogram>

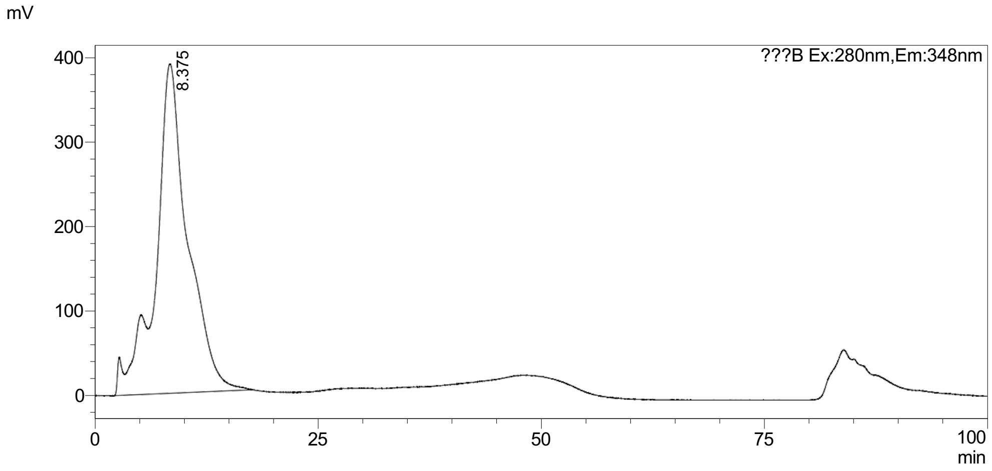

<Peak Table>

???B Ex:280nm,Em:348nm

Peak#	Ret. Time	Area	Height	Conc.	Unit	Mark	Name
1	8.375	97036478	389910	100.000		M	

[Trastuzumab (Herceptin®)]

Analysis Report

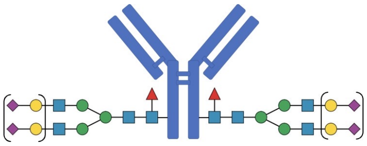

<Sample Information>

Sample Name	: 230610_Herceptin		
Sample ID	: 230610_Herceptin		
Data Filename	: 230610_Herceptin1.lcd		
Method Filename	: 2303_FcRIII_bunseki_AS_100min.lcm		
Batch Filename	:		
Vial #	: 10	Sample Type	: Unknown
Injection Volume	: 20 uL		
Date Acquired	: 2023/06/10 12:29:02	Acquired by	: System Administrator
Date Processed	: 2023/06/30 8:54:37	Processed by	: System Administrator

<Chromatogram>

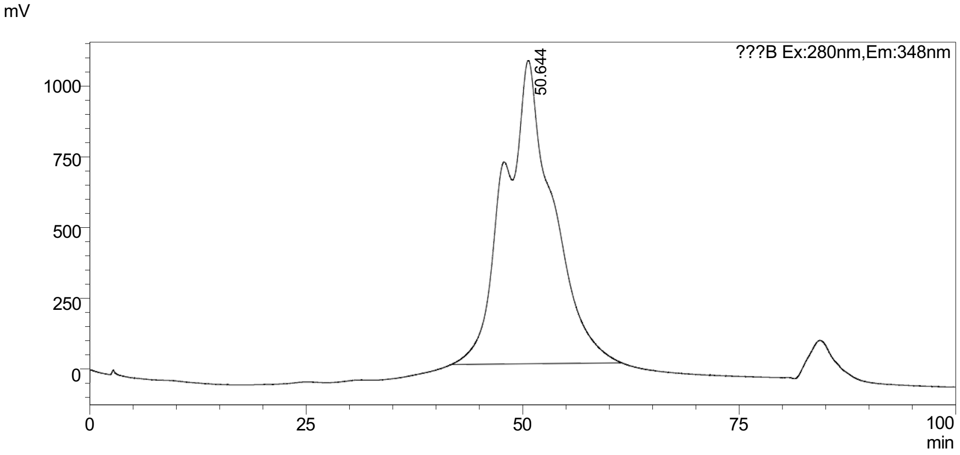

<Peak Table>

???B Ex:280nm,Em:348nm

Peak#	Ret. Time	Area	Height	Conc.	Unit	Mark	Name
1	50.644	421794150	1071129	100.000		M	

Figure S5.
Intact MS analysis of trastuzumab with homogeneous glycan.

1-1 [SG-F/SG-F]

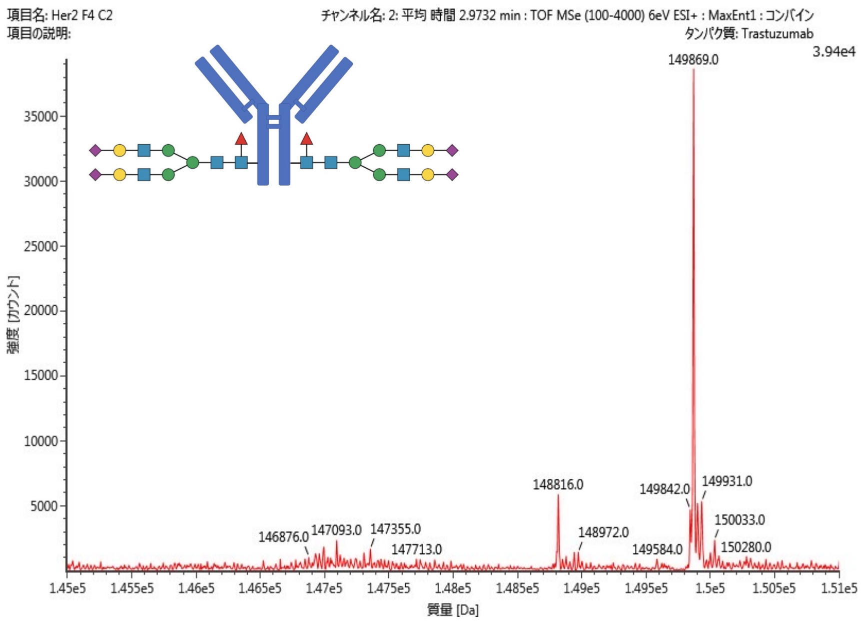

1-1 [SG-F/SG-F]

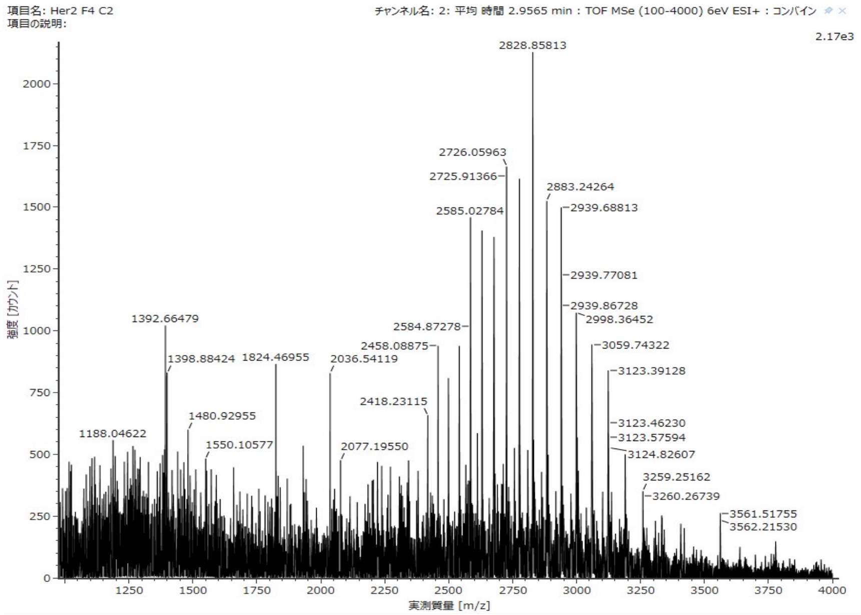

1-2 [SG-F/A1a-F]

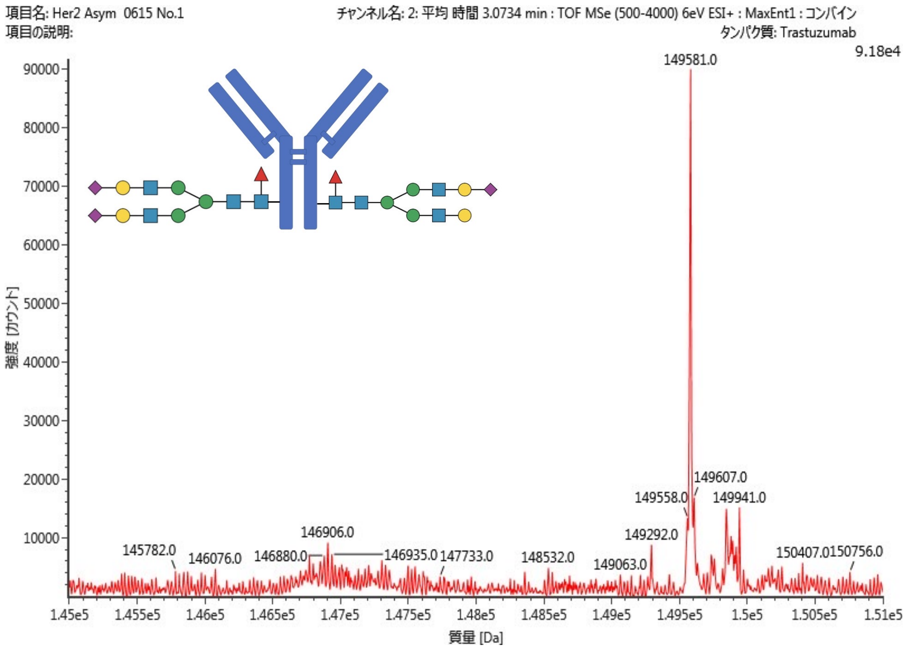

1-2 [SG-F/A1a-F]

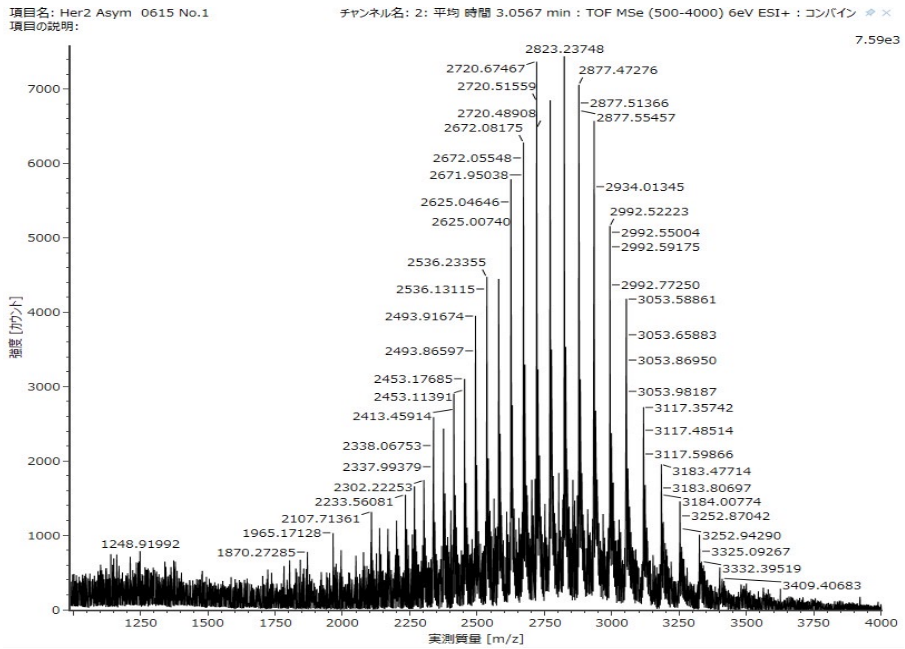

1-3 [SG-F/A1b-F]

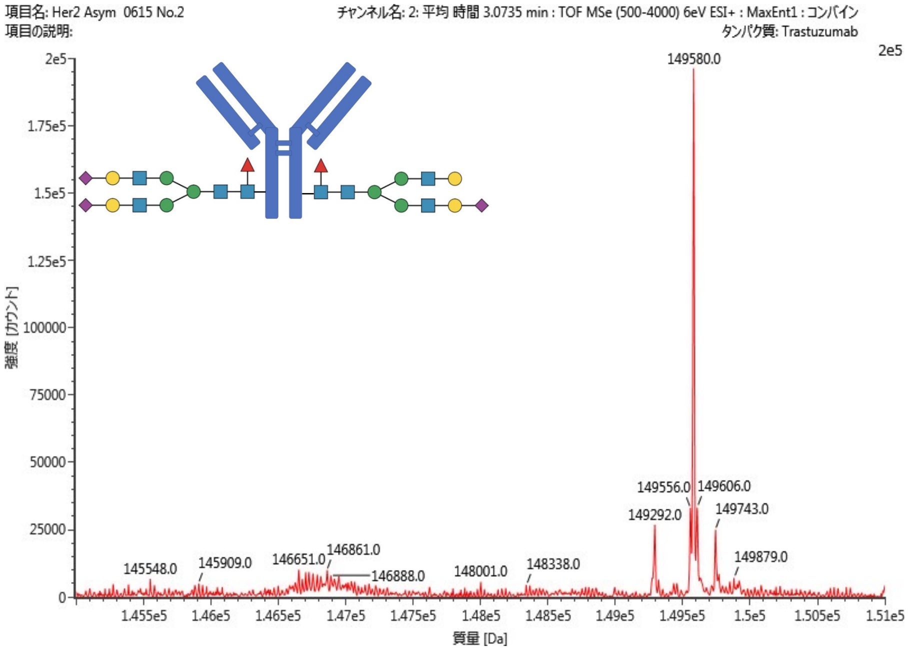

1-3 [SG-F/A1b-F]

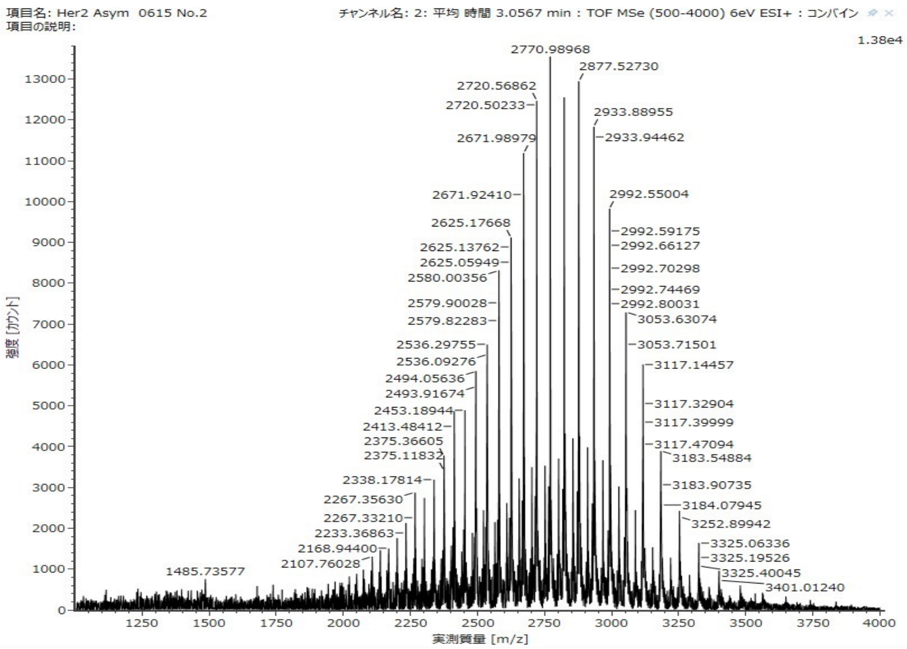

1-4 [SG-F/A1a-Gal-F]

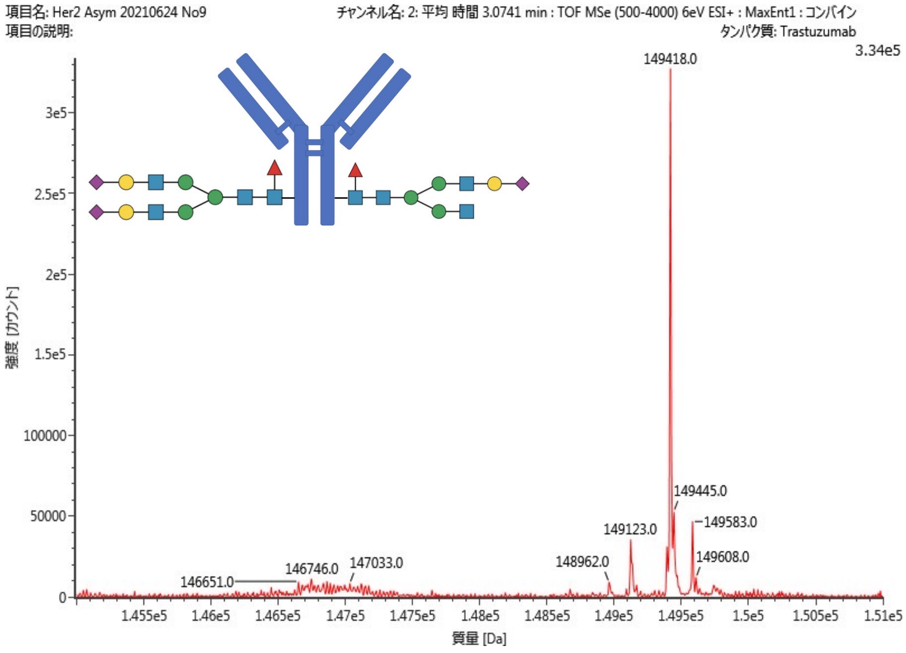

1-4 [SG-F/A1a-Gal-F]

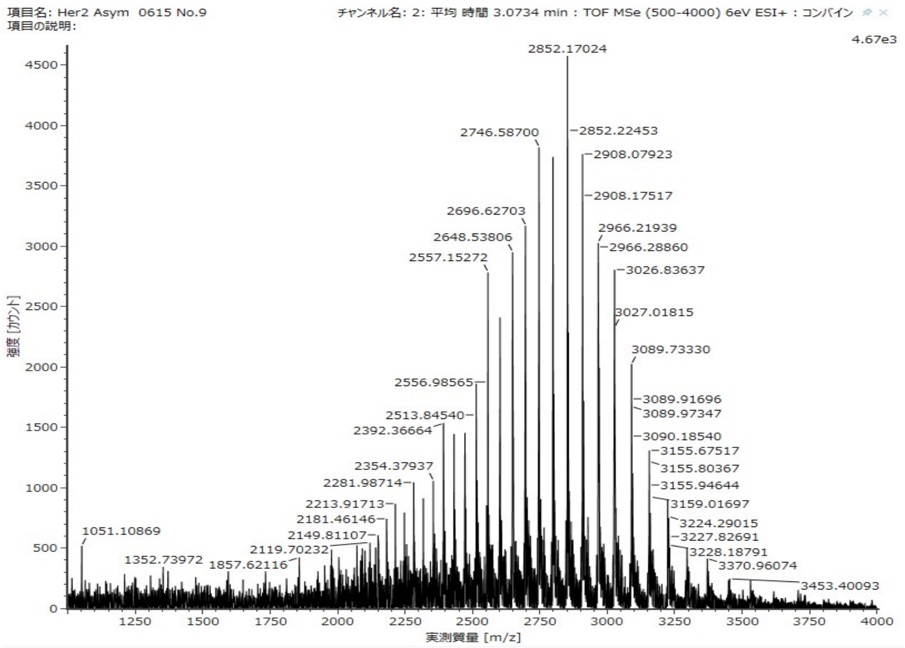

1-5 [SG-F/A1b-Gal-F]

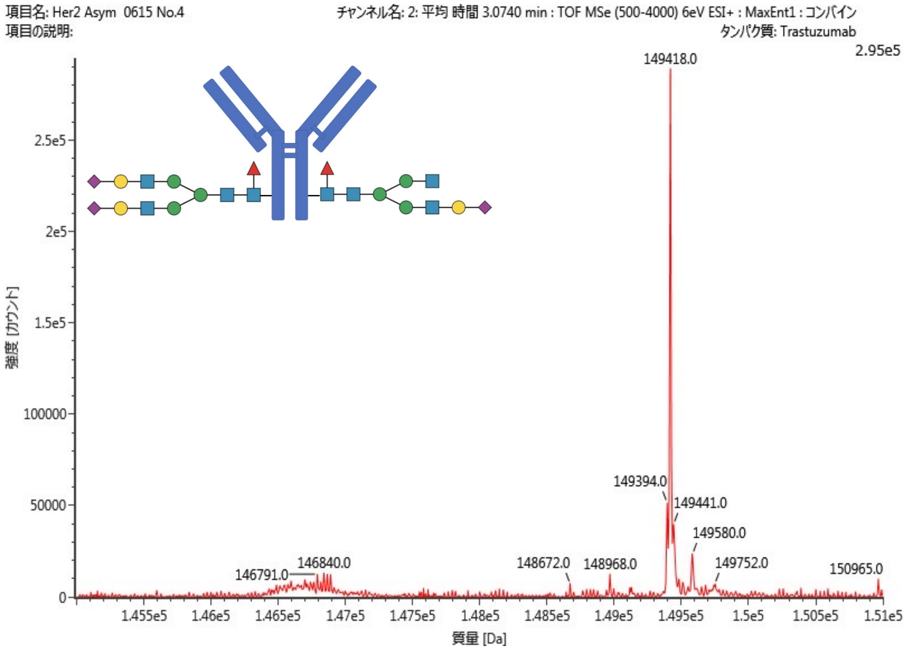

1-5 [SG-F/A1b-Gal-F]

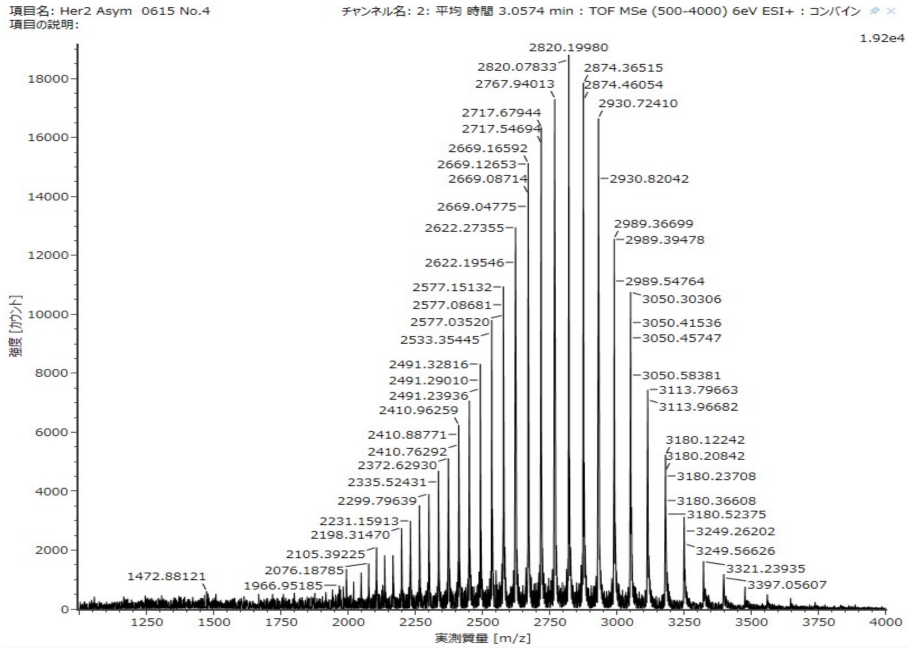

1-6 [SG-F/G2-F]

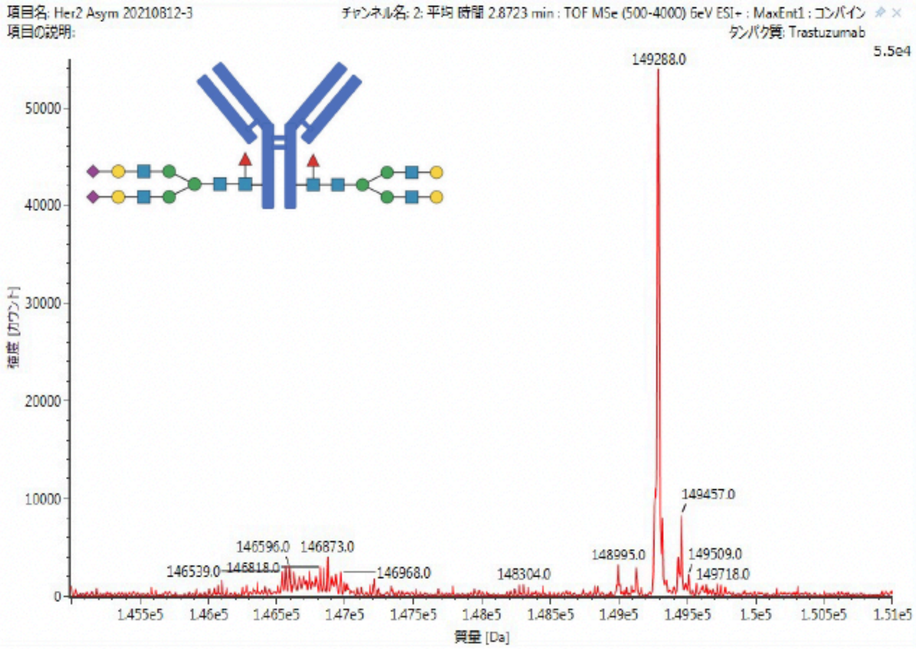

1-6 [SG-F/G2-F]

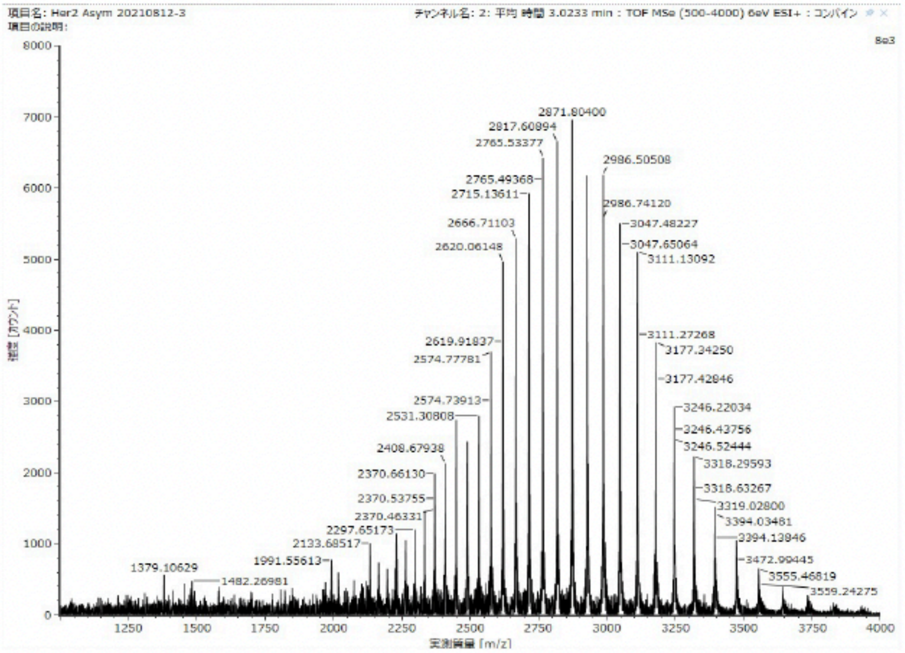

1-7 [SG-F/G1a-F]

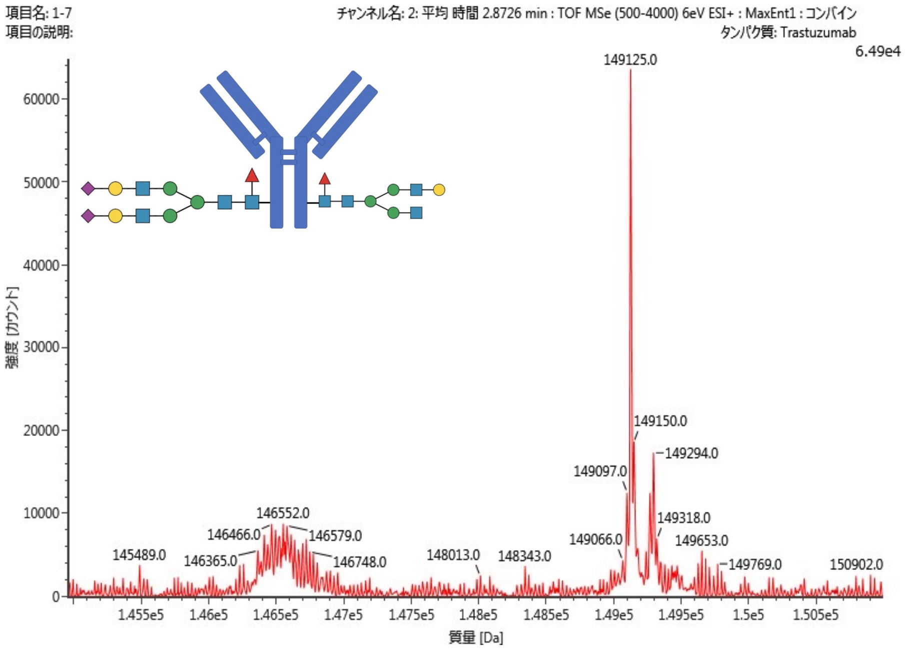

1-7 [SG-F/G1a-F]

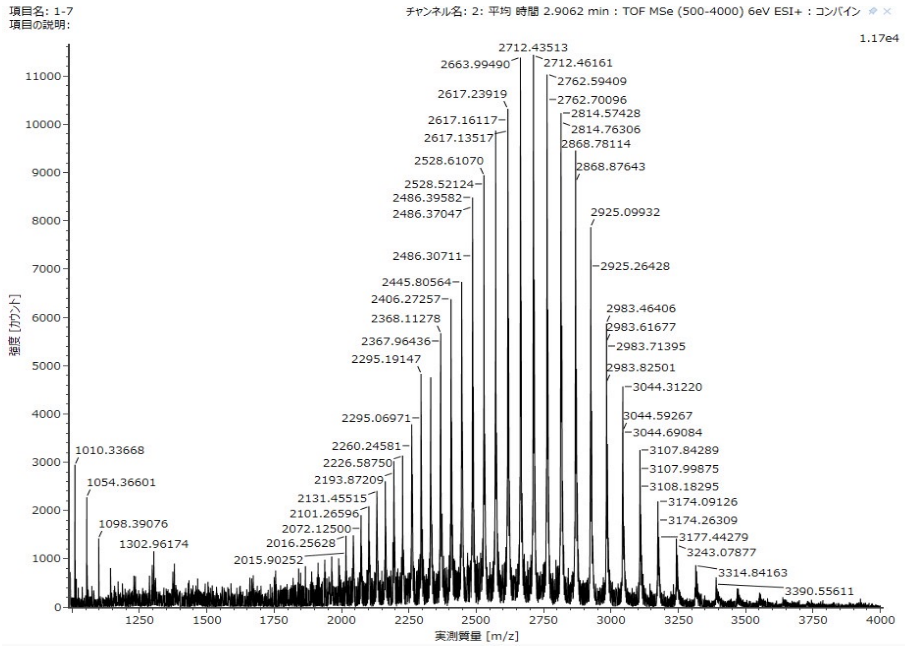

1-8 [SG-F/G1b-F]

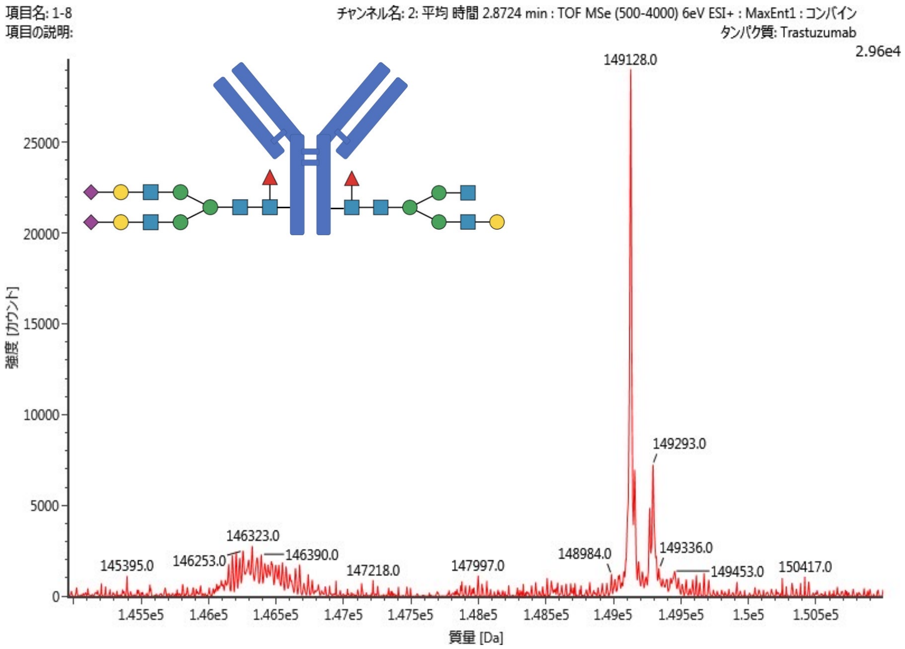

1-8 [SG-F/G1b-F]

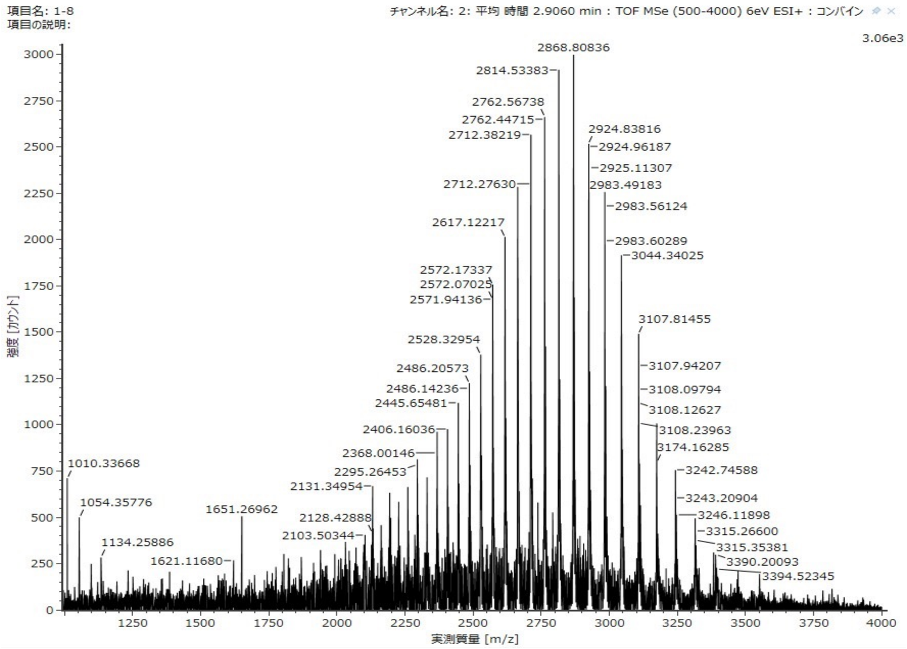

1-9 [SG-F/G0-F]

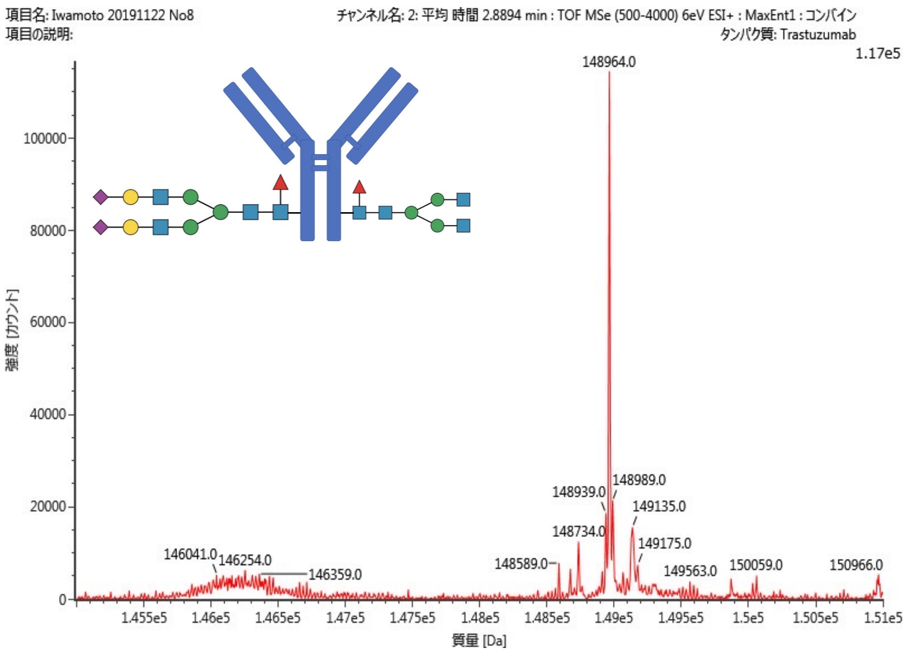

1-9 [SG-F/G0-F]

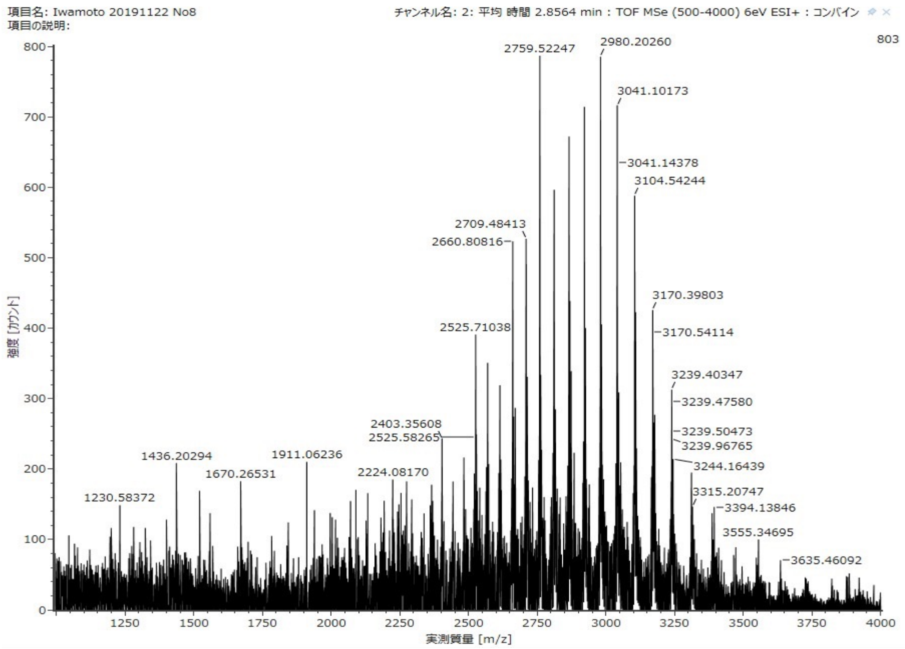

1-10 [SG-F/M3-F]

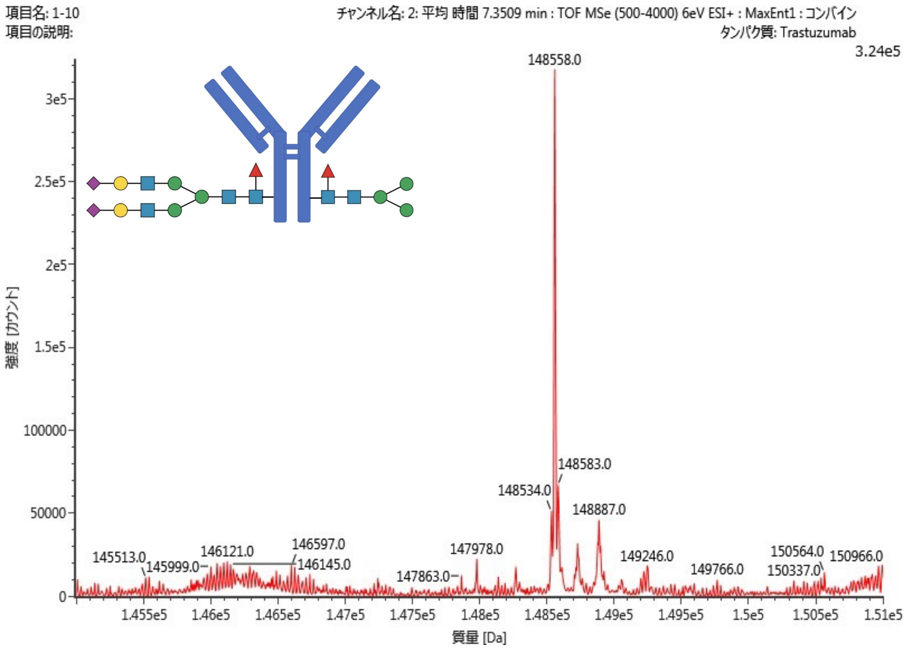

1-10 [SG-F/M3-F]

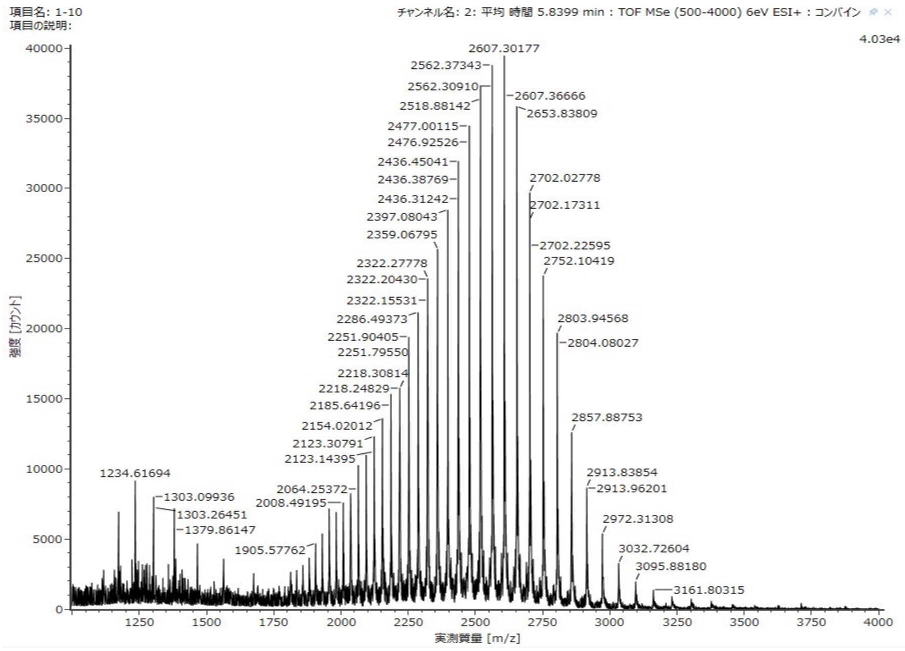

1-11 [SG-F/GlcNAc-F]

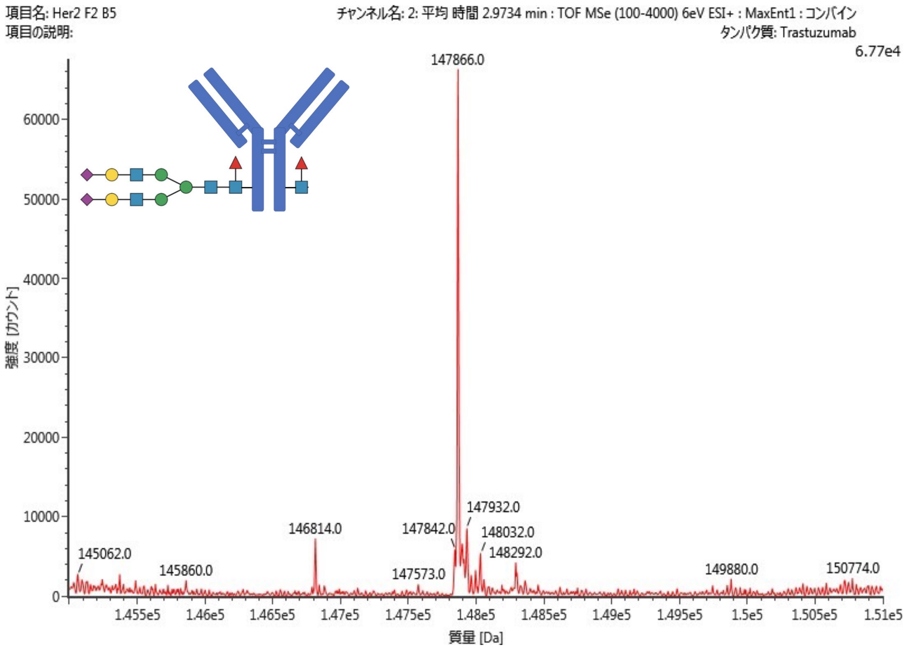

1-11 [SG-F/GlcNAc-F]

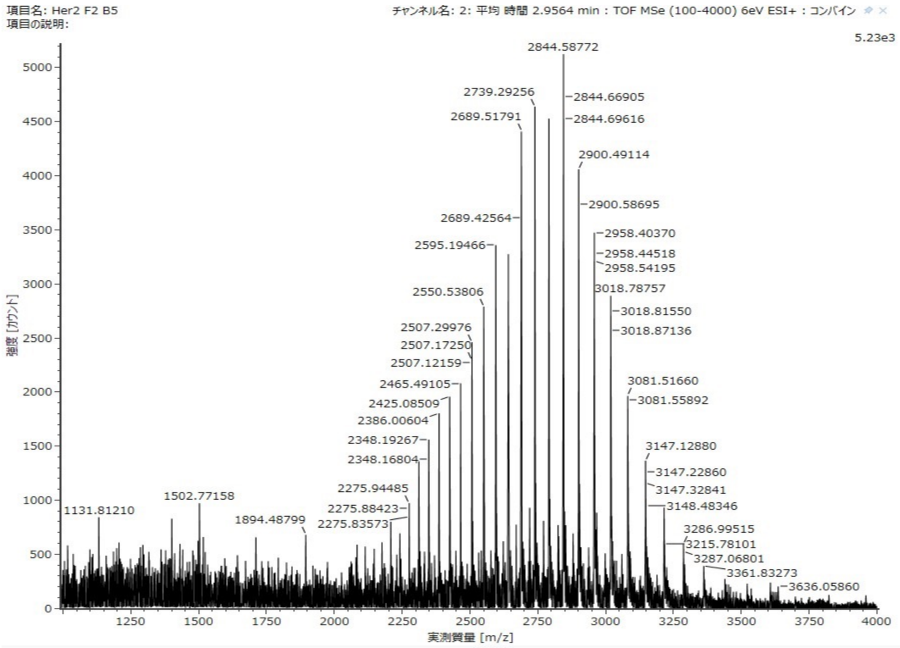

2-2 [A1a-F/A1a-F]

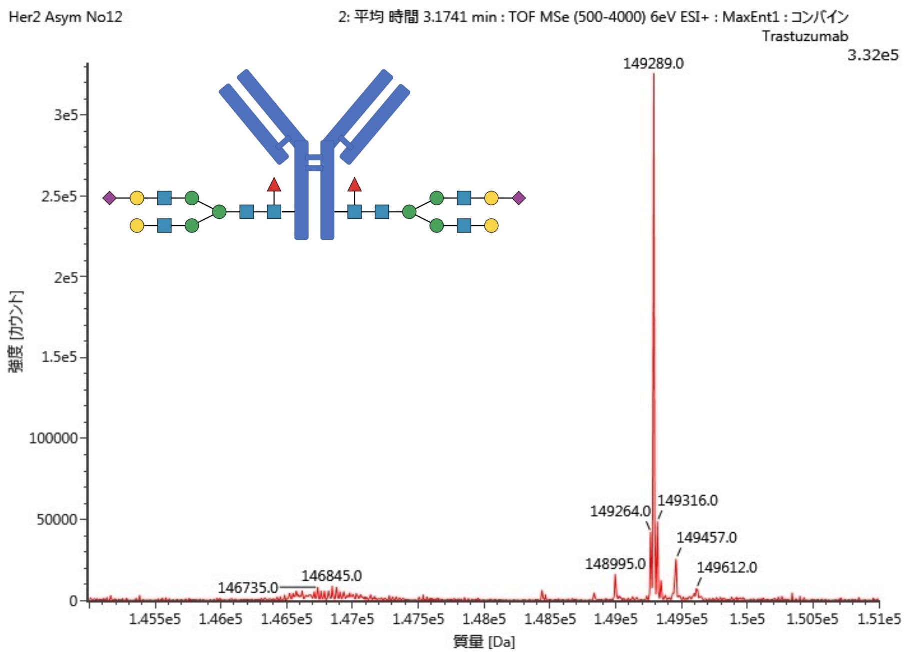

2-2 [A1a-F/A1a-F]

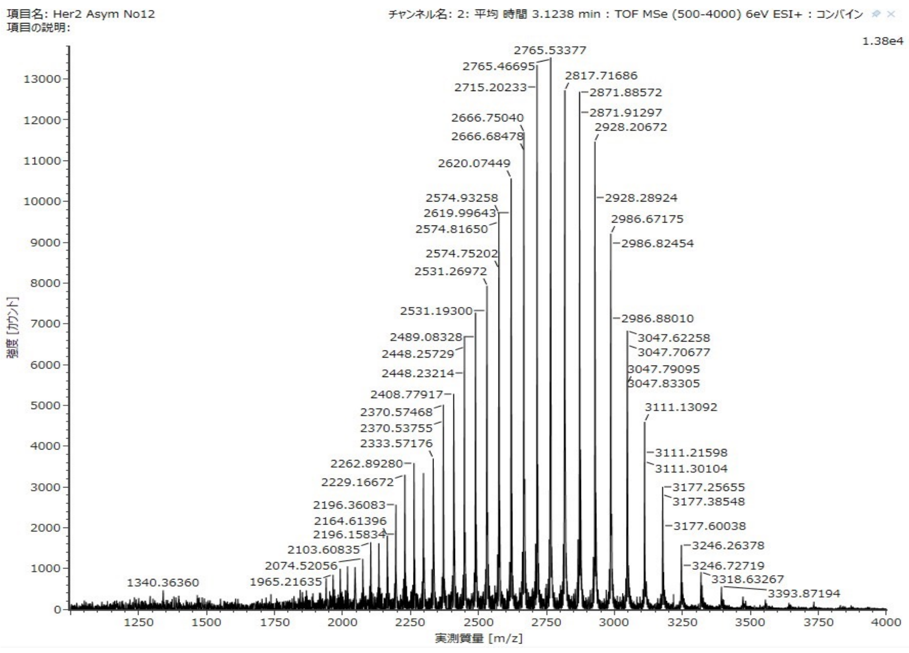

2-3 [A1a-F/A1b-F]

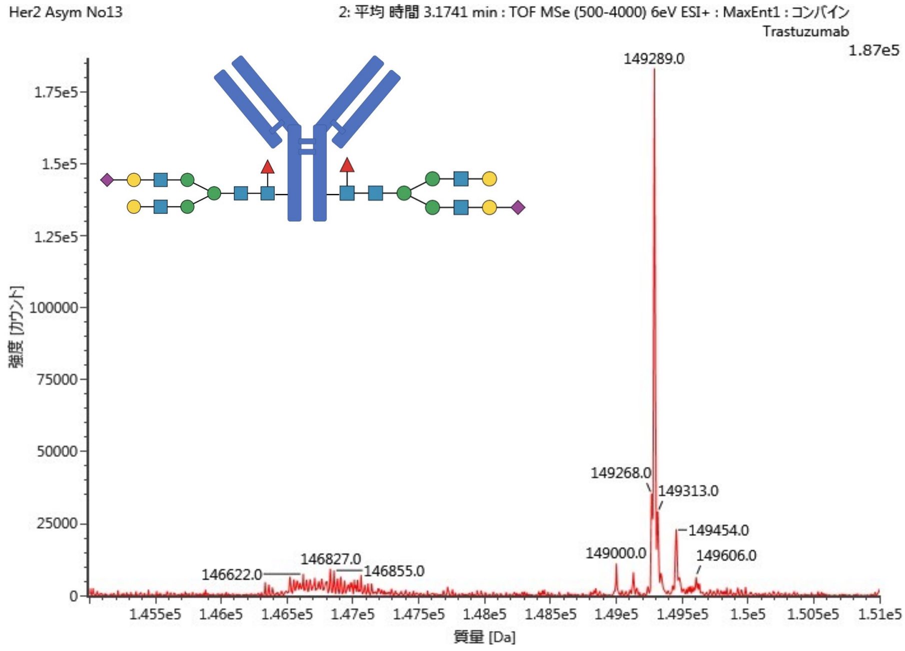

2-3 [A1a-F/A1b-F]

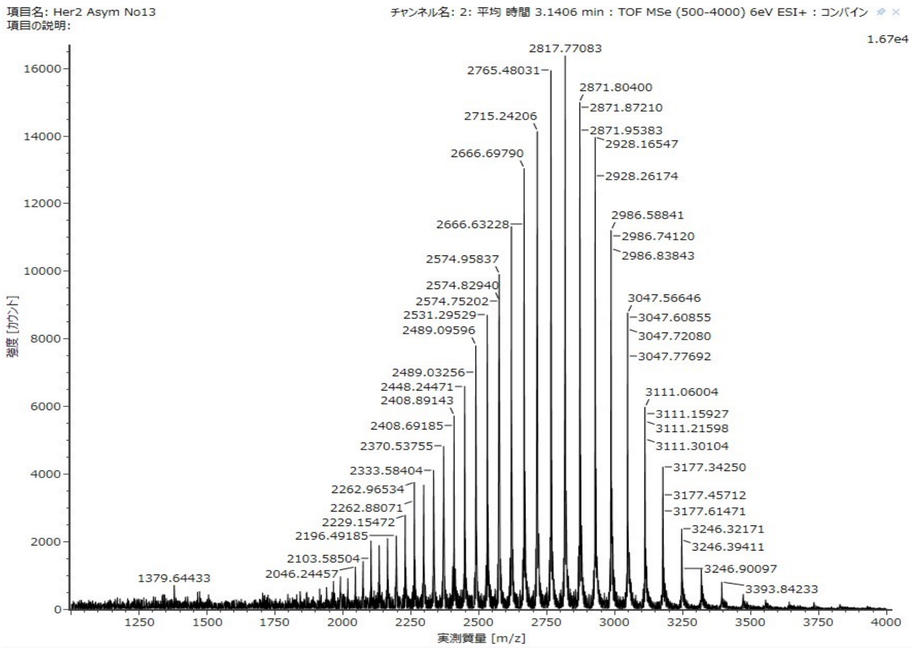

2-4 [A1a-F/A1a-Gal-F]

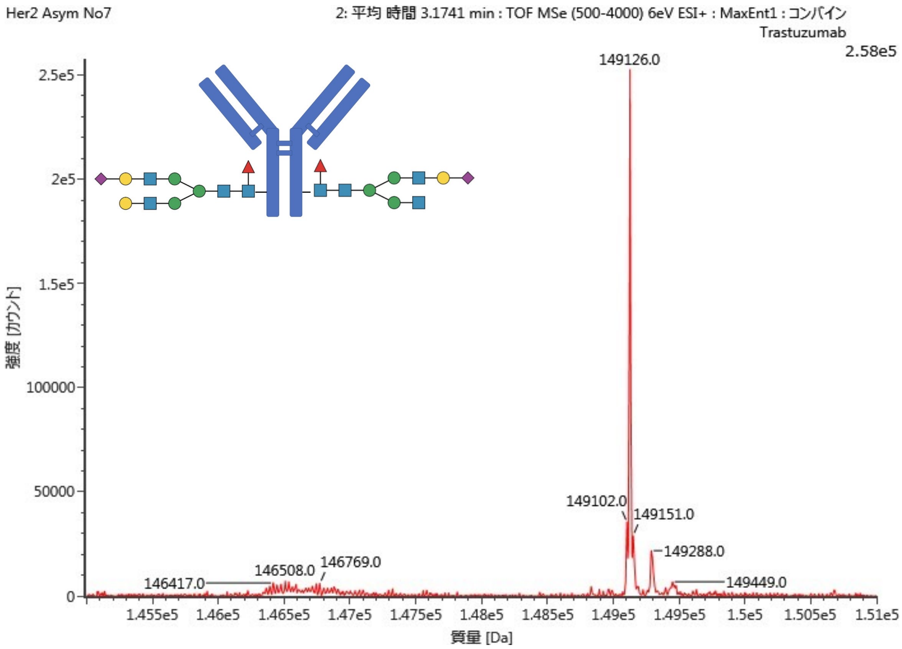

2-4 [A1a-F/A1a-Gal-F]

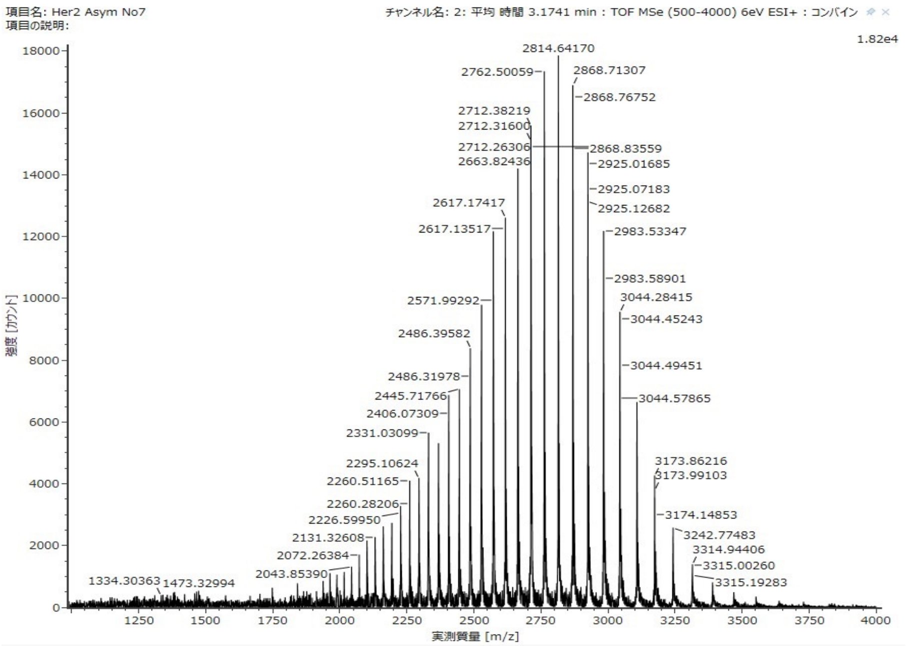

2-5 [A1a-F/A1b-Gal-F]

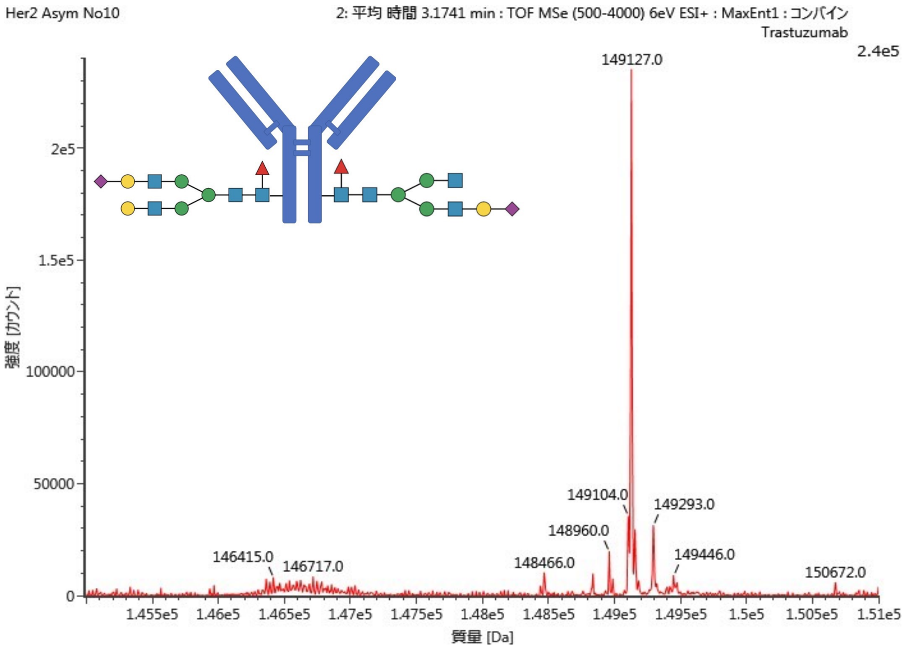

2-5 [A1a-F/A1b-Gal-F]

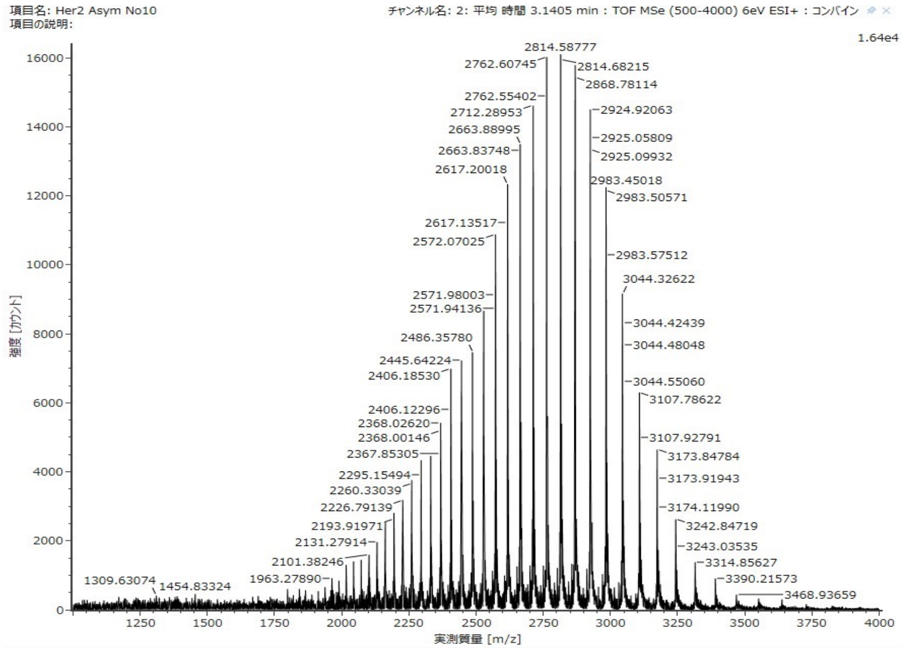

2-6 [A1a-F/G2-F]

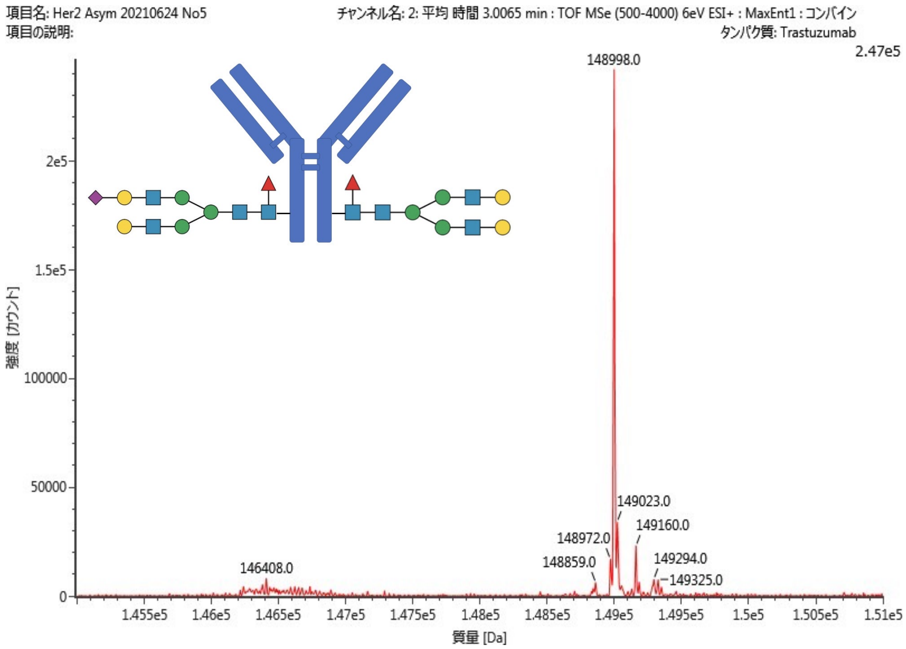

2-6 [A1a-F/G2-F]

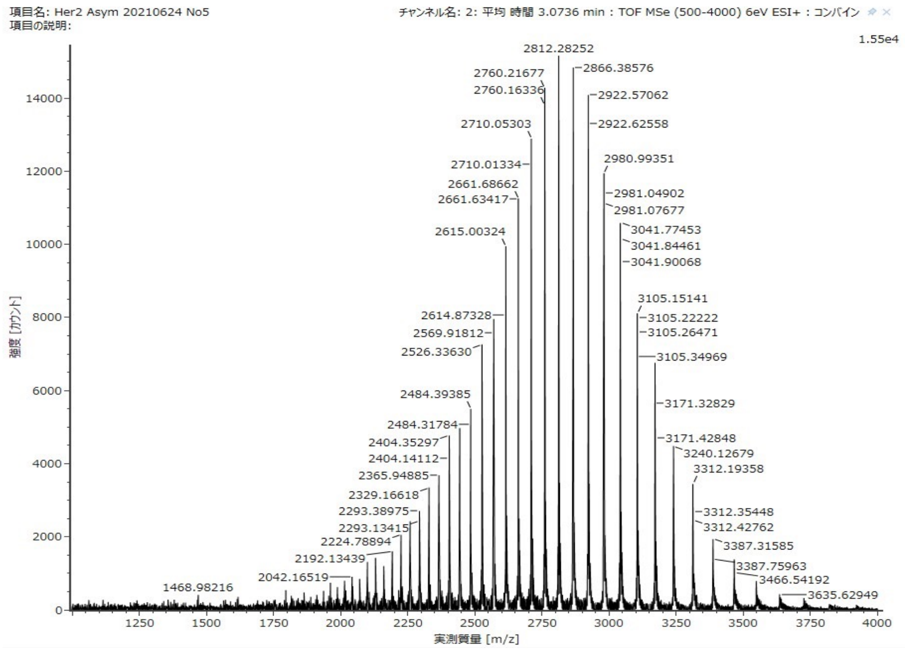

2-7 [A1a-F/G1a-F]

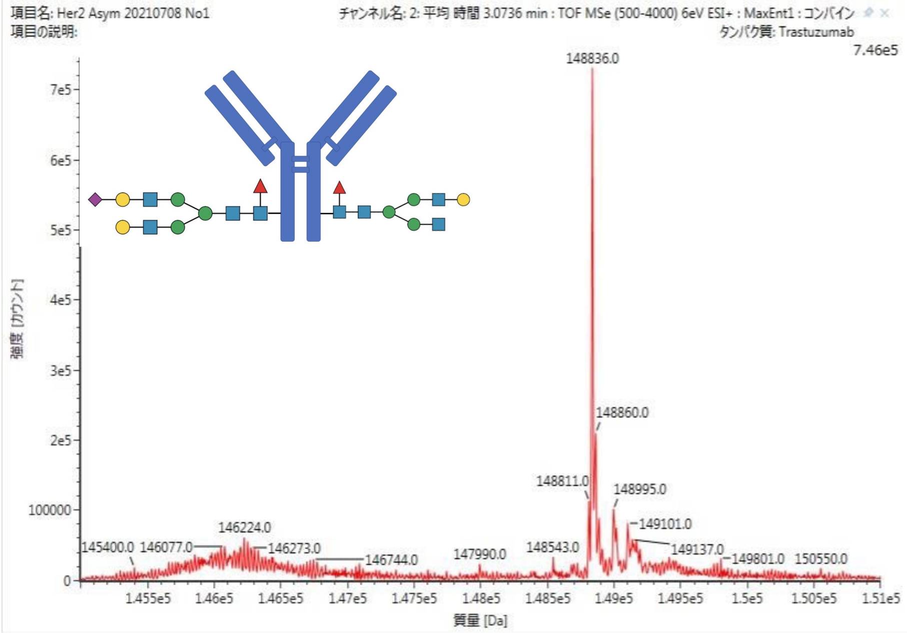

2-7 [A1a-F/G1a-F]

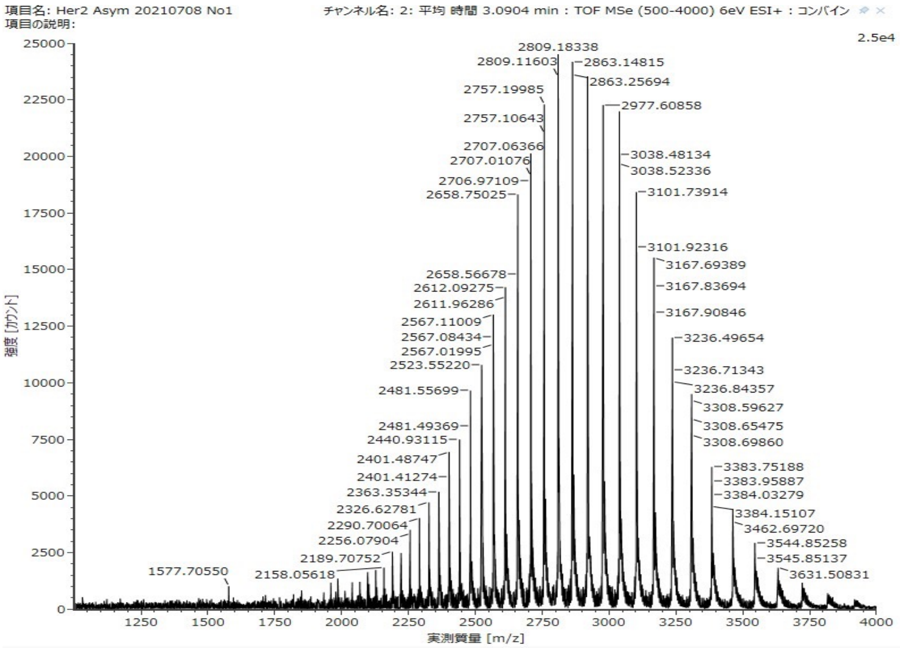

2-8 [A1a-F/G1b-F]

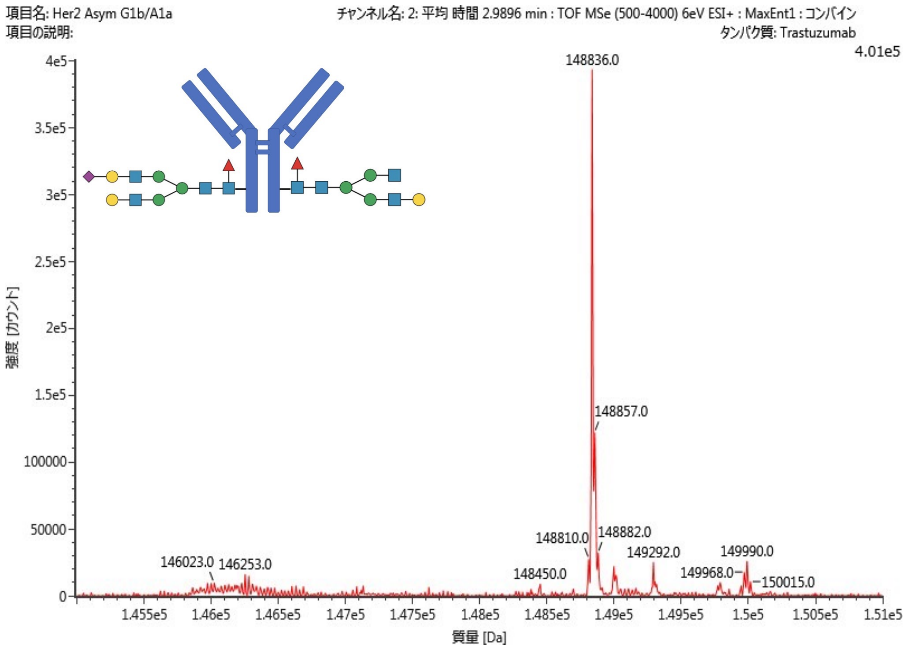

2-8 [A1a-F/G1b-F]

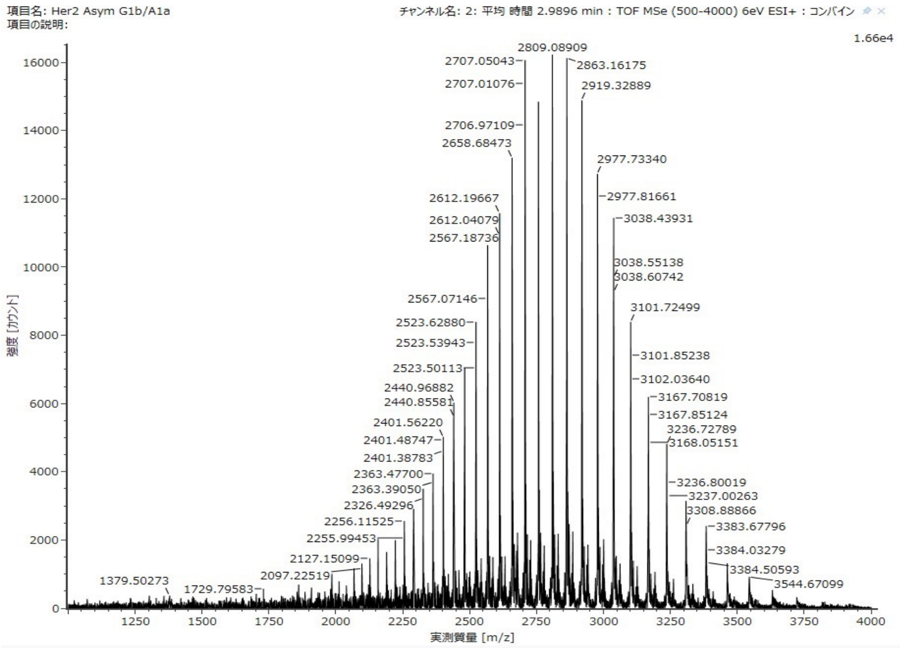

2-9 [A1a-F/G0-F]

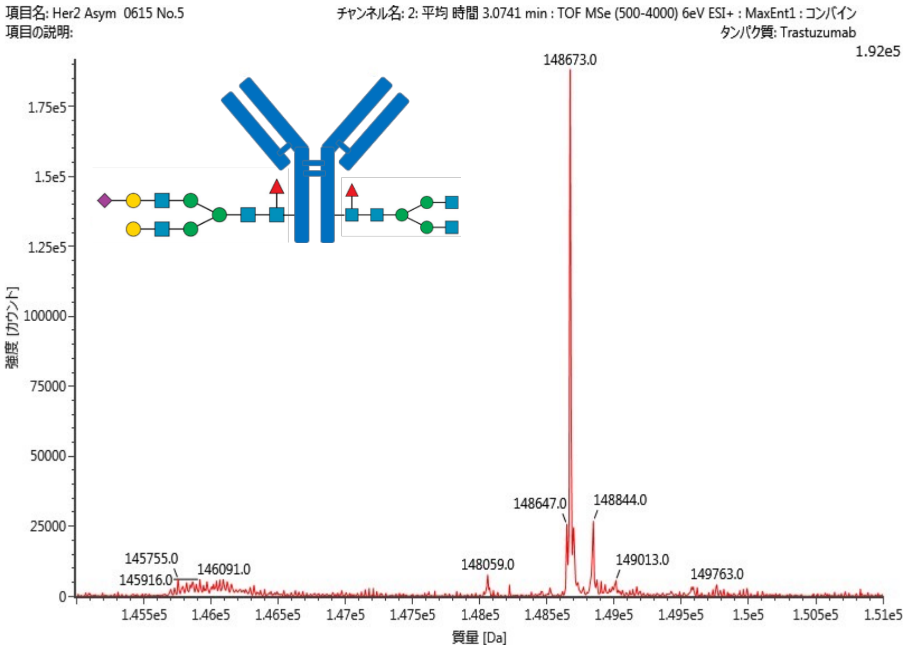

2-9 [A1a-F/G0-F]

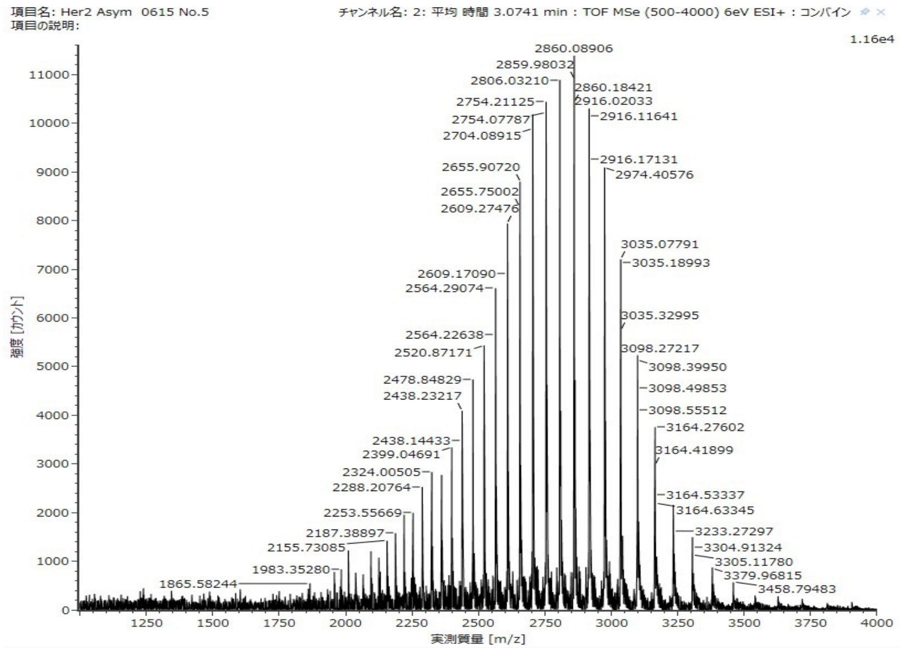

2-10 [A1a-F/M3-F]

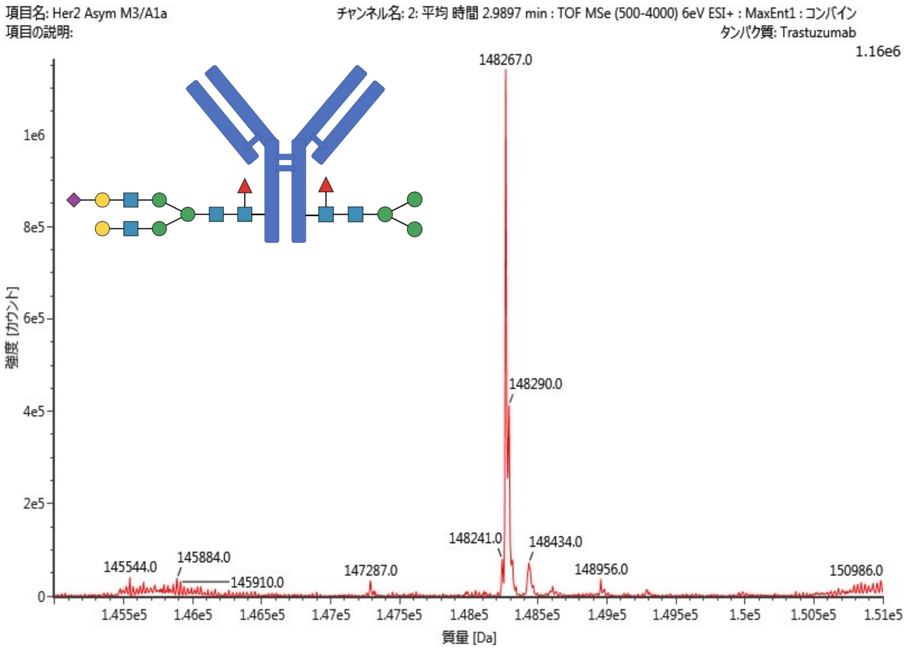

2-10 [A1a-F/M3-F]

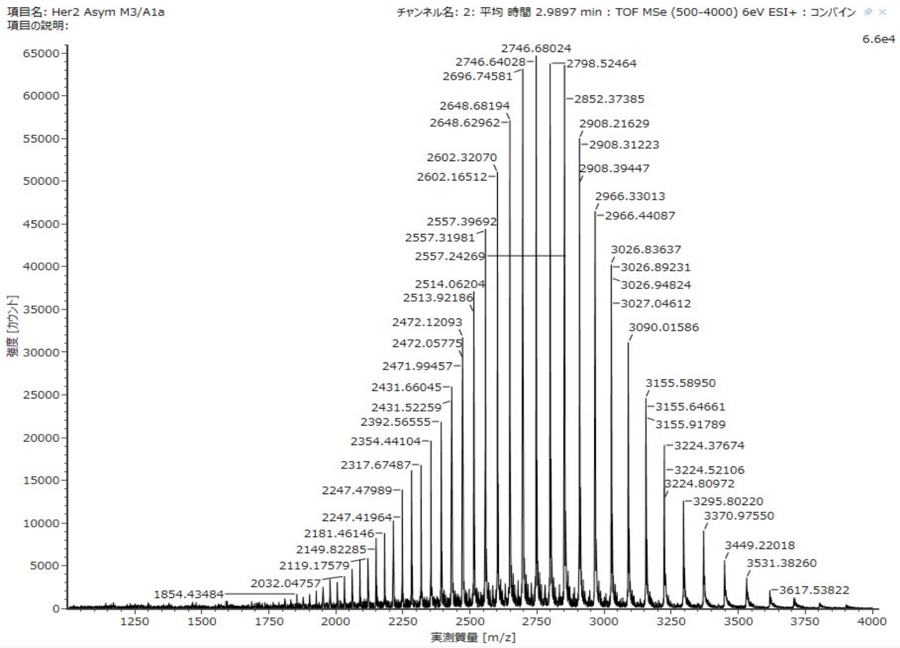

2-11 [A1a-F/GlcNAc-F]

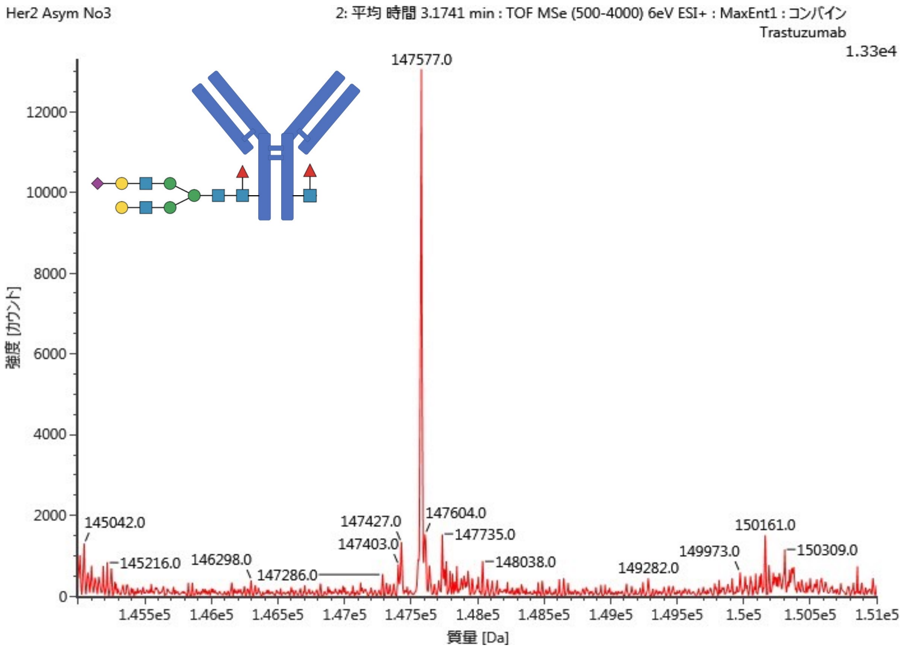

2-11 [A1a-F/GlcNAc-F]

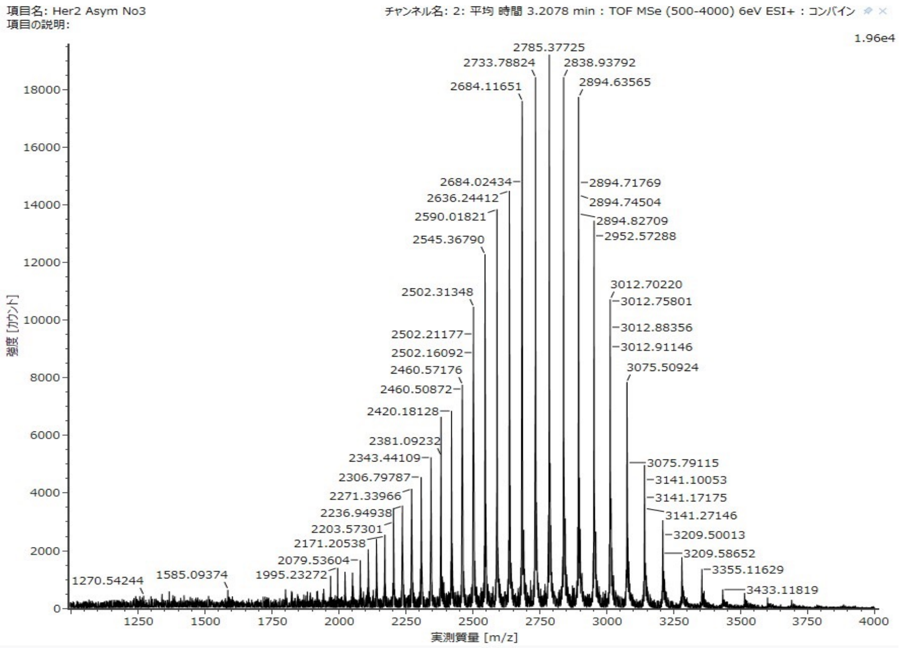

3-3 [A1b-F/A1b-F]

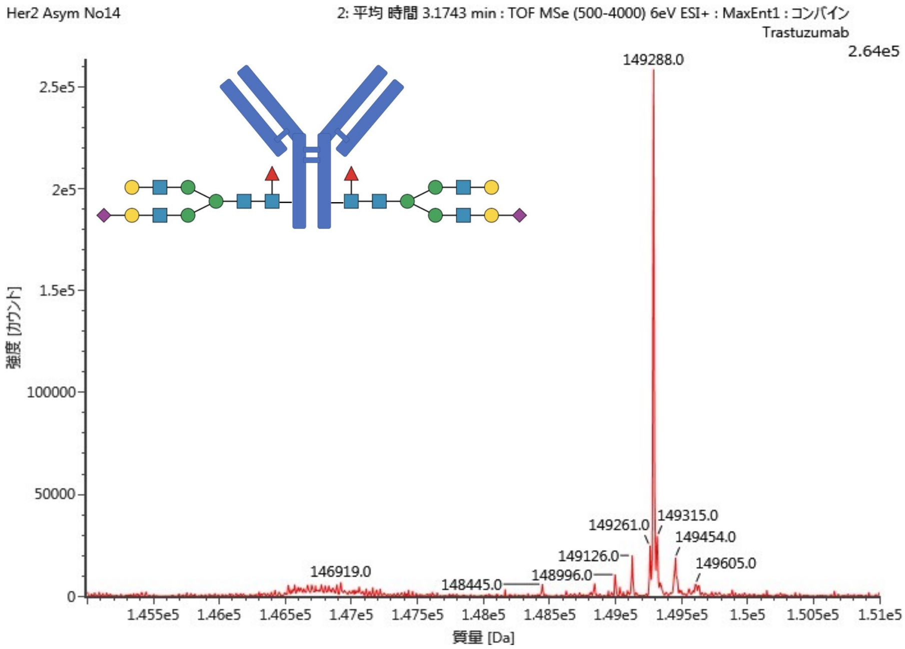

3-3 [A1b-F/A1b-F]

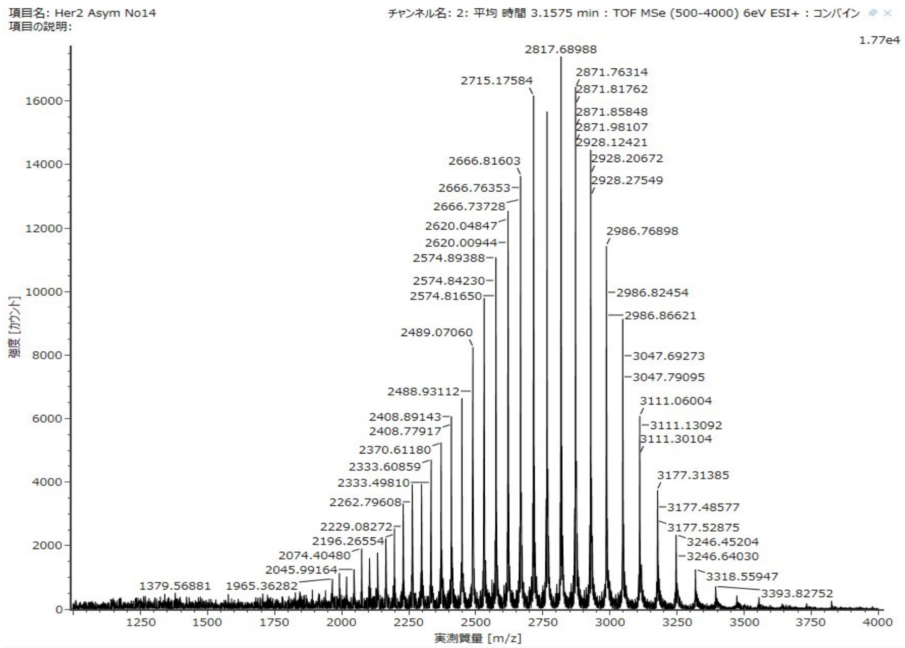

3-4 [A1b-F/A1a-Gal-F]

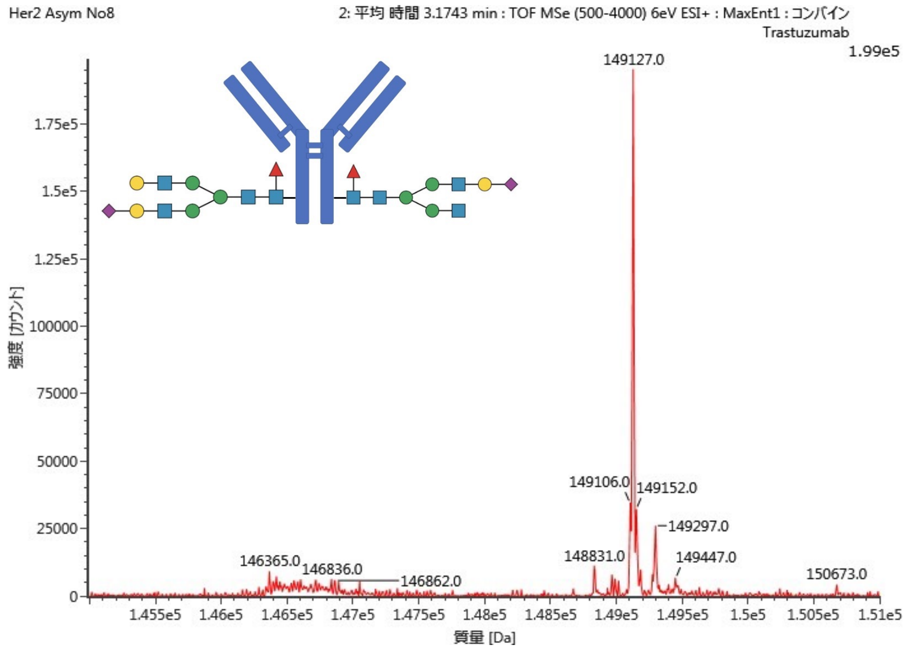

3-4 [A1b-F/A1a-Gal-F]

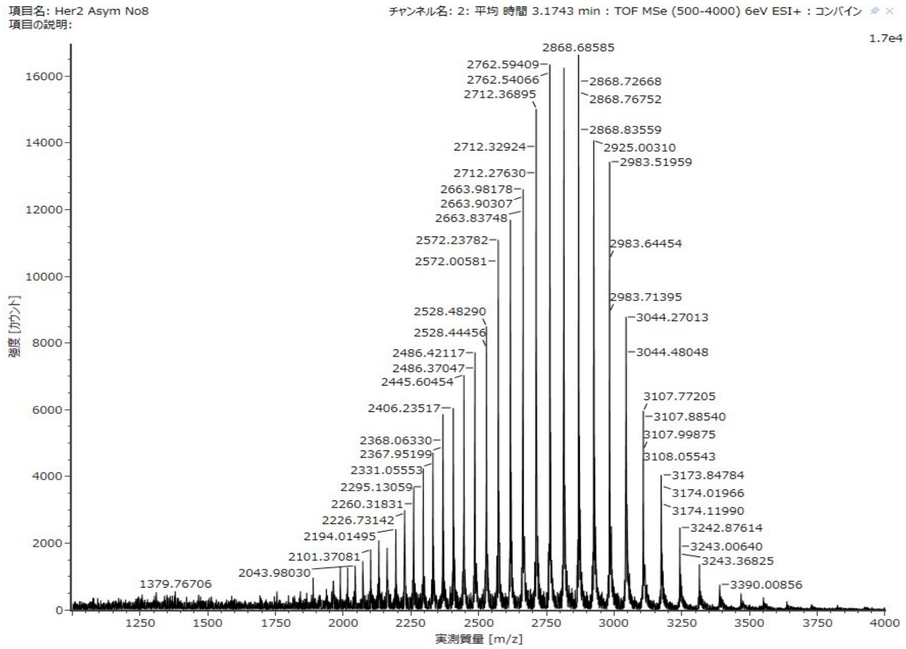

3-5 [A1b-F/A1b-Gal-F]

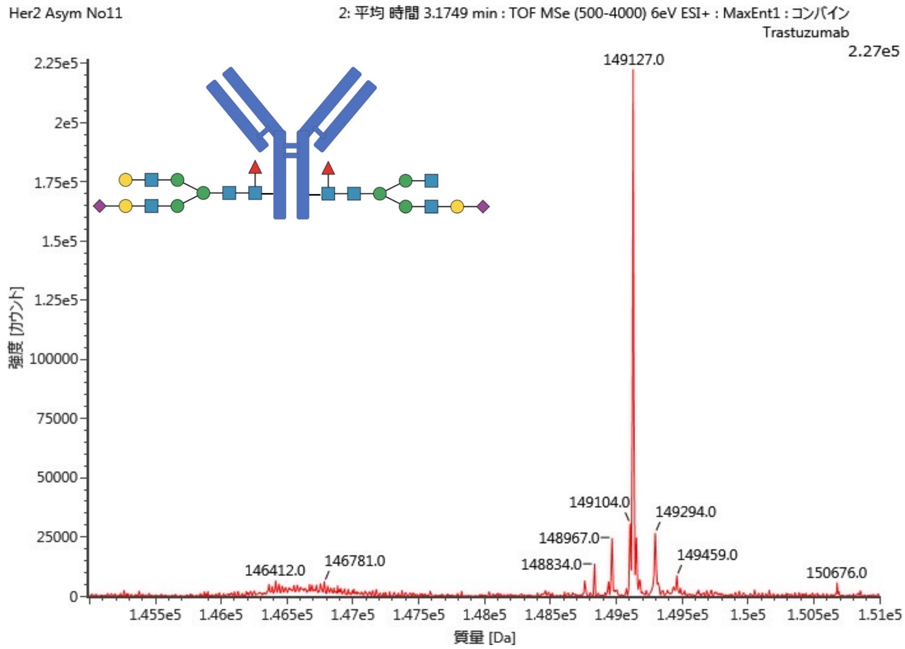

3-5 [A1b-F/A1b-Gal-F]

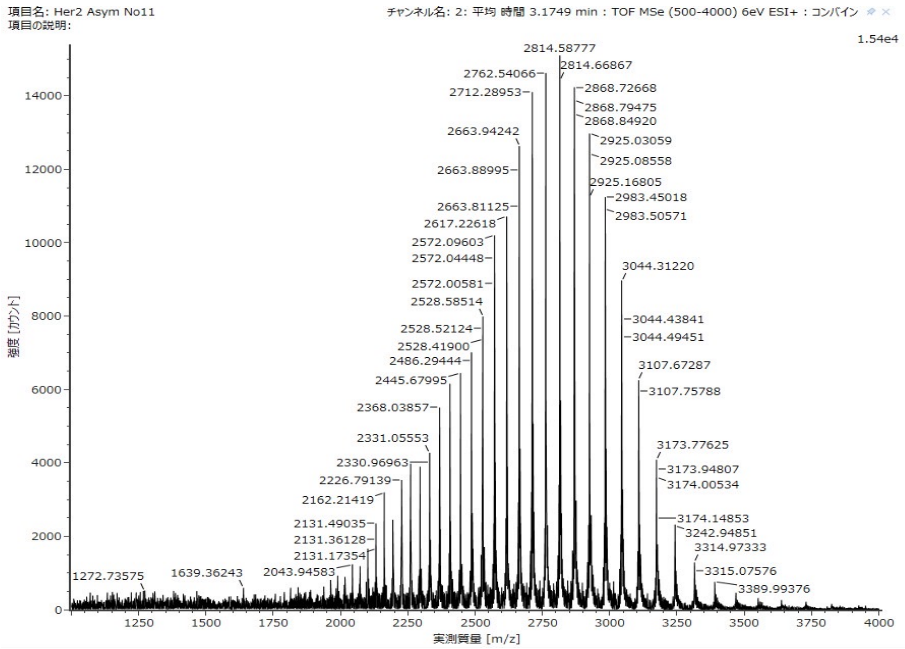

3-6 [A1b-F/G2-F]

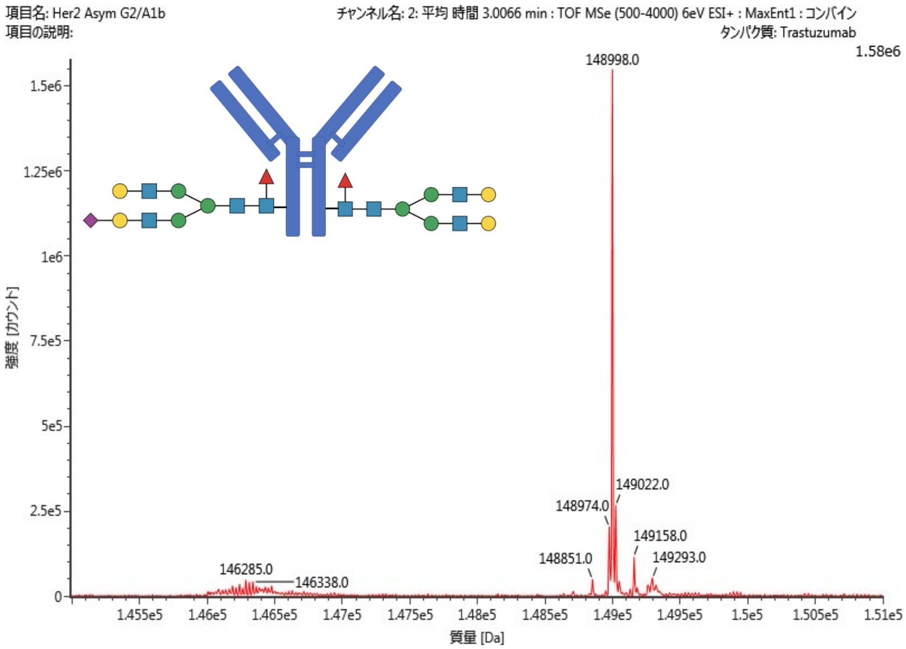

3-6 [A1b-F/G2-F]

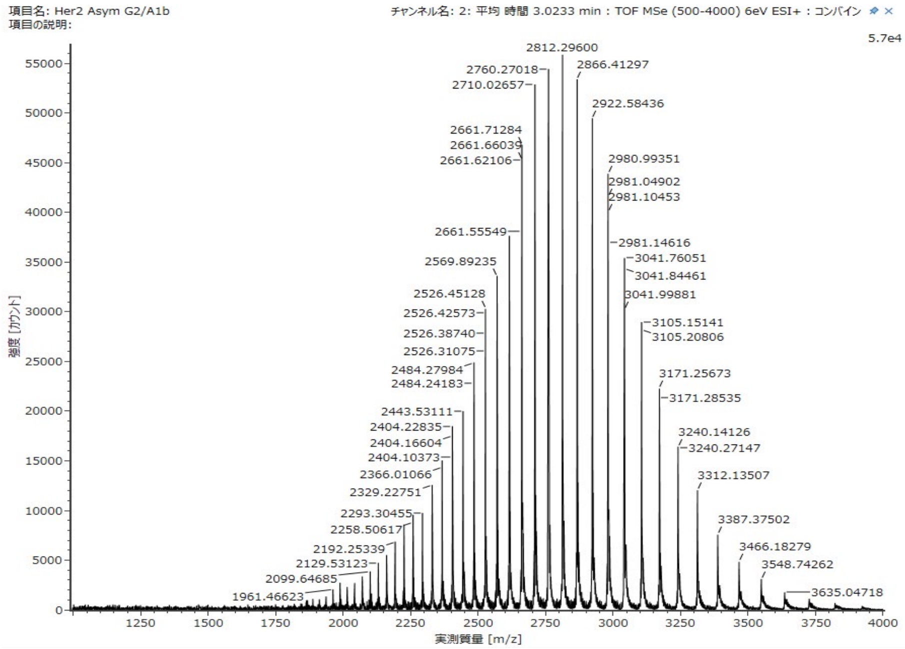

3-7 [A1b-F/G1a-F]

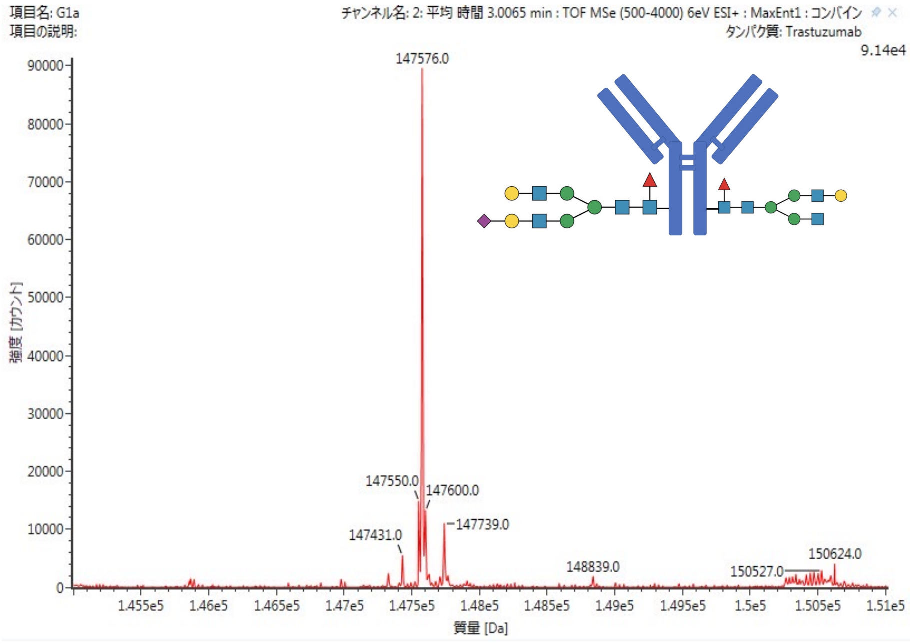

3-7 [A1b-F/G1a-F]

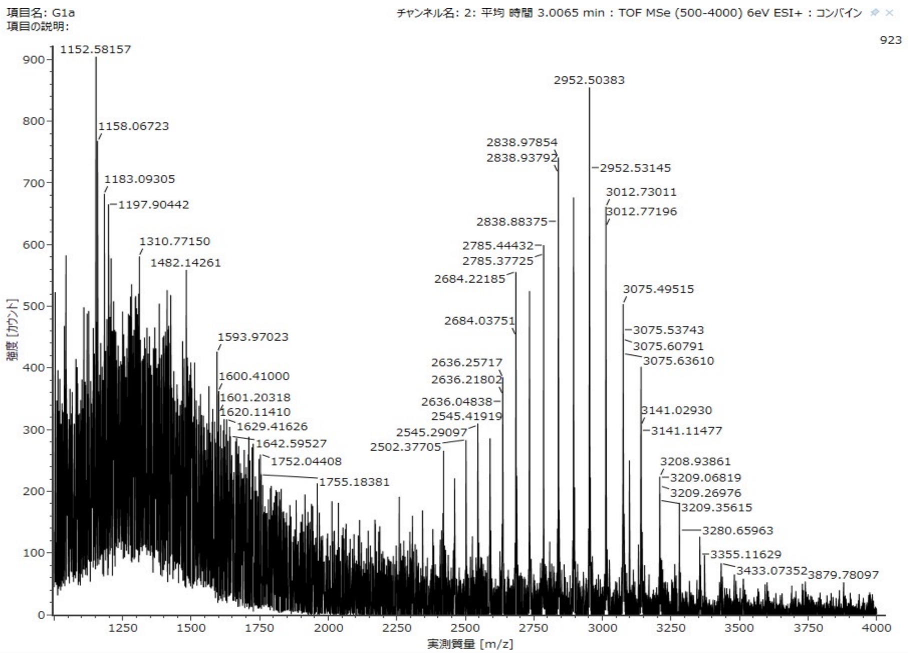

3-8 [A1b-F/G1b-F]

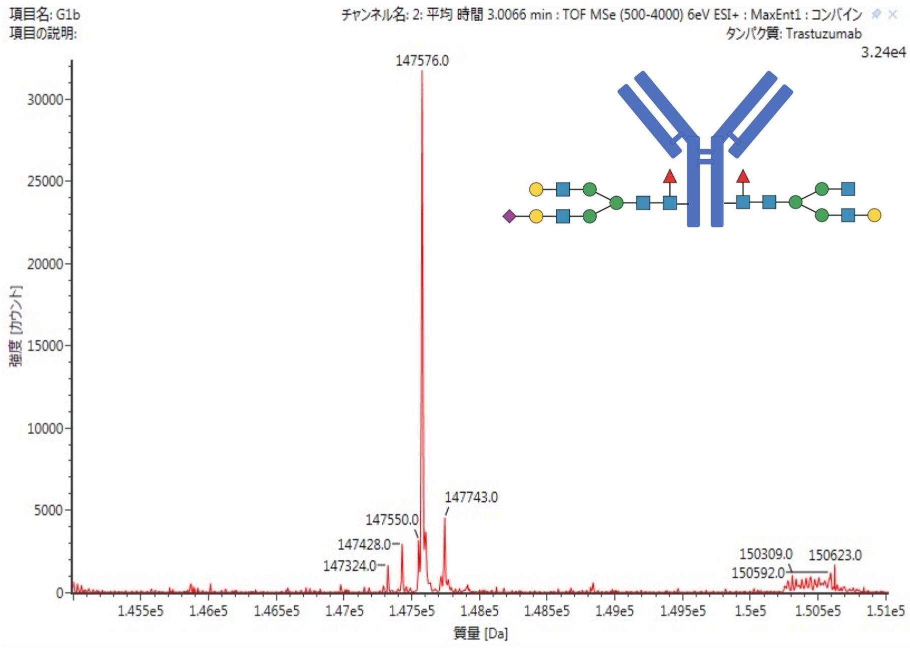

3-8 [A1b-F/G1b-F]

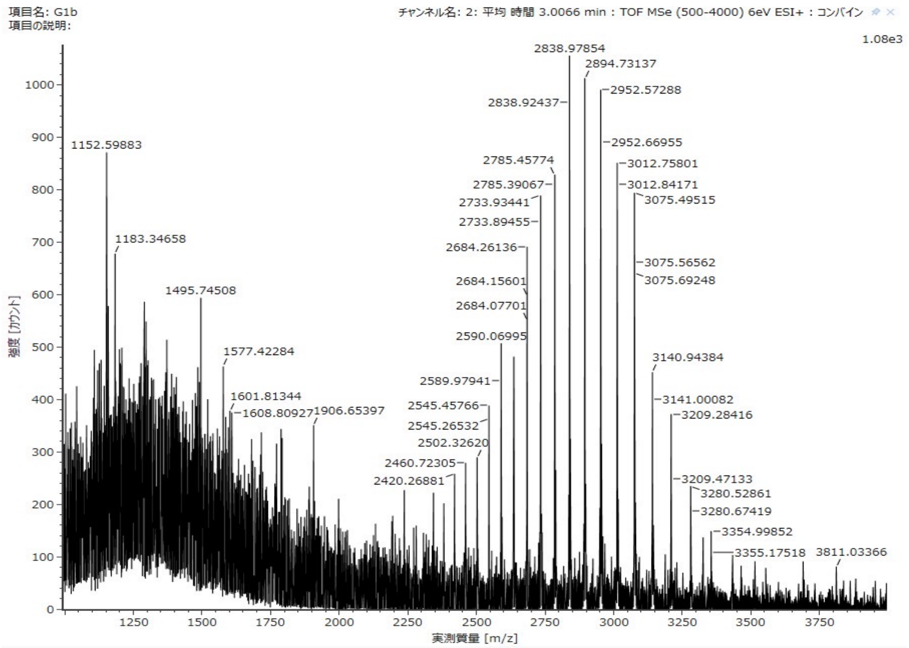

3-9 [A1b-F/G0-F]

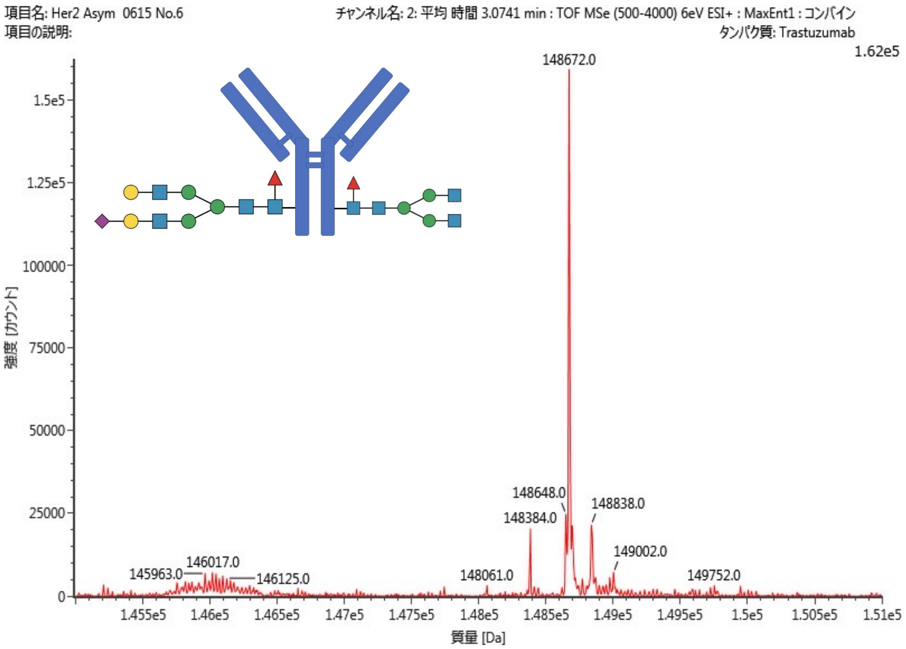

3-9 [A1b-F/G0-F]

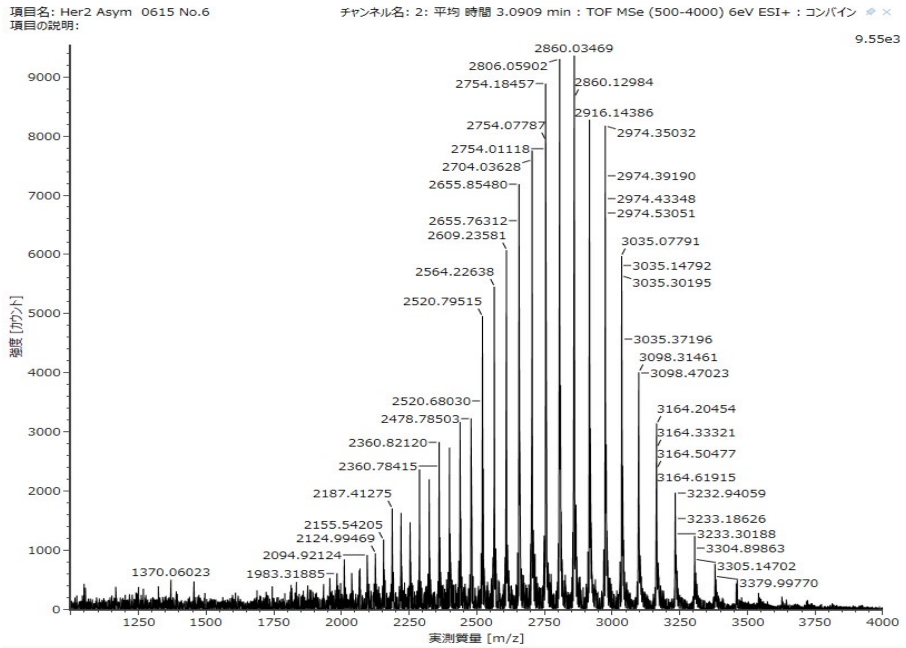

3-10 [A1b-F/M3-F]

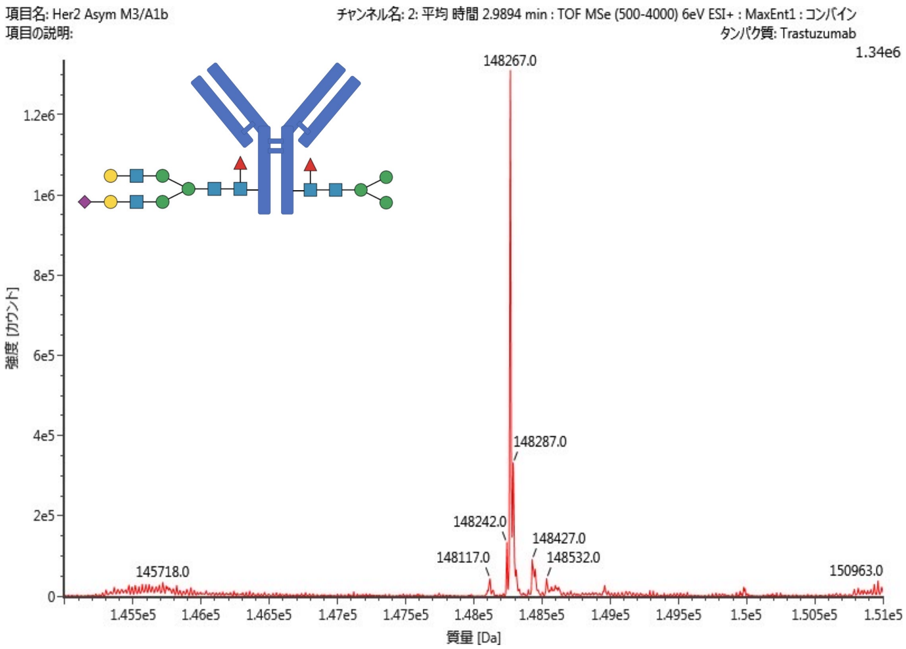

3-10 [A1b-F/M3-F]

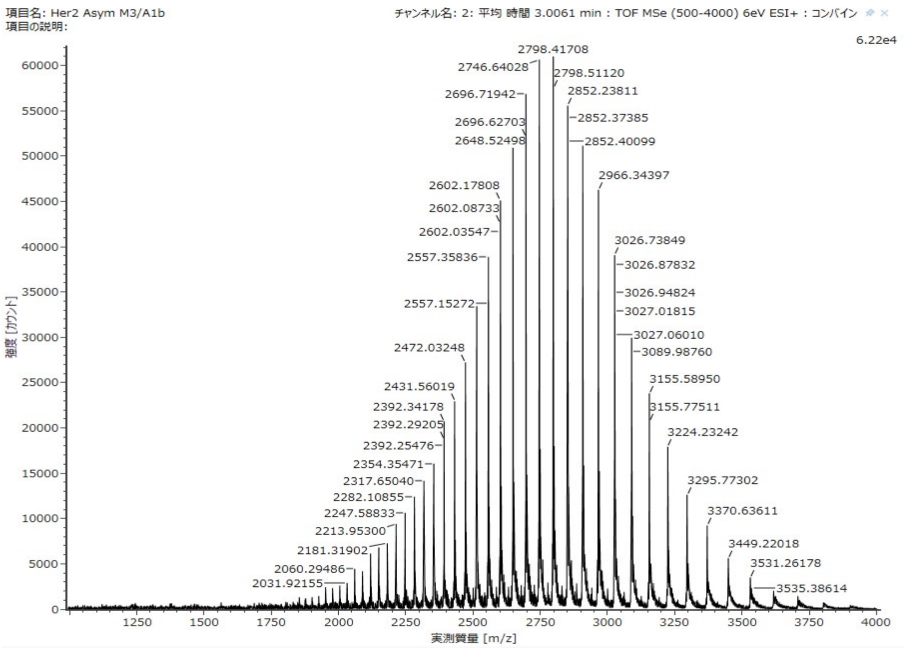

3-11 [A1b-F/GlcNAc-F]

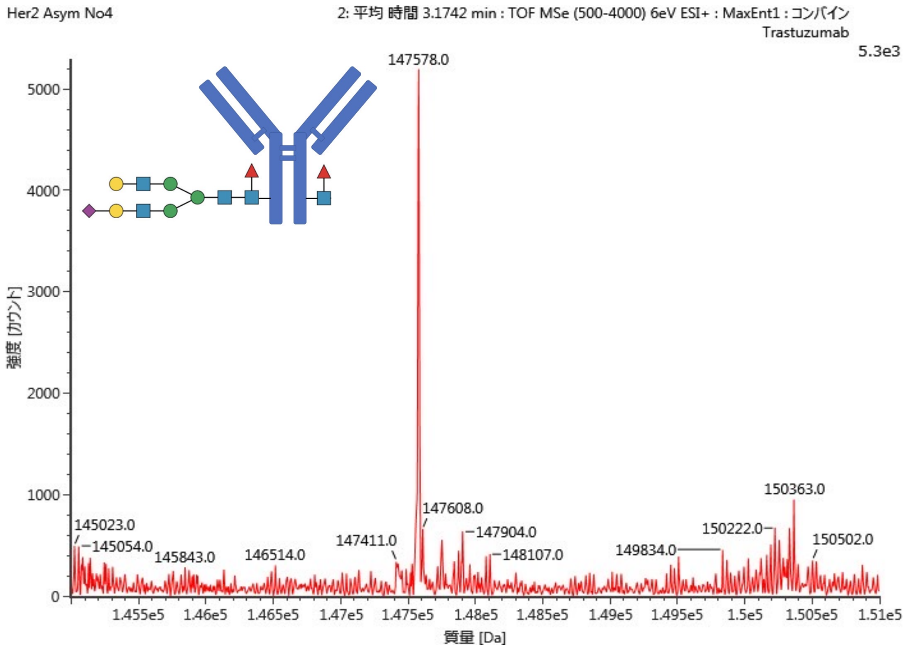

3-11 [A1b-F/GlcNAc-F]

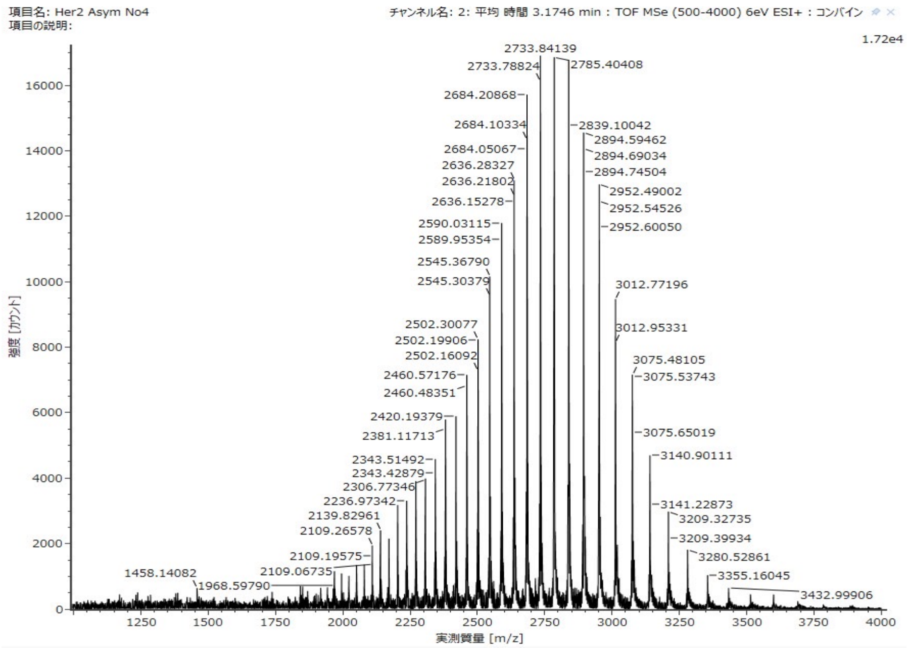

4-4 [A1a-Gal-F/A1a-Gal-F]

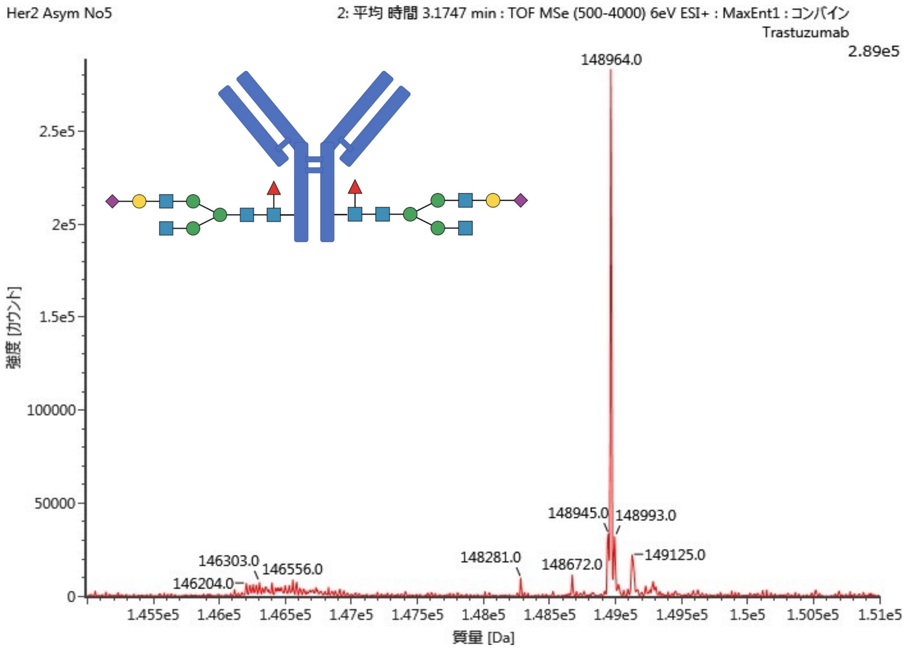

4-4 [A1a-Gal-F/A1a-Gal-F]

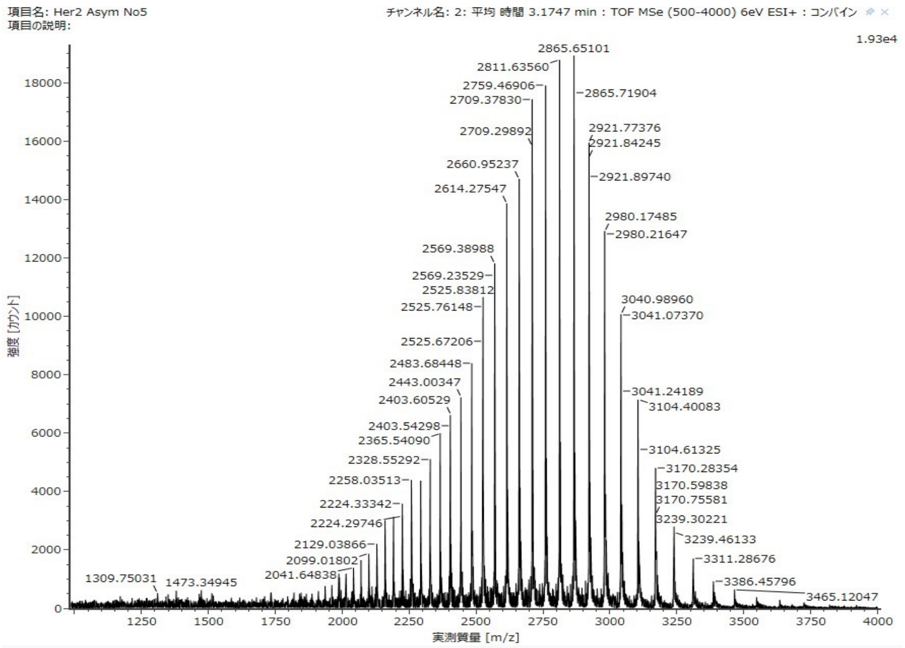

4-5 [A1a-Gal-F/A1b-Gal-F]

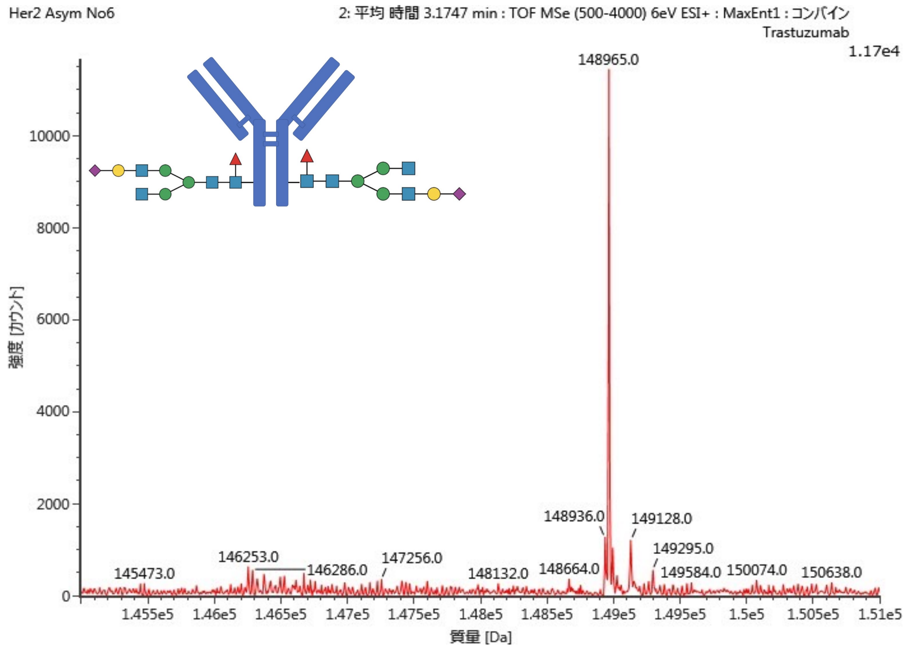

4-5 [A1a-Gal-F/A1b-Gal-F]

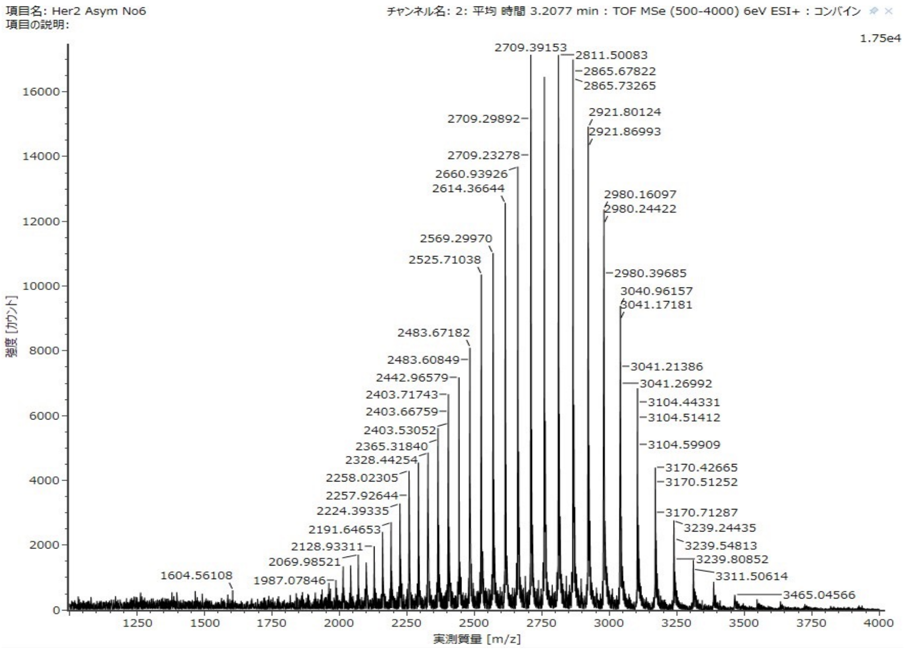

4-6 [A1a-Gal-F/G2-F]

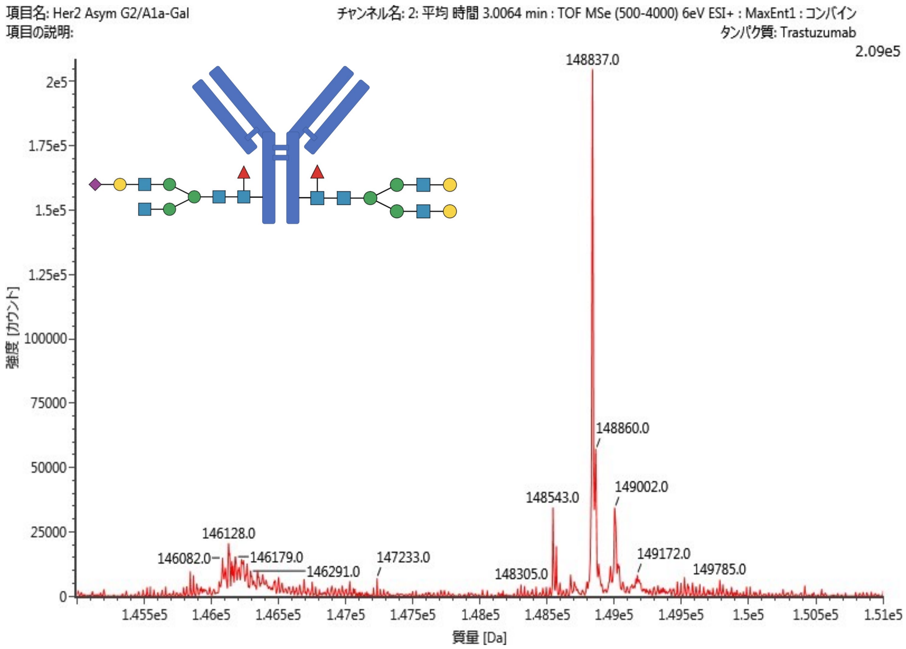

4-6 [A1a-Gal-F/G2-F]

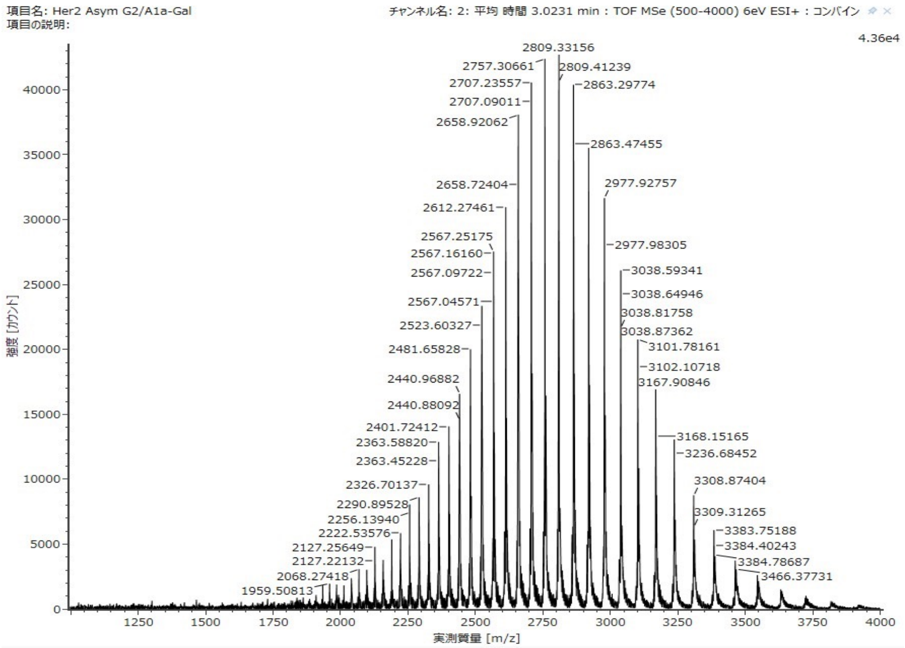

4-7 [A1a-Gal-F/G1a-F]

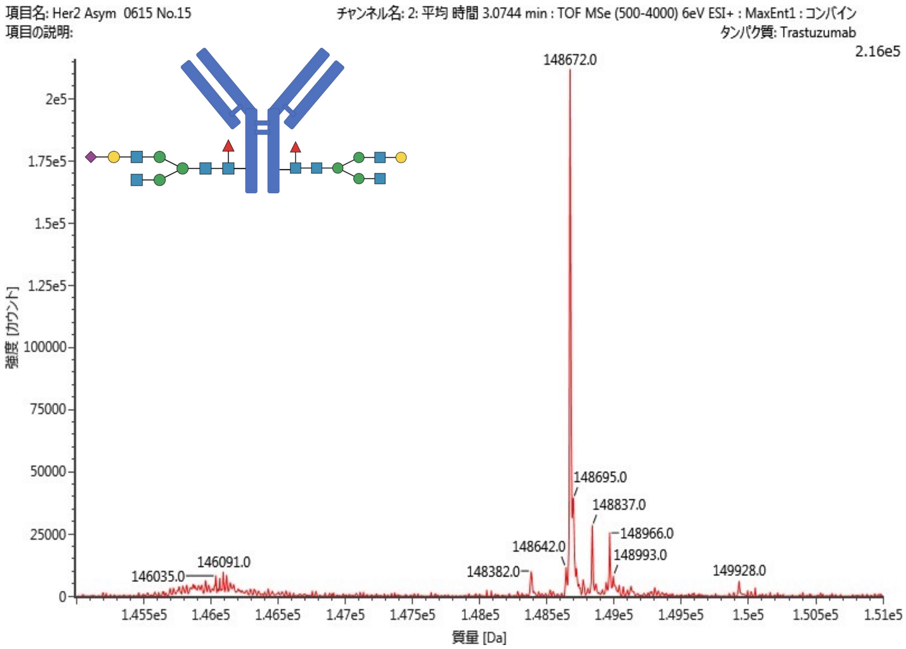

4-7 [A1a-Gal-F/G1a-F]

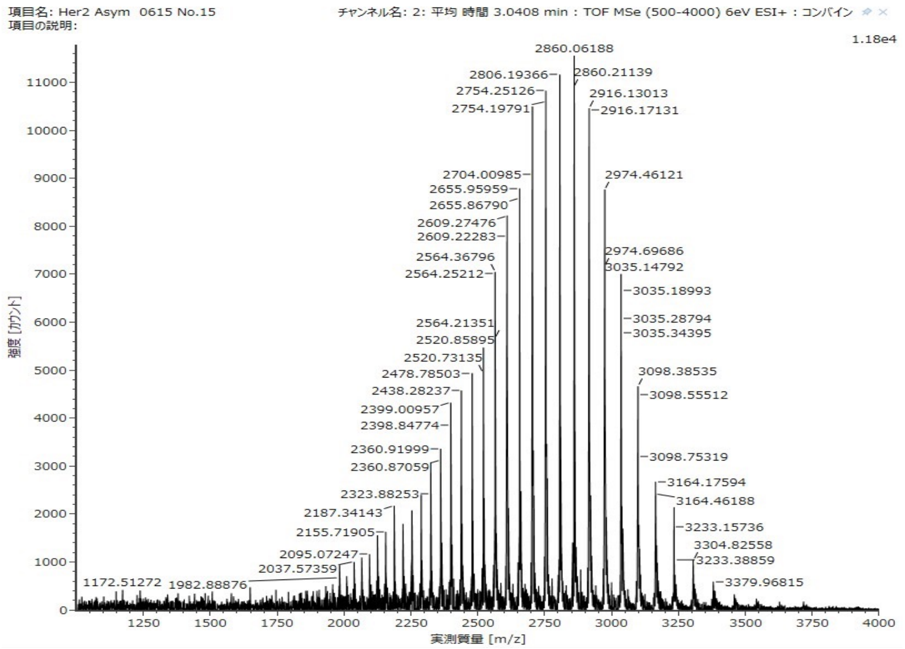

4-8 [A1a-Gal-F/G1b-F]

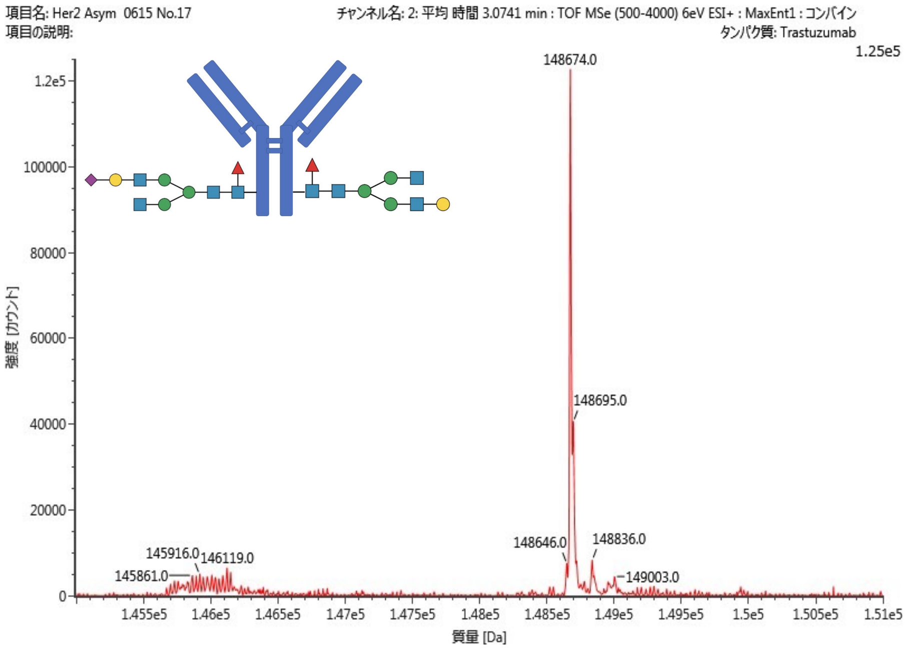

4-8 [A1a-Gal-F/G1b-F]

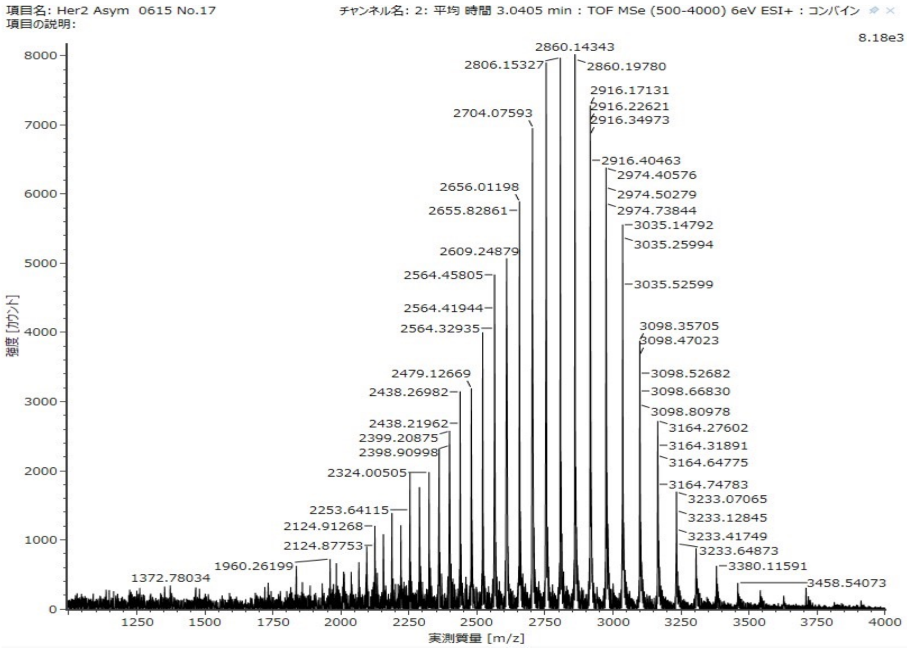

4-9 [A1a-Gal-F/G0-F]

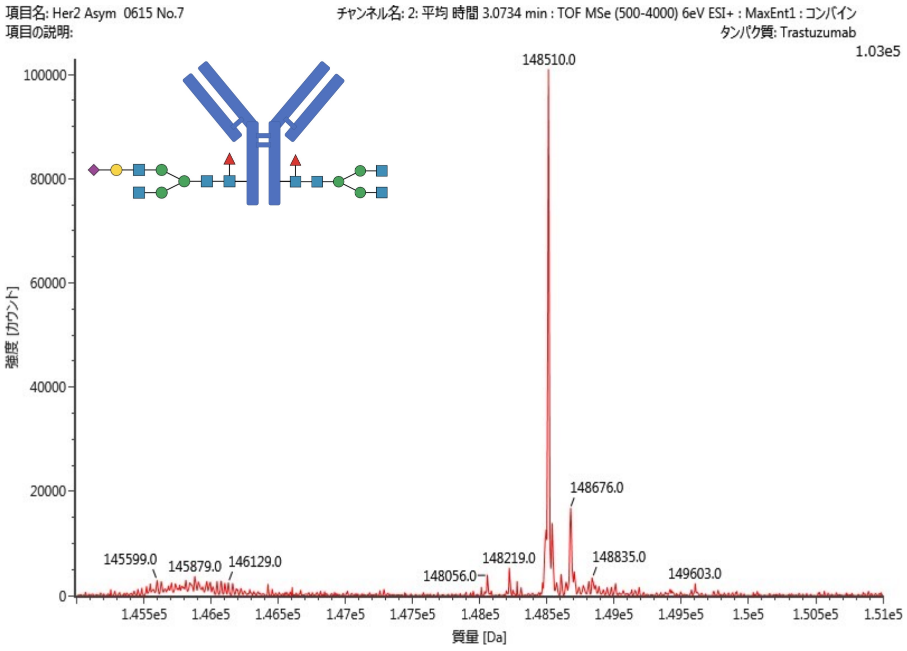

4-9 [A1a-Gal-F/G0-F]

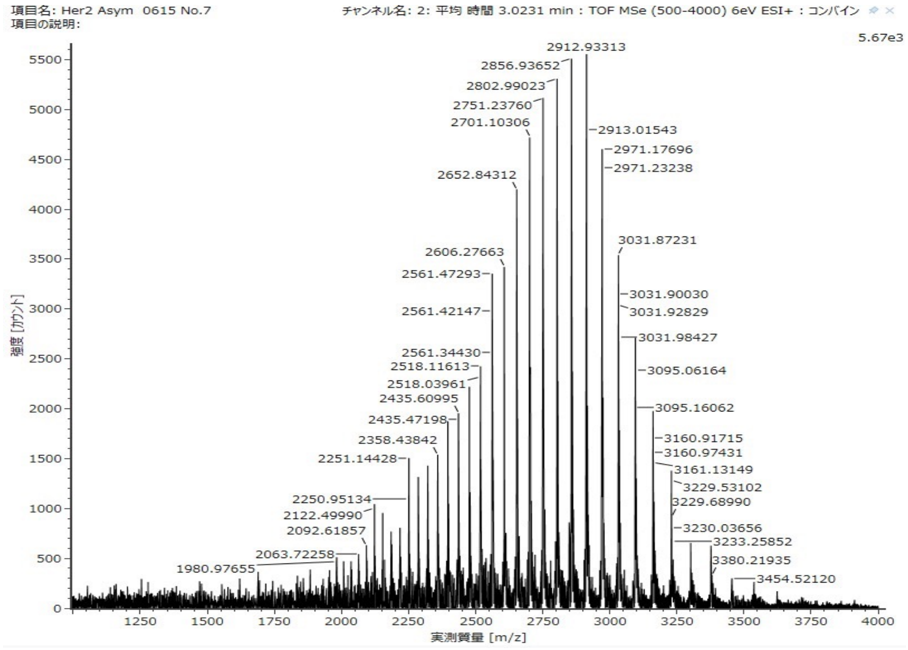

4-10 [A1a-Gal-F/M3-F]

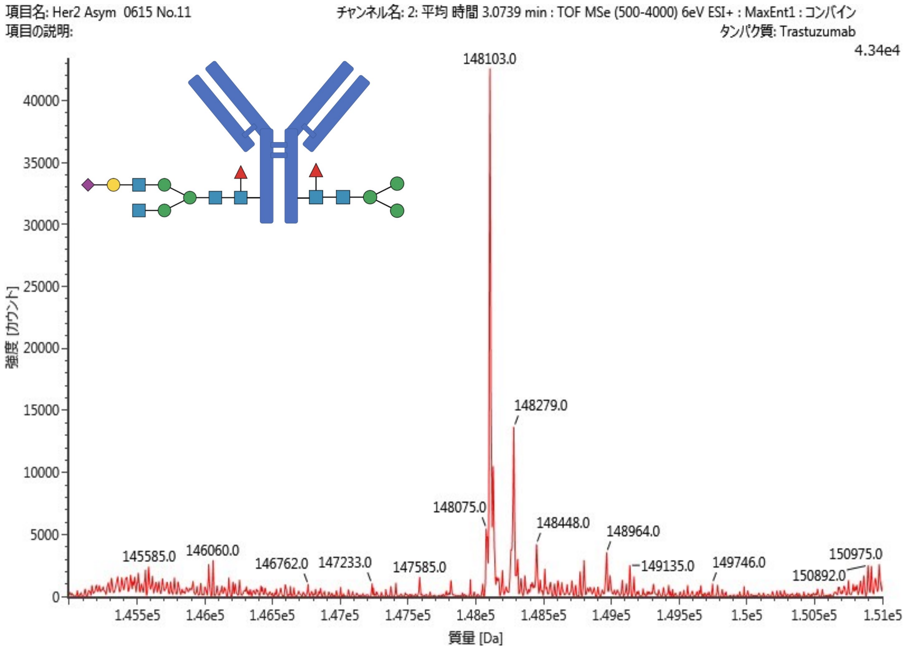

4-10 [A1a-Gal-F/M3-F]

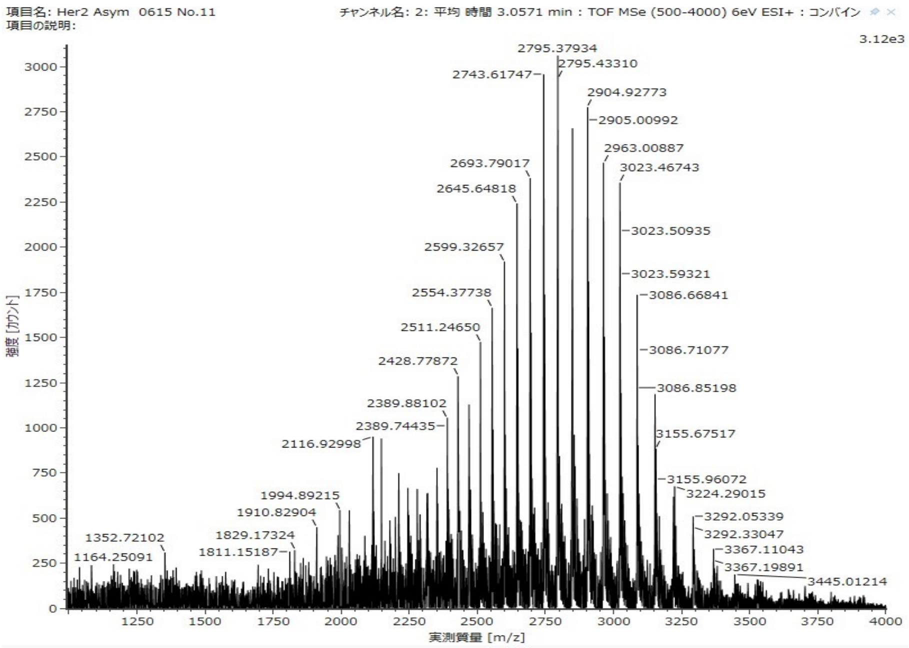

4-11 [A1a-Gal-F/GlcNAc-F]

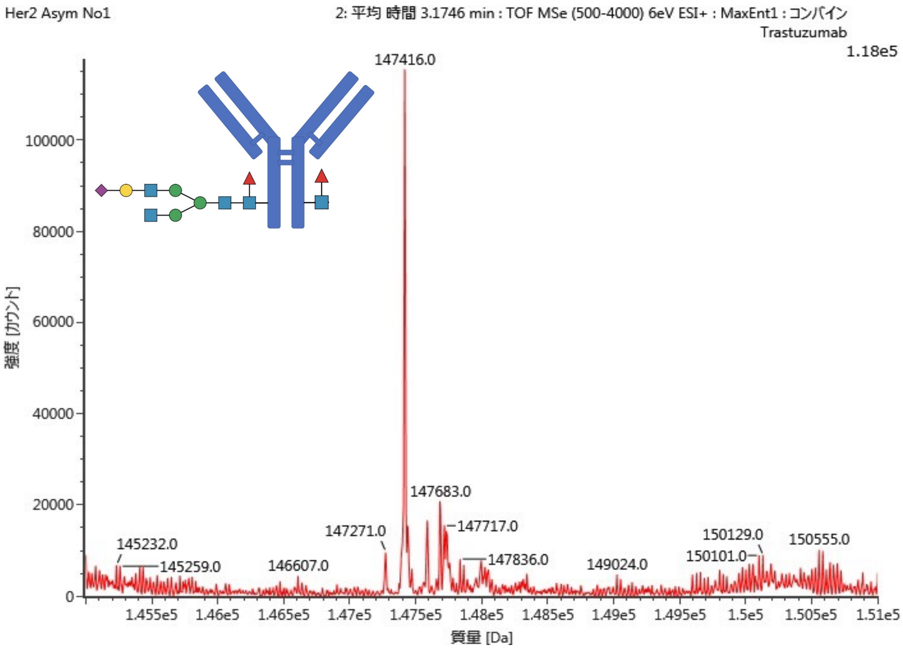

4-11 [A1a-Gal-F/GlcNAc-F]

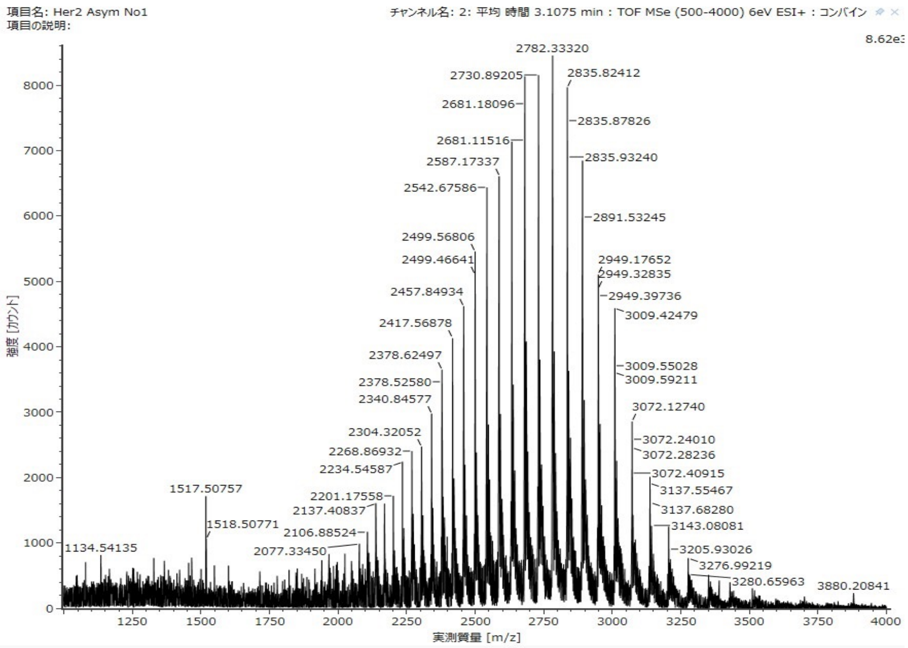

5-5 [A1b-Gal-F/A1b-Gal-F]

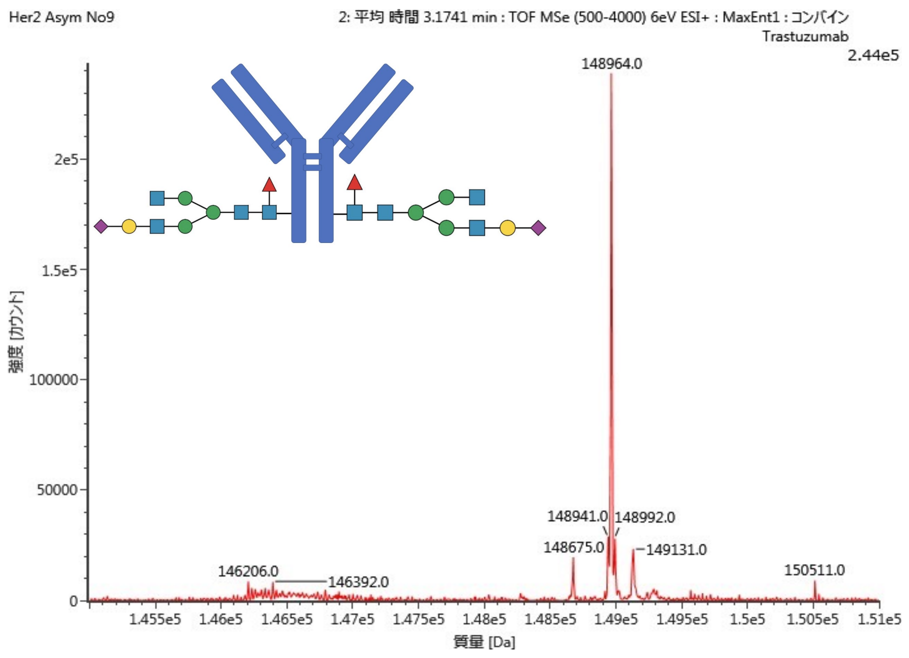

5-5 [A1b-Gal-F/A1b-Gal-F]

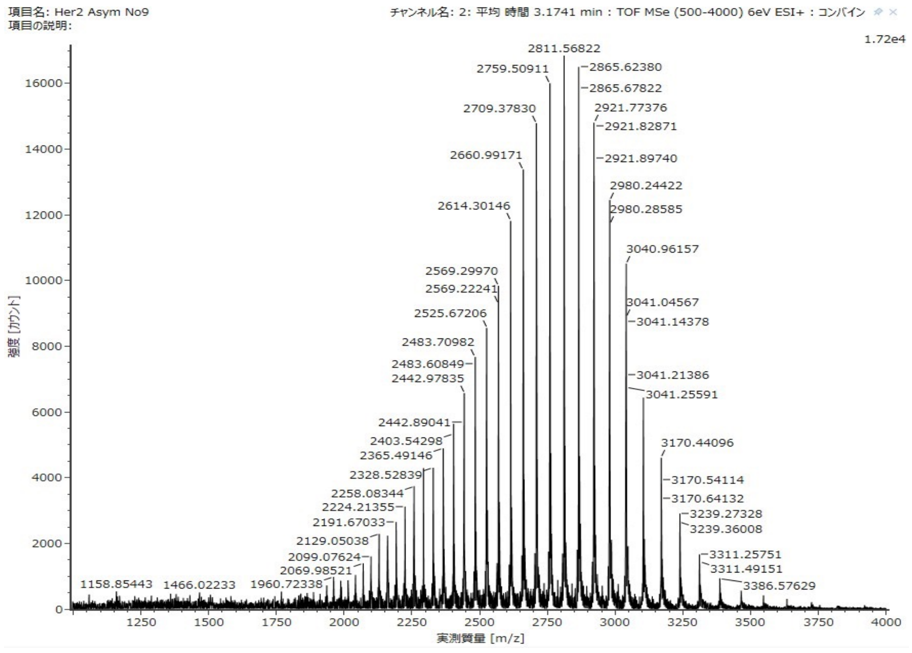

5-6 [A1b-Gal-F/G2-F]

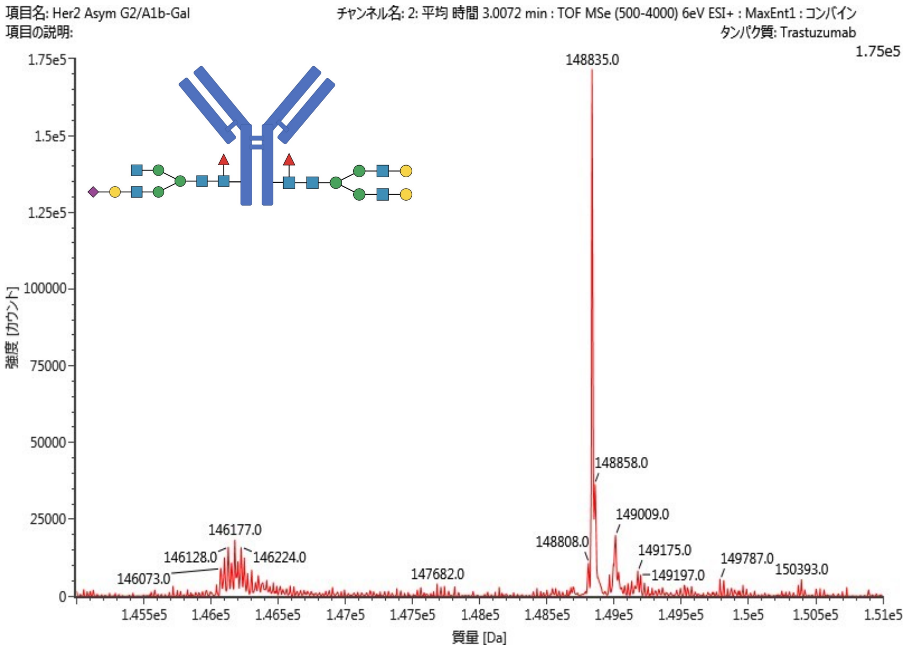

5-6 [A1b-Gal-F/G2-F]

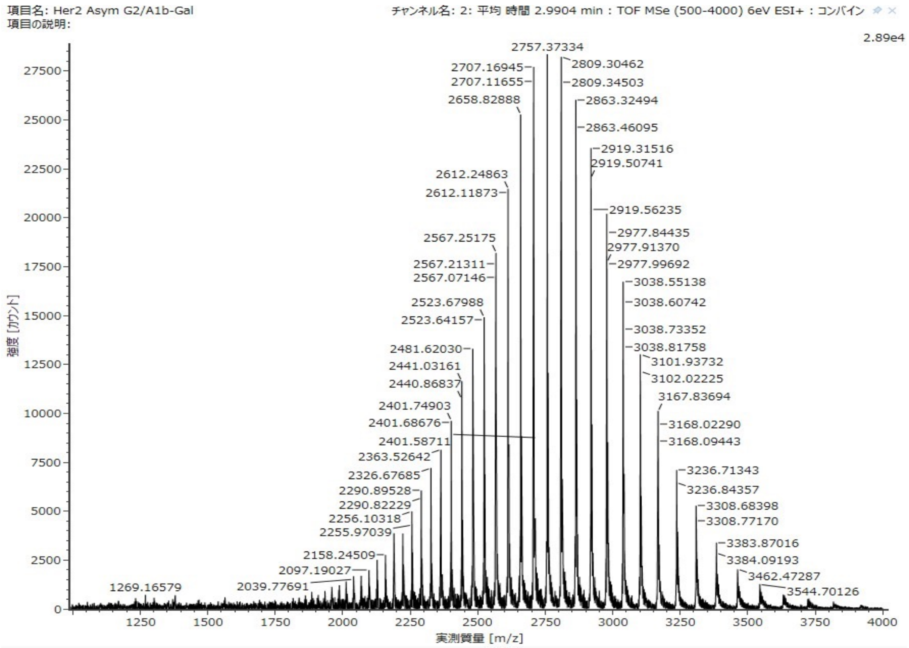

5-7 [A1b-Gal-F/G1a-F]

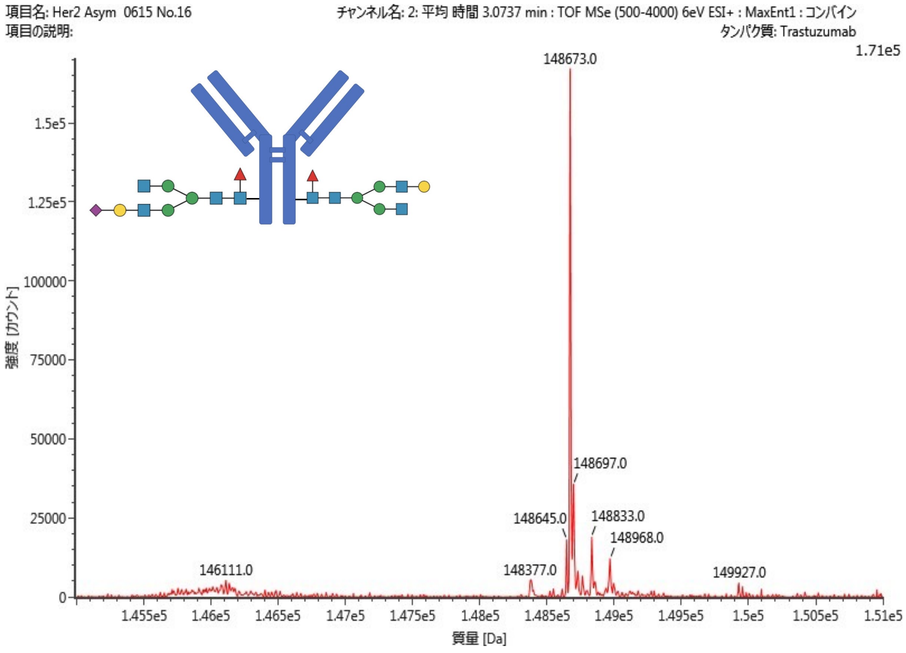

5-7 [A1b-Gal-F/G1a-F]

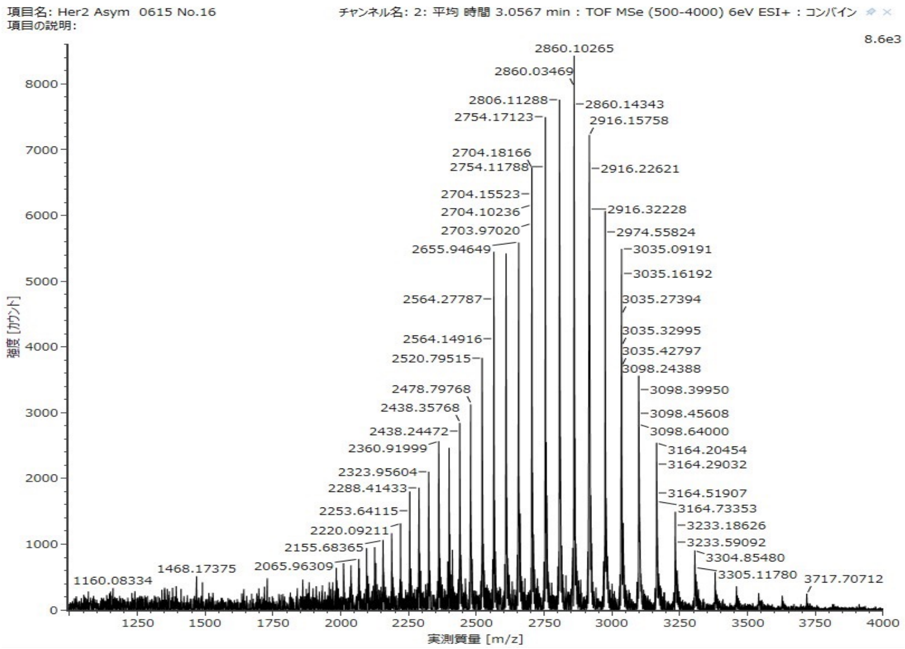

5-8 [A1b-Gal-F/G1b-F]

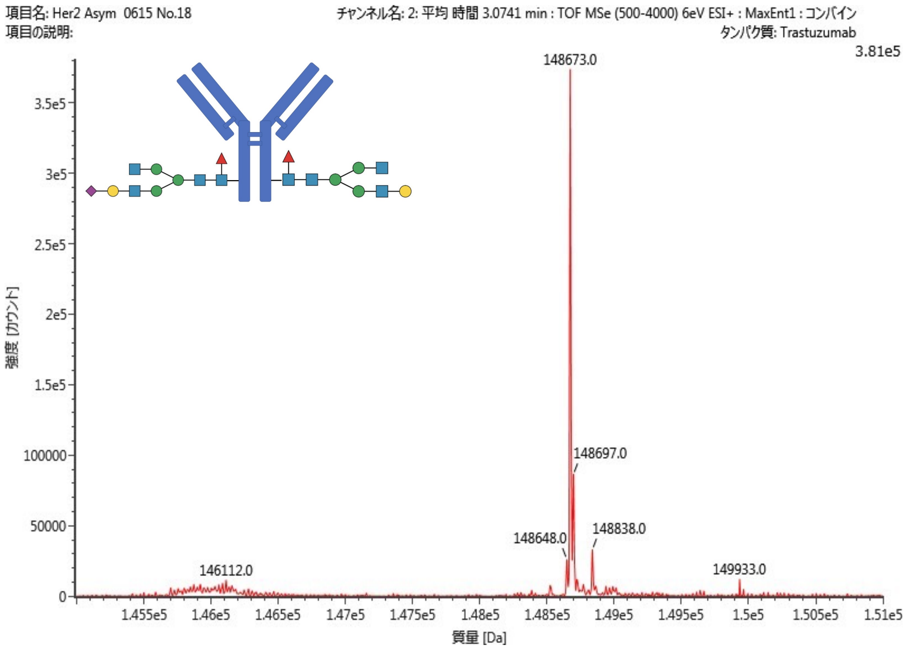

5-8 [A1b-Gal-F/G1b-F]

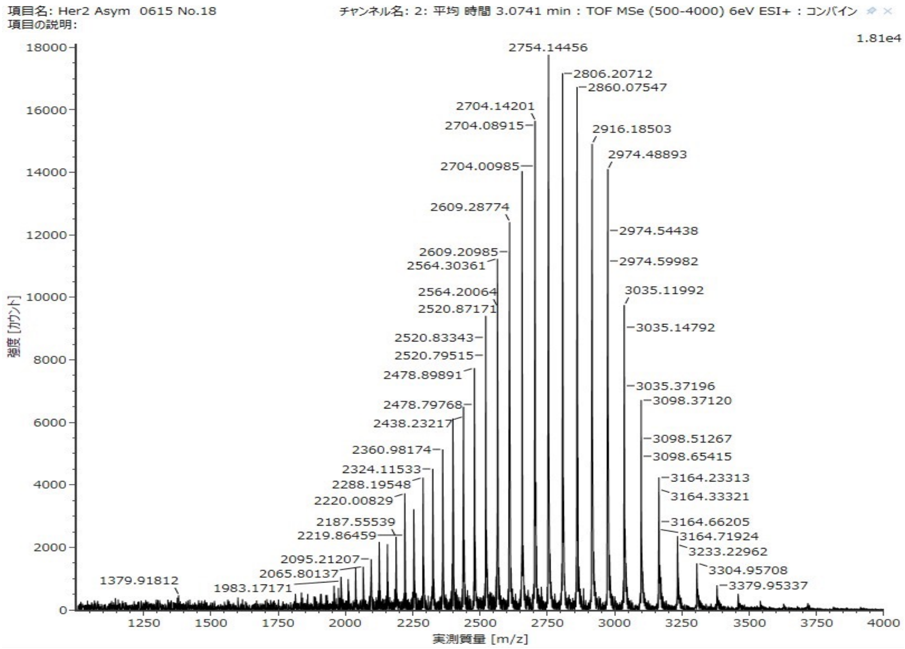

5-9 [A1b-Gal-F/G0-F]

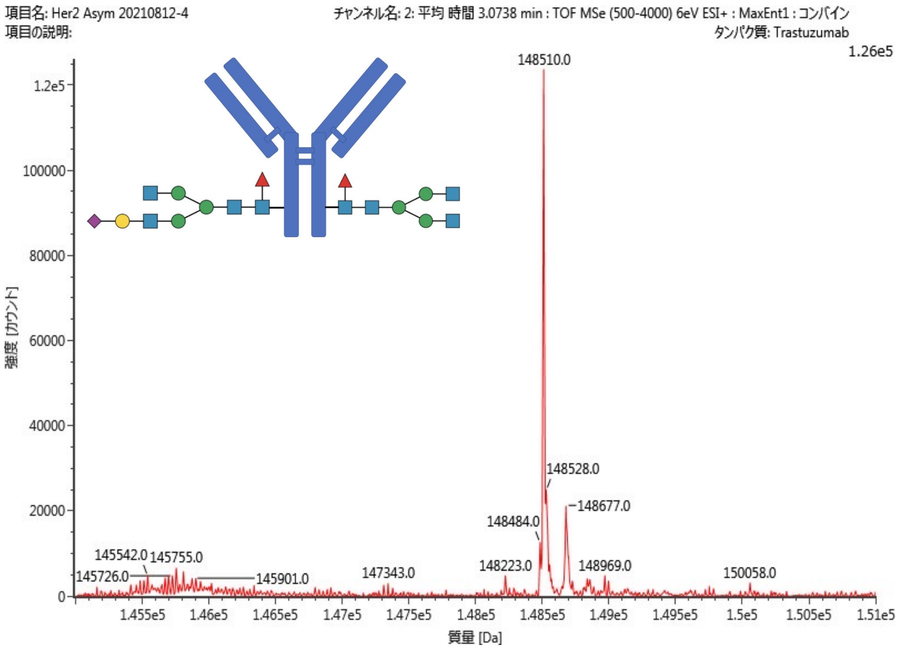

5-9 [A1b-Gal-F/G0-F]

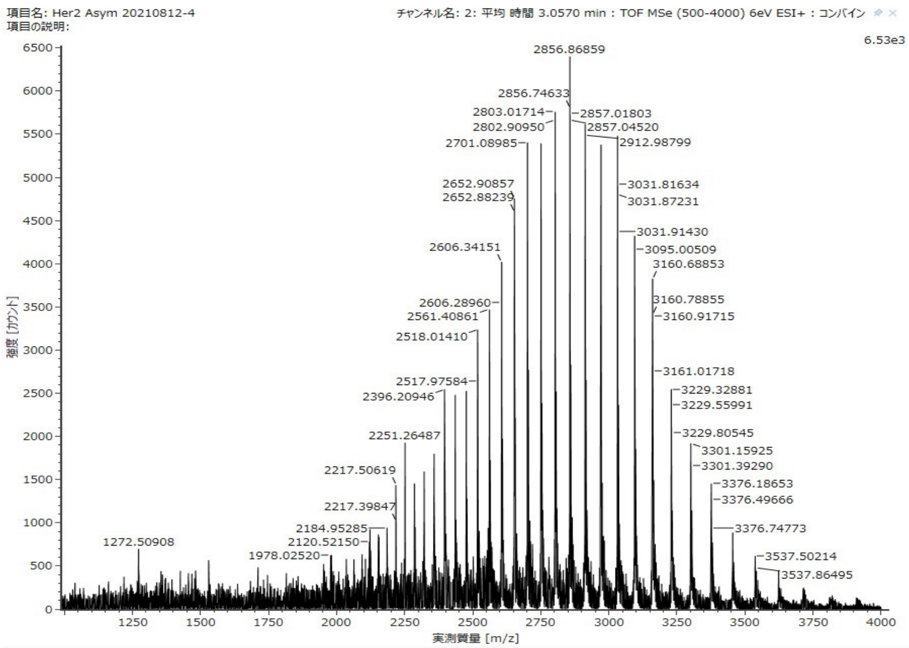

5-10 [A1b-Gal-F/M3-F]

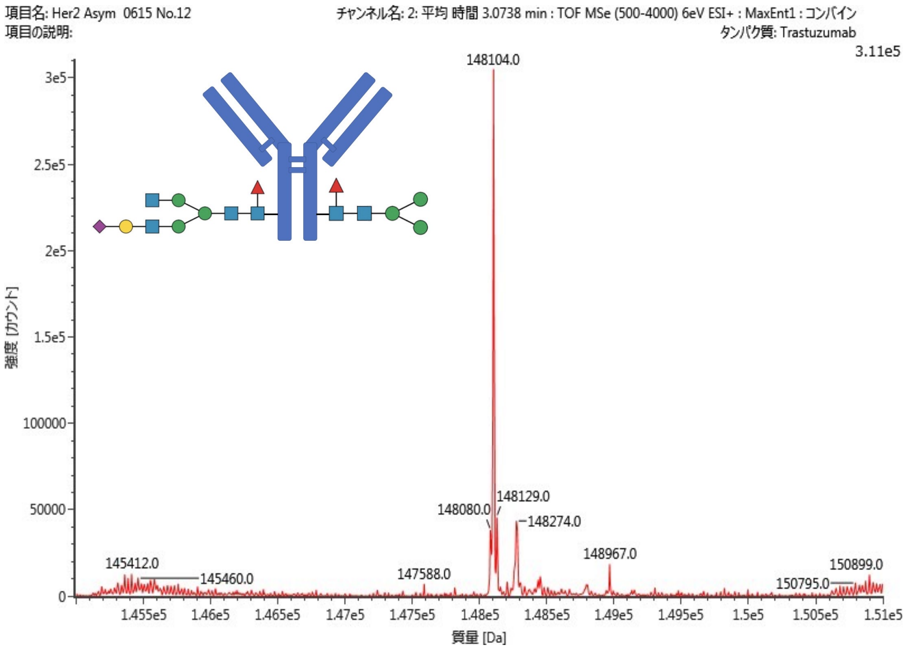

5-10 [A1b-Gal-F/M3-F]

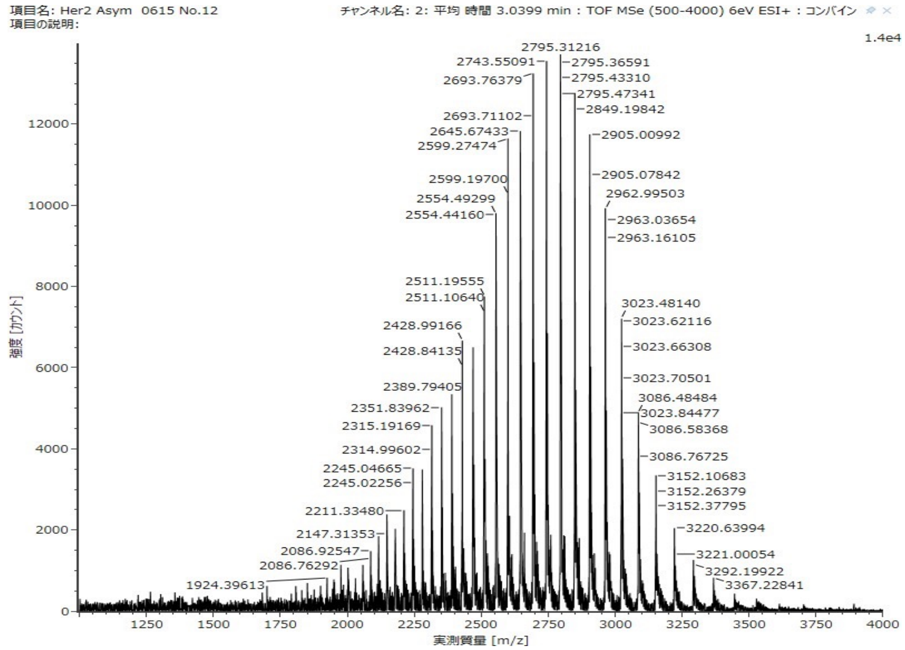

5-11 [A1b-Gal-F/GlcNAc-F]

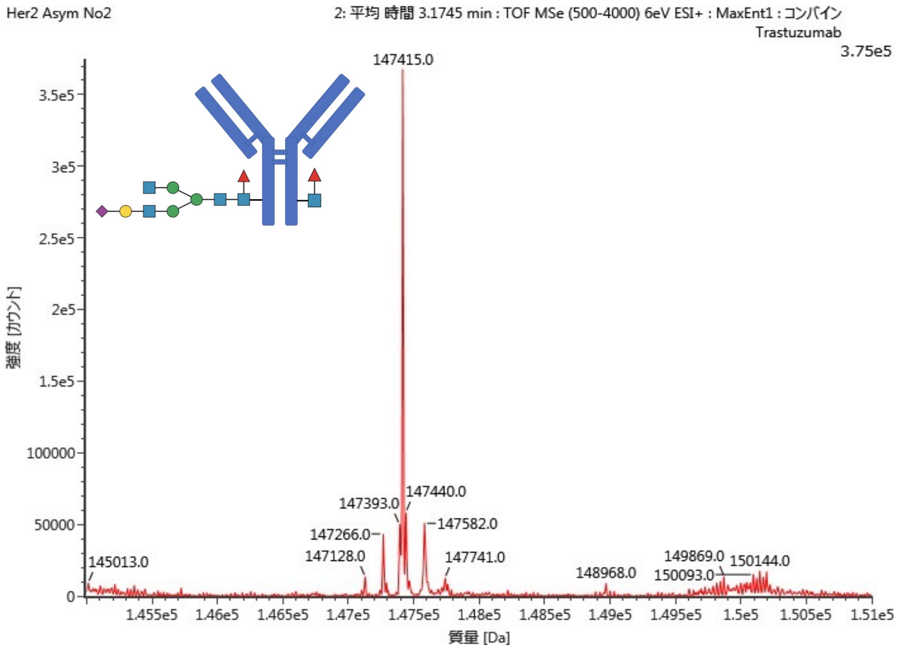

5-11 [A1b-Gal-F/GlcNAc-F]

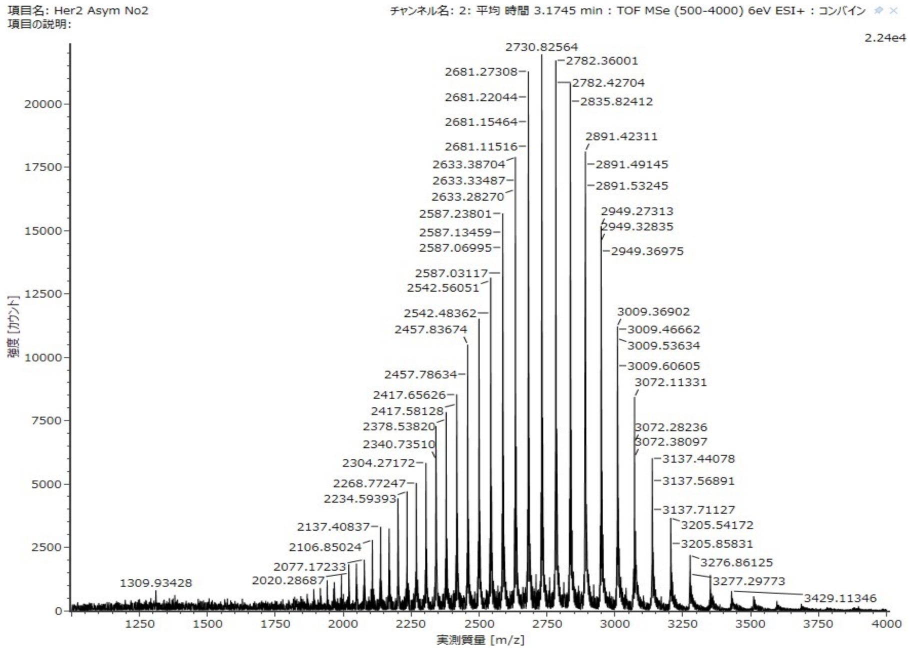

6-6 [G2-F/G2-F]

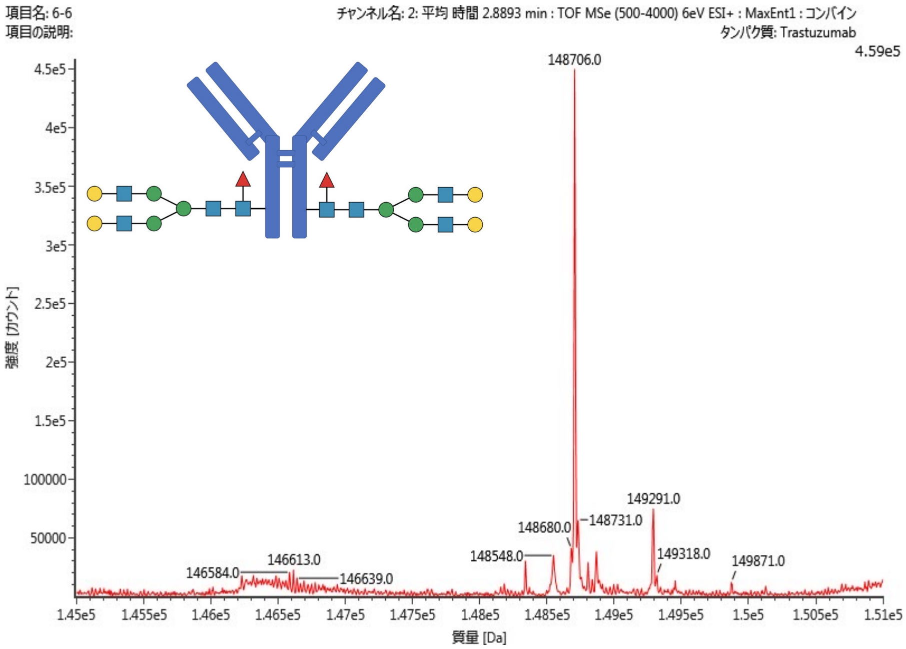

6-6 [G2-F/G2-F]

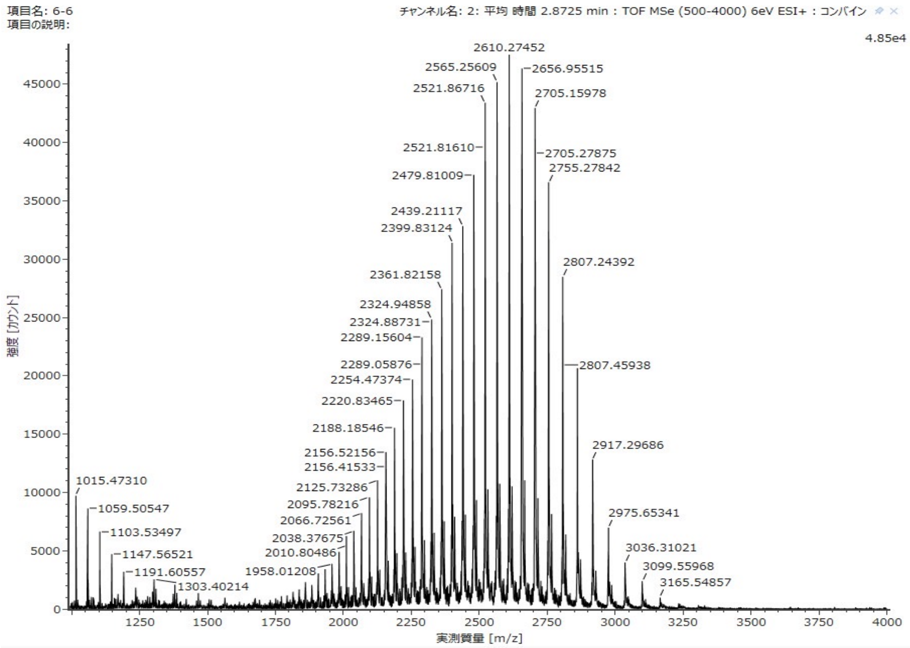

6-7 [G2-F/G1a-F]

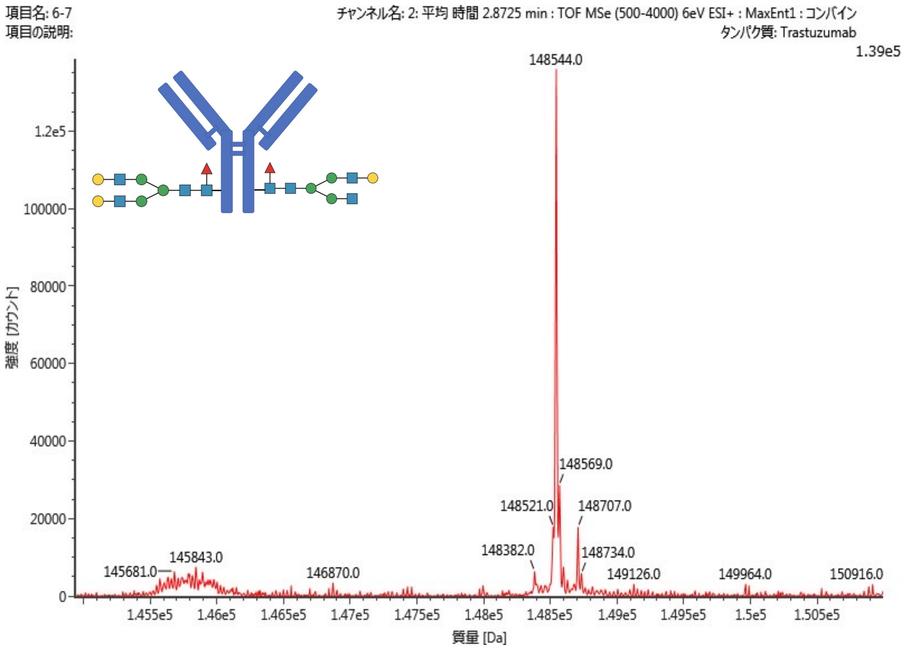

6-7 [G2-F/G1a-F]

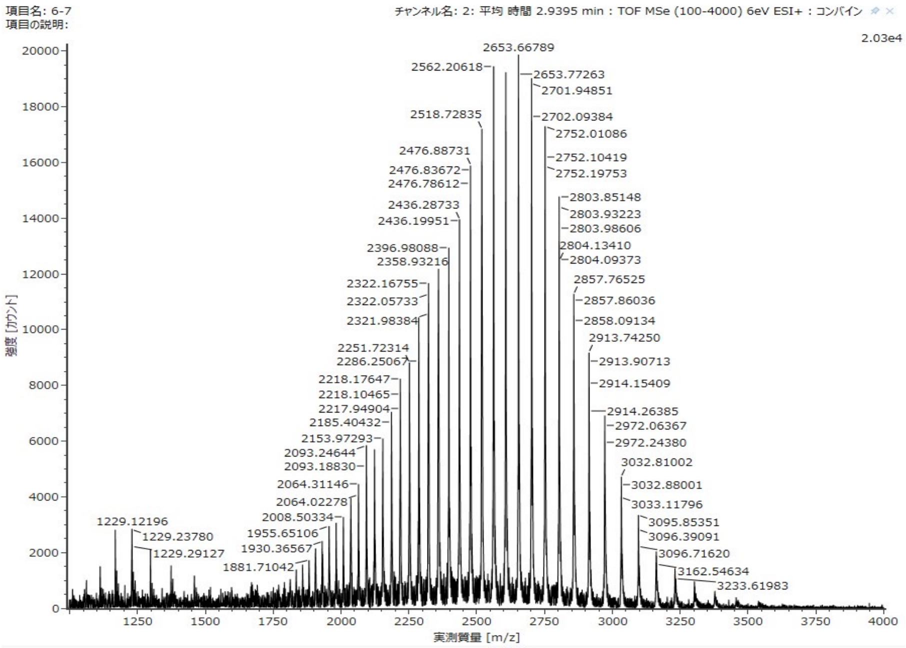

6-8 [G2-F/G1b-F]

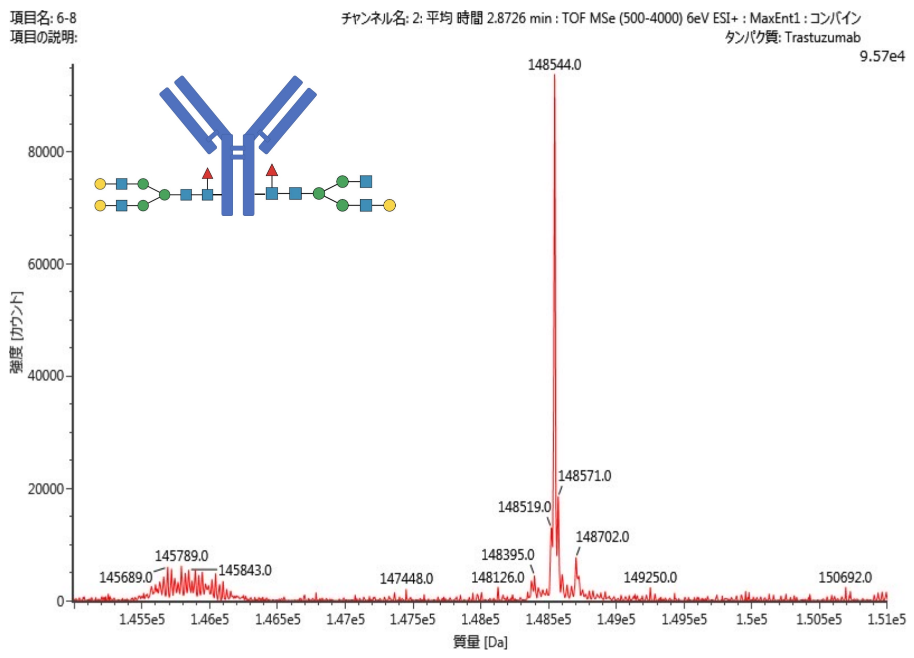

6-8 [G2-F/G1b-F]

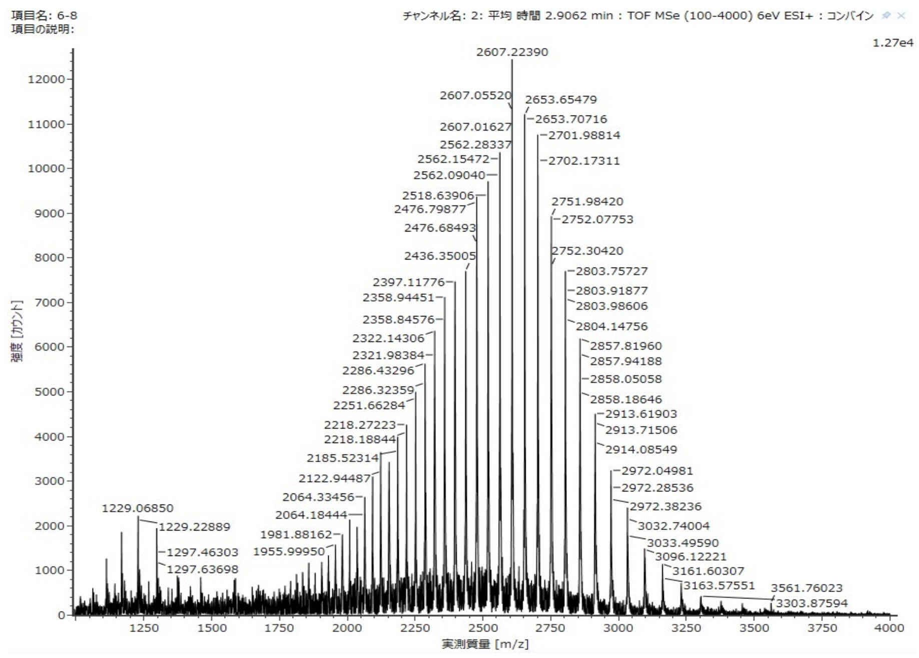

6-9 [G2-F/G0-F]

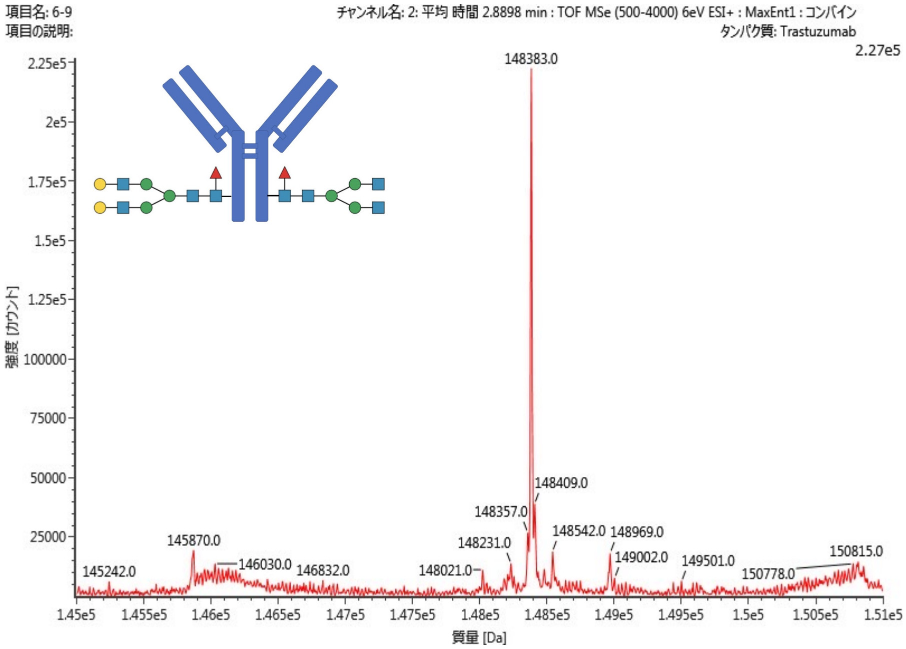

6-9 [G2-F/G0-F]

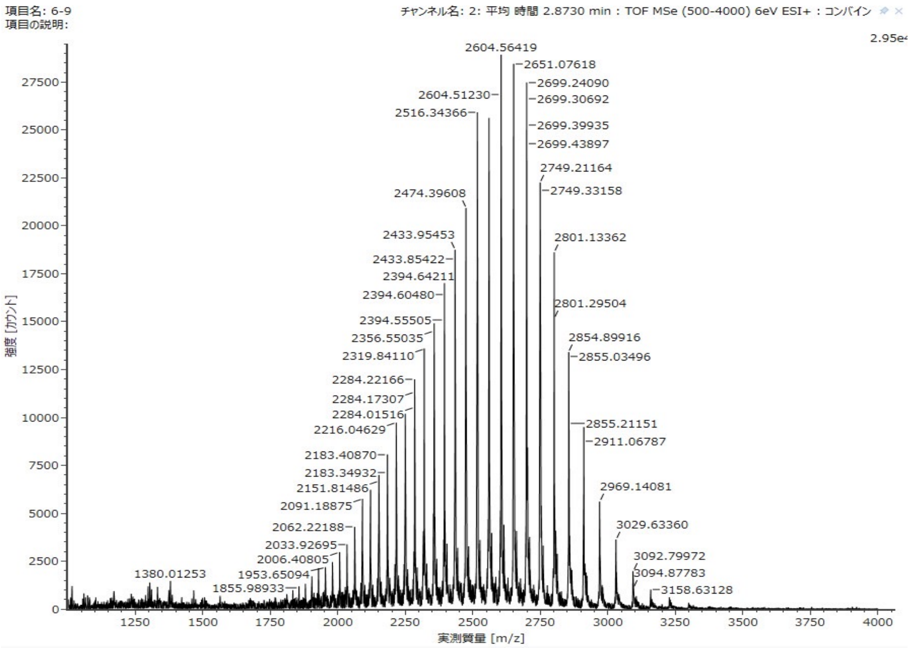

6-10 [G2-F/M3-F]

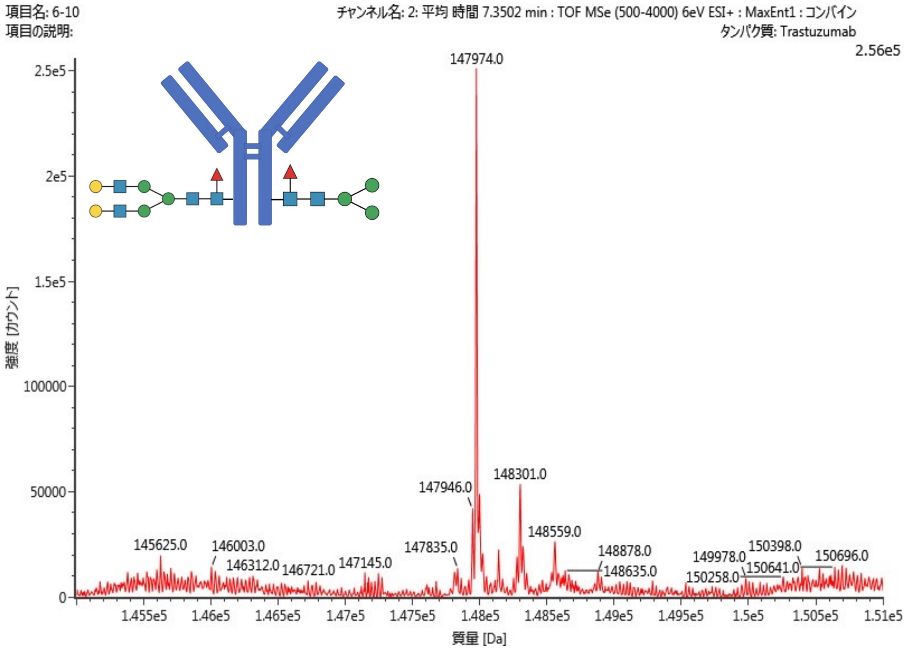

6-10 [G2-F/M3-F]

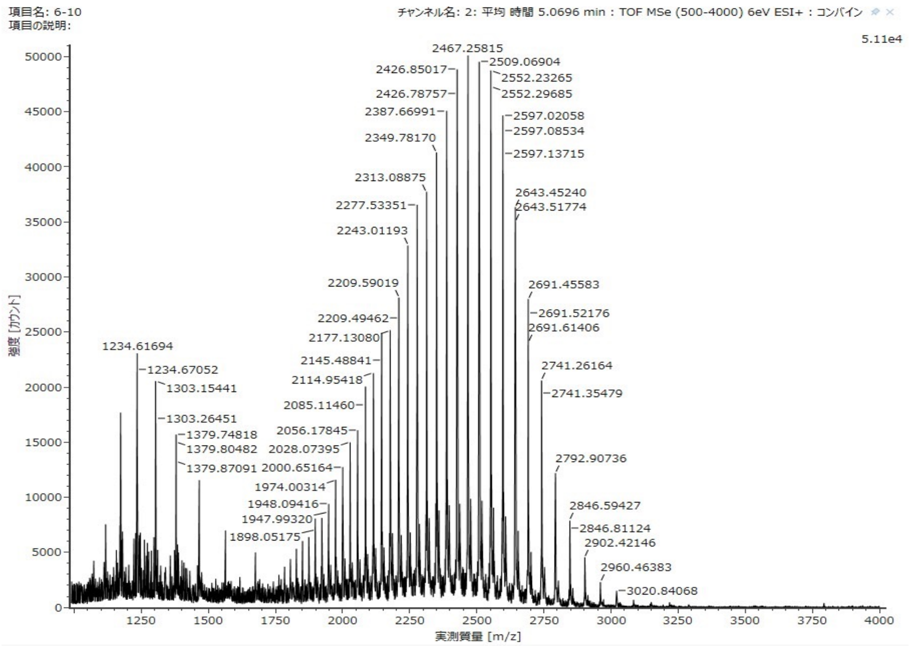

6-11 [G2-F/GlcNAc-F]

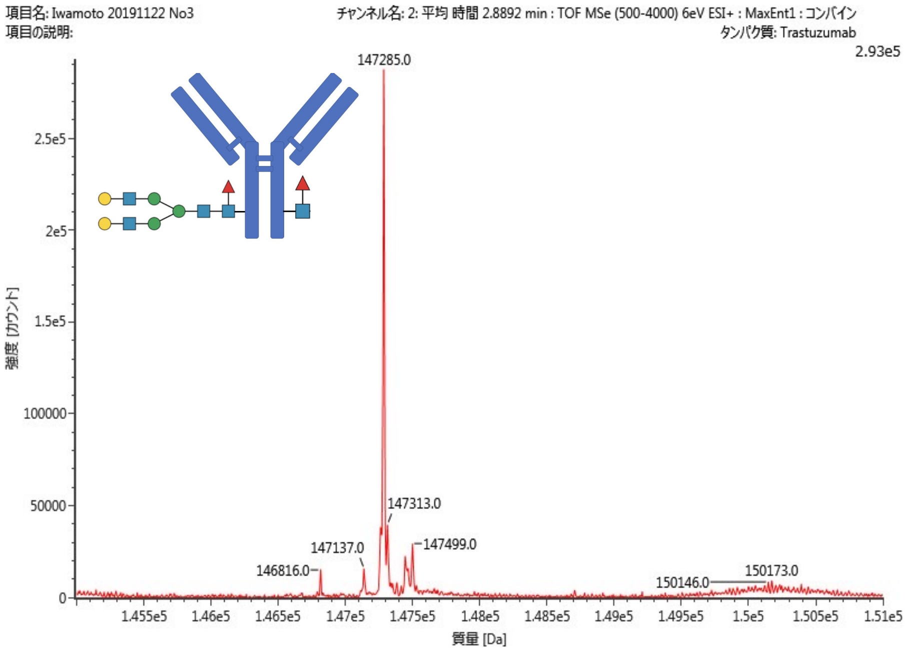

6-11 [G2-F/GlcNAc-F]

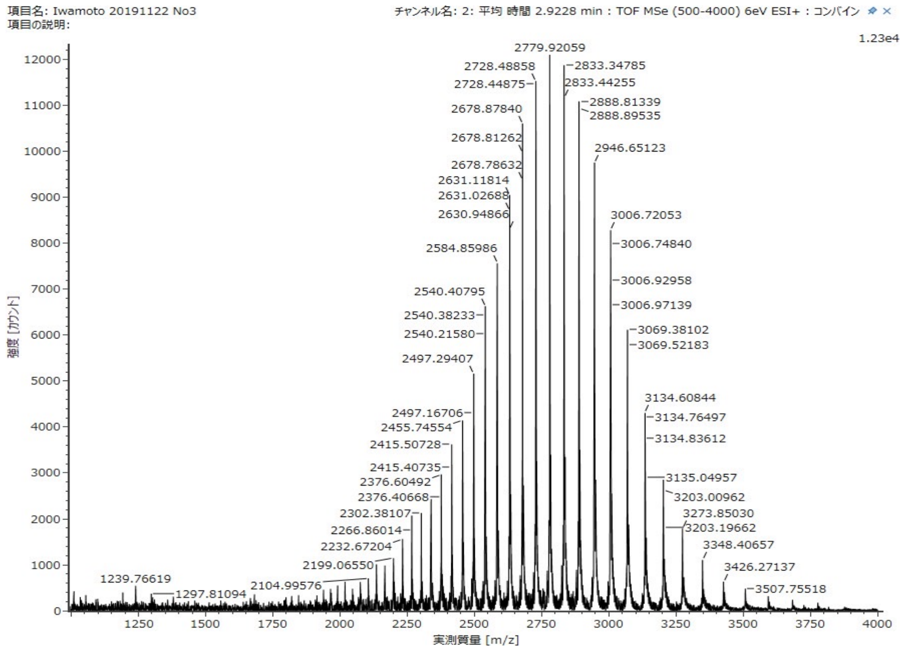

7-7 [G1a-F/G1a-F]

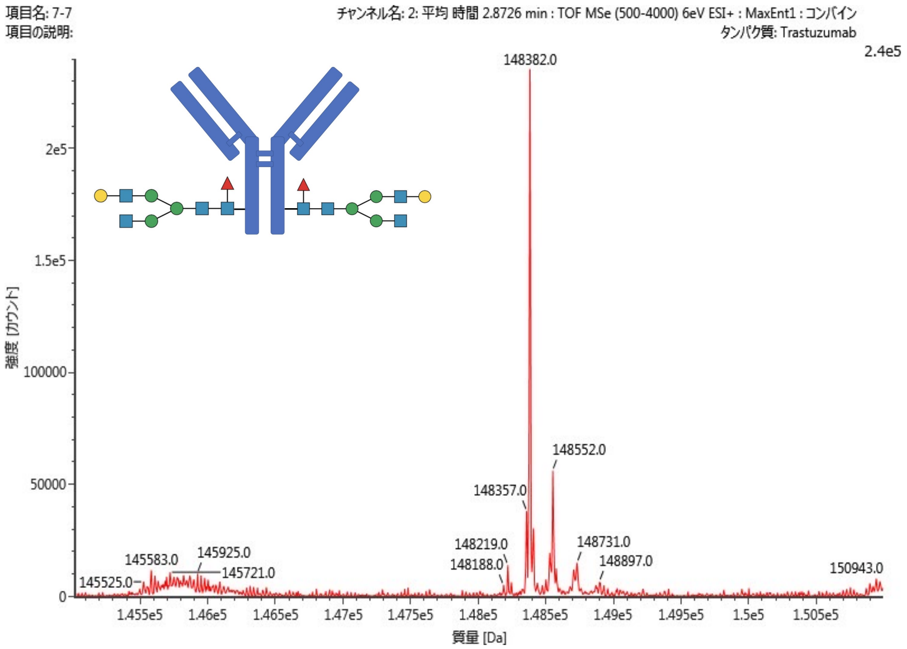

7-7 [G1a-F/G1a-F]

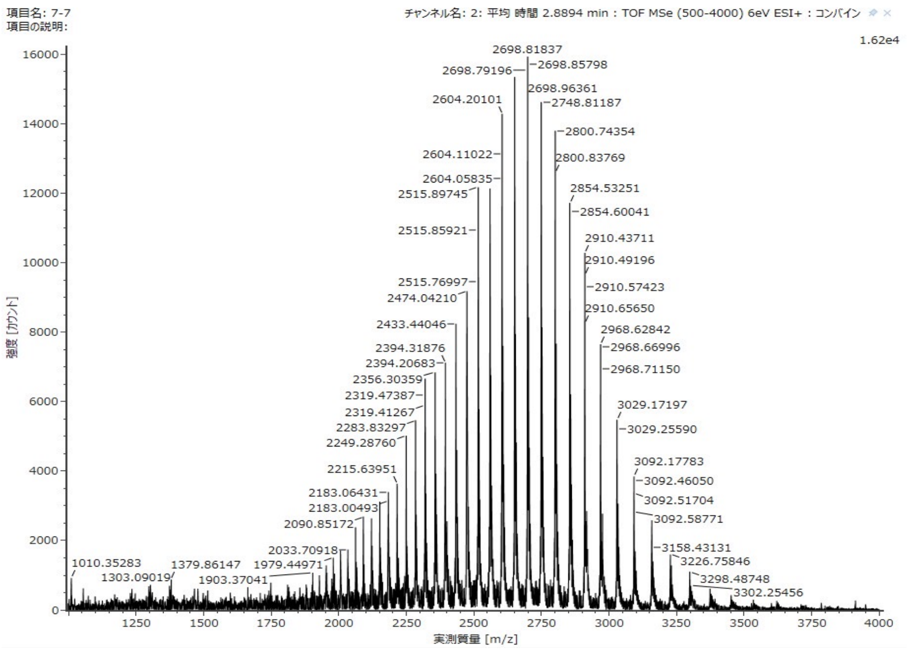

7-8 [G1a-F/G1b-F]

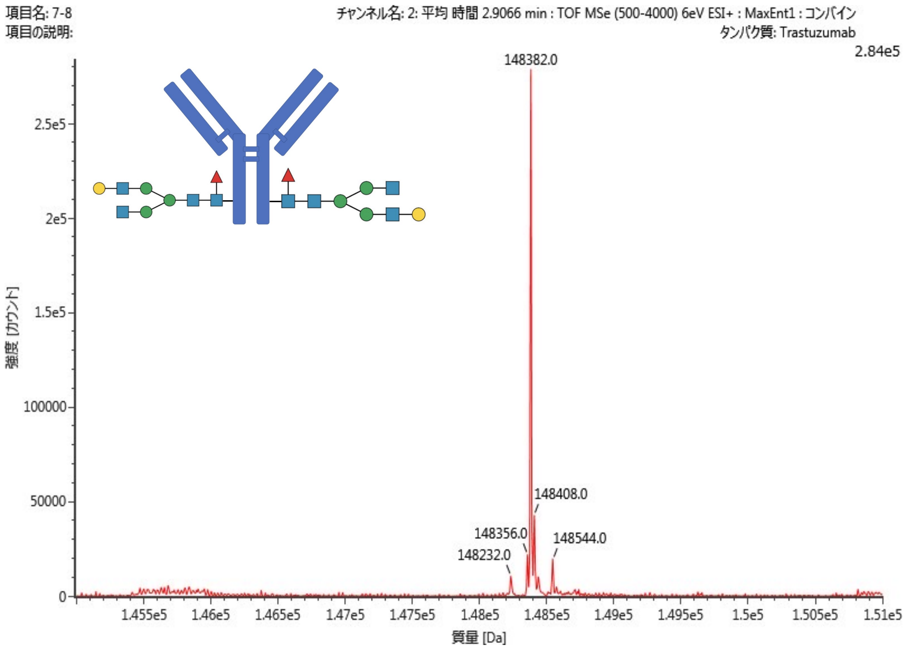

7-8 [G1a-F/G1b-F]

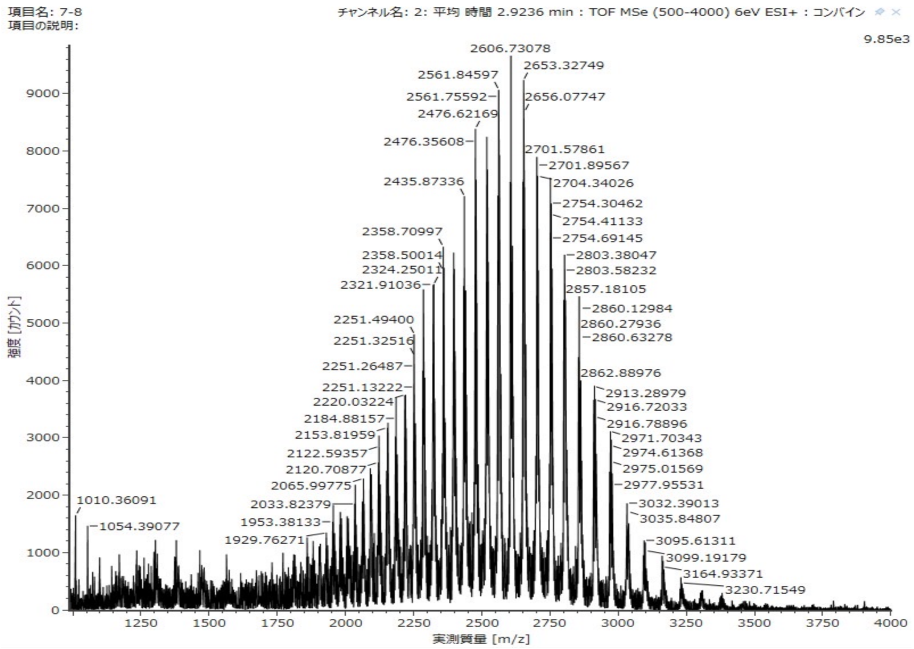

7-9 [G1a-F/G0-F]

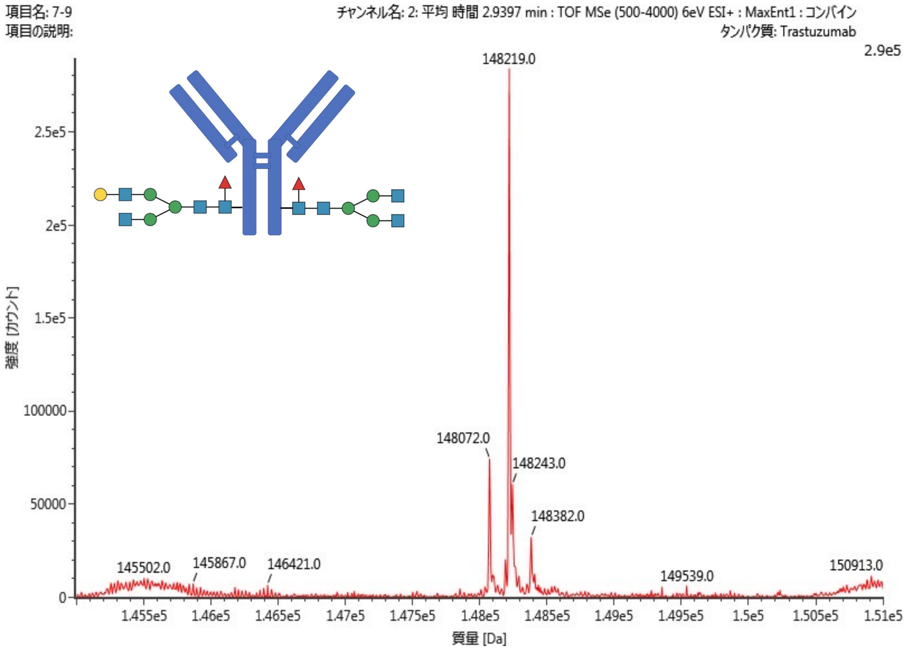

7-9 [G1a-F/G0-F]

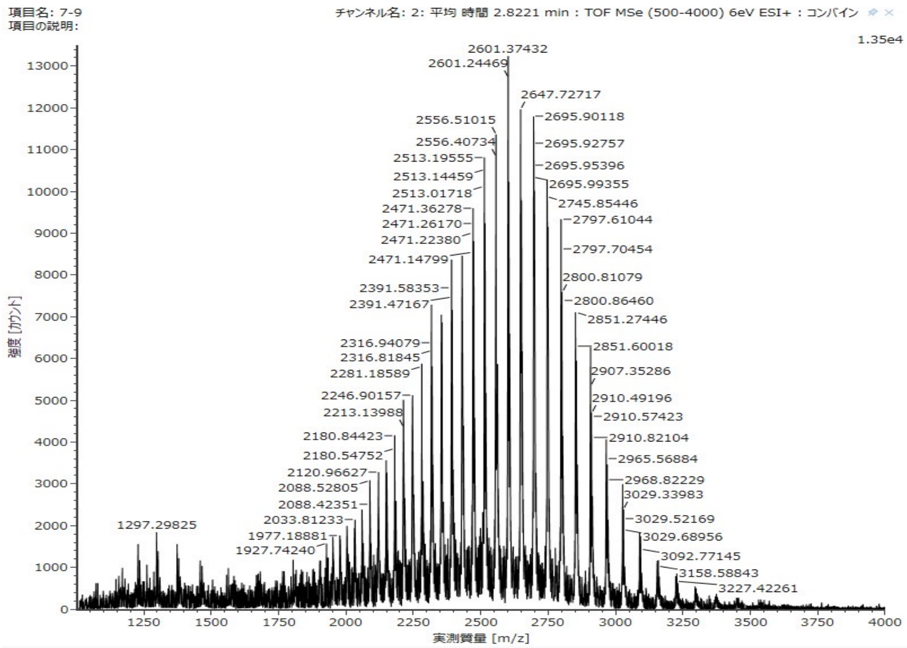

7-10 [G1a-F/M3-F]

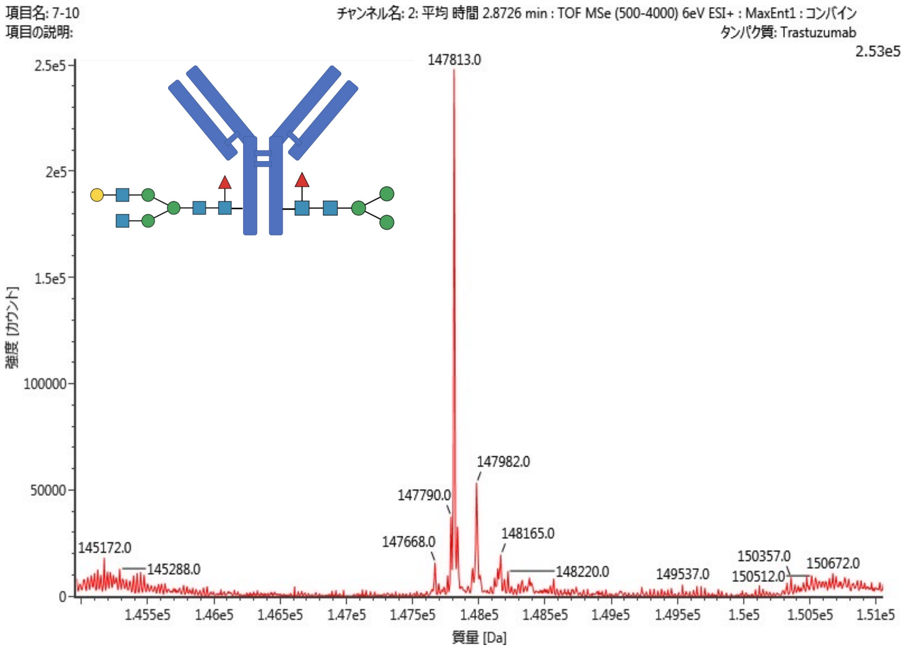

7-10 [G1a-F/M3-F]

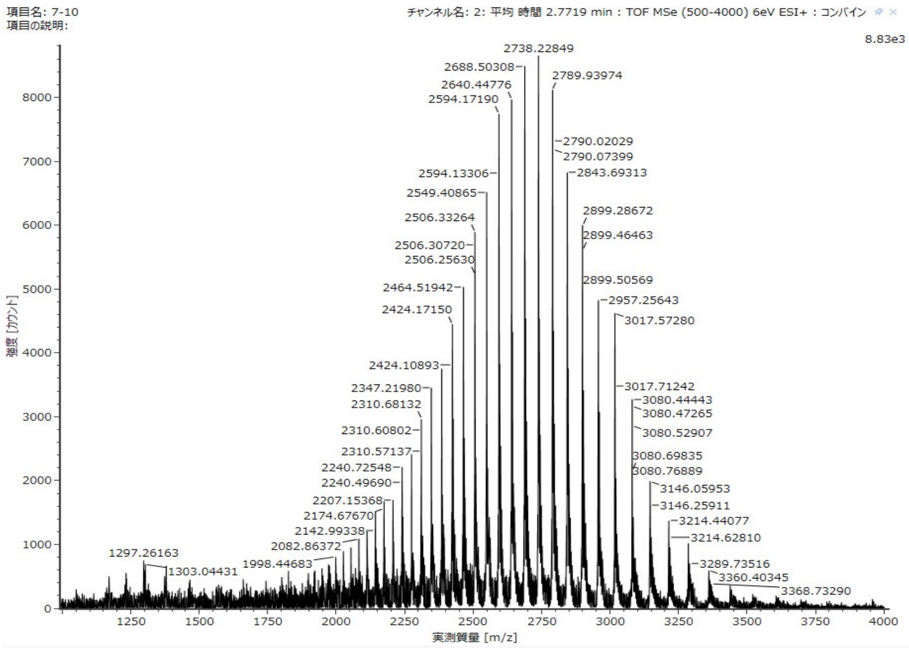

7-11 [G1a-F/GlcNAc-F]

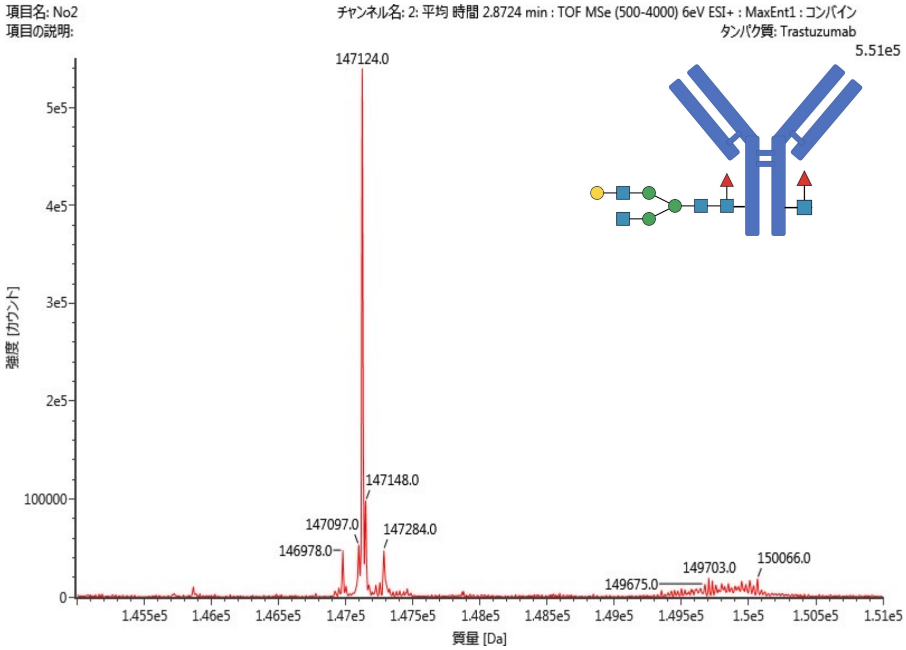

7-11 [G1a-F/GlcNAc-F]

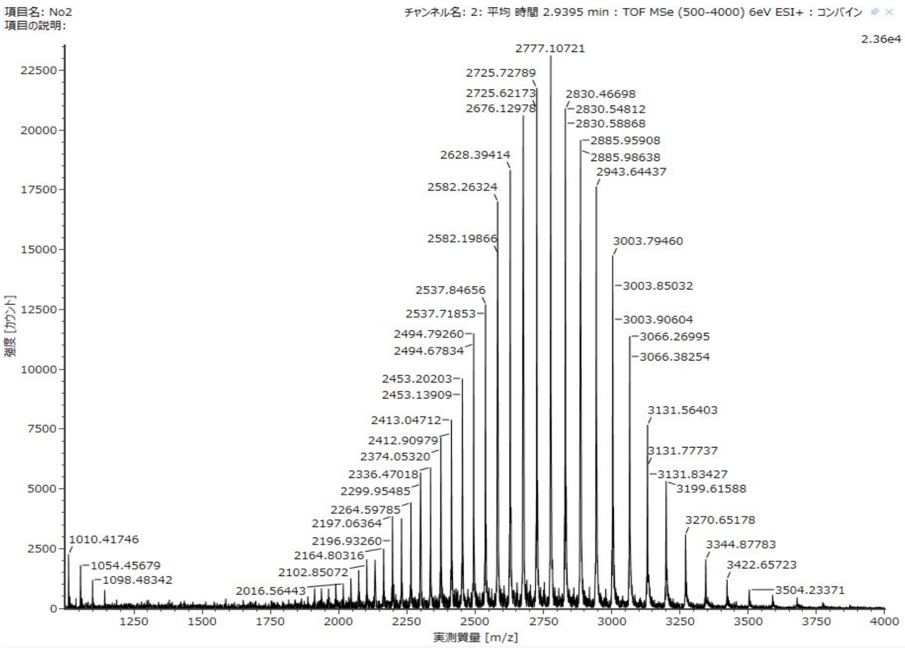

8-8 [G1b-F/G1b-F]

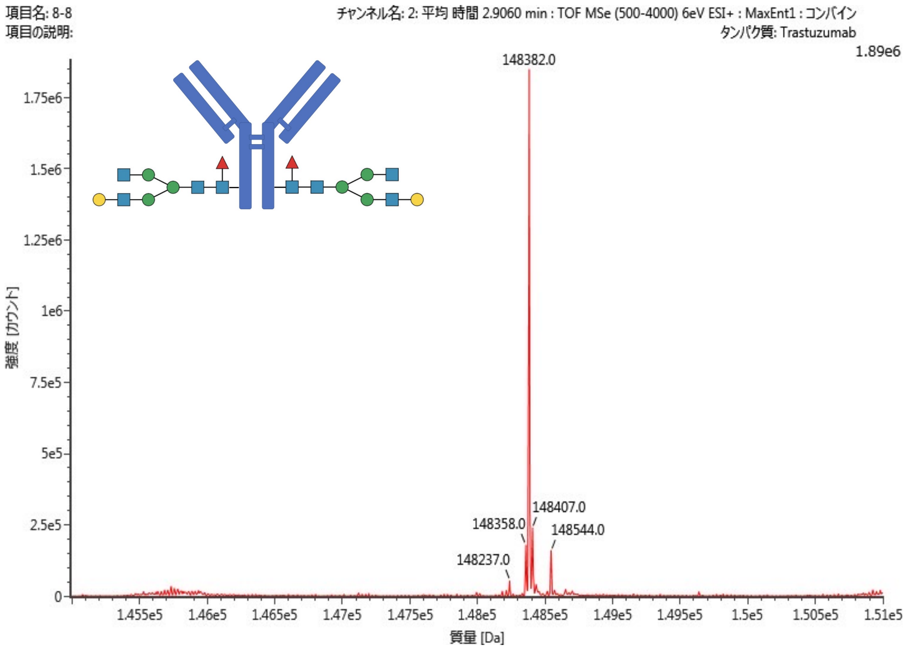

8-8 [G1b-F/G1b-F]

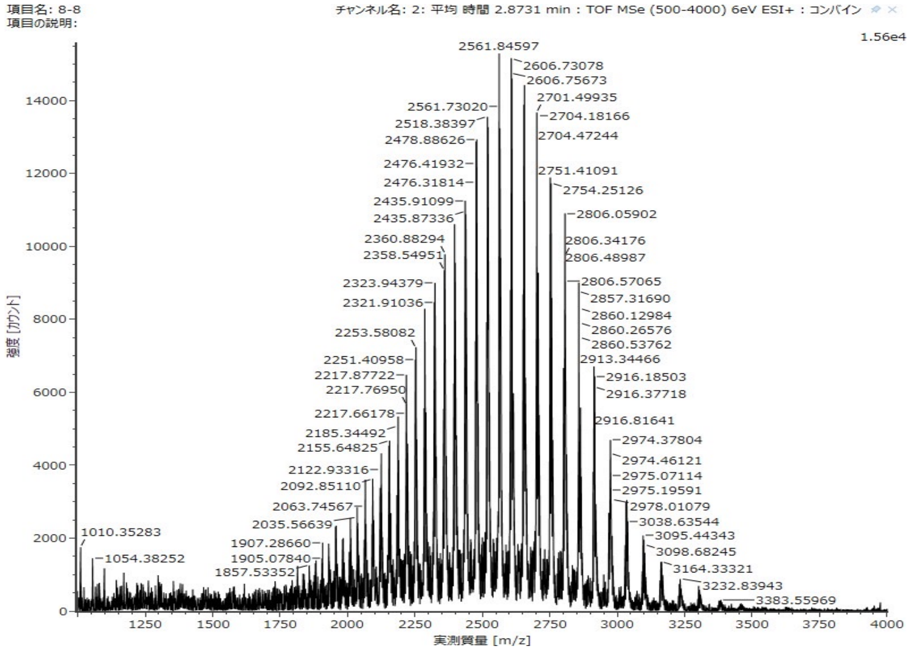

8-9 [G1b-F/G0-F]

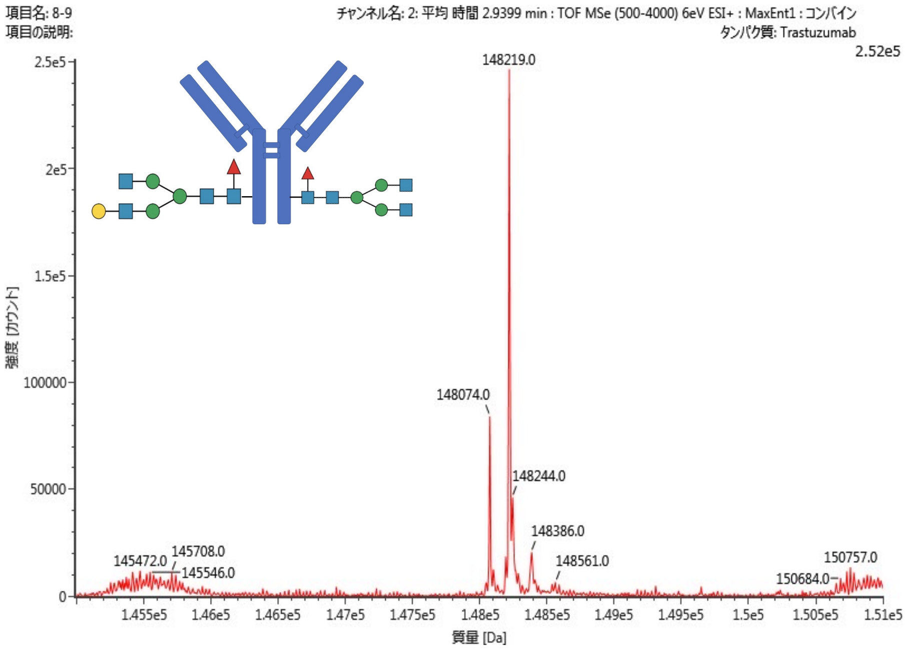

8-9 [G1b-F/G0-F]

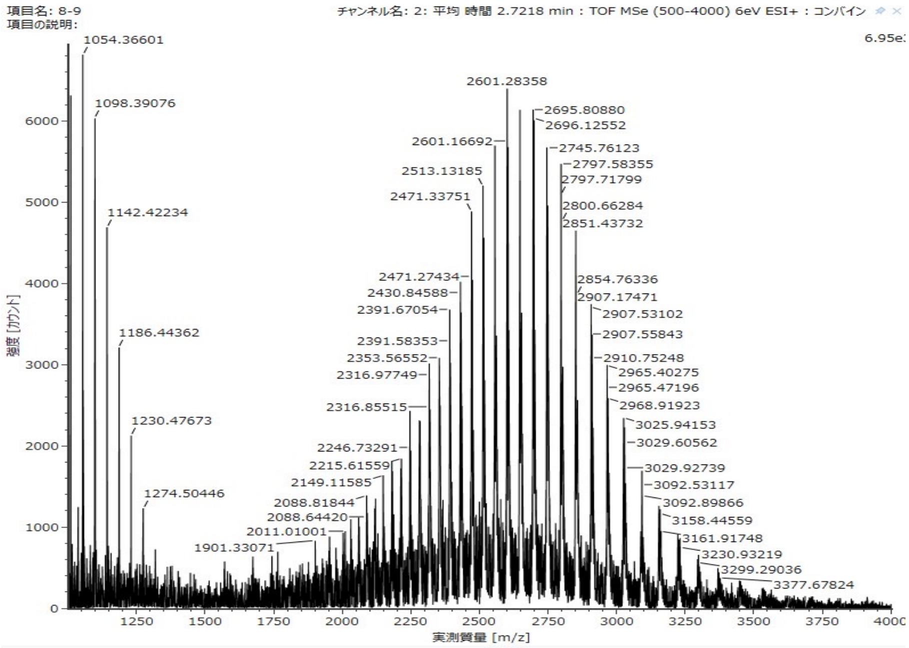

8-10 [G1b-F/M3-F]

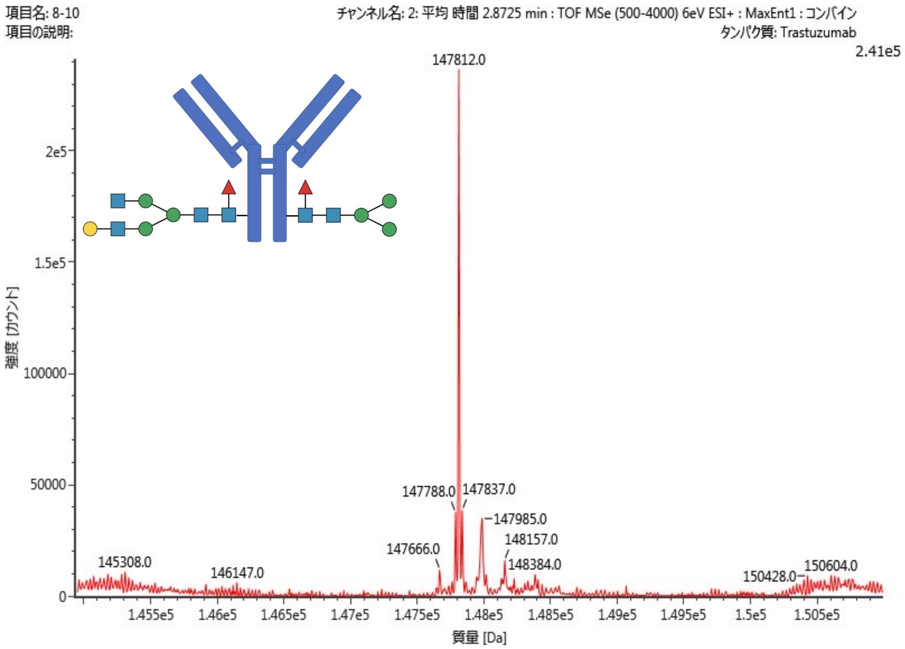

8-10 [G1b-F/M3-F]

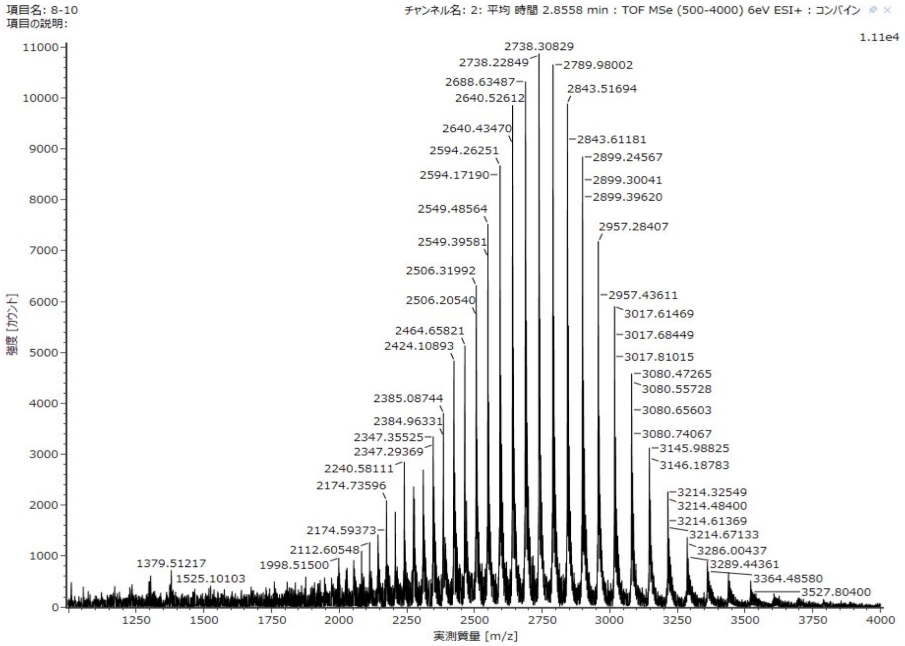

8-11 [G1b-F/GlcNAc-F]

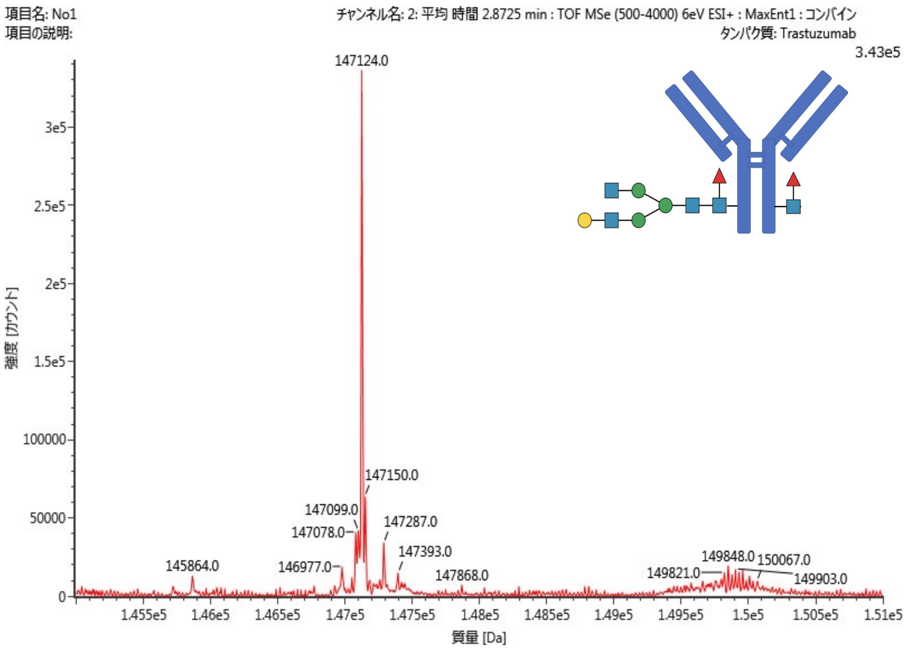

8-11 [G1b-F/GlcNAc-F]

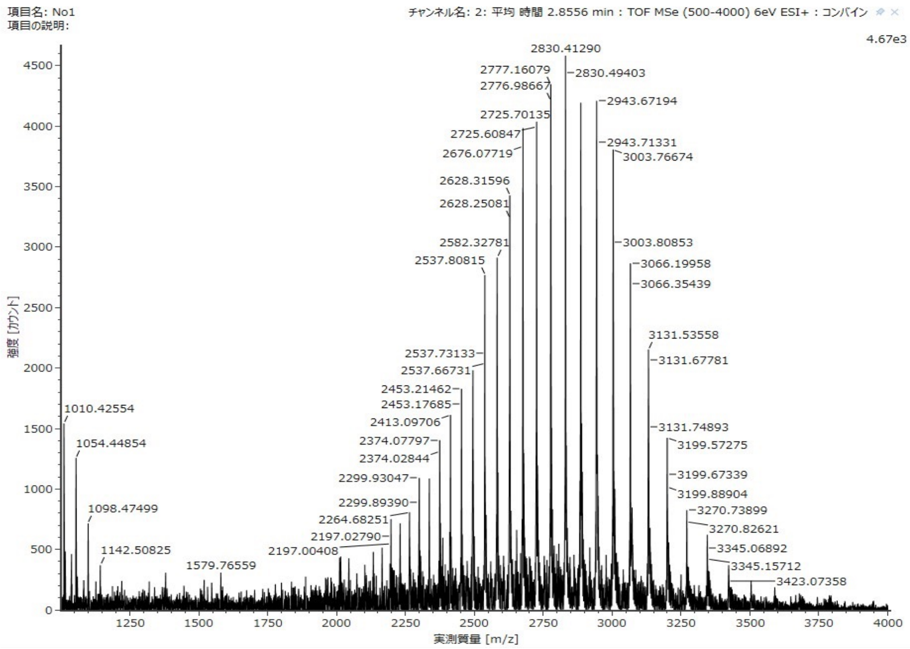

9-9 [G0-F/G0-F]

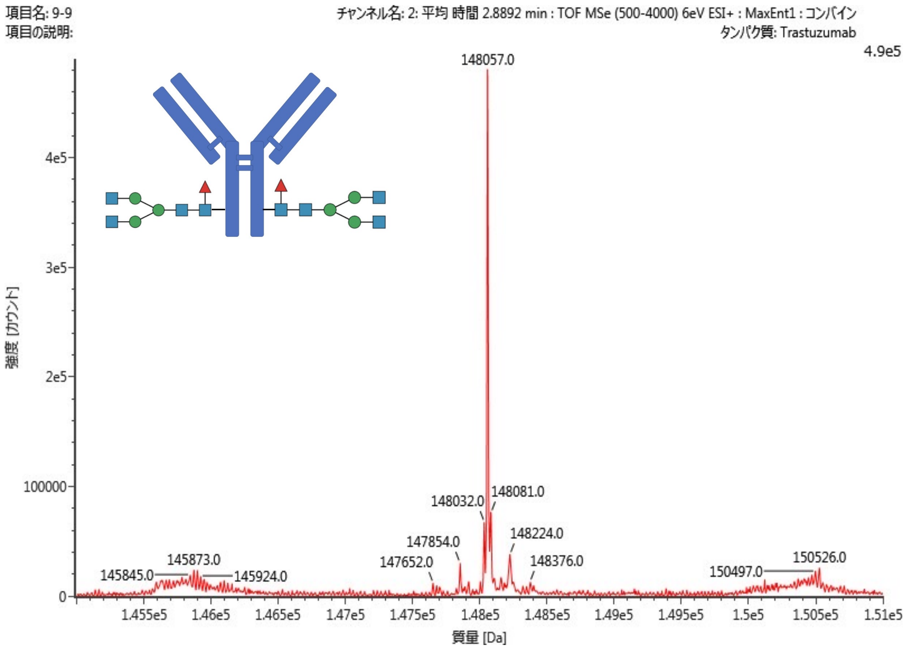

9-9 [G0-F/G0-F]

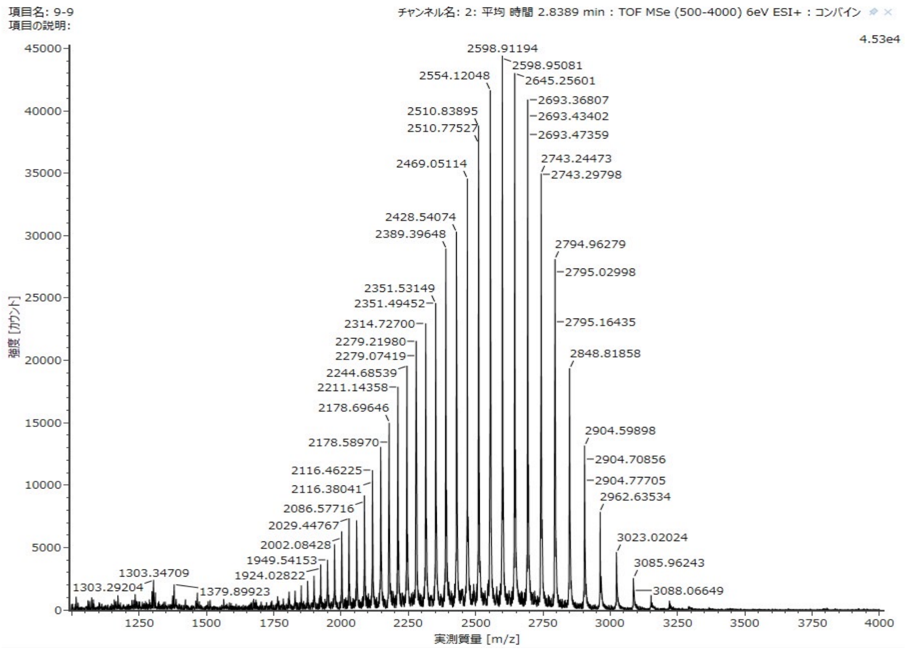

9-10 [G0-F/M3-F]

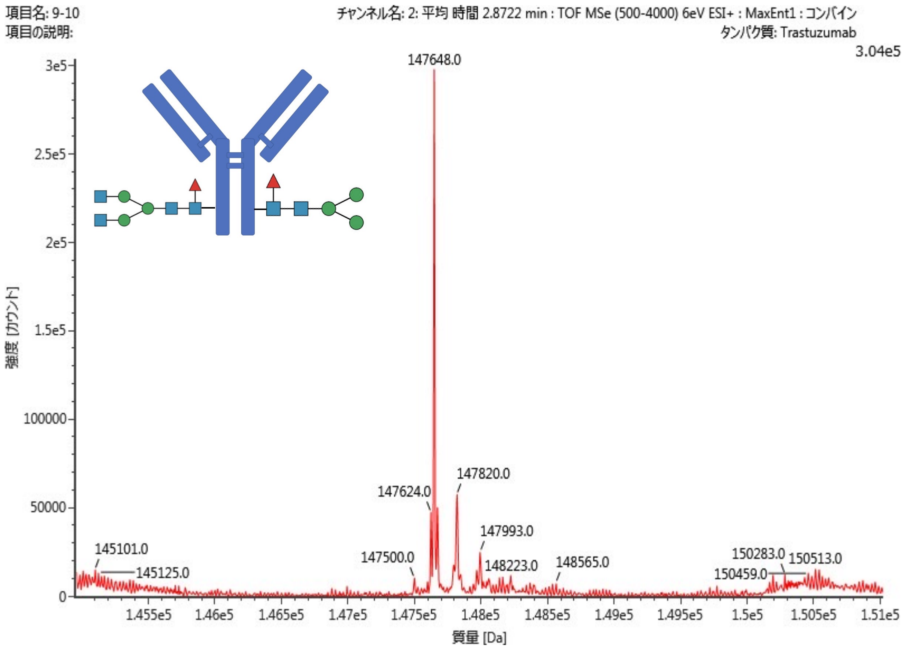

9-10 [G0-F/M3-F]

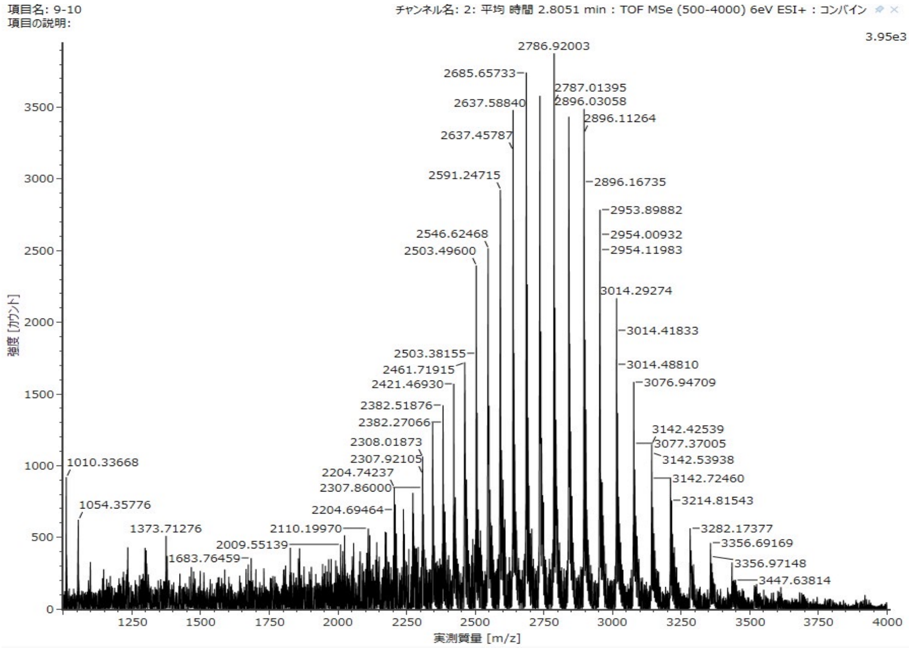

9-11 [G0-F/GlcNAc-F]

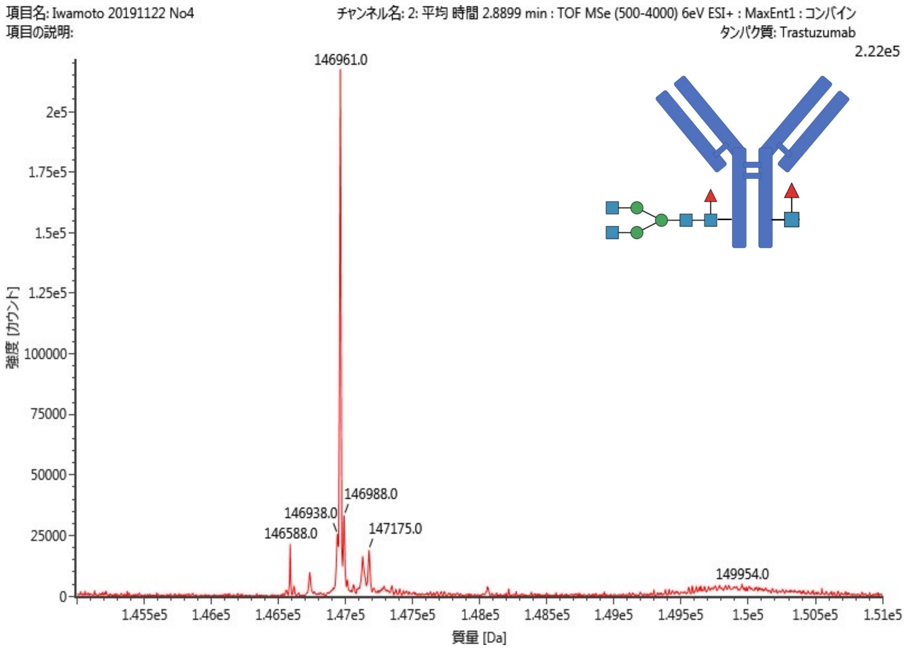

9-11 [G0-F/GlcNAc-F]

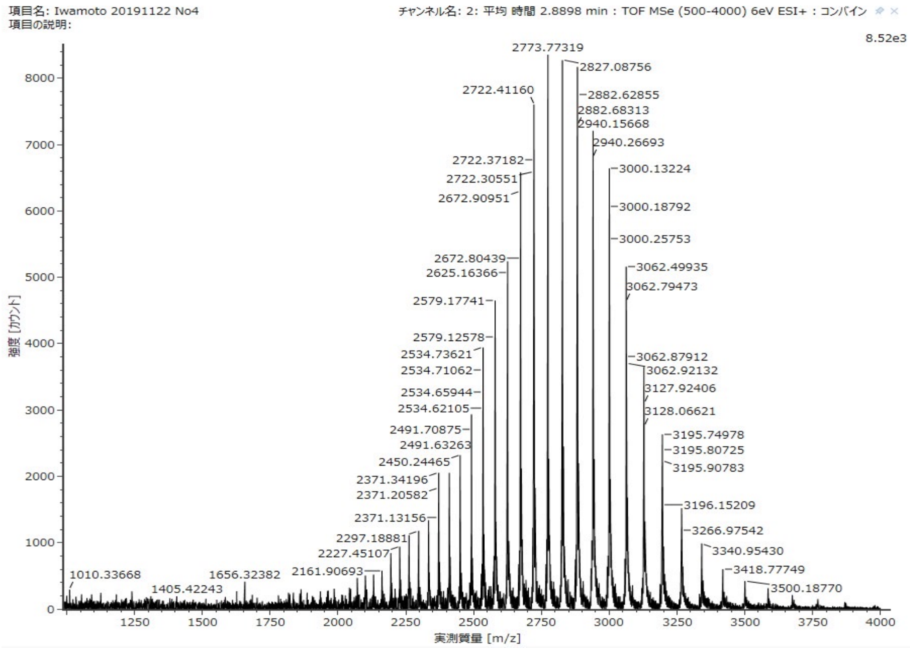

10-10 [M3-F/M3-F]

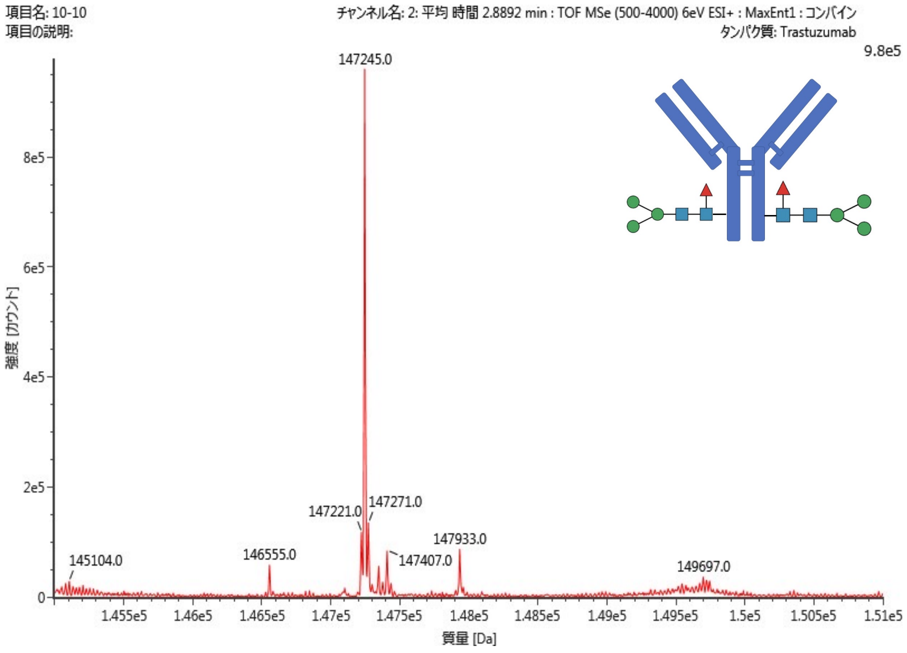

10-10 [M3-F/M3-F]

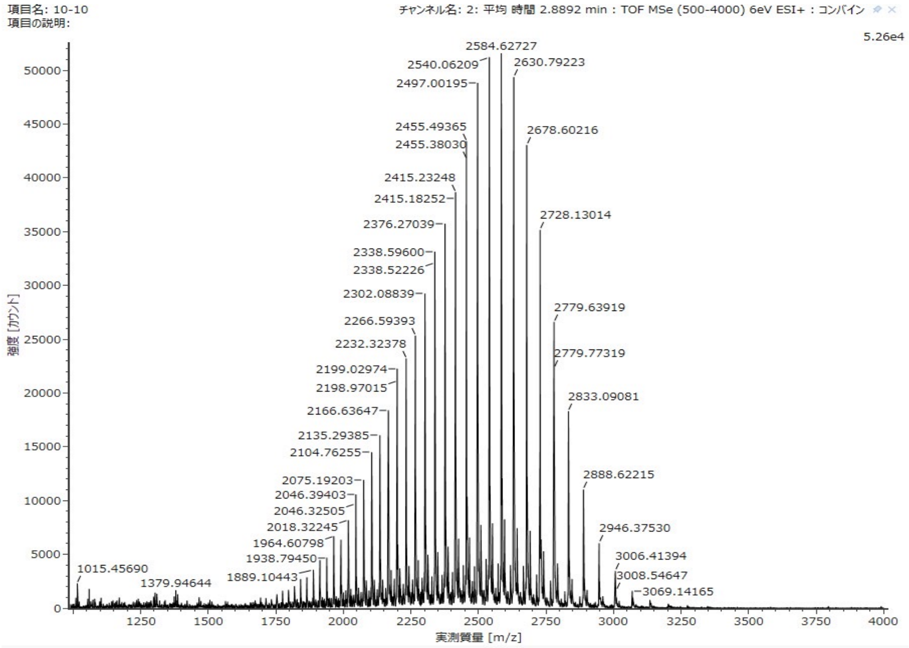

10-11 [M3-F/GlcNAc-F]

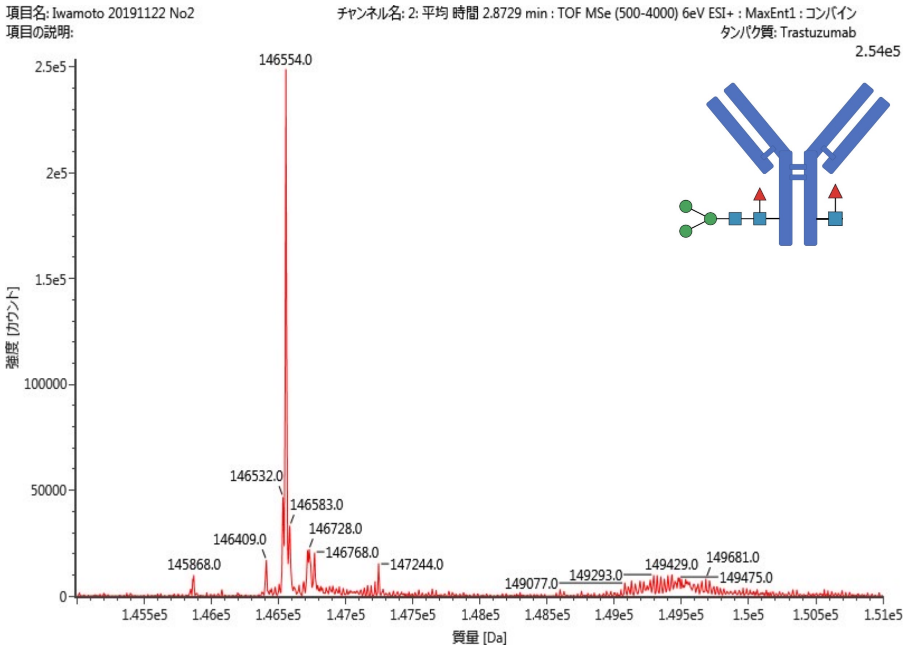

10-11 [M3-F/GlcNAc-F]

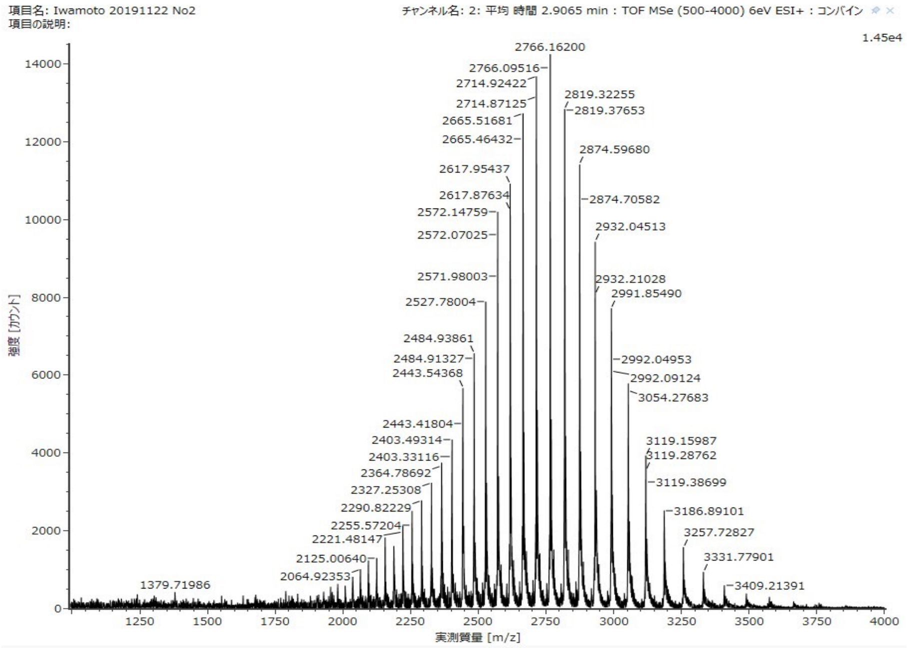

11-11 [GlcNAc-F/GlcNAc-F]

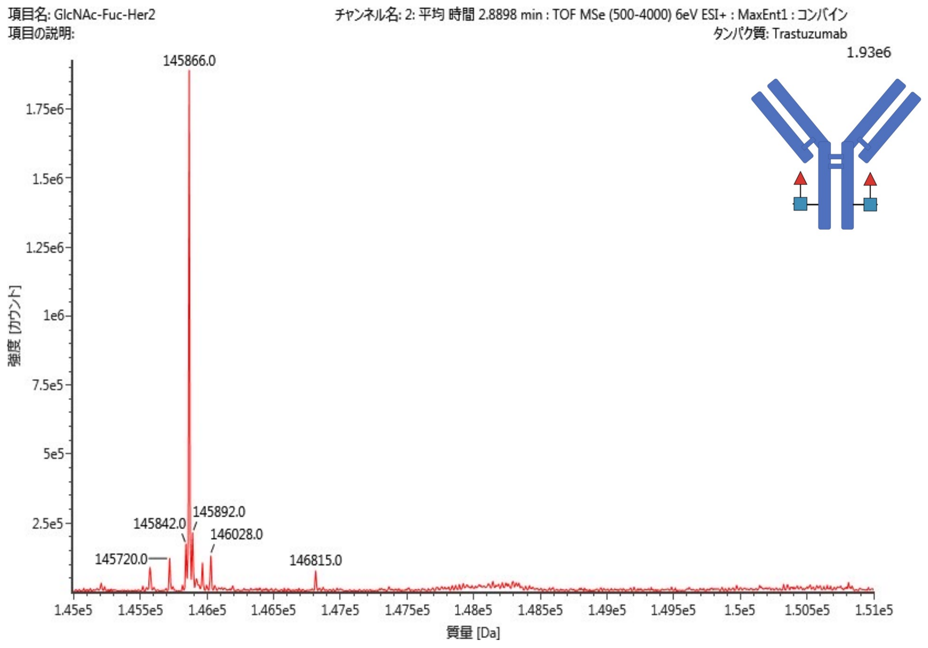

11-11 [GlcNAc-F/GlcNAc-F]

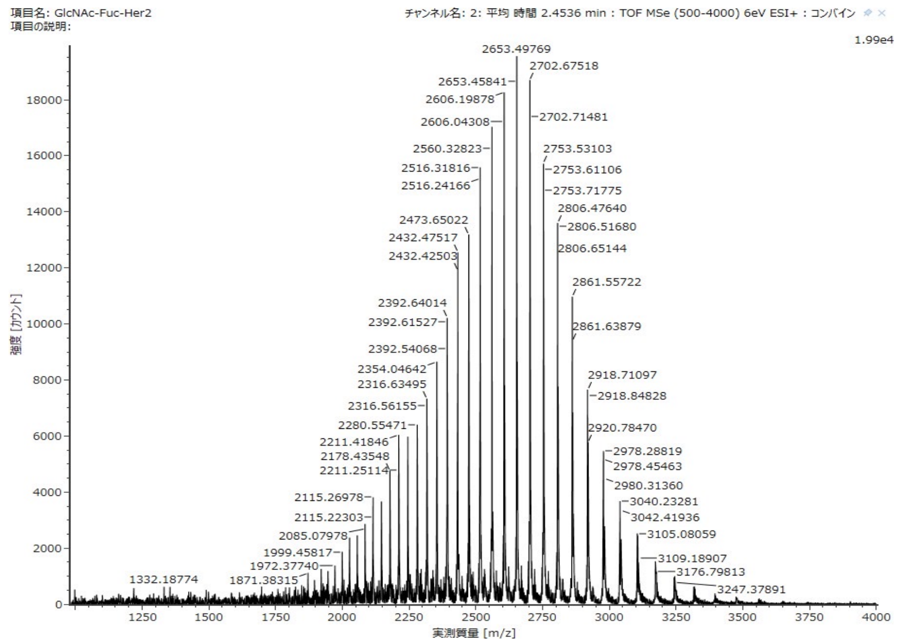

Figure S6.

DSC measurement of trastuzumab with homogeneous glycan.

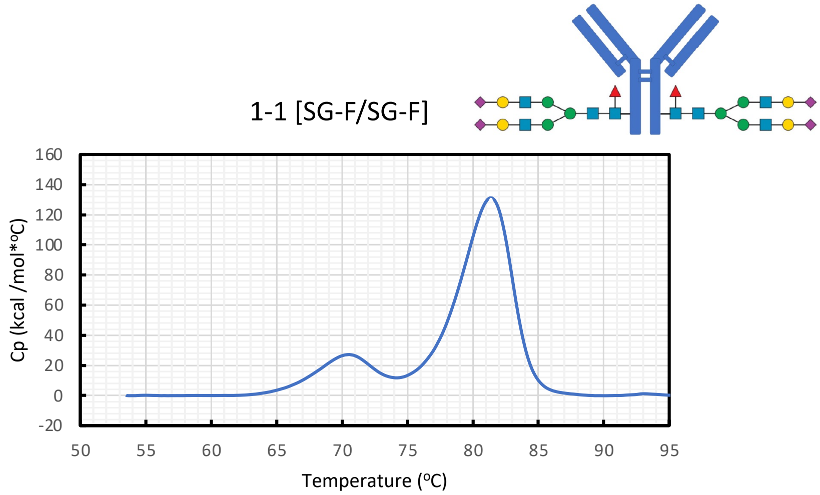

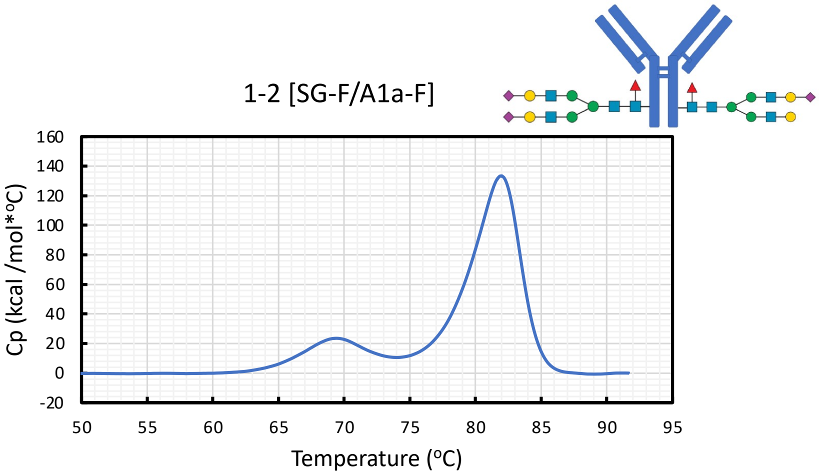

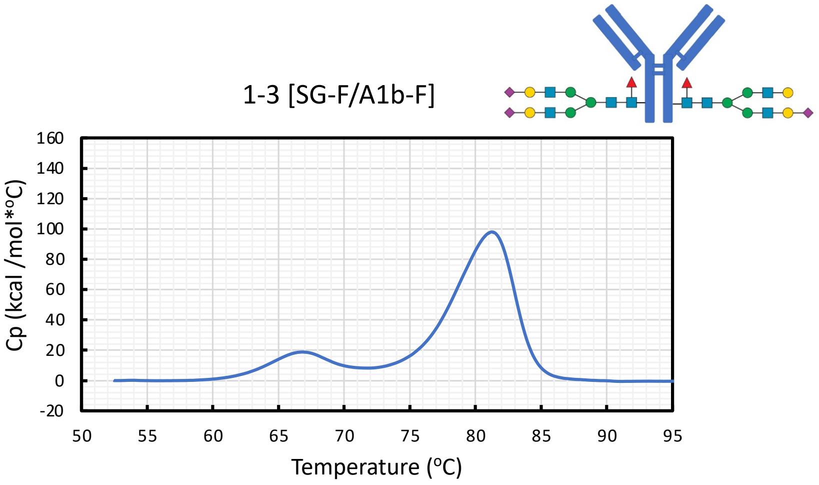

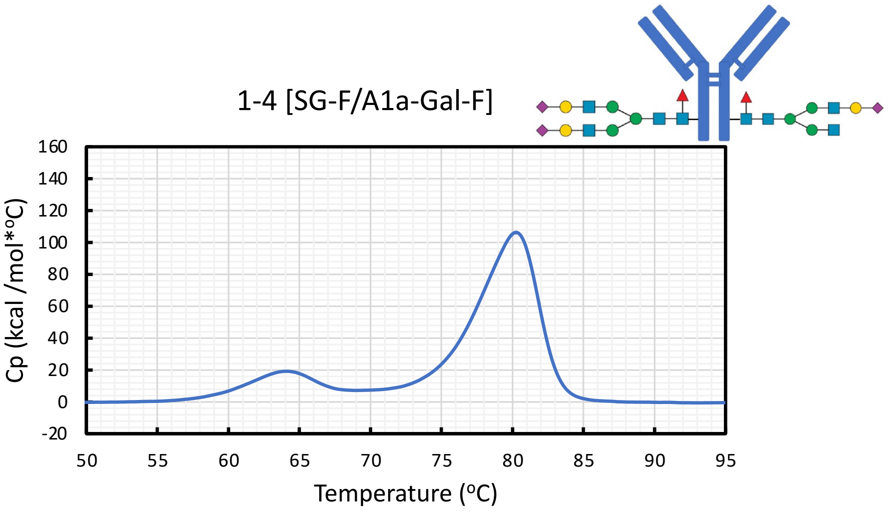
1-4 [SG-F/A1a-Gal-F]
Cp (kcal /mol*ºC)
Temperature (ºC)
160
140
120
100
80
60
40
20
0
-20
50
55
60
65
70
75
80
85
90
95

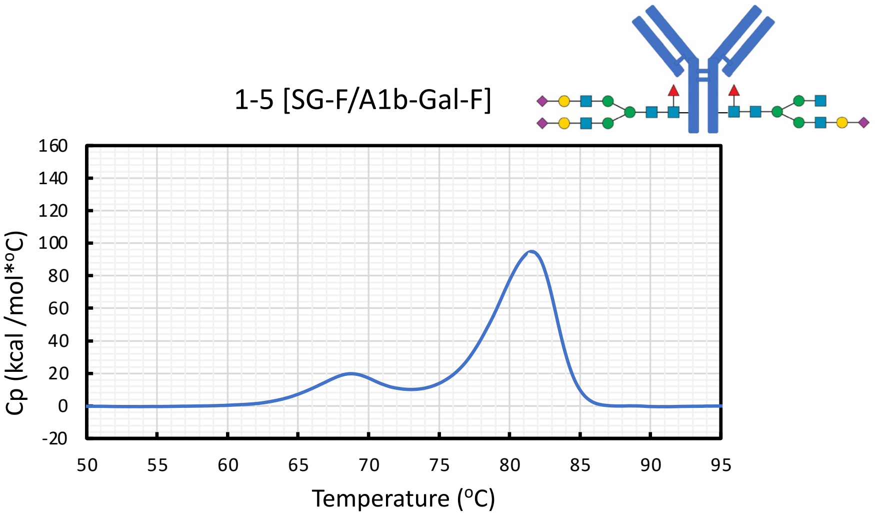
1-5 [SG-F/A1b-Gal-F]
Cp (kcal /mol*ºC)
Temperature (ºC)
160
140
120
100
80
60
40
20
0
-20
50
55
60
65
70
75
80
85
90
95

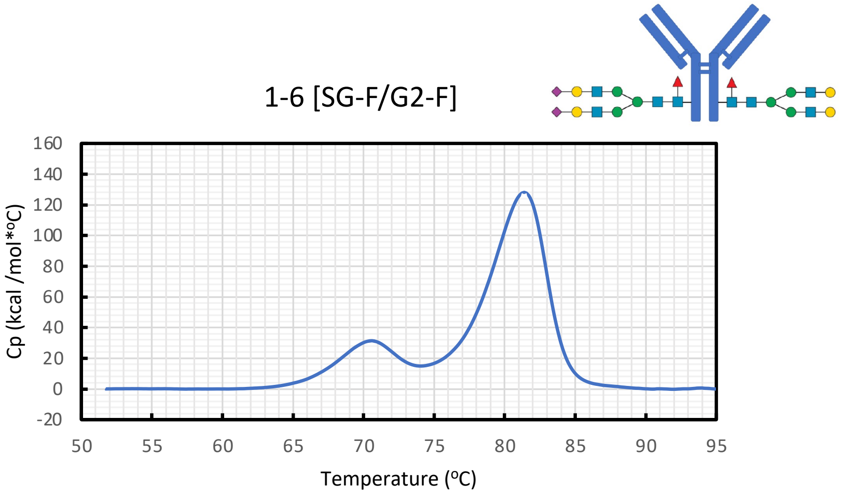
1-6 [SG-F/G2-F]
Cp (kcal /mol*ºC)
Temperature (ºC)
160
140
120
100
80
60
40
20
0
-20
50
55
60
65
70
75
80
85
90
95

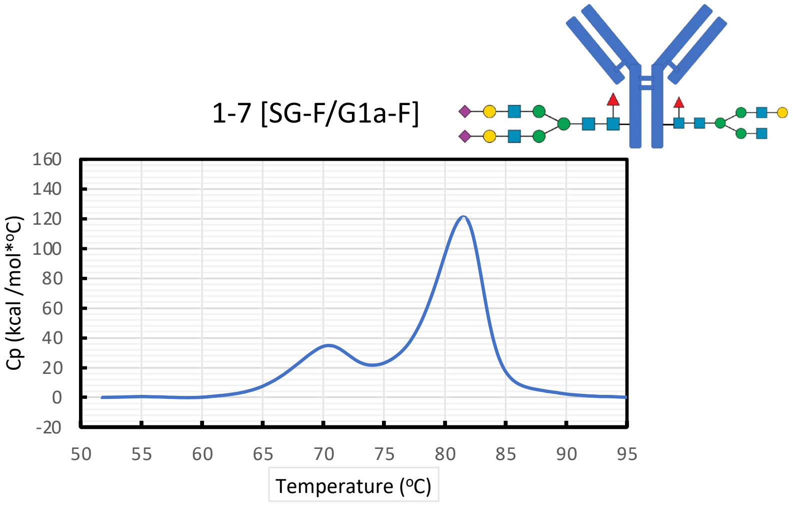
1-7 [SG-F/G1a-F]
Cp (kcal /mol*ºC)
Temperature (ºC)

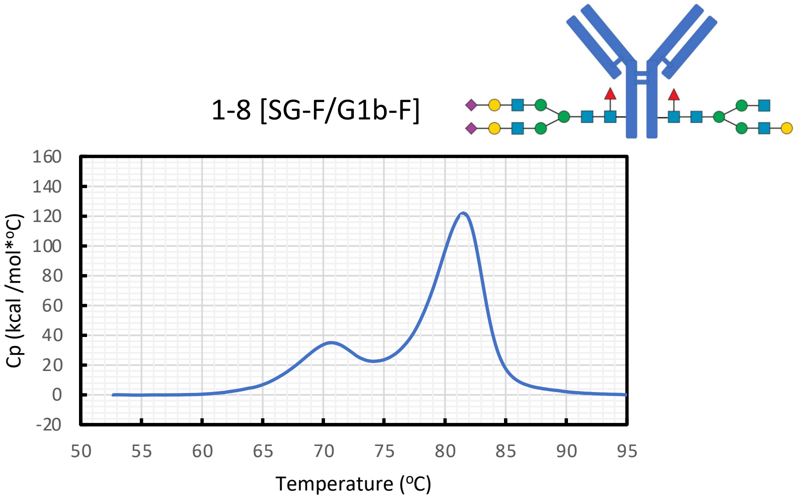
1-8 [SG-F/G1b-F]
Cp (kcal /mol*ºC)
Temperature (ºC)

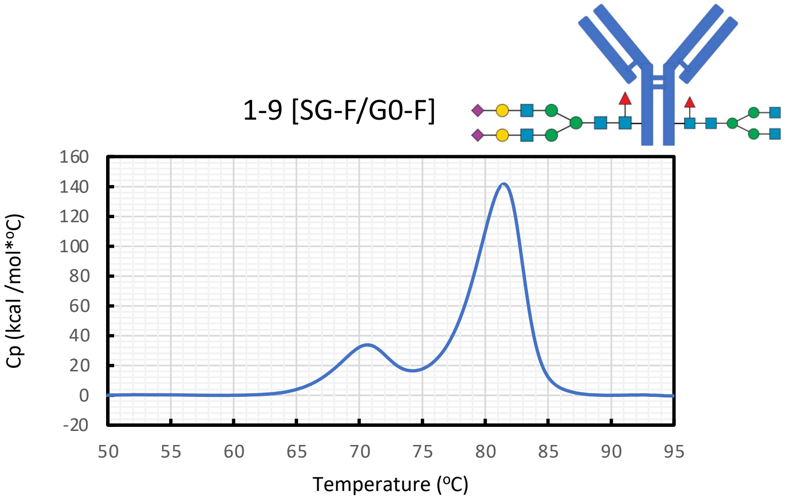
1-9 [SG-F/G0-F]
Cp (kcal /mol*ºC)
Temperature (ºC)

1-10 [SG-F/M3-F]

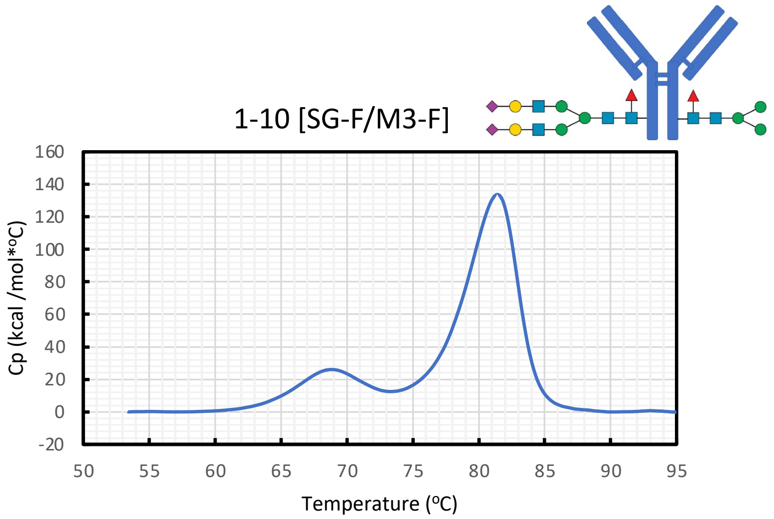

1-11 [SG-F/GlcNAc-F]

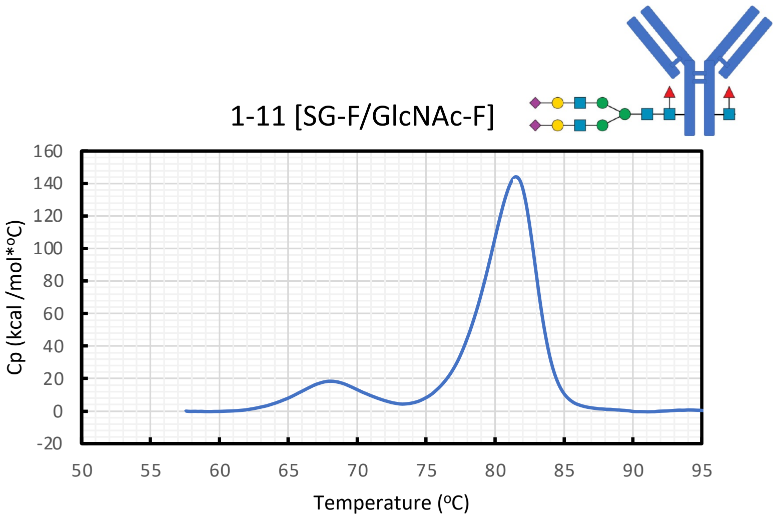

2-2 [A1a-F/A1a-F]

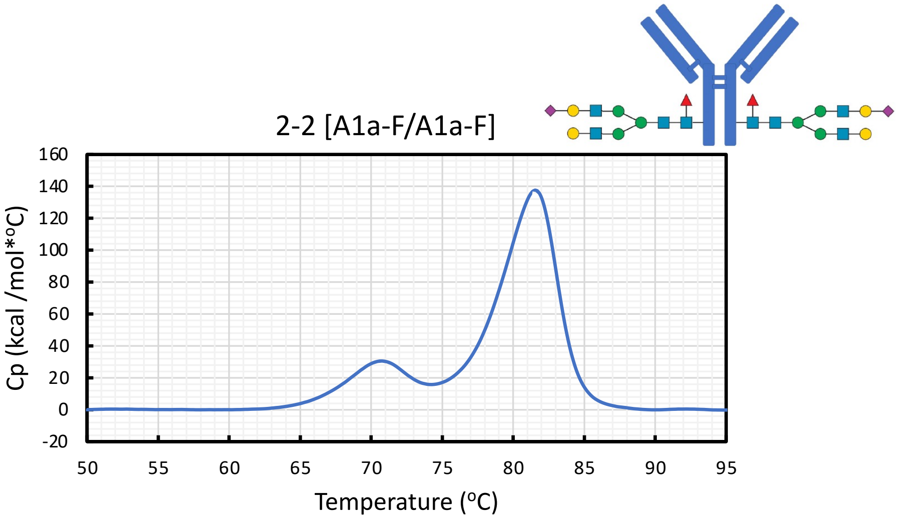

2-3 [A1a-F/A1b-F]

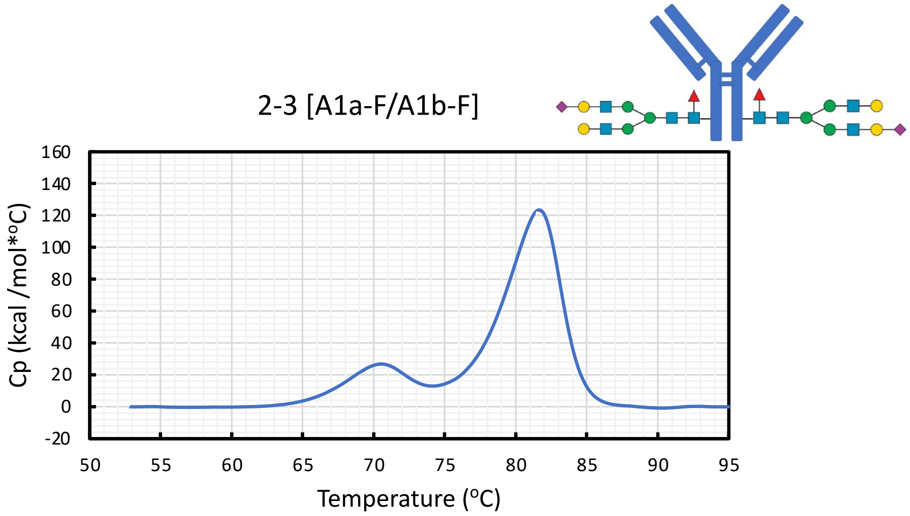

2-4 [A1a-F/A1a-Gal-F]

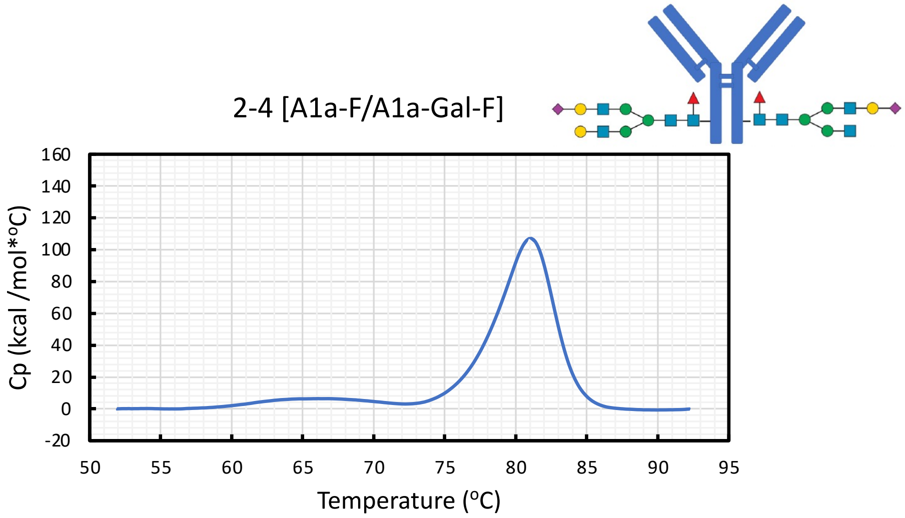

2-5 [A1a-F/A1b-Gal-F]

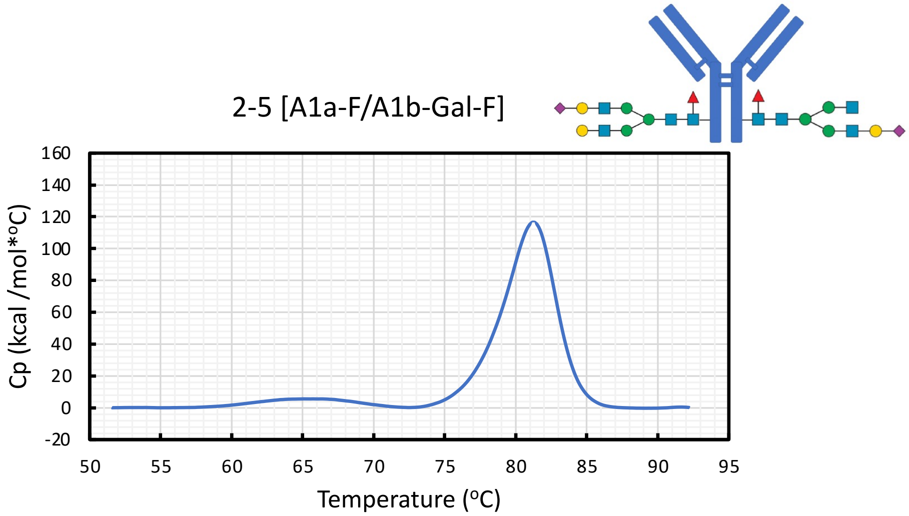

2-6 [A1a-F/G2-F]

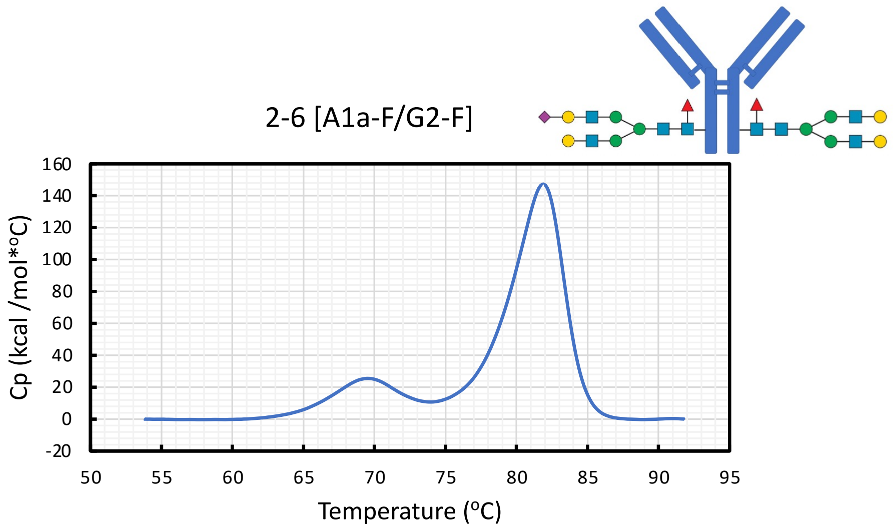

2-7 [A1a-F/G1a-F]

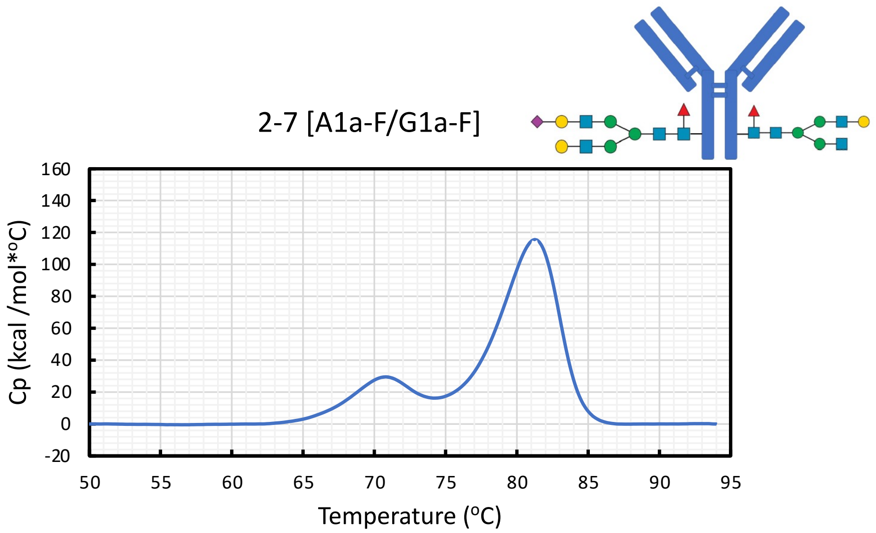

2-8 [A1a-F/G1b-F]

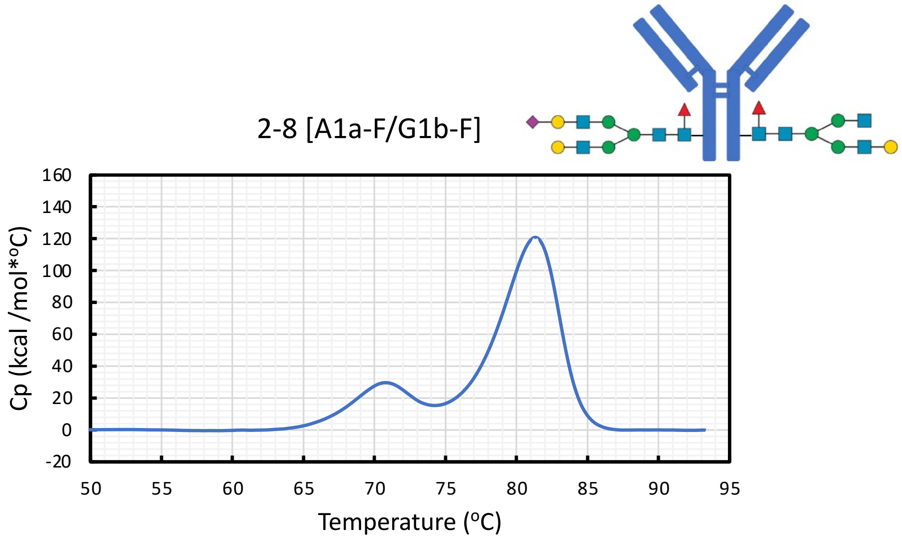

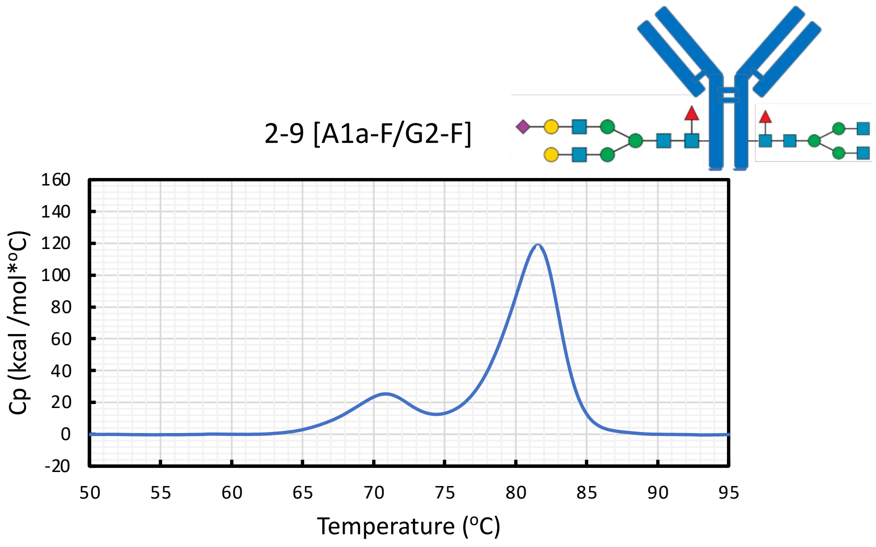
2-9 [A1a-F/G2-F]
Cp (kcal /mol*°C)
Temperature (°C)

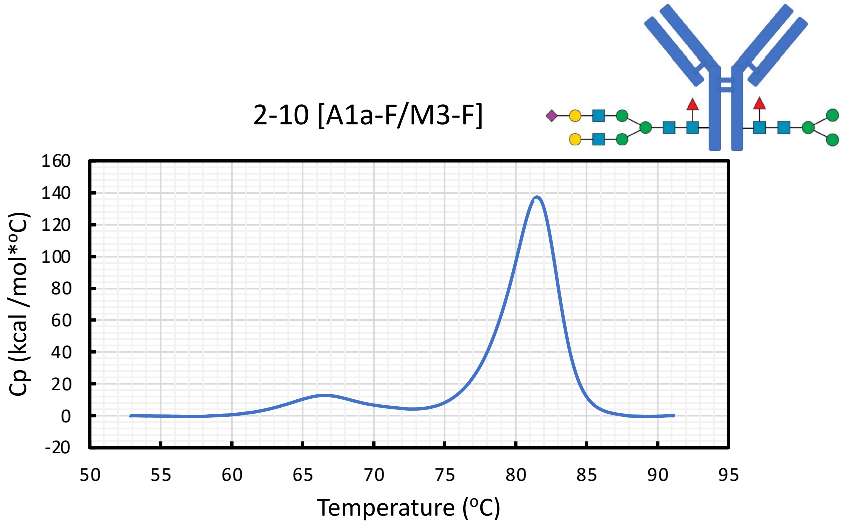
2-10 [A1a-F/M3-F]
Cp (kcal /mol*°C)
Temperature (°C)

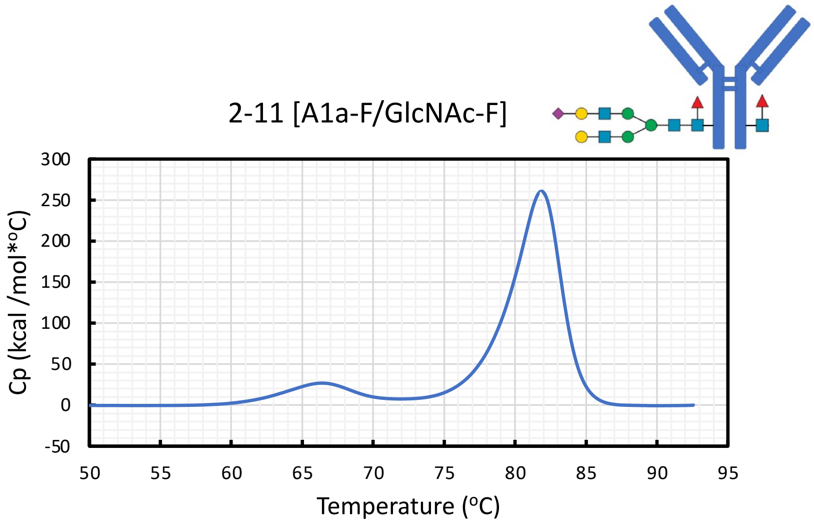
2-11 [A1a-F/GlcNAc-F]
Cp (kcal /mol*°C)
Temperature (°C)

3-3 [A1b-F/A1b-F]

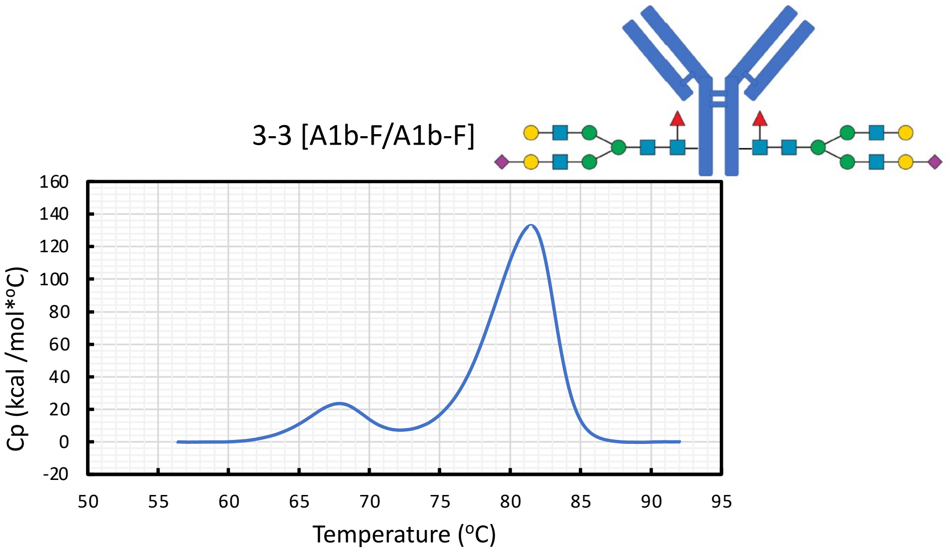

3-4 [A1b-F/A1a-Gal-F]

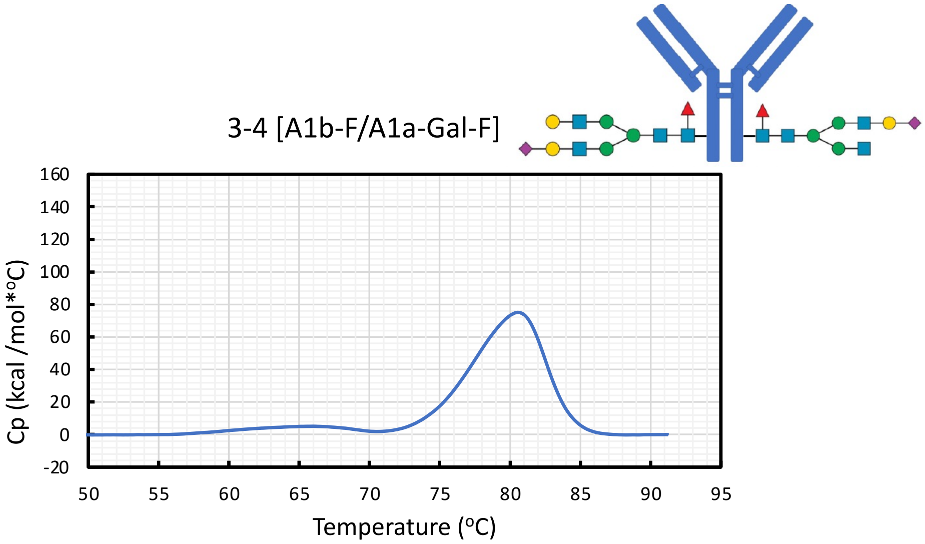

3-5 [A1b-F/A1b-Gal-F]

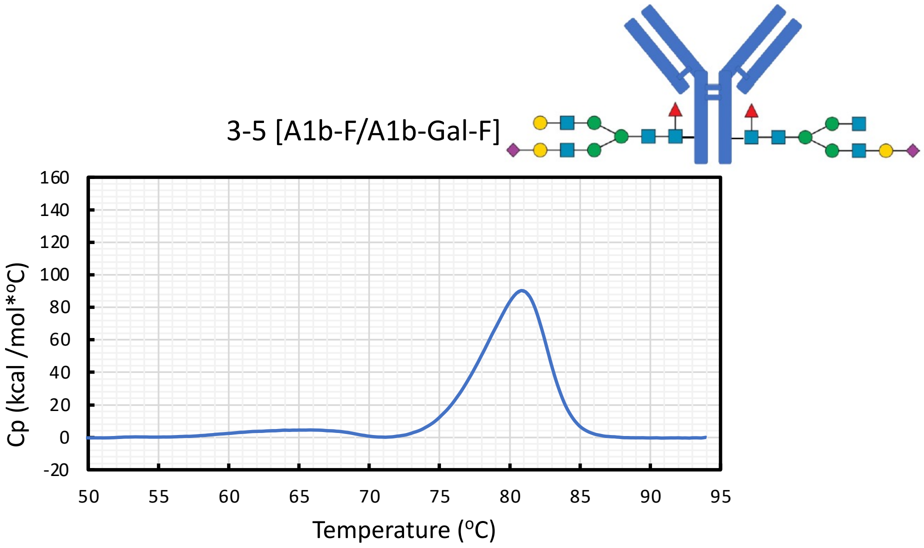

3-6 [A1b-Gal-F/G2-F]

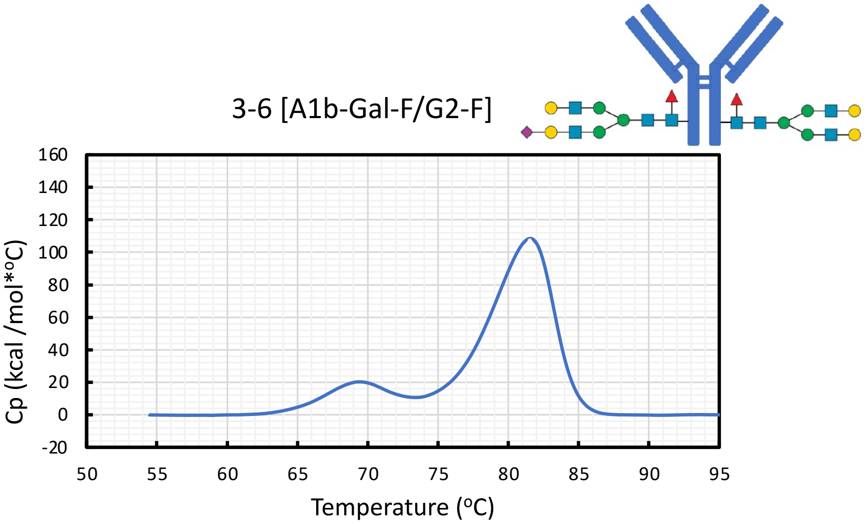

3-7 [A1b-Gal-F/G1a-F]

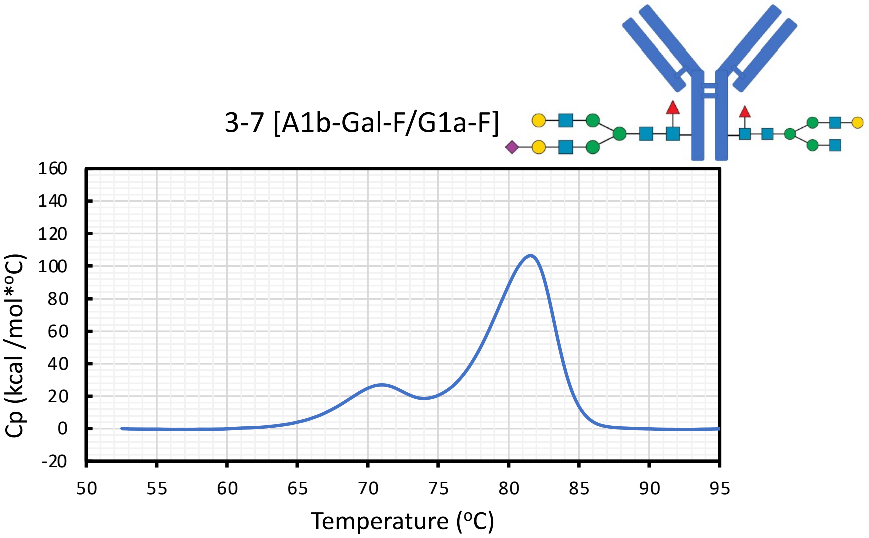

3-8 [A1b-Gal-F/G1b-F]

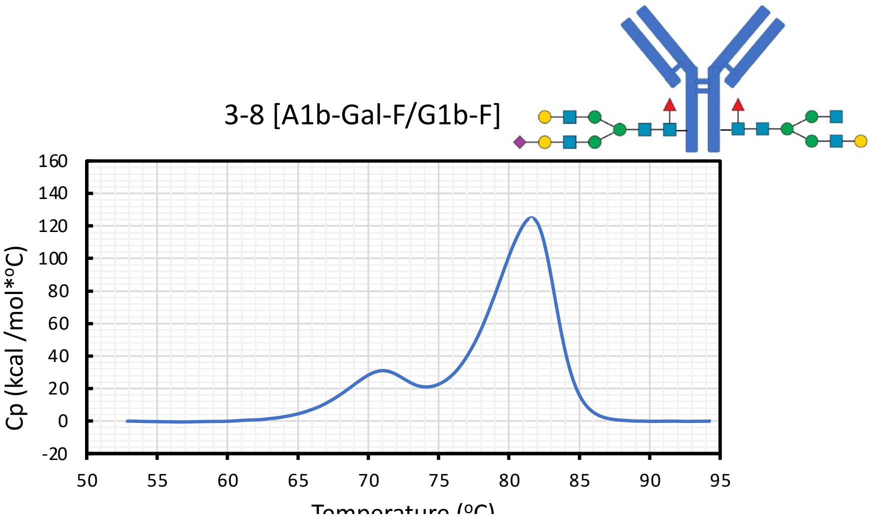

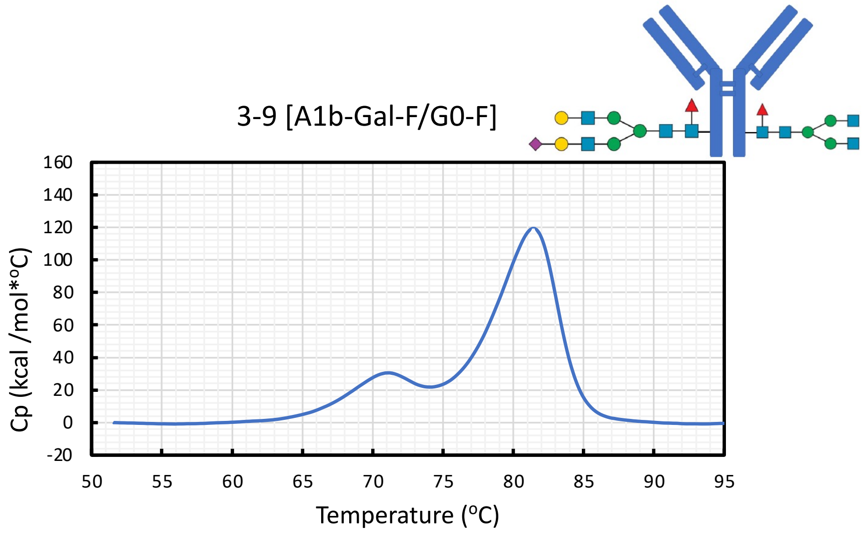
3-9 [A1b-Gal-F/G0-F]
Cp (kcal /mol*°C)
Temperature (°C)

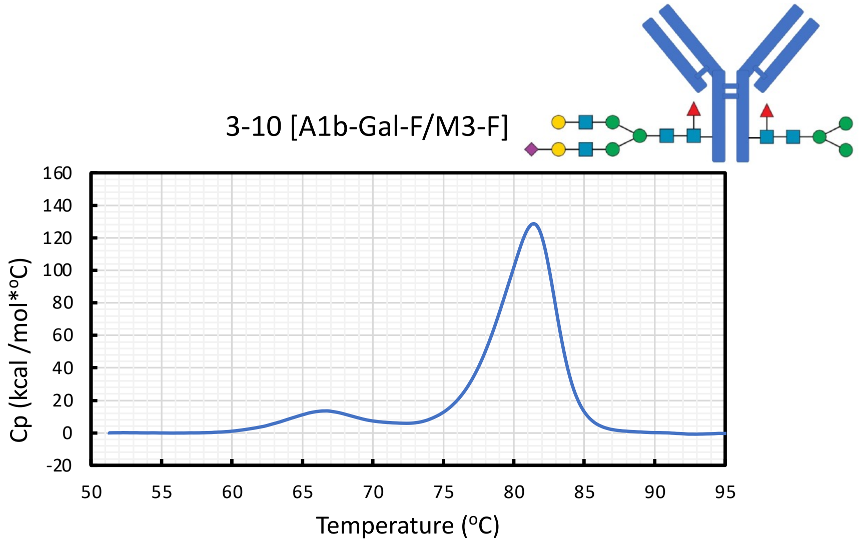
3-10 [A1b-Gal-F/M3-F]
Cp (kcal /mol*°C)
Temperature (°C)

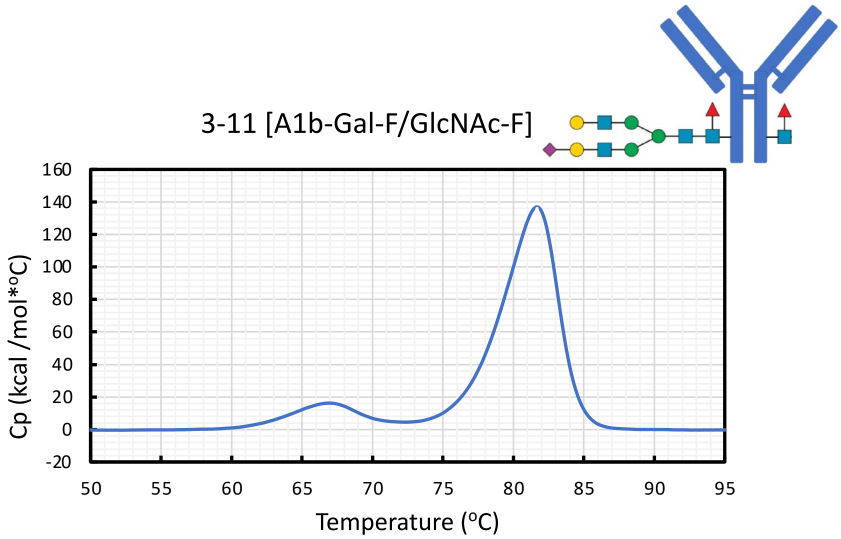
3-11 [A1b-Gal-F/GlcNAc-F]
Cp (kcal /mol*°C)
Temperature (°C)

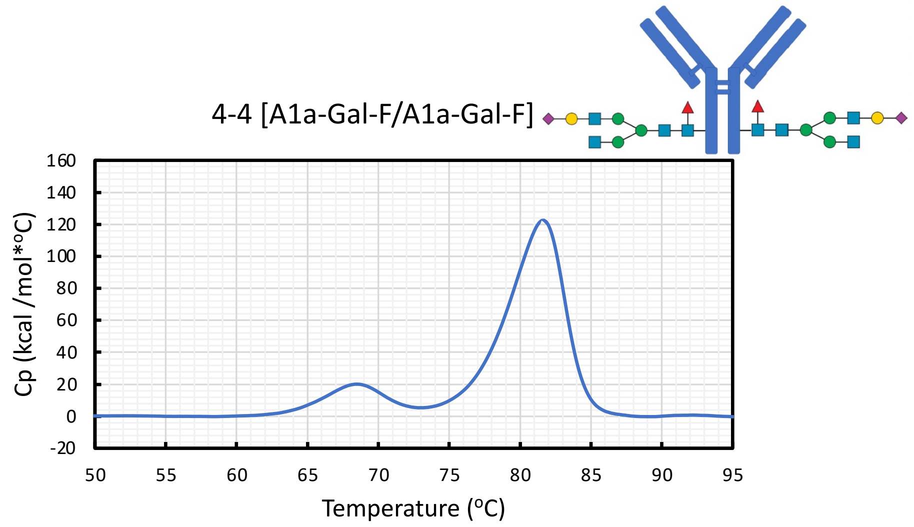
4-4 [A1a-Gal-F/A1a-Gal-F]
Cp (kcal /mol*°C)
Temperature (°C)
160
140
120
100
80
60
40
20
0
-20
50
55
60
65
70
75
80
85
90
95

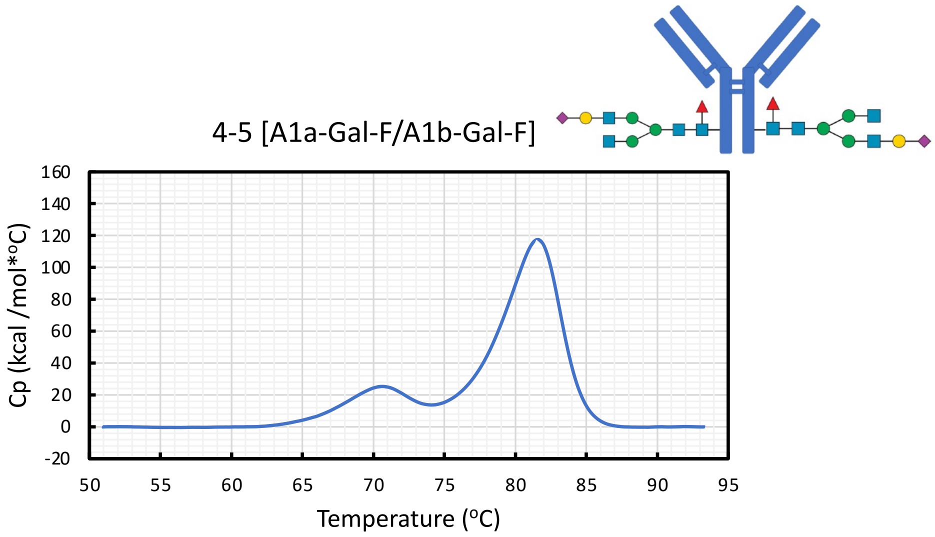
4-5 [A1a-Gal-F/A1b-Gal-F]
Cp (kcal /mol*°C)
Temperature (°C)
160
140
120
100
80
60
40
20
0
-20
50
55
60
65
70
75
80
85
90
95

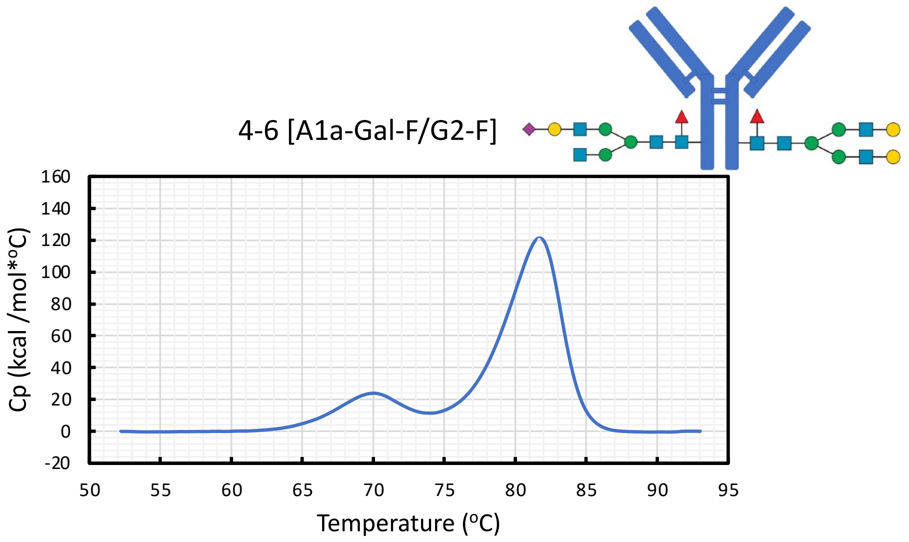
4-6 [A1a-Gal-F/G2-F]
Cp (kcal /mol*°C)
Temperature (°C)
160
140
120
100
80
60
40
20
0
-20
50
55
60
65
70
75
80
85
90
95

4-7 [A1a-Gal-F/G1a-F]

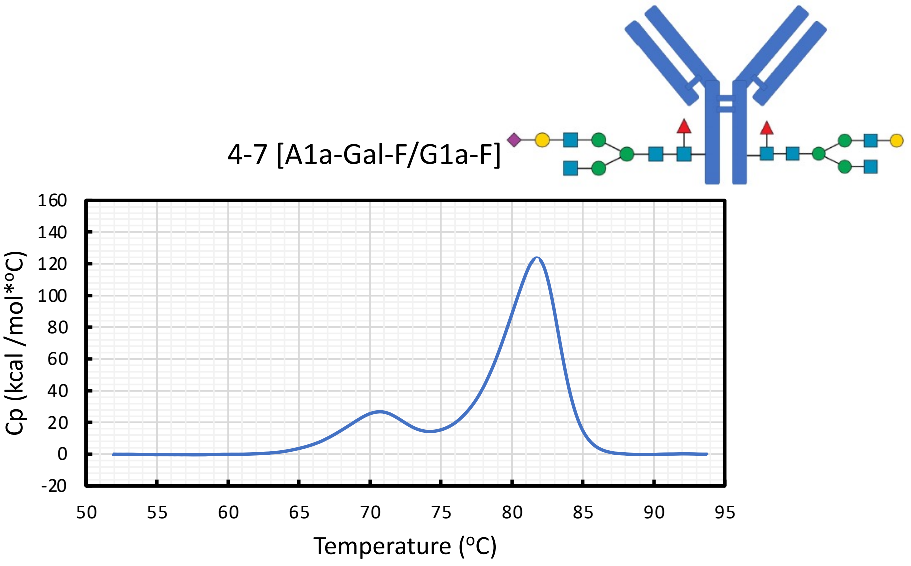

4-8 [A1a-Gal-F/G1b-F]

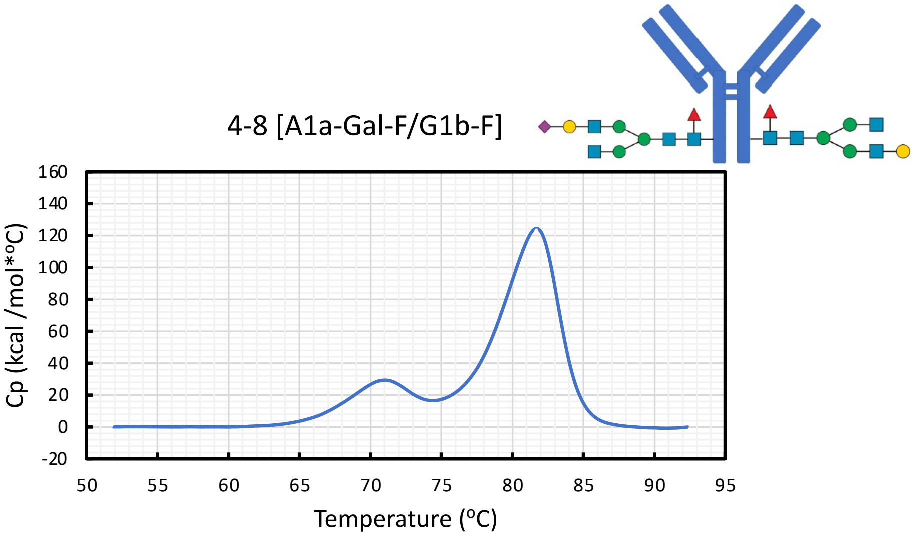

4-9 [A1a-Gal-F/G0-F]

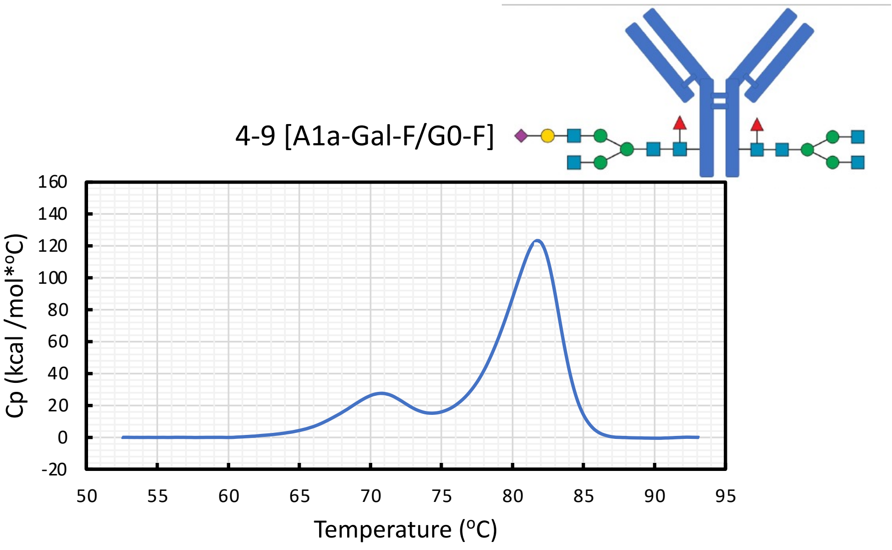

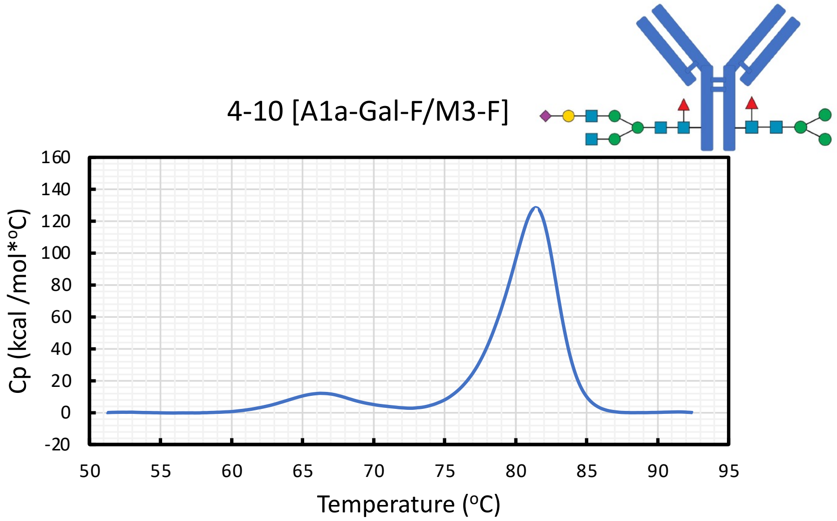

4-10 [A1a-Gal-F/M3-F]

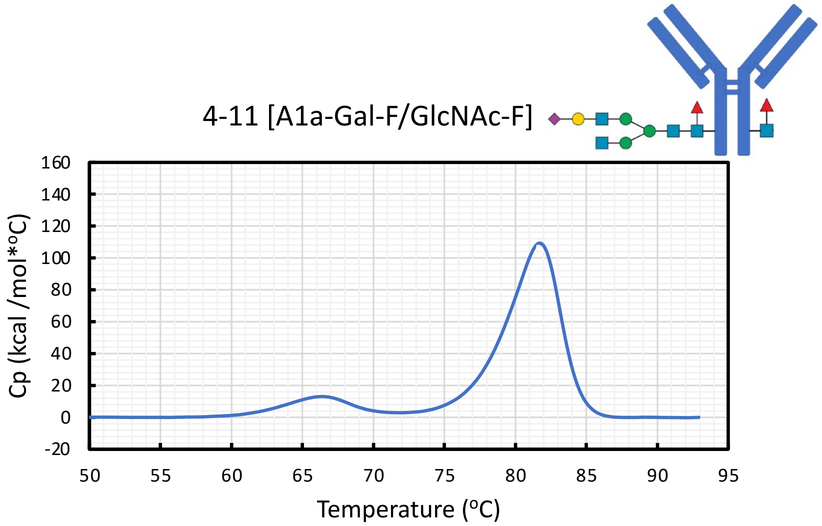

4-11 [A1a-Gal-F/GlcNAc-F]

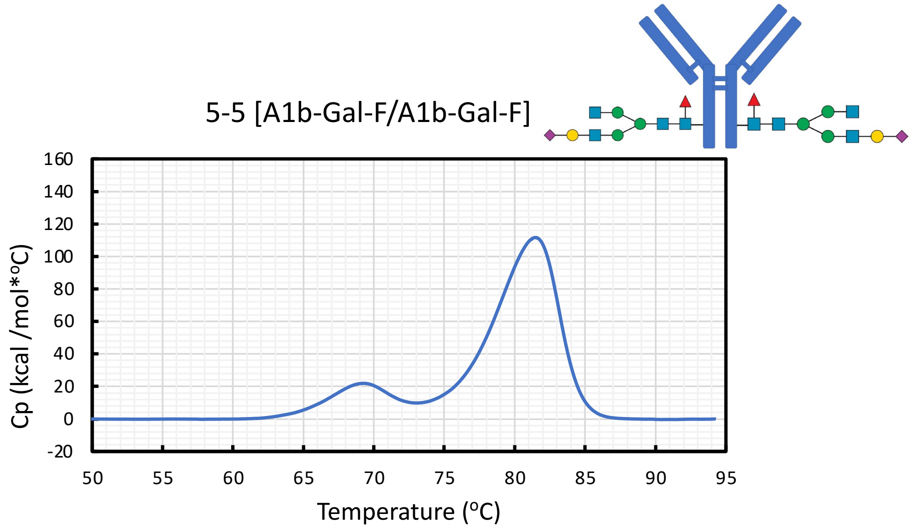

5-5 [A1b-Gal-F/A1b-Gal-F]

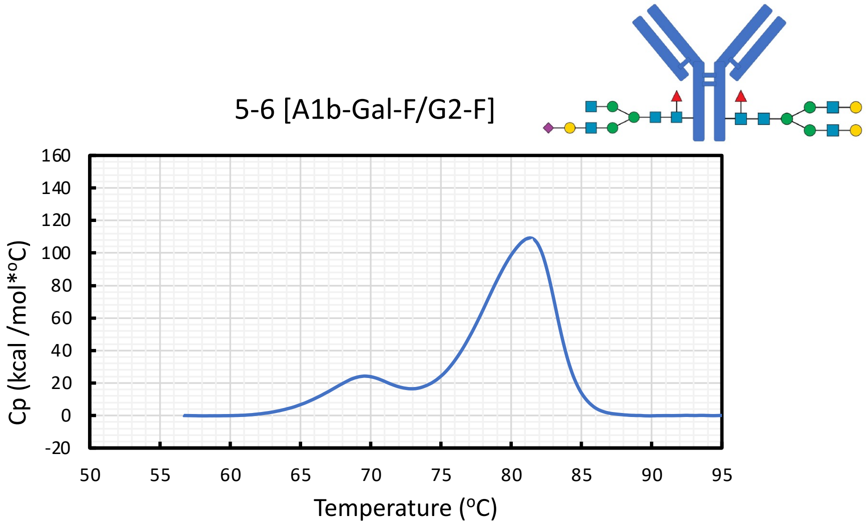

5-6 [A1b-Gal-F/G2-F]
Cp (kcal /mol*°C)
Temperature (°C)

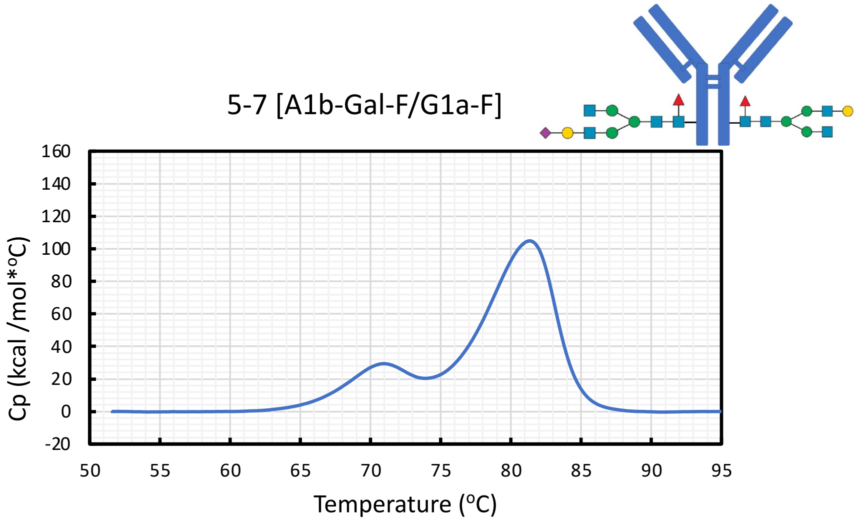

5-7 [A1b-Gal-F/G1a-F]
Cp (kcal /mol*°C)
Temperature (°C)

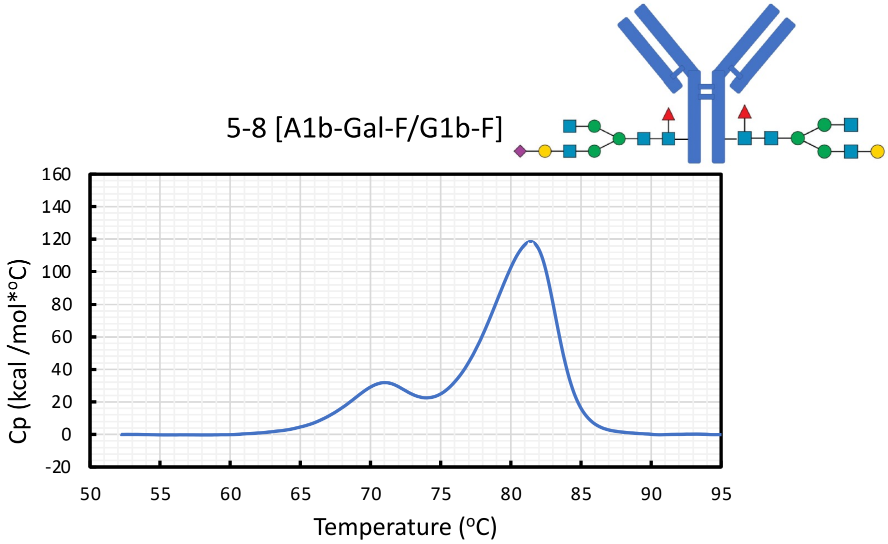

5-8 [A1b-Gal-F/G1b-F]
Cp (kcal /mol*°C)
Temperature (°C)

5-9 [A1b-Gal-F/G0-F]

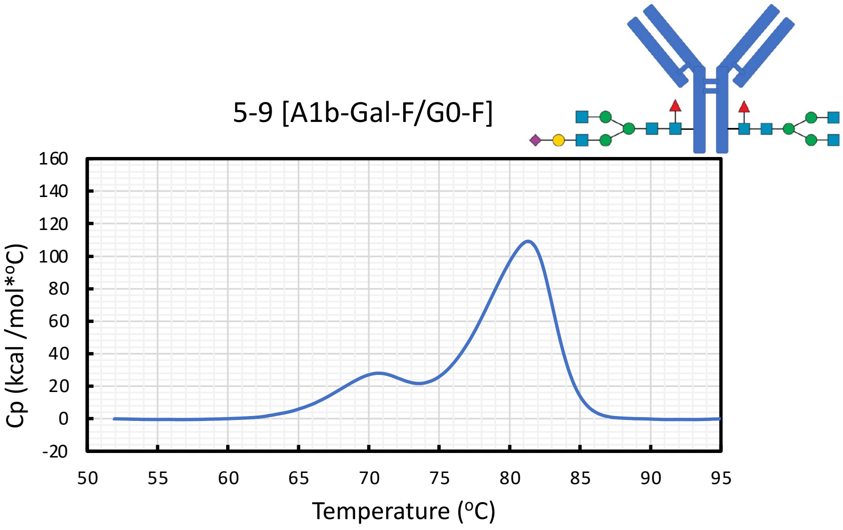

5-10 [A1b-Gal-F/M3-F]

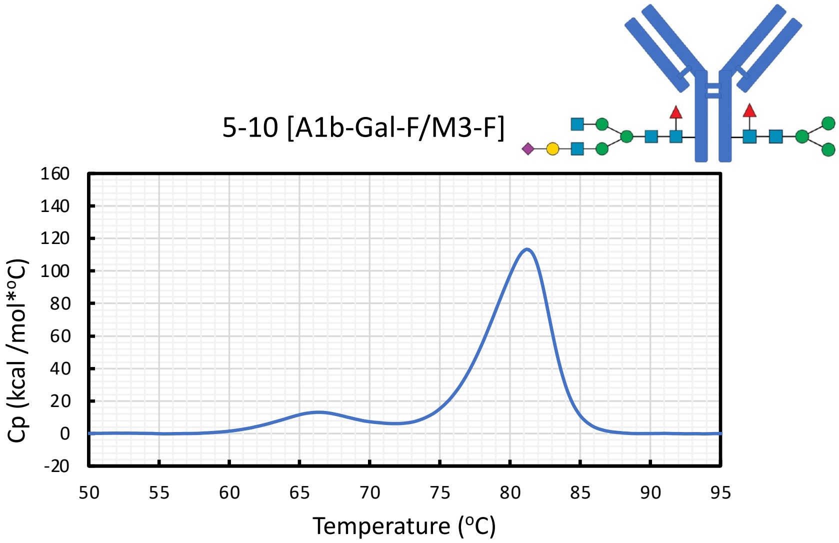

5-11 [A1b-Gal-F/GlcNAc-F]

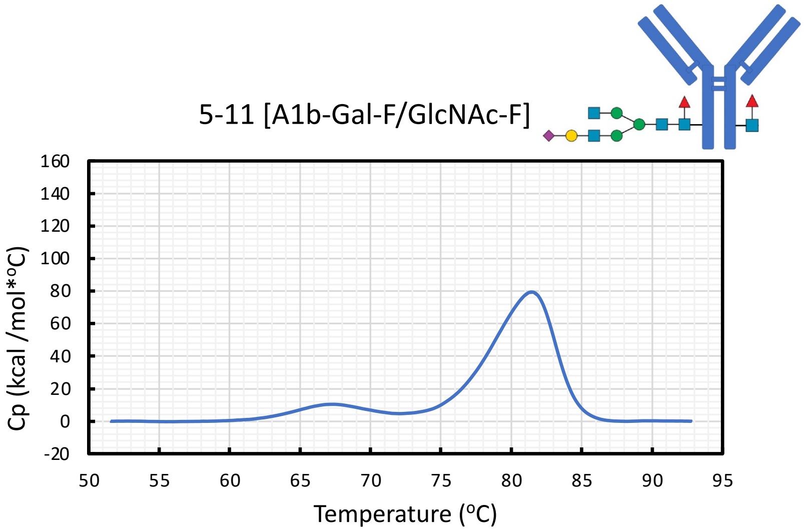

6-6 [G2-F/G2-F]

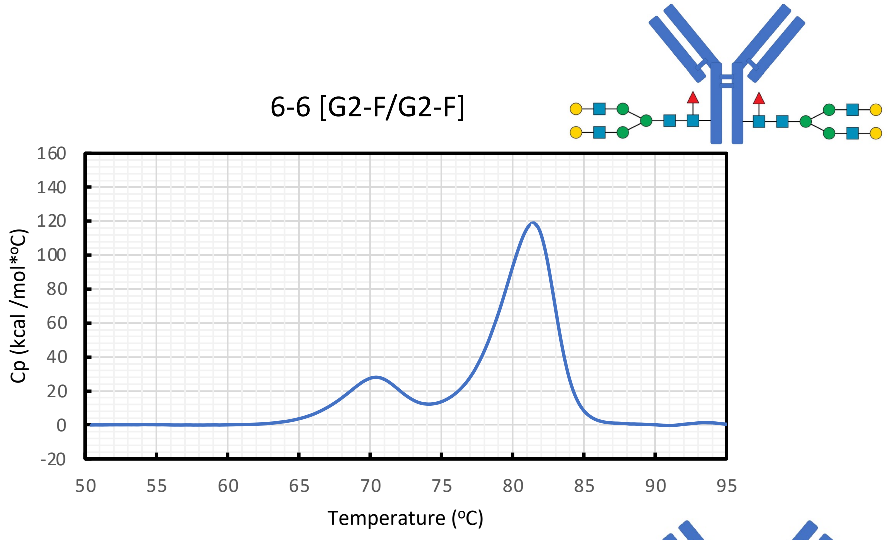

6-7 [G2-F/G1a-F]

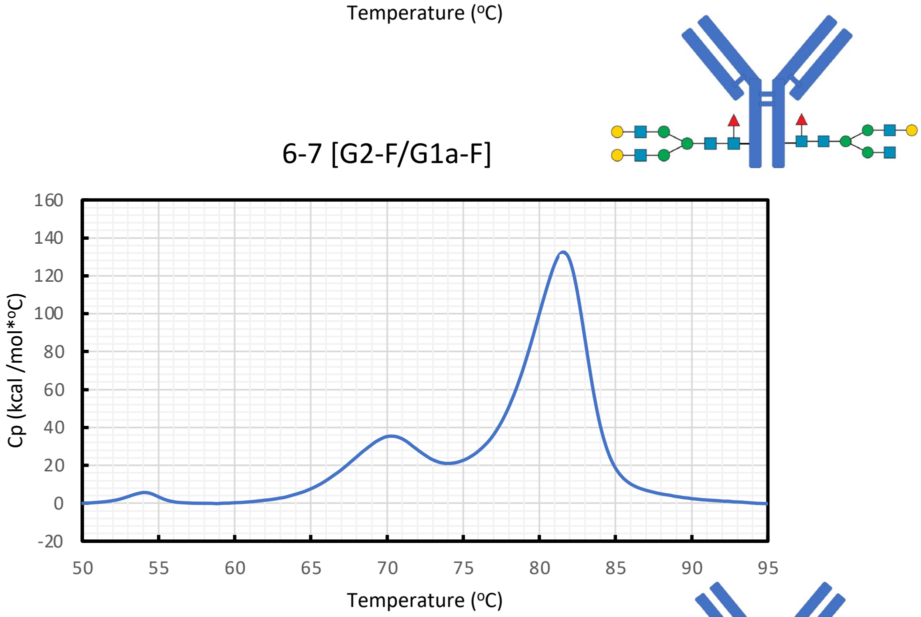

6-8 [G2-F/G1b-F]

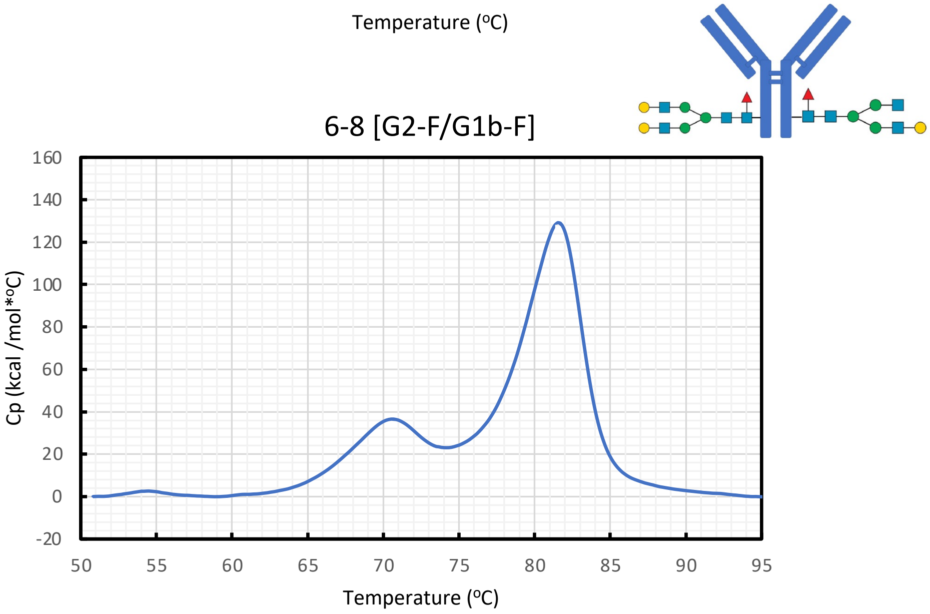

6-9 [G2-F/G0-F]

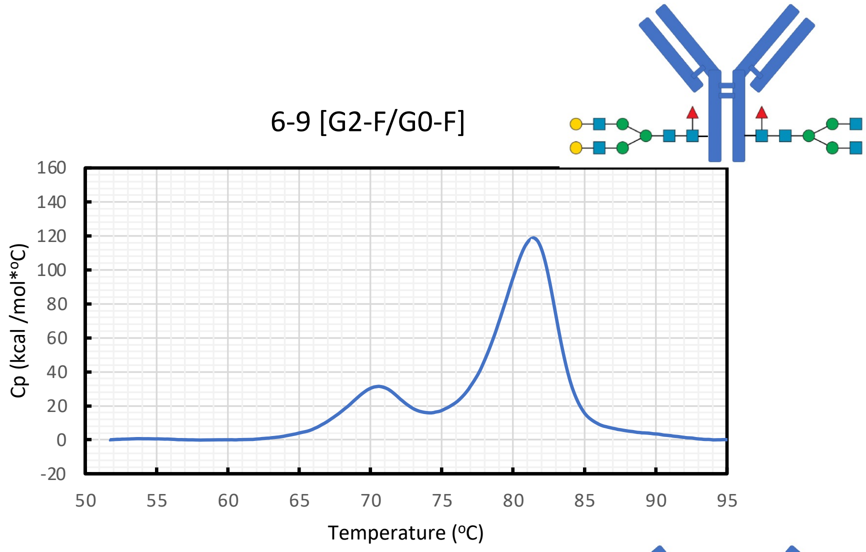

6-10 [G2-F/Man-F]

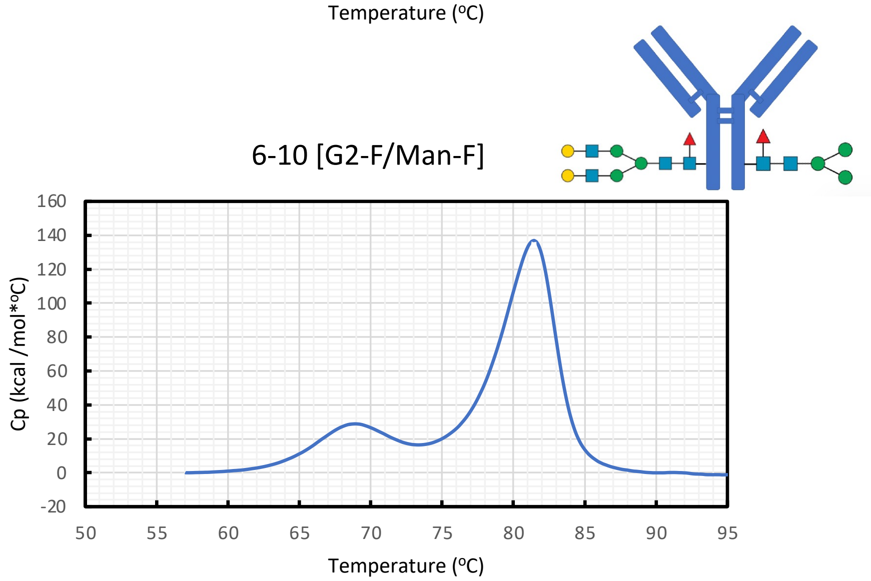

6-11 [G2-F/GlcNAc-F]

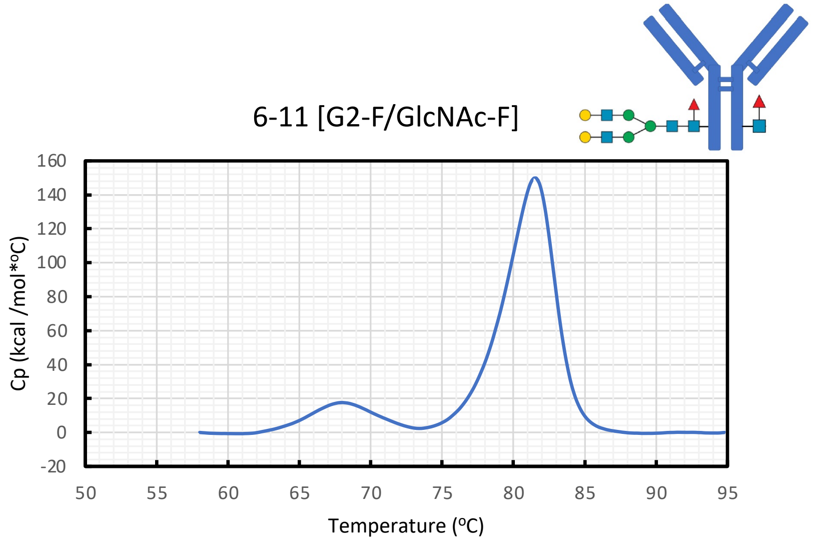

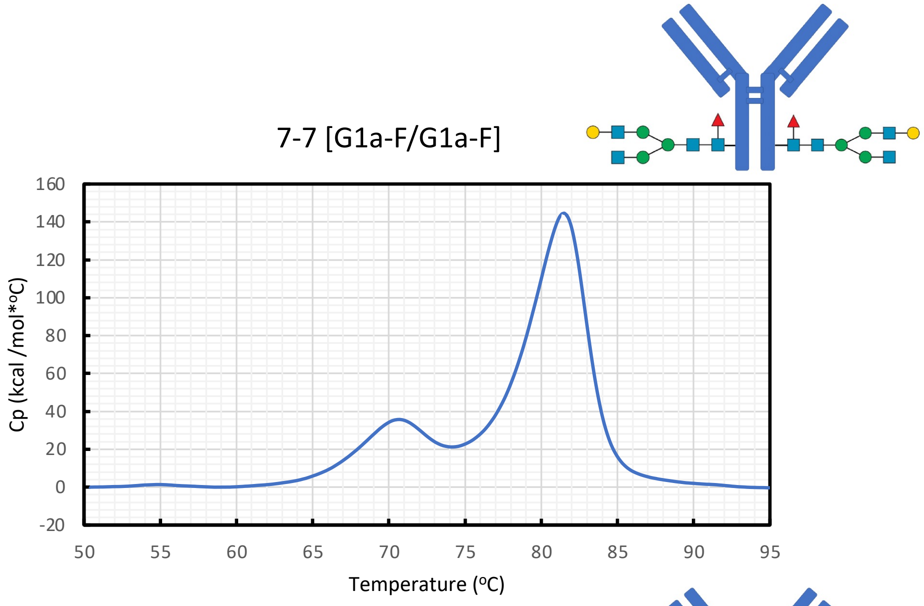
7-7 [G1a-F/G1a-F]
Cp (kcal /mol*ºC)
Temperature (ºC)
160
140
120
100
80
60
40
20
0
-20
50
55
60
65
70
75
80
85
90
95

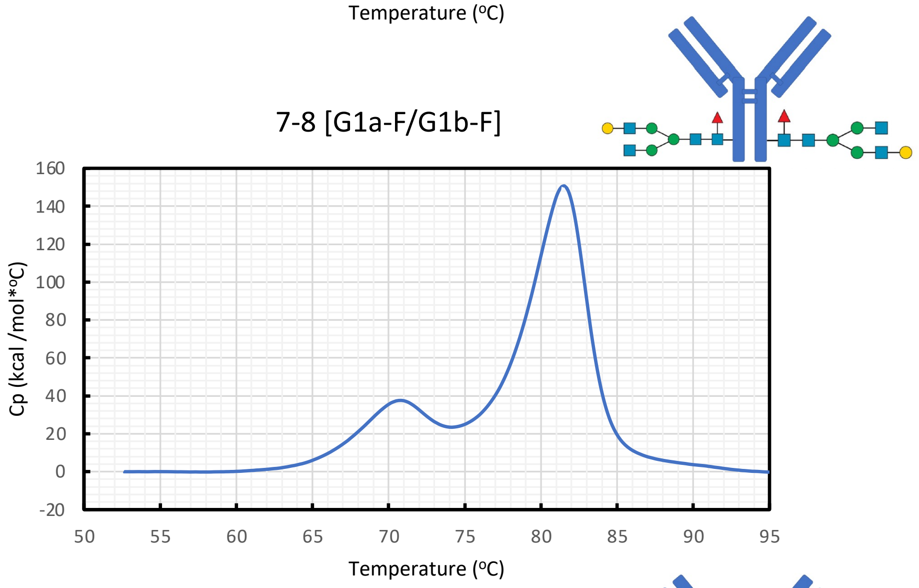
7-8 [G1a-F/G1b-F]
Cp (kcal /mol*ºC)
Temperature (ºC)
160
140
120
100
80
60
40
20
0
-20
50
55
60
65
70
75
80
85
90
95

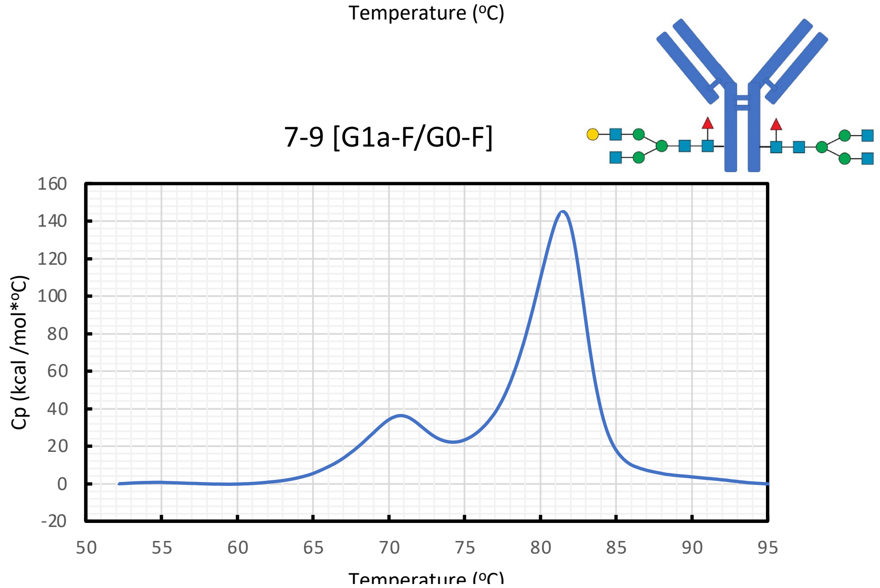
7-9 [G1a-F/G0-F]
Cp (kcal /mol*ºC)
160
140
120
100
80
60
40
20
0
-20
50
55
60
65
70
75
80
85
90
95

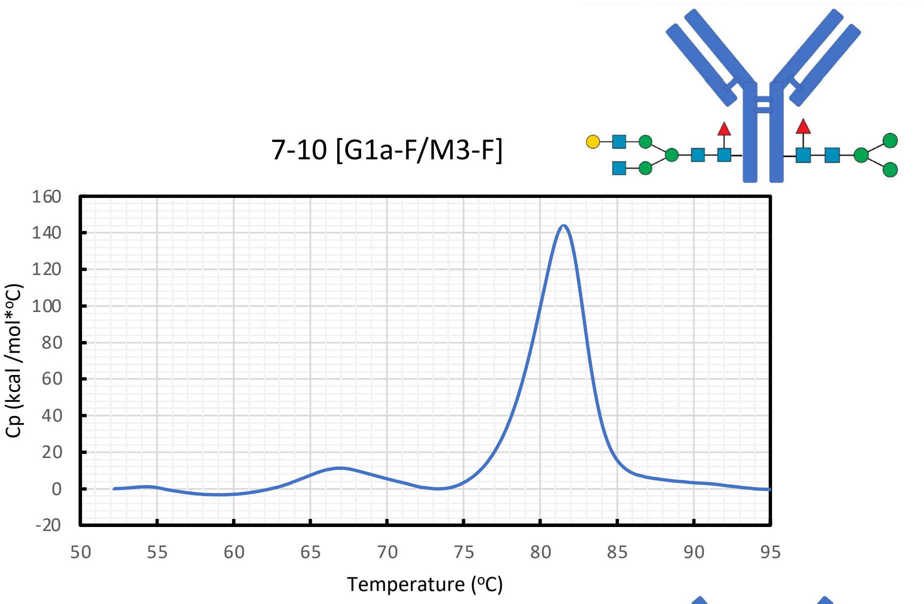
7-10 [G1a-F/M3-F]
Cp (kcal /mol*ºC)
Temperature (ºC)

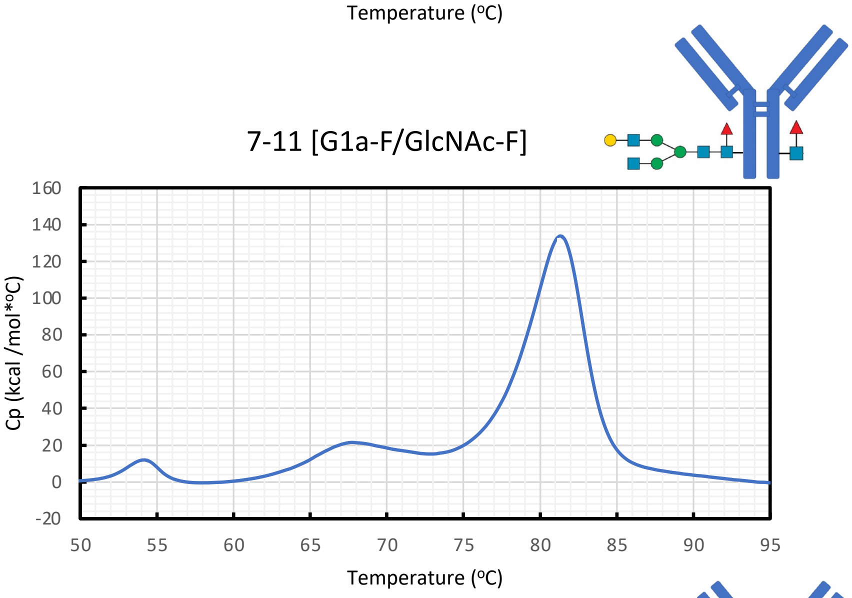
7-11 [G1a-F/GlcNAc-F]
Cp (kcal /mol*ºC)
Temperature (ºC)

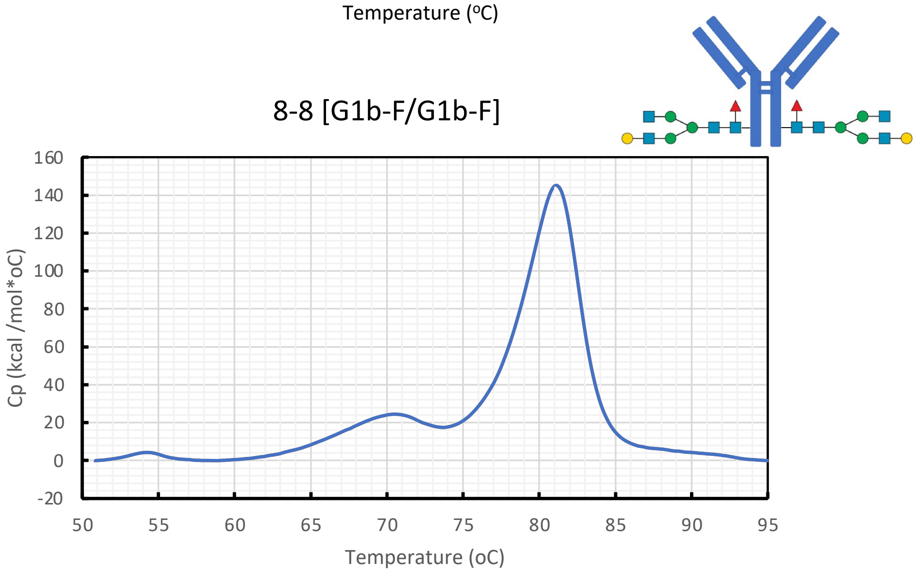
8-8 [G1b-F/G1b-F]
Cp (kcal /mol*oC)
Temperature (oC)

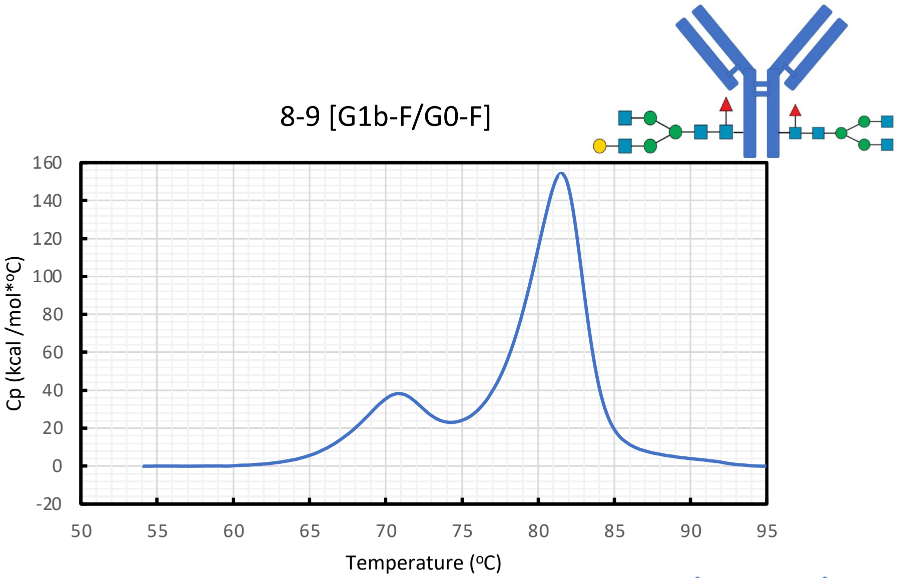
8-9 [G1b-F/G0-F]
Cp (kcal /mol*ºC)
Temperature (ºC)

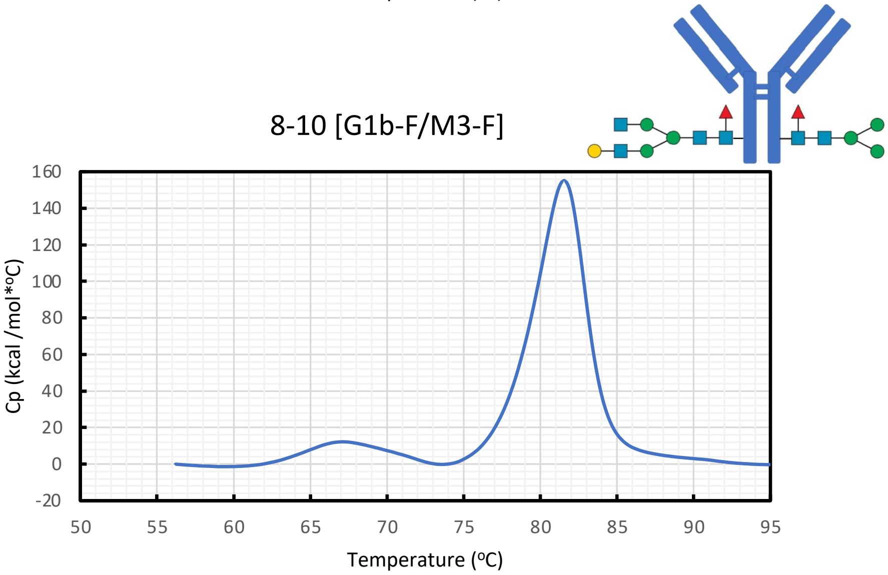
8-10 [G1b-F/M3-F]
Cp (kcal /mol*ºC)
Temperature (ºC)

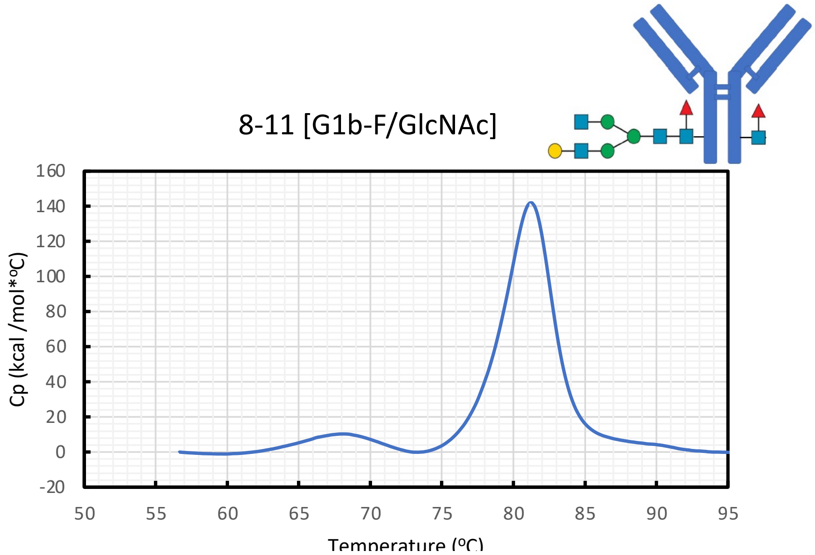
8-11 [G1b-F/GlcNAc]
Cp (kcal /mol*ºC)
Temperature (ºC)

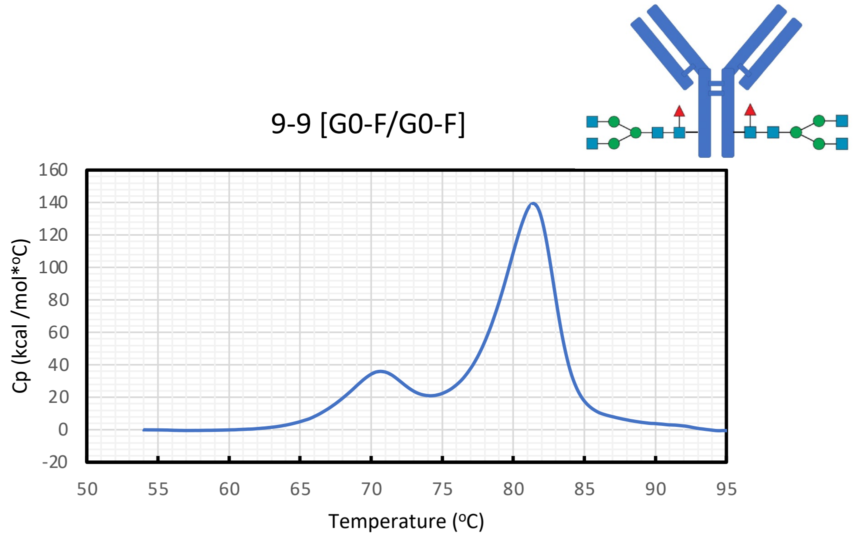
9-9 [G0-F/G0-F]
Cp (kcal /mol*ºC)
Temperature (ºC)

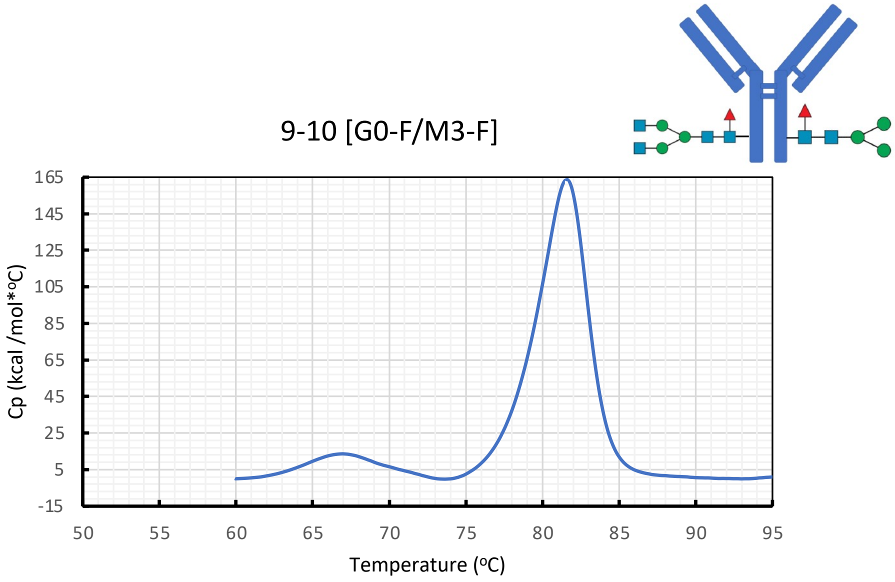
9-10 [G0-F/M3-F]
Cp (kcal /mol*ºC)
Temperature (ºC)

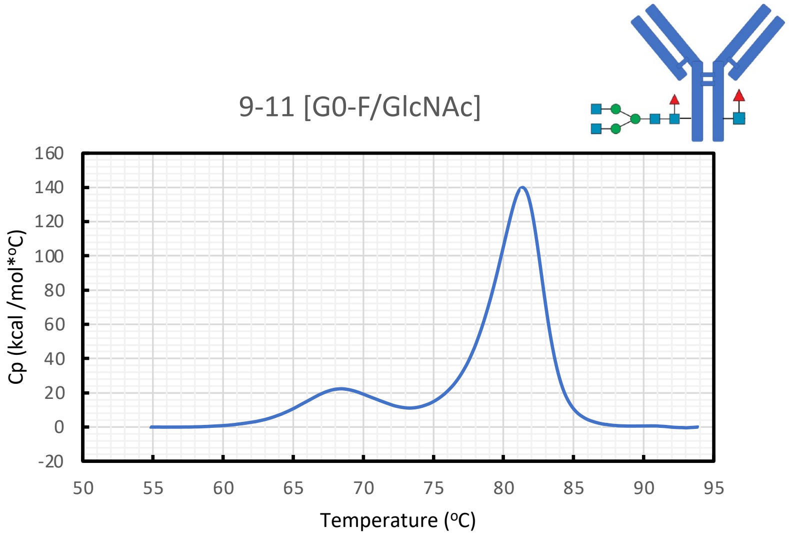
9-11 [G0-F/GlcNAc]
Cp (kcal /mol*ºC)
Temperature (ºC)

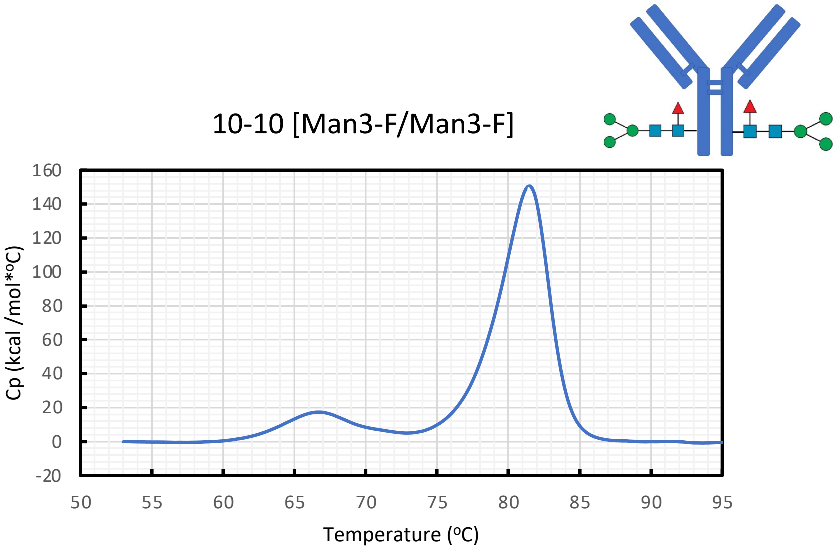

10-10 [Man3-F/Man3-F]
Cp (kcal /mol*ºC)
Temperature (ºC)

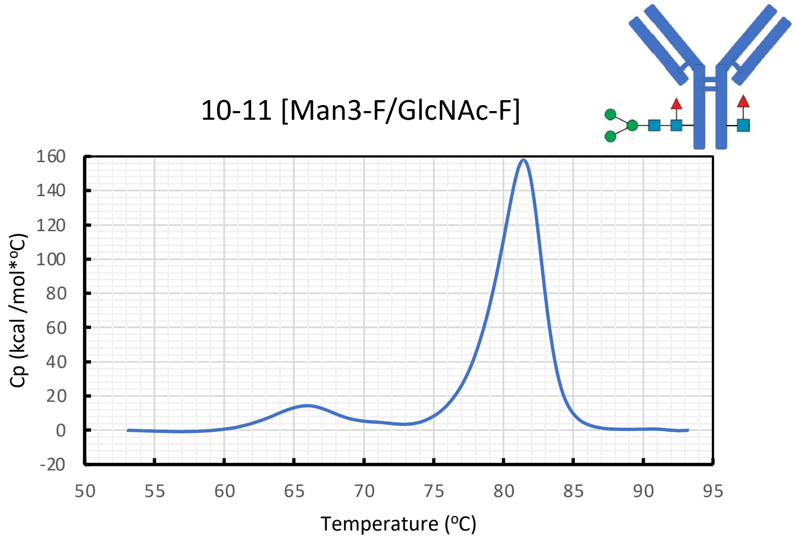

10-11 [Man3-F/GlcNAc-F]
Cp (kcal /mol*ºC)
Temperature (ºC)

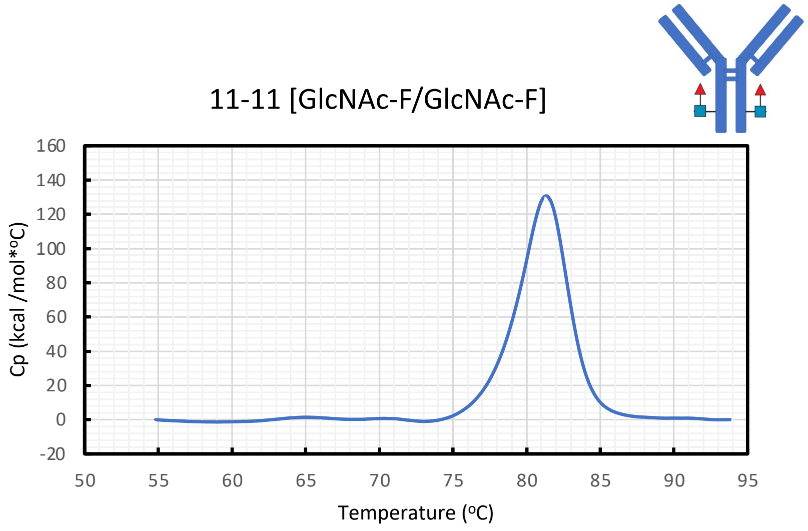

11-11 [GlcNAc-F/GlcNAc-F]
Cp (kcal /mol*ºC)
Temperature (ºC)
